# Bridging Heterocycle-Mediated Hydrogen Bonding Facilitates Permeability of Polar Macrobicycles

**DOI:** 10.1021/jacs.6c01490

**Published:** 2026-04-15

**Authors:** Gabriella I. D. Cooper, Botao Dai, Noah Durham, Gilbert L. Walker, Hongyi Yu, Angel Mendoza, Salvador J. Bernardino, Yun-Dong Wu, Patrick G. Harran

**Affiliations:** † Department of Chemistry and Biochemistry, University of California, Los Angeles, Los Angeles, California 90095, United States; ‡ School of Chemical Biology and Biotechnology, 429362 Peking University Shenzhen Graduate School, Shenzhen 518055, China; § Institute of Chemical Biology, Shenzhen Bay Laboratory, Shenzhen 518132, China; ∥ College of Chemistry and Molecular Engineering, Peking University, Beijing 100871, China

## Abstract

Octafluorocyclopentene engages linear, unprotected peptides in relative-rate-controlled polysubstitution cascades. In one flask at ambient temperature, the reactions generate stable, fluoro-crowned macrobicyclic structures that display dual-loop surfaces. A subset of these molecules was found to have unusually high membrane permeability, as measured by PAMPA. Structures with a bridging imidazole unit were overrepresented in this group. To probe this finding, a larger set of compounds was synthesized wherein peripheral functionality and the bridging residue were incrementally varied. Crystallographic data, NMR studies, and MD simulations indicate the imidazole-bridged structures adopt conformations stabilized by internal H-bonding. The heterocycle further serves to occlude cavity water and allows macrobicycles harboring polar residues, such as serine and aspartate, to retain passive permeability. Calculations reveal a quantity termed ‘desolvation cost efficiency’ that is predictive of PAMPA performance. This parameter may be leveraged for the *de novo* design of polar peptidomimetics that can enter cells passively.

## Graphical abstract

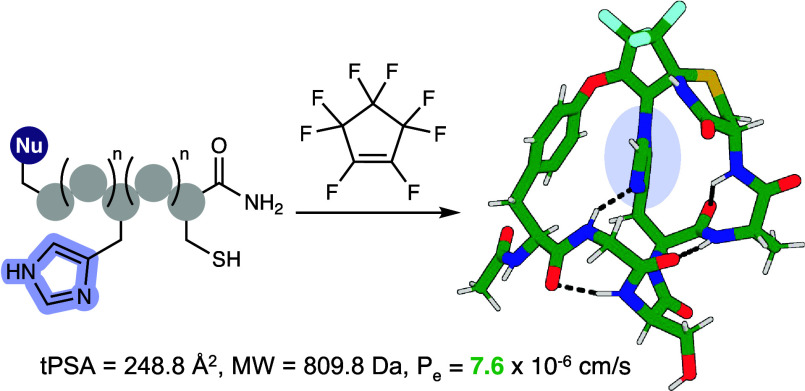

## Introduction

Laboratories across the world, both in academia and the private sector, are studying cyclic peptides and peptidomimetics. These molecules serve as leads in drug discovery, as probe compounds in chemical biology, and as building blocks in materials science.^1,2^ With the development of synthesis and screening technologies, such as mRNA display and DNA-encoded libraries (DELs), these macrocycles have demonstrated unparalleled potential in targeting ‘undruggable’ intracellular protein–protein interactions (PPIs).^1,3^ Attempts to refine the properties of cyclic peptides is an equally active area. Because they generally fail to passively cross cell membranes, targeting intracellular receptors can be a challenge. There has been a massive effort to understand factors governing this property, in hopes of designing cell-permeable analogs.^4^ The benchmark for these studies has been the natural product cyclosporin (Figure 1A).^5^ Not only does this immunosuppressive drug traverse cell membranes, it can be dosed orally. Oral cyclosporin achieves ∼30% bioavailability despite a molecular mass of 1202 Da and polar surface area (PSA) of 152.40 Å^2^.^5,6^ Biophysical data suggest the molecule can reduce the free energy barrier for membrane translocation by altering its conformation to match the polarity of the environment.^7–9^ This so-called “chameleonic effect” sees membranous cyclosporin masking backbone polarity by transannular hydrogen bonding (THB) (Figure 1A).^9,10^ Emulating this capability has become a common design principle. For example, peptide stapling using Grubbs metathesis results in THB stabilized α-helical conformers with improved permeability.^11^ Further, Baker has computationally designed permeable head-to-tail macrocycles by identifying cyclopeptide sequences whose predominate conformers are structured by internal hydrogen bonding.^12^ Yudin has shown that replacing amides in cyclopeptides with azole heterocycles (akin to backbone desiccation observed in peptide natural products)^13^ rigidifies and stabilizes H-bond shielded conformers, improving passive permeability.^14^ Other facets of cyclosporin’s structure include extensive *N*-methylation and a hydrophobic periphery. This has led to the concept of lipophilic permeability efficacy (LPE), which relates two crucial factors for achieving permeable cyclopeptides: aqueous solubility and membrane partitioning.^15^ LPE demonstrates a high degree of linearity between permeability rates obtained from the parallel artificial membrane permeability assay (PAMPA, log*P*_e_) for compounds within the lipophilicity range of ALogP ≈ 0.5–3. Lokey has leveraged this metric to design numerous highly permeable lipophilic cyclic peptides following the cyclosporin model.^16–18^

**Figure 1:**
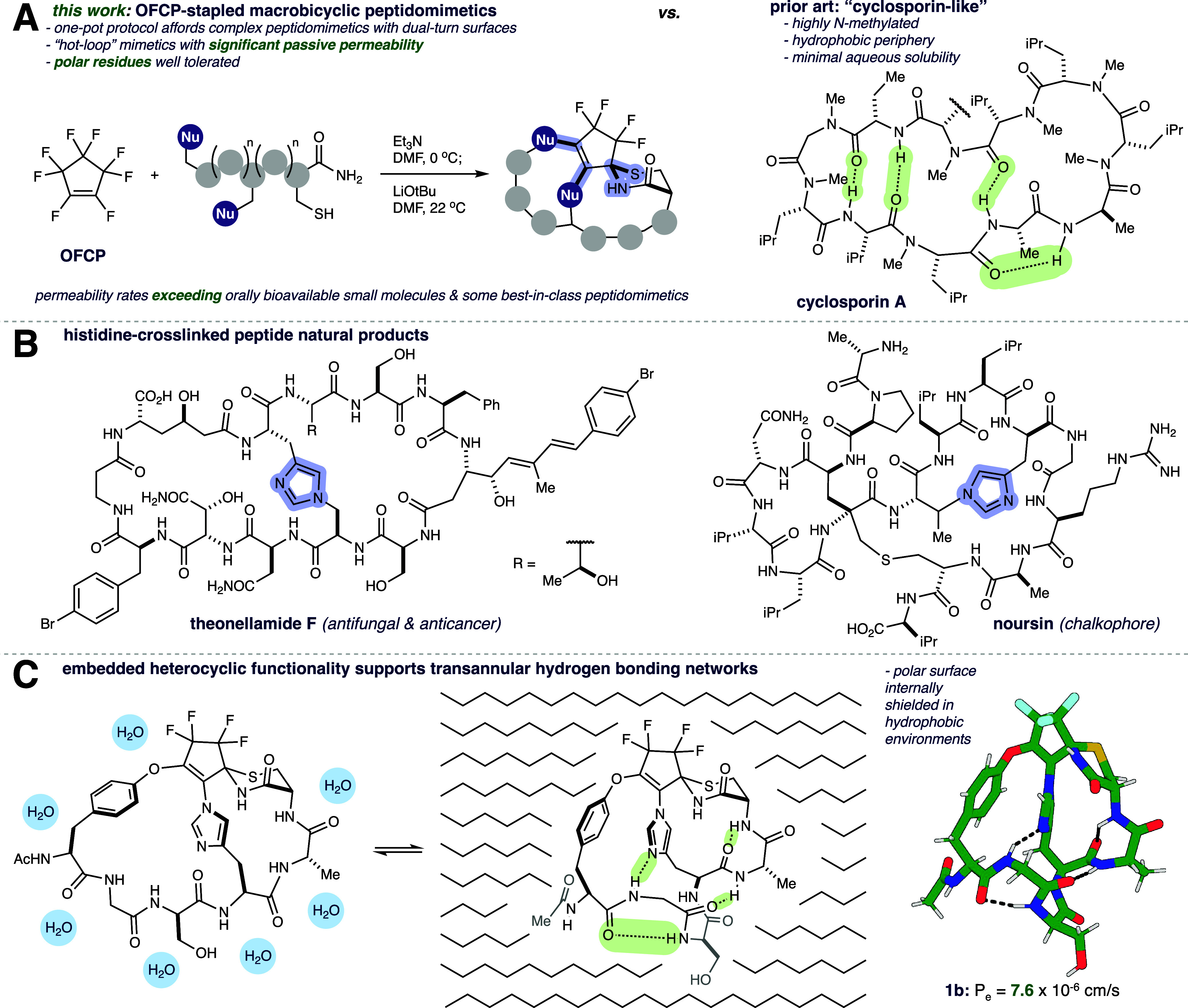
(A) Polysubstitution cascade of OFCP with unprotected linear peptides yields fluoro-crowned macrobicycles with dual-loop surfaces. OFCP-derived macrobicycles maintain high passive permeability rates, while tolerating high polarity. In contrast, ‘cyclosporin-like’ peptidomimetics attain passive permeability through high degrees of hydrophobicity and *N*-methylation. (B) Representative histidine-cross-linked peptide natural products. Reproduced with permission from ref 23. Copyright 2024 Royal Society of Chemistry. See ref 23 for additional examples. (C) Molecular dynamic simulations indicate bridging histidine scaffolding supports extensive hydrogen-bonding networks.

We recently discovered that commercial octafluorocyclopentene can engage linear unprotected peptides in relative-rate controlled polysubstitution cascades (Figure 1A).^19,20^ In one flask at ambient temperature, hyper-electrophilic OFCP^21^ can form four new bonds with the peptide, creating three new rings. OFCP undergoes two vinylic substitutions first with cysteine and then additional nucleophile (*i.e.*, histidine) to form an intermediate macrocycle. The C-terminal carboxamide then *ipso* substitutes a third fluoride, resulting in the spirocyclic thiazinone in substrate-controlled high diastereoselectivity. The final vinyl substitution of OFCP with a fourth nucleophile yields bridged macrobicycles displaying dual loop surfaces. Computations indicated a subset of those constrained rings mimic PPI mediating protein surface loops identified by Kritzer in their comprehensive analysis of the Protein Data Bank.^20,22^ Motivated by the potential of this novel compound class to serve as molecular glues for, or antagonists of, intracellular PPIs, we began to investigate their permeability characteristics.

Initial experiments revealed that several OFCP derived macrobicycles had PAMPA permeability rates exceeding *P*_e_ ≈ 1,^20^ a threshold generally considered ‘significant’ for cyclic peptides.^12,16^ A majority of those polycycles were bridged by a histidine (His) residue. Peptide-derived natural products that feature a bridging His residue (confer Figure 1B) are relatively rare, but in those cases the His bridge has been shown to contribute to bioactivity.^23^ From available data, it is not known if that contribution includes an impact on cell permeability. Nonetheless, it was apparent the bridging imidazole in our systems could be a locus for internal hydrogen bonding. It was possible we had inadvertently discovered another means by which ‘chameleonic’ behavior could be imparted to cyclic peptide derivatives.

Here we show that OFCP derived polycycles can have both high PSA and high passive permeability (Figure 1C). Using molecular dynamics (MD) simulations, we formulate a new metric to rationalize the permeability of polar polycycles. ‘Desolvation cost efficiency’ quantifies the energetic penalty of traversing a cell membrane and ranks a given molecule’s ability to shield polar surface via internal H-bonding. This parameter correlates linearly with PAMPA permeability across diverse set of OFCP derived macrobicycles. It provides a rational basis to design polar peptidomimetics that enter cells passively.

## Results and Discussion

Structures **1–3** were among the best performers in initial PAMPA assays (Figure 2A). They also reflect three distinct OFCP processing modes. Compound **1** derives from a linear sequence having a C-terminal cysteinamide. Product **2** derives from the reaction cascade being initiated at branched cysteinamide, and **3** is assembled incrementally from OFCP, a linear hexapeptide and a cysteinamine insert.^20^ A library of analogs was generated to probe structure/permeability relationships. For compound **1**, a total of 21 variants were synthesized wherein: (1) the glycine (Gly), serine (Ser), and alanine (Ala) residues were individually replaced by a selection of aspartate (Asp), asparagine (Asn), and Ser residues (see Figure 3 and SI for details); (2) the stereochemistry at the Ser and His positions was inverted; (3) the Ser residue was *N*-methylated; (4) the N-terminal acetamide was removed; (5) Gly was replaced by proline (Pro); (6) the Ser residue was removed to contract the macrocycle. For compound **2**, the stereochemistry of the bridging His was inverted, the N-terminal acetamide was removed and the Ser residue was replaced by L-*cis*-hydroxyproline (Hyp). For threonine (Thr) bridged compound **3**, 18 analogs were synthesized (see SI for details). These included *allo*-Thr and Ser variants of the bridging residue, several cysteamine insert variations, multiple modifications at the Pro position, and a one-carbon homologated variant of the thiazinone ring.

**Figure 2:**
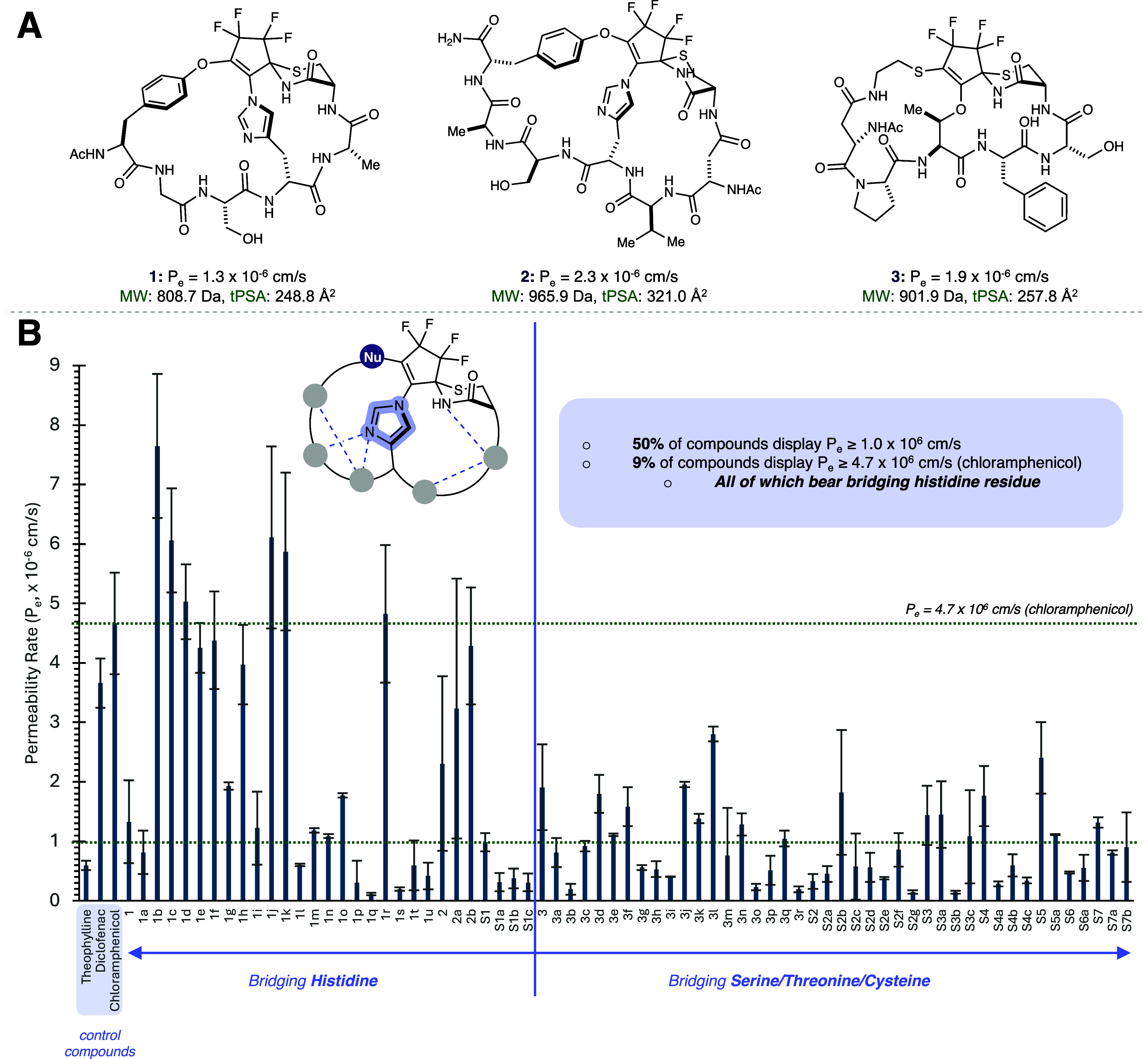
(A) Scaffolds **1–3**, identified to have promising permeability by PAMPA in our previous studies. Reproduced from ref 20. Copyright 2023 American Chemical Society. (B) Permeability measurement by PAMPA reveals bridging histidine compounds are overrepresented as highly permeable scaffolds. Theophylline, diclofenac, and chloramphenicol used as low, medium, and high permeability standards, respectively. Error bars reflect one standard deviation from seven independent PAMPA experiments, each preformed in technical duplicate. See SI for further details.

**Figure 3:**
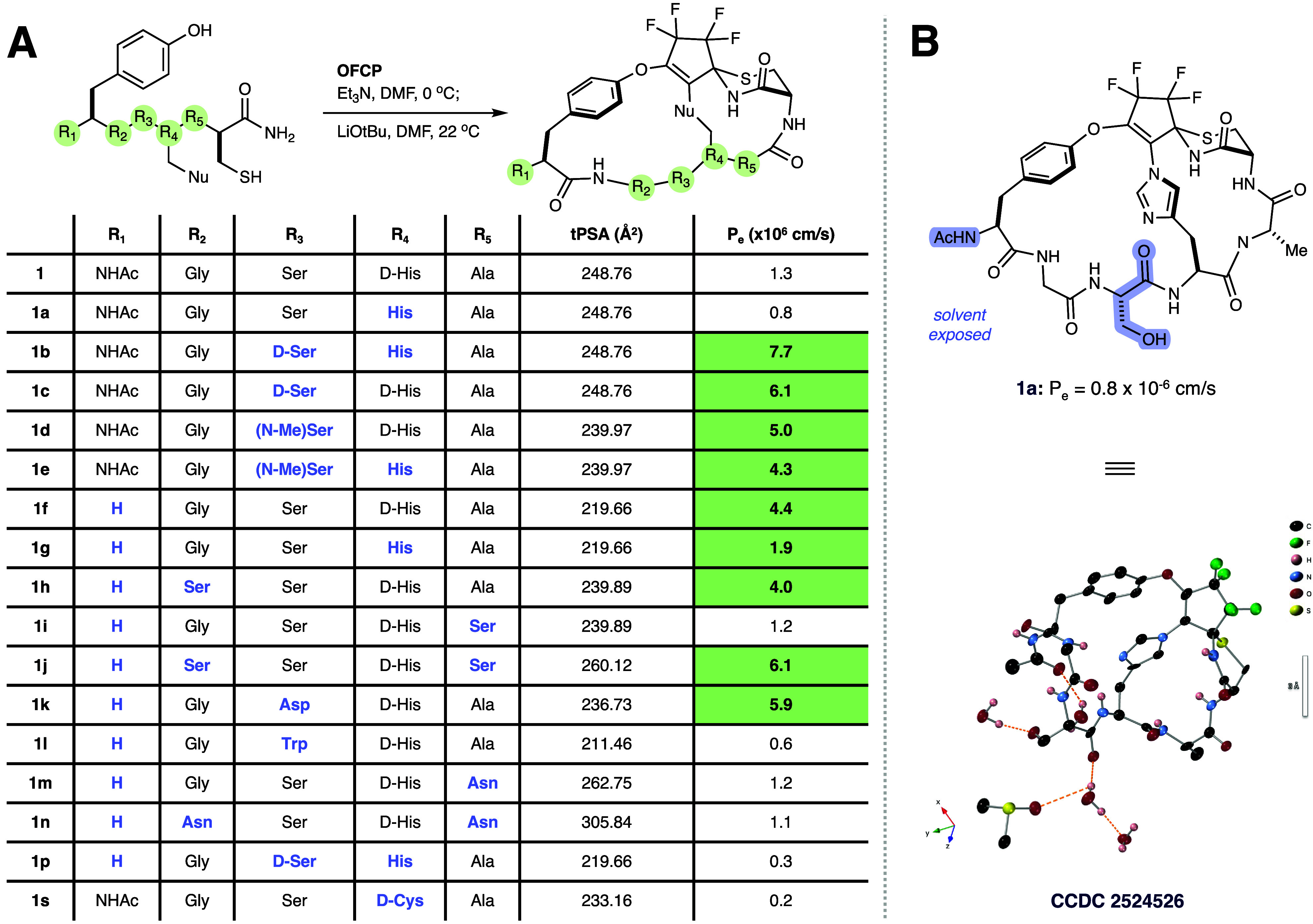
Structure–permeability relationship and crystallographic analysis of **1a** and analogs. (A) Synthesis and permeability profiling. Top: General synthetic scheme for the macrocyclization of linear precursors with OFCP. See SI for details. Bottom: Table summarizing stereochemical and residue identity variations at positions R_1_–R_5_. Unless otherwise specified, all amino acids were L-configured. Analogs exhibiting high permeability are highlighted in green, while specific structural modifications relative to the parent scaffold **1** are indicated in the blue text. The data demonstrates that the scaffold can accommodate polar side chains (e.g., **1j**, **1k**) while maintaining membrane permeability. (B) X-ray crystal structure of analog **1a** (CCDC 2524526). Single crystals were grown via vapor diffusion of 1:1 MeOH:H_2_O into a DMSO solution of compound. Top: 2D representation highlighting the solvent-exposed acetamide (AcHN−) and serine side chains. Bottom: X-ray diffraction (CrystalMaker, 50% probability thermal ellipsoids) revealing one molecule of DMSO and four molecules of H_2_O per molecule of **1a** exist in the unit cell. Intermolecular hydrogen bonds are depicted as orange dotted lines; nonpolar hydrogen atoms are omitted for clarity.

Evaluation of the complete analog set by PAMPA revealed a clear bifurcation (Figure 2B): for structures having a bridging Ser/Thr or Cys residue, modifications were generally tolerated but none resulted in significantly improved PAMPA performance. In contrast, nearly half of the His bridged variants now had permeability on par with the high permeability standard, orally bioavailable chloramphenicol.^24^ In addition, *N*-methylating the bridging imidazole or replacing it with a sulfide or 1,2,4-triazole (see SI) linkage gave the worst performers in the data set.

The identity of the bridge was a major permeability determinant, but there were others (Figure 3A). For compound **1**, inverting the stereochemistry of the bridging His from D- to L- (**1a**) had a slightly detrimental effect, but inverting the stereochemistry of the adjacent Ser residue (**1c**) or inverting both the His and Ser simultaneously resulted in a ∼6-fold increase in permeability. Single crystals of **1a** were obtained from vapor diffusion of 1:1 MeOH/H_2_O mixture into a concentrated DMSO solution of **1a**. X-ray diffraction (0.8 Å resolution) analysis showed no evidence of a trifluoroacetate (TFA) ion, despite HPLC purification using a TFA modified eluent. Further spectroscopic analysis of all the compounds lead us to conclude the products isolated were in free base form, with the basicity of the imidazole perhaps attenuated by the appended polyfluoro-cyclopentene. Further analysis of the X-ray diffraction revealed the N-terminal acetamide and Ser residue were engaged with four molecules of water and one molecule of DMSO via H-bonding (Figure 3B). This observation explains the permeability increase caused by *N*-methylating the Ser amide (**1d**) and deleting the N-terminal acetamide (**1f**). Beyond the core scaffold, side-chain compatibility showed distinct electronic and steric preferences (Figure 3A). Introducing polar Ser or Asn at the R_5_ position (**1i**, **1m**) had a detrimental effect relative to **1f**, yet the analogs still maintained permeability above *P*_e_ = 1.

Notably, at R_3_, the polar Asp residue (**1k**) enhanced permeability, whereas the bulky, hydrophobic Trp (**1l**) was detrimental. Similarly, at R_2_, Ser (**1h**) was well-tolerated while Asn (**1n**) reduced permeability. Collectively, the scaffold appears to disfavor H-bond donor-rich (Asn) and bulky hydrophobic (Trp) groups, while the H-bond acceptor-rich Asp is remarkably well-accommodated. Finally, replacing the bridging d-His with d-Cys (**1s**, Figure 3A) caused the most dramatic reduction in permeability. We turned to computations for a more comprehensive analysis of these results.

Extensive MD simulations were conducted in both polar (aqueous) and nonpolar (chloroform) environments. Traditional MD simulations often suffer from low sampling efficiency. As a remedy, we leveraged high-temperature molecular dynamics (HT-MD). Our previous studies demonstrated the high efficiency of high-temperature simulations in exploring the conformational space of cyclic peptides and protein-peptide complexes.^25^ To recover the biologically relevant ensemble at room temperature, we applied the probability density reweighting (PDR) method.^26,27^ PDR allows for the accurate reconstruction of the potential energy surface (PES) and population distributions at 300 K from the high-temperature trajectories. This method has been validated to accurately predict the structures of cyclic peptides containing proline residues and other constrained systems.^27,28^ The accuracy of the force field is paramount in predicting conformational behavior. Previous studies have indicated that general molecular force fields can accurately describe the conformational changes of permeable cyclic peptides.^8,9,29^ Accordingly, we employed the GAFF2 force field for our simulations.^30^ The method successfully predicted a top cluster model that aligned with the experimental crystal structure of **1a** with an all-heavy atom RMSD of 0.98 Å (Figure 4A), which demonstrates that our HT-MD/PDR workflow with GAFF2 is capable of accurately capturing the native states of these macrobicycles.

**Figure 4:**
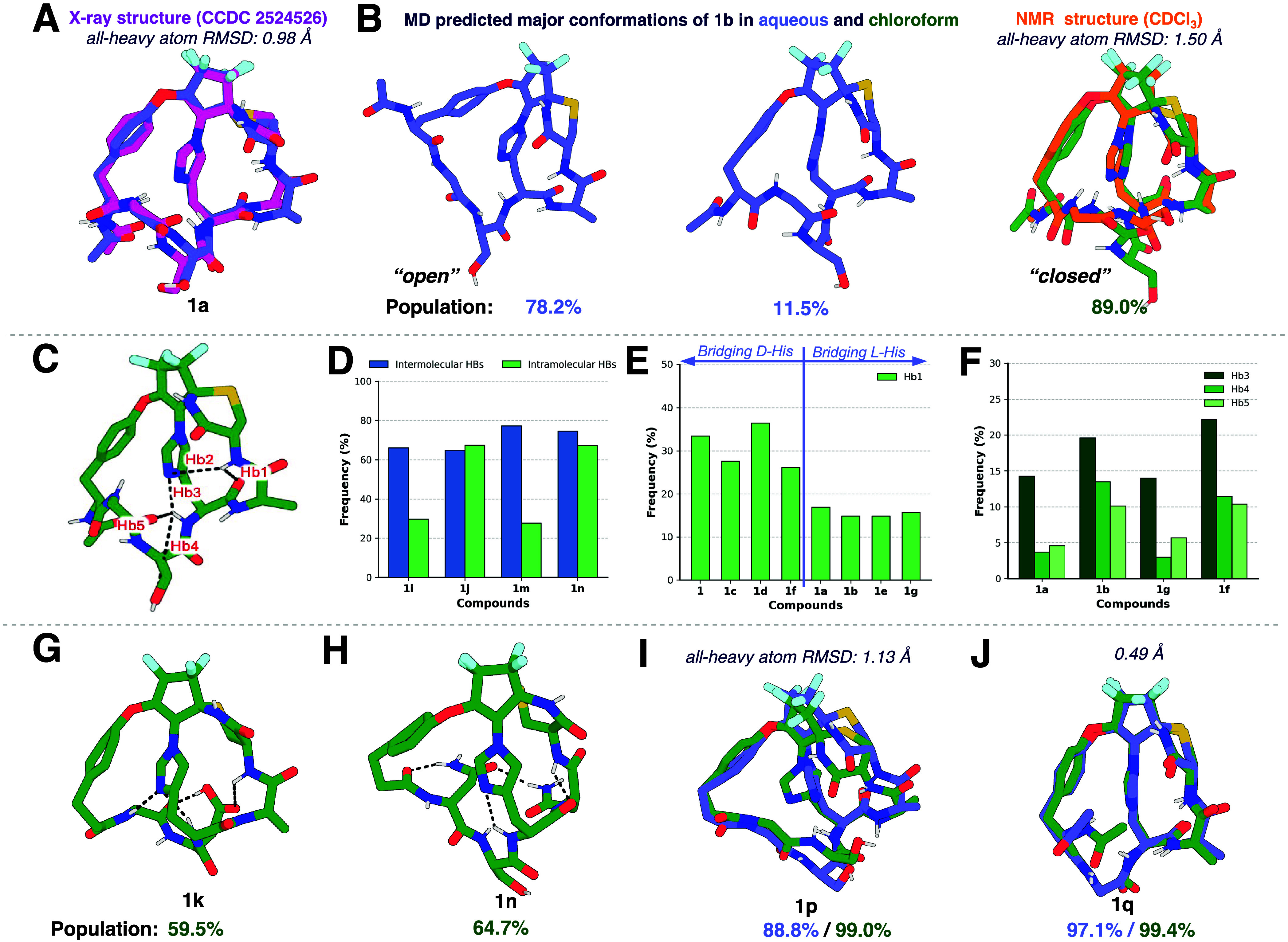
Structural validation and conformational analysis of some derivatives of **1**. (A) Superposition of the MD predicted top-ranked cluster model in aqueous (blue) and the X-ray structure (magenta) of analog **1a** (all-heavy atom RMSD = 0.98 Å). (B) Chameleonic behavior of the highly permeable analog **1b**. MD predicts both “open” (78.2%) and “closed” (11.5%) conformations for **1b** in aqueous solution (blue), but “closed” conformation (89.0%) is dominant in CHCl_3_ (green), in good agreement with the NMR structure obtained in CDCl_3_ (NOESY distance restraints, orange, see SI). (C) The MD predicted IMHBs between bridging-His and other residues within the compound **1b**. Specific H-bonding indices are highlighted in red. (D) Analysis of solvent exposure vs internal stabilization. Blue bars (intermolecular HBs): Frequency of H-bonds formed between the polar side chain at the R_5_ position (Ser/Asn) and bulk water molecules. Green bars (intramolecular HBs): Frequency of internal H-bonds formed between residues at the R_2_ and R_3_ positions. (E) Quantitative analysis of the Hb1 populations across compounds **1–1g**. (F) H-bonding analysis of Hb3–5 of compounds **1a**, **1b**, **1g**, and **1f**. (G) “Closed” conformation of **1k** in chloroform shows the increased H-bonds between the Asp carboxylate and the bicyclic scaffold. (H) Although the introduction of hydrophilic Asn in **1n** can facilitate more internal H-bonds, the excess hydrogen donors remains exposed to the solvent. (I) Poorly permeable analog **1p** is rigid in water (88.8% single population), and the conformation does not change in CHCl_3_. (J) Analog **1q** exhibits poor permeability (*P*_e_ = 0.1 × 10^–6^ cm/s) and is locked in a single “open” conformation in both aqueous and chloroform. IMHBs are shown in black dash lines and nonpolar hydrogen atoms are omitted for clarity.

We leveraged this workflow to analyze the conformational landscape of our most permeable structure, macrobicycle **1b**. We found that **1b** has multiple conformations in aqueous solution, but only one predominant conformation in chloroform (Figure 4B). To validate the conformer population of **1b** experimentally, we determined internuclear distance restraints for atom pairs in varying polarity solvents using NOE spectroscopy (see Figures S1–S3 for details). Few distance restraints were obtained in 9:1 MeOD:D_2_O, implying an open conformation and/or multiple conformations in a polar protic environment. However, many distance restraints were identified in 5:1 CDCl_3_:DMSO, and the resulting conformation determined from these distance boundaries aligned well with the MD predicted structure in chloroform (Figure 4B). Flexibility in aqueous environments and a rigidified ‘closed’ conformation in hydrophobic environments indicates that THBs are imparting chameleonic character to the permeable macrobicycles. Many highly permeable macrobicycles exhibited chameleonic conformational landscapes (see Figures S4 and S5). Further comparative analysis of the THB networks across different scaffolds (e.g., His-bridged **1b** vs Cys-bridged **1s**, Thr-bridged **3b**, and Ser-bridged **3h**) reveals that the bridging-His plays a central role (Figures 4C and S6). Unlike other residues, the imidazole side chain acts as a “hub” for the internal network, capable of serving as a hydrogen bond donor and acceptor simultaneously. In the chloroform ensemble of **1b**, the bridging- His engages in intramolecular hydrogen bonds (IMHBs) with up to four different residues, forming different hydrogen bonds as shown in Figure 4C, a connectivity that is notably absent in the less permeable analogs.

To elucidate the mechanistic basis of the observed permeability variations, we performed a systematic analysis of the THB networks and conformational ensembles across the synthesized library. We dissected the structure-permeability relationships by systematically evaluating modifications at positions R_1_ through R_5_.

1. The introduction of polar side chains, such as Ser (**1i**) or Asn (**1m**) at the R_5_ position, significantly reduced permeability. Structural analysis suggests that this stems from a geometric constraint of the ring right to the bridge, which contains the R_5_. The ring is smaller and lacks flexibility to sequester the polar alcohol or carboxamide side chains. Thus, these polar groups remain solvent-exposed. As shown in Figure 4D, both **1i** and **1m** have higher frequencies of intermolecular H-bonds and low frequencies of intramolecular H-bond. This increases the desolvation energy and decreases permeability.
2. At the R_4_ (bridging) and R_3_ positions, a highly cooperative effect was observed between the two residues. Generally, analogs with a d-His exhibited higher permeability than their L-counterparts. This correlates well with an increased frequency of the Hb1 interaction as shown in Figure 4E. Analogs **1b** and **1f** have opposite chiralities for R_3_ and R_4_, and they have much higher permeabilities than **1a** and **1g**, respectively, which have the same chirality. This seems to be correlated well with the fact that **1b** and **1f** have much higher Hb3, Hb4, and Hb5 contents than **1a** and **1g**, as shown in Figure 4F. Thus, **1b** and **1f** are in a more “closed” conformation.
3. Side-chain variation at R_3_ further underscores the network’s adaptability. With **1f** as reference, when R_3_ is Asp (**1k**), permeability is increased. But when R_3_ is Trp (**1l**), permeability is significantly reduced. The R_3_ (and R_2_ and R_1_, see below) are in a larger ring, which has conformational flexibility to allow hydrophilic side-chain groups to interact with the backbone. We observed that the introduction of the hydrophilic Asp facilitates more interactions between the Asp carboxylate and the bicyclic scaffold (Figure 4G). In contrast, the bulky Trp residue (**1l**) disrupts this connectivity (Figure S6).
4. The impact of a polar side chain at R_2_ is governed by the electronic profile of the group. While Ser substitution (**1j** vs **1i**) significantly enhances permeability, Asn substitution (**1n** vs **1m**) maintains the same permeability. Just like the case of **1k**, the hydroxyl group of Ser can participate hydrogen bond network to facilitate the “closed” conformation of the bicyclic backbone. The polar group of Asn (**1n**) can also form IMHBs with other residues on the scaffold. However, the side chain of Asn has two H-bond donors but only one acceptor. This excess of donors increases the probability of forming intermolecular hydrogen bonds with bulk water (Figure 4H), thereby raising the desolvation cost.
5. At the R_1_ position, the N-terminal acetamide (NHAc) group is typically solvent-exposed in the “open” conformations according to our MD simulations. It has little interaction with the bicyclic backbone. Therefore, its removal or modification generally enhances permeability by reducing the desolvation penalty, and it also contributes little to the stabilizing internal network.

A comparison of **1b** and **1p** reveals an anomaly. The difference is the replacement of the NHAc of R_1_ by H. According to (5), **1p** should have greater permeability than **1b**. But experimental results found that **1p** has a significantly reduced permeability. MD simulations revealed that **1p** is restricted to a single major “open” conformation in both aqueous and chloroform solvents (Figure 4I). Except for the solvent-exposed NHAc group, the remaining scaffold of the **1p** “open” conformation resembles the “open” conformation of **1b**. Thus, although the removal of NHAc reduces the interaction between the structure and water, it may also impart rigidity to the left ring of the macrocycle. We also identified three additional bicyclic peptides in the data set that, like **1p**, present a single conformation in both water and chloroform and exhibit poor permeability (**1q**, S6, and S7a, see Figure S7). For example, **1q** has the R_2_ removed, contracting the ring left to the bridge and reducing flexibility. It disrupts the THB network and maintains an “open” conformation in both the aqueous and CHCl_3_ solvents, as shown in Figure 4J.

Using our OFCP-based data set, we first attempted to predict permeability using conventional physicochemical descriptors, such as average three-dimensional polar surface area (3D_PSA), and average solvent accessible surface area (SASA) (see Table S1).^31^ While Lokey et al. found that SASA in cyclohexane correlated excellently with cell permeability for cyclic hexapeptides, we observed a poor correlation for our scaffolds using this approach.^17^ This discrepancy likely arises from differences in lipophilicity between the two data sets. Our data set contains significantly polar functionality, and SASA fails to capture the specific penalty of desolvating polar groups. The 3D_PSA derived from MD trajectories showed a marginally better correlation with the logarithm of PAMPA permeability (Figure 5A), reflecting the importance of hydrophilicity.

**Figure 5:**
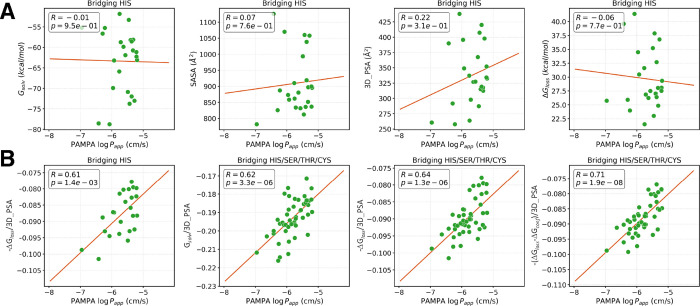
Exploration of predictive metrics for the prediction of permeable potential loop mimetics. (A) Scatter plots showing poor correlations between logarithm of PAMPA permeability (log*P*_app_) and standard physicochemical descriptors (*G*_solv_, SASA, 3D_PSA, and Δ*G*_loss_) for the bridging-His bicycles. (B) Improved correlations achieved by normalizing energetic penalties by 3D_PSA and correcting for conformational transition costs.

According to the classical solubility diffusion model of passive membrane permeability, the kinetic permeability (*P*_e_) is inversely related to the diffusional resistance across the membrane. For a rate-limiting barrier, typically the hydrophobic core where the molecule is fully desolvated, the relationship simplifies to

$$P_{e}=K_{p}D/d$$

where *P*_e_ is the permeabililty (cm/s), *K*_p_ is the unitless partition coefficient of the molecule in the membrane, *D* is the diffusion coefficient in the membrane (cm^2^/s), and *d* is the membrane thickness (cm).

If we assume that the membrane interior and surface are isotropic, then we can consider that the diffusion coefficient *D* is relatively constant for molecules of similar size. Therefore, the kinetic *P*_e_ is mainly determined by the thermodynamic partition coefficient. In other words, the kinetic flux across the membrane is directly governed by the thermodynamic free energy penalty required for the molecule to move from aqueous to the low-dielectric membrane.

Initially, the comparison between **1f** and **1p** suggested a link between free energy of water solvation and permeability. However, fitting this descriptor across the entire data set of bridging-His bicyclic peptides revealed a poor correlation (Figure 5A). Inspired by Jacobson et al., we also investigated the correlation with the solvation free energy loss upon transferring from water to chloroform (Δ*G*_loss_).^18^ This metric alone also showed poor correlation.

We hypothesized that the failure of these individual descriptors stems from their dependence on absolute molecular size. Consequently, we tested the ratio of Δ*G*_loss_ to 3D_PSA (Δ*G*_loss_/3D_PSA) against the logarithm of PAMPA permeability for the bridging-His bicycles. Surprisingly, this yielded a strong linear correlation, with a Pearson correlation coefficient of 0.61 (Figure 5B). Expanding this to include all bicyclic peptides in the library maintained this correlation. The solvation free energy difference quantifies the total energetic penalty of transferring the peptide from a polar to a nonpolar environment. However, Δ*G*_loss_ alone assumes a static penalty. By normalizing this penalty by the 3D_PSA, we derive a metric that represents the “desolvation cost efficiency” (DCE). DCE represents the average energy penalty paid per unit of polar surface area. A lower Δ*G*_loss_/3D_PSA ratio indicates that for every square angstrom of polar surface, the molecule pays a smaller energetic price to enter the membrane. This is consistent with a molecule’s ability to internally shield or reorganize its polar groups via hydrogen bonding and conformational adaptation.

We further expanded our analysis by systematically evaluating the linear correlations between experimental permeability and various physicochemical descriptors derived from MD simulations in both aqueous and chloroform. After excluding the monocyclic analogues from the bridging Cys series (see SI), the remaining bicycles exhibited robust linear correlations between the logarithm of PAMPA permeability and two specific energetic metrics: *G*_solv_/3D_PSA and Δ*G*_loss_/3D_PSA (see Figure S8). Both metrics demonstrated statistically significant correlations (*p* < 0.01). Interestingly, we observed that descriptors derived from aqueous simulations consistently outperformed those calculated from the chloroform. This predictive discrepancy likely stems from the limitations of using bulk chloroform as a membrane mimetic, while chloroform approximates the low dielectric constant of the membrane interior, it fails to recapitulate the specific organization and chemical heterogeneity, such as the presence of phosphatidylcholine headgroups, inherent to the lipid bilayers employed in PAMPA assays.

Building on the theoretical physical model of Jacobson et al., we postulated that the permeability of OFCP-based bicyclic peptides is governed not just by the static transfer energy, but by the free energy barrier of the transition.^32^ If a peptide adopts a single conformation in water (like **1p** and **1q**), it undergoes minimal conformational change upon entering the membrane. However, if this single conformation is polar (“open”), it incurs a high desolvation penalty and exhibits poor permeability. Conversely, if a peptide is chameleonic (like **1b**), it must undergo a conformational change to the “closed” state. The rate of passive membrane permeation is determined by the free energy barrier of the closed form from the bulk to the membrane phase.^32^ The permeability is thus a function of the solvation free energy difference between two phases minus the energetic cost of shifting the equilibrium from the open state to the closed state. Following this principle, we calculated the conformational free energy penalty (Δ*G*_conf_) for each peptide by clustering the aqueous ensembles and determining the population ratio of the “closed” state:

$$\Delta G_{\mathrm{conf}}=-RT\ln(P_{\mathrm{closed}}/P_{\mathrm{open}})$$

Where *R* is the ideal gas constant (8.314 J/(mol·K)), *T* is the absolute temperature (300 K). *P*_closed_ and *P*_open_ are the conformational population of “closed” and “open” macrostates, respectively.

When we added Δ*G*_conf_ as a correction term to our solvation free energy model, the correlation coefficient for the complete data set improved from 0.64 to 0.71 (Figure 5B). This improvement validates the physical model that permeability is determined by the sum of the desolvation penalty and the conformational work required to adopt the permeating pose.

## Conclusions

The presence of ‘chameleonic’ conformational ensembles is a determinant of passive membrane permeability for many cyclic peptides and peptidomimetics.^5^ Molecules showing this behavior can reduce the free energy barrier of membrane translocation by adopting conformations that match the polarity of their environment.^7–9^ Our experimentally validated MD workflow indicates that permeable OFCP-scaffolded macrobicycles are flexible and adopt internally hydrogen bonded conformers in nonpolar media. Crucially, the bridging imidazole acts as a dynamic hub in this process. It balances the need for aqueous solubility by remaining accessible in aqueous, yet readily pivots to form an extensive IMHB network that shields polar surface area during membrane insertion. This finding aligns with Baker’s data showing designed cyclic peptides become increasingly impermeable as the number of ‘unsatisfied’ backbone amide hydrogens increases.^12^

While chameleonic flexibility is advantageous, we need to fundamentally understand these permeability trends. In this framework of solubility-diffusion theory, permeation is governed by the sum of the static desolvation penalty (Δ*G*_loss_) and the conformational penalty (Δ*G*_conf_) required to reach a membrane-permeating “closed” state. A rigid molecule restricted to a polar “open” state (e.g., **1p**, **1q**) incurs a massive desolvation penalty and permeates poorly. Conversely, if a rigid scaffold were preorganized into a shielded “closed” state, it would minimize both Δ*G*_loss_ and Δ*G*_conf_, significantly enhancing permeability.

Perhaps most significant from our study is the finding that heterocycle mediated internal hydrogen bonding enables macrobicycles having polar side chains to retain permeability. Conventional metrics (cLogP, MW, tPSA, HBA, HBD, and NRotB)^31^ for these molecules would predict poor PAMPA performance. The anomalous behavior is explained by THB mediated ‘chameleonicity’, but we have also developed a more holistic parameter for predicting passive permeability of polar compounds generally. Quantifying the desolvation penalty upon moving from polar to nonpolar environments could fuel many additional studies. For example, we previously computationally evaluated a library of OFCP derived macrobicyclic backbones^20^ for overlap with the major peptide loop types that mediate domain–domain interactions in the protein data bank.^22^ At the level of amide backbone similarity, these studies identified potential mimics for nearly half of Kritzer’s ‘hot’ loop types, which represents thousands of individual protein–protein interactions. Using the linear relationship between desolvation penalty and permeability identified in this work, it should be possible to decorate those OFCP derived backbones with appropriate side chains and rank order the structures predicted to have the highest probability of traversing membranes passively (see Figure S9 for representative preliminary results). Studies along these lines are ongoing, with the goal of developing a systematic workflow to discover new ligands for all types of disease relevant, intracellular receptors.

## Supplementary Material



## Data Availability

The MD trajectory files, predicted top models for all macrobicycles, are available at 10.5281/zenodo.18242334.

## Notes

Experimental procedures, and HRMS, LC, and NMR characterization data for synthesized compounds; PAMPA procedure and associated data; and the details for the molecular dynamics simulations and associated analysis (PDF)
