# Supplementary material for: Bridging Heterocycle-Mediated Hydrogen Bonding Facilitates Permeability of Polar Macrobicycles
Source: J Am Chem Soc. 2026 Apr 15;148(16):17025–34. doi: 10.1021/jacs.6c01490 (PMC13134636; doi:10.1021/jacs.6c01490)
Supplement: Supplementary file 1 [file ja6c01490_si_001.pdf]

SUPPORTING INFORMATION

Bridging Heterocycle-Mediated Hydrogen Bonding Facilitates Permeability of Polar Macrobicycles

Gabriella I. D. Cooper[1,3], Botao Dai[2,3], Noah Durham[1], Gilbert L. Walker[1], Hongyi Yu[1], Angel Mendoza[1], Salvador J. Bernardino[1], Yun-Dong Wu[2]*, & Patrick G. Harran[1]*.

[1]Department of Chemistry and Biochemistry, University of California, Los Angeles, Los Angeles, California, 90095.

[2]School of Chemical Biology and Biotechnology, Peking University Shenzhen Graduate School, Shenzhen 518055, China (B. D. & Y-D. W.); Institute of Chemical Biology, Shenzhen Bay Laboratory, Shenzhen 518132, China (Y-D. W.); College of Chemistry and Molecular Engineering, Peking University, Beijing 100871, China (Y-D. W.).

[3]These authors contributed equally: Gabriella I. D. Cooper & Botao Dai.

**Corresponding author addresses: pharran@ucla.edu & wuyd@pkusz.edu.cn*

Supplementary Figures and Tables…………………………………………………………… **S3**

Figure S1. NOESY internuclear distances of **1b** determined in varying solvents.............. **S3**

Figure S2. NOESY vs. MD internuclear distances for **1b** in aqueous……………………… **S4**

Figure S3. NOESY vs. MD internuclear distances for **1b** in chloroform……………………. **S4**

Figure S4. Population of MD predicted top-ranked conformations in aqueous……………… **S5**

Figure S5. Population of MD predicted top-ranked conformations in chloroform…………… **S7**

Figure S6. MD predicted top conformations for some derivatives of **1** in chloroform……... **S9**

Figure S7. Poorly permeable polycycles have stabilized open conformations…………….... **S9**

Figure S8. Linear correlations between physicochemical descriptors and PAMPA……….. **S10**

Figure S9. Application in the prediction of potential permeable loop mimetics…………..... **S10**

Table S1. Calculated physicochemical descriptors for all bicyclic compounds…………..... **S11**

Synthetic Methods…………………………………………………………………………….. **S12**

General Information…………………………………………………………………… **S12**

General Procedures…………………………………………………………………….. **S12**

General Procedure A. *Solid-Phase Peptide Synthesis (SPPS)*………………….. **S12**

Figure S10. Representative synthesis of branched linear peptides…….. **S13**

Figure S11. Incorporation of 1,2,4-triazoyl analog of histidine………….. **S13**

General Procedure B. *Standard OFCP Processing*……………………………….. **S13**

General Procedure C. *Vinyl Fluoride Trapping with Exogenous Nucleophiles*…. **S14**

General Procedure D. *Free Acid Liberation*……………………………………… **S14**

General Procedure E. *Macrobicyclization via lactonization*………………………. **S14**

General Procedure F. *Macrobicyclization via lactamization*………………………. **S14**

Polycycle Synthesis…………………………………………………………………….. **S15**

*Polycycle **1** Analogs*…………………………………………………………………….. **S15**

*Polycycle **2** Analogs*…………………………………………………………………….. **S26**

*Polycycle **3** Analogs*…………………………………………………………………….. **S27**

*Polycycle **S1** Analogs*…… S33

*Polycycle **S2** Analogs*…… S34

*Polycycle **S3** Analogs*…… S37

*Polycycle **S4** Analogs*…… S39

*Polycycle **S5** Analogs*…… S40

*Polycycle **S6** Analogs*…… S41

*Polycycle **S7** Analogs*…… S42

Computational Methods…… S43

MD simulations…… S43

Clustering analysis…… S43

Calculation of conformational transition penalty…… S43

Hydrogen bonding analysis…… S44

Surface area analysis…… S44

Parallel artificial membrane permeability assay (PAMPA)…… S45

Table S2. Averaged and z-score filtered permeability data …… S45

Figure S12. Bar graph with averaged and z-score filtered permeability data …… S47

Table S3. Z-score filtered full PAMPA dataset…… S48

Table S4. Full PAMPA dataset and Z-score calculations…… S49

Solution-Phase Structural Determination…… S50

Polycycle 1b in DMSO-d_6…… S51

Table S5. *Chemical shifts and correlations for **1b** in DMSO-d_6*…… S51

Table S6. *Distance restraints for **1b** in DMSO-d_6*…… S52

Polycycle 1b in 5:1 $CDCl_3$:DMSO-d_6…… S55

Table S7. *Chemical shifts and correlations for **1b** in 5:1 $CDCl_3$:DMSO-d_6*…… S55

Table S8. *Distance restraints for **1b** in 5:1 $CDCl_3$:DMSO-d_6*…… S56

Polycycle 1b in 9:1 MeOD-d_3:D_2O…… S58

Table S9. *Chemical shifts and correlations for **1b** in 9:1 MeOD-d_3:D_2O*…… S58

Table S10. *Distance restraints for **1b** in 9:1 MeOD-d_3:D_2O*…… S59

Crystallographic Data…… S61

Table S11. Crystal data and structure refinement for **1a**…… S61

Table S12. Atomic coordinates and U_{eq} [Å^2] for **1a**…… S62

Table S13. Anisotropic displacement parameters [Å^2] for **1a**…… S64

Table S14. Bond lengths and angles for **1a**…… S65

Table S15. Torsion angles for **1a**…… S68

Spectroscopic Data…… S70

LC-HRMS Spectra…… S70

NMR Spectra…… S89

References…… S103

Supplementary Figures

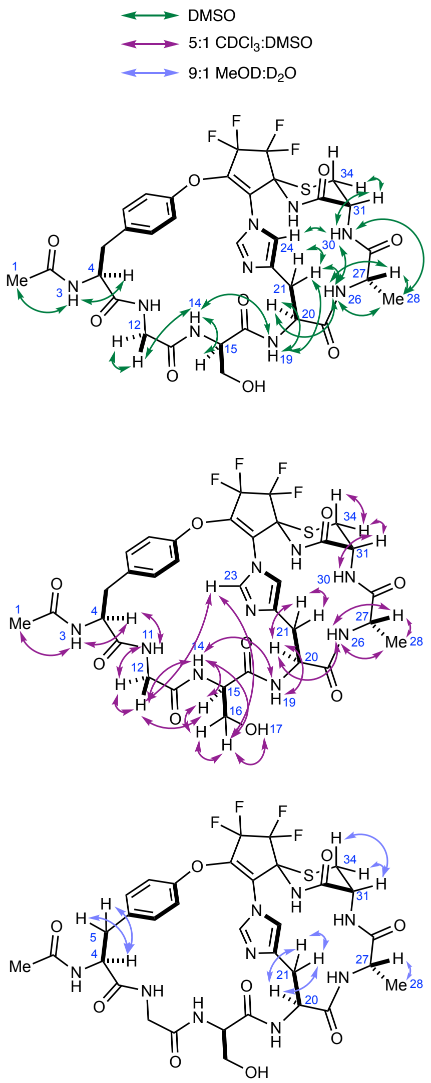

NOE Correlation	DMSO r_{unk} (Å)	5:1 $CDCl_3$:DMSO r_{unk} (Å)	9:1 MeOD:D_2O r_{unk} (Å)
1↔3	2.8	3.2	-
3↔4	3.3	2.9	-
4↔5	-	-	2.2
4↔5’	-	-	2.4
4↔11	-	2.6	-
11↔12	-	2.6	-
12↔12’	1.9	1.8	-
12↔14	3.3	-	-
12’↔14	-	2.7	-
12’↔23	-	3.3	-
12’↔15	-	3.1	-
14↔15	2.9	2.6	-
14↔16’	-	3.3	-
14↔19	3.1	2.9	-
15↔16	-	2.4	-
16↔16’	-	1.8	-
16’↔17	-	3.9	-
16’↔23	-	3.6	-
19↔20	2.6	2.7	-
20↔21	-	-	3.2
20↔21’	-	2.9	3.2
20↔26	-	2.9	-
21↔21’	1.8	1.7	1.8
21↔26	2.3	-	-
21↔19	3.0	-	-
24↔30	3.3	-	-
26↔20	2.8	-	-
26↔27	3.0	2.6	-
26↔28	2.6	3.2	-
26↔30	3.0	-	-
27↔28	2.6	2.5	2.4
30↔28	3.7	-	-
30↔31	2.7	2.9	-
31↔34	-	-	3.3
31↔34’	2.6	3.1	3.3
34↔34’	-	1.7	1.8

Figure S1. Comparison of distance restraints obtained by NOESY NMR in varying solvents for polycycle **1b**. Indicated geminal protons (blue) were used as references distances where r_{ref} = 1.8 Å. See page S50 for further experimental details.

Polycycle **1b**
MD predicted
conformations
in aqueous

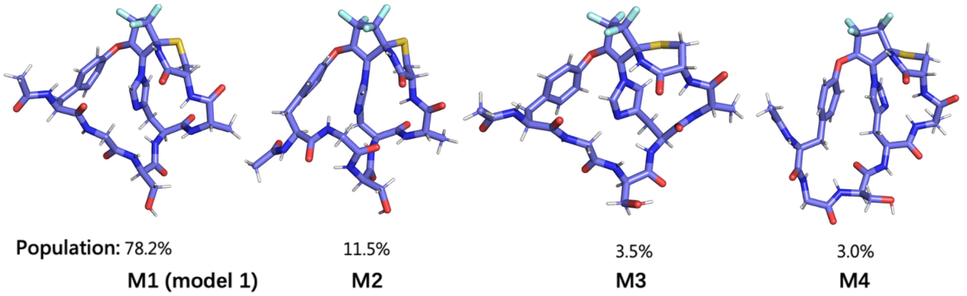

NOE Correlation	9:1 MeOD:D_2O r_{unk} (Å)	Predicted Distance (Å)			
		M1	M2	M3	M4
4↔5	2.2	2.3	2.8	2.2	3.0
4↔5’	2.4	2.4	3.0	2.7	2.4
20↔21	3.2	2.2	2.6	2.4	2.9
20↔21’	3.2	2.2	2.2	2.5	2.6
27↔28	2.4	2.4	2.5	2.1	2.5
31↔34	3.3	3.0	3.1	2.4	2.5
31↔34’	3.3	2.4	2.5	2.4	2.5

Figure S2. Experimental (NOESY) vs. simulated (MD) internuclear distances for **1b** in aqueous environment. Similar experimental and theoretical distances values are highlighted in green. See page S50 for further experimental details.

NOE Correlation	5:1 $CDCl_3$:DMSO r_{unk} (Å)	Predicted Distance (Å)	
		M1	Selected
1↔3	3.2	3.1	3.0
3↔4	2.9	2.9	2.9
4↔11	2.6	2.6	2.4
11↔12	2.6	2.5	2.7
12’↔14	2.7	2.8	2.8
12’↔23	3.3	3.4	4.4
**12’↔15**	**3.1**	**4.6**	**3.5**
14↔15	2.6	2.7	2.8
14↔16’	3.3	3.5	3.5
14↔19	2.9	3.0	3.0
15↔16	2.4	2.9	2.6
16’↔17	3.9	2.9	2.7
**16’↔23**	**3.6**	**7.0**	**3.8**
19↔20	2.7	2.8	3.1
20↔21’	2.9	2.7	2.8
20↔26	2.9	3.1	2.6
26↔27	2.6	2.5	2.1
26↔28	3.2	3.4	3.6
27↔28	2.5	2.5	2.5
30↔31	2.9	3.0	3.1
31↔34’	3.1	3.1	2.8

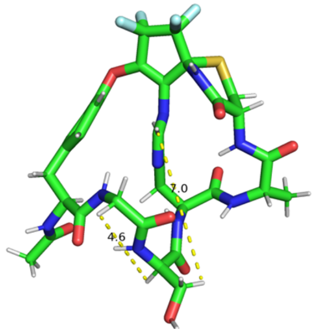

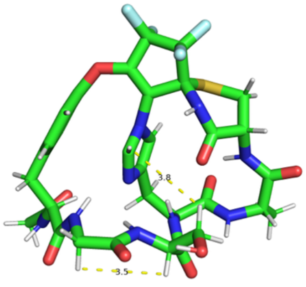

Figure S3. Experimental (NOESY) vs. simulated (MD) internuclear distances for **1b** in chloroform environment. The top and bottom models in the right panel are predicted top1 (M1) and selected conformations, respectively. For the MD selected model, we searched for the conformation with the closest internuclear distance to 12’-15 and 16’ -23 in the 5:1$CDCl_3$:DMSO from the entire trajectory. See page S50 for further experimental details.

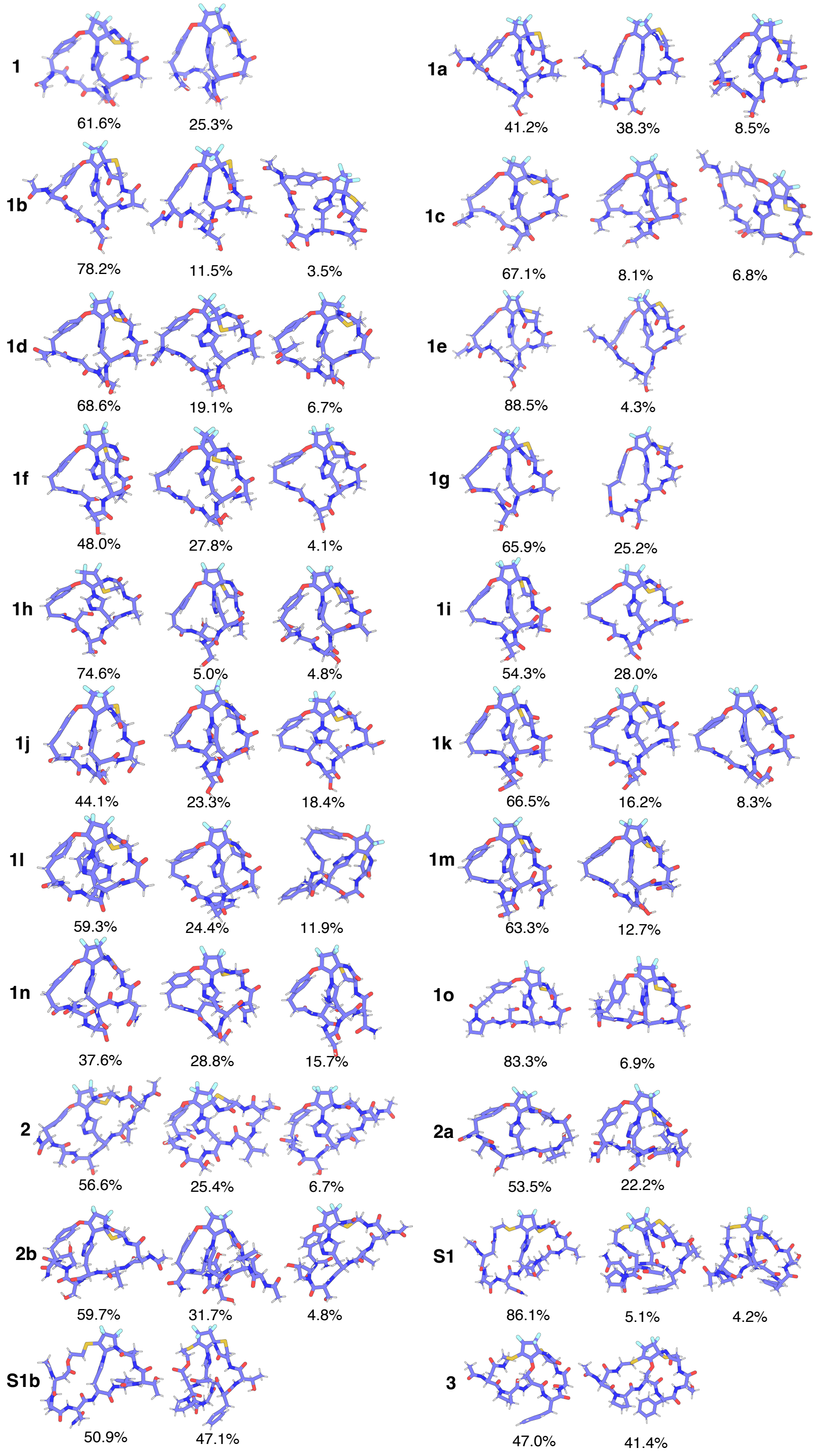

Figure S4. Population of MD predicted top-ranked conformations for different macrobicycles in aqueous. See page S43 for details of clustering methods.

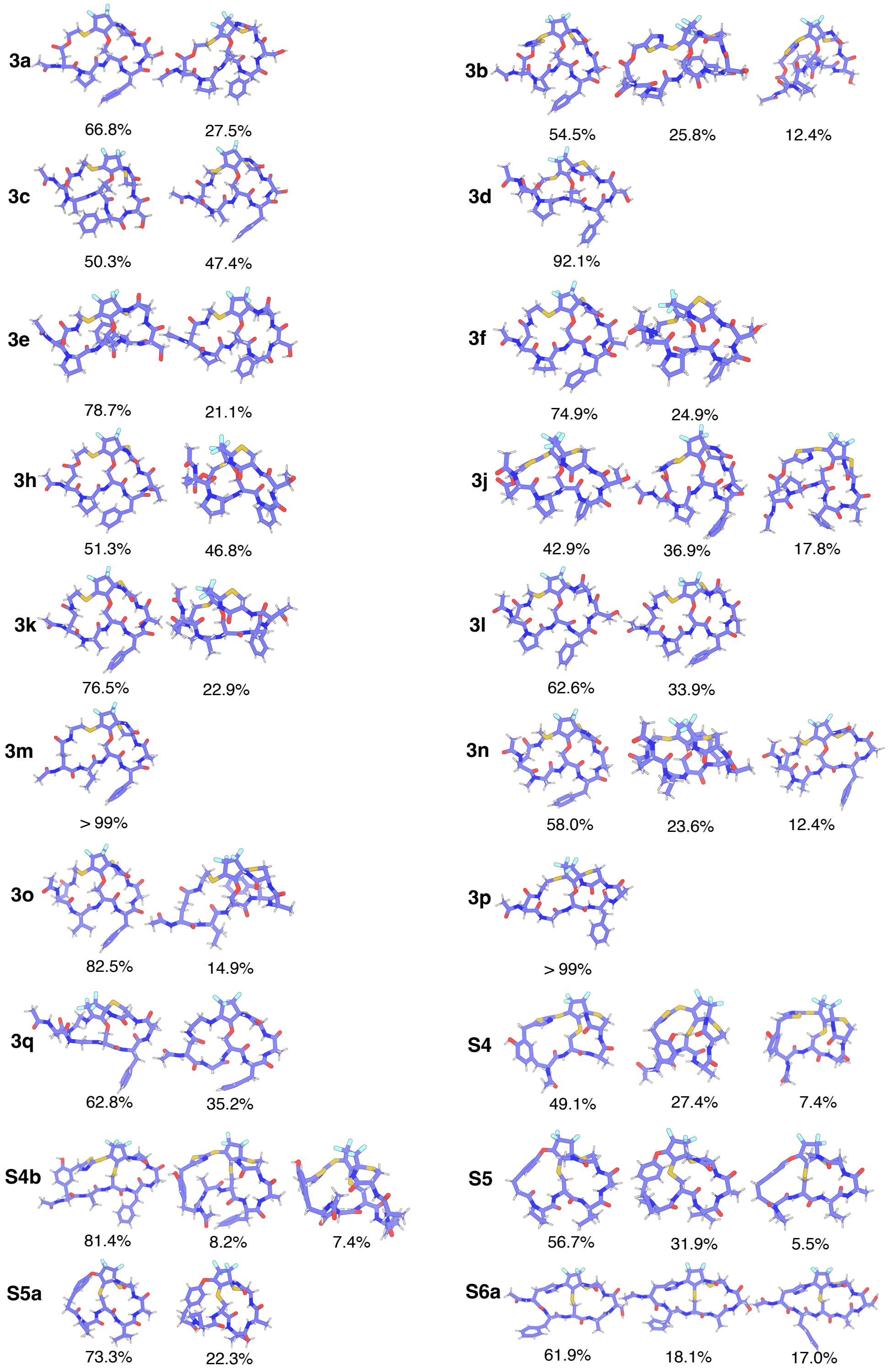

Figure S4 cont. Population of MD predicted top-ranked conformations for different macrobicycles in aqueous. See page S43 for details of clustering methods.

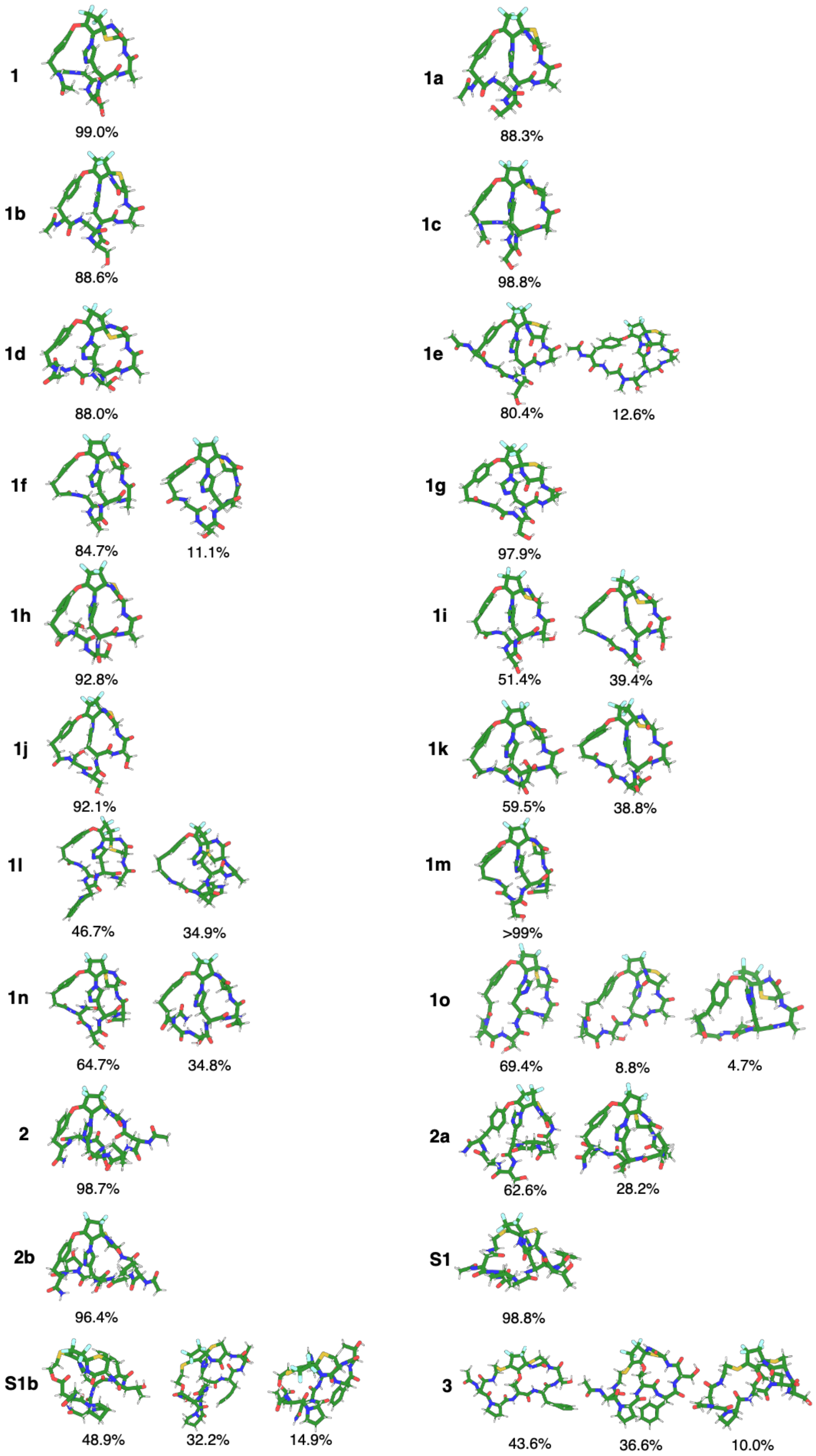

Figure S5. Population of MD predicted top-ranked conformations for different macrobicycles in chloroform. See page S43 for details of clustering methods.

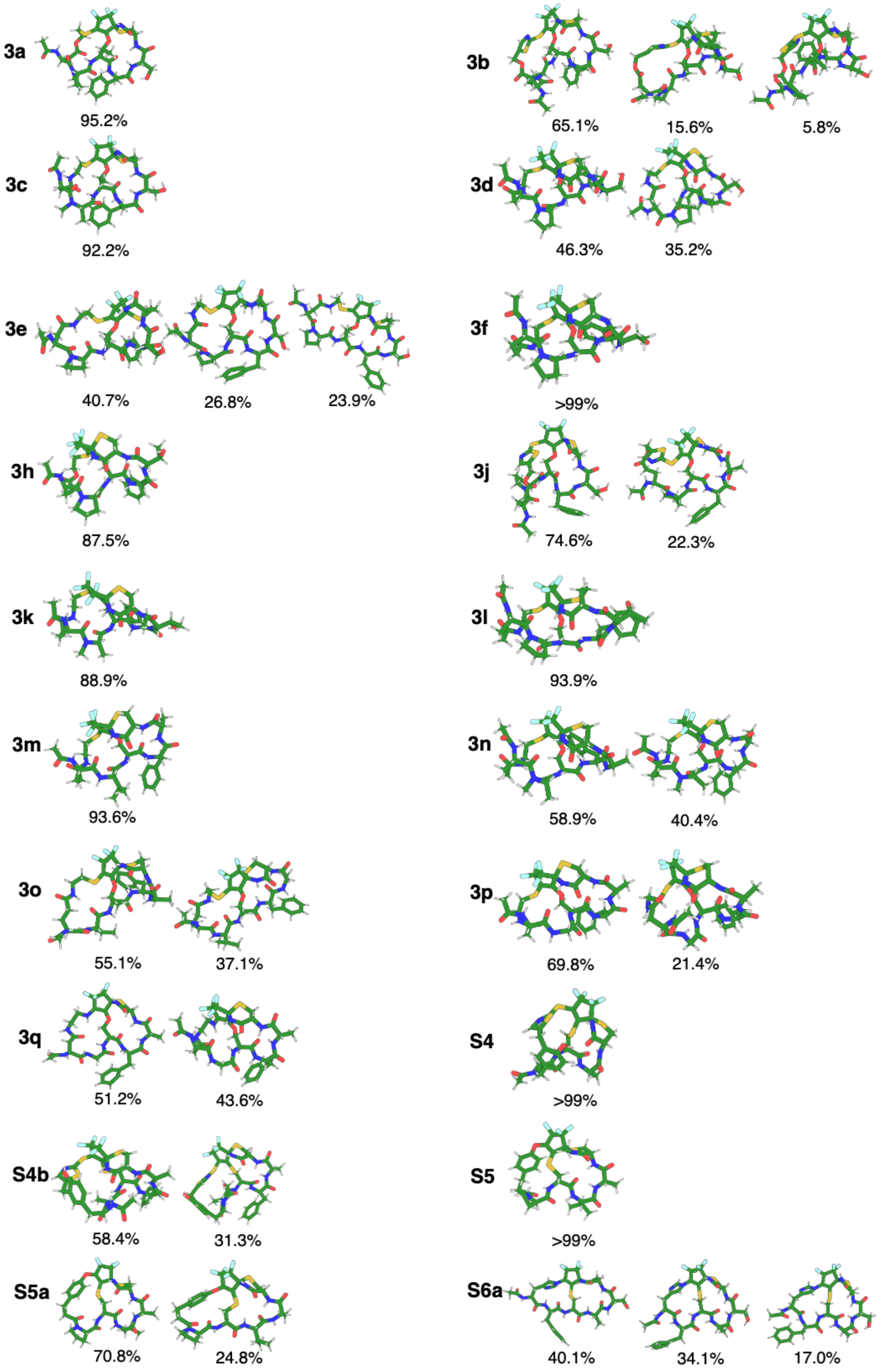

Figure S5 cont. Population of MD predicted top-ranked conformations for different macrobicycles in chloroform. See page S43 for details of clustering methods.

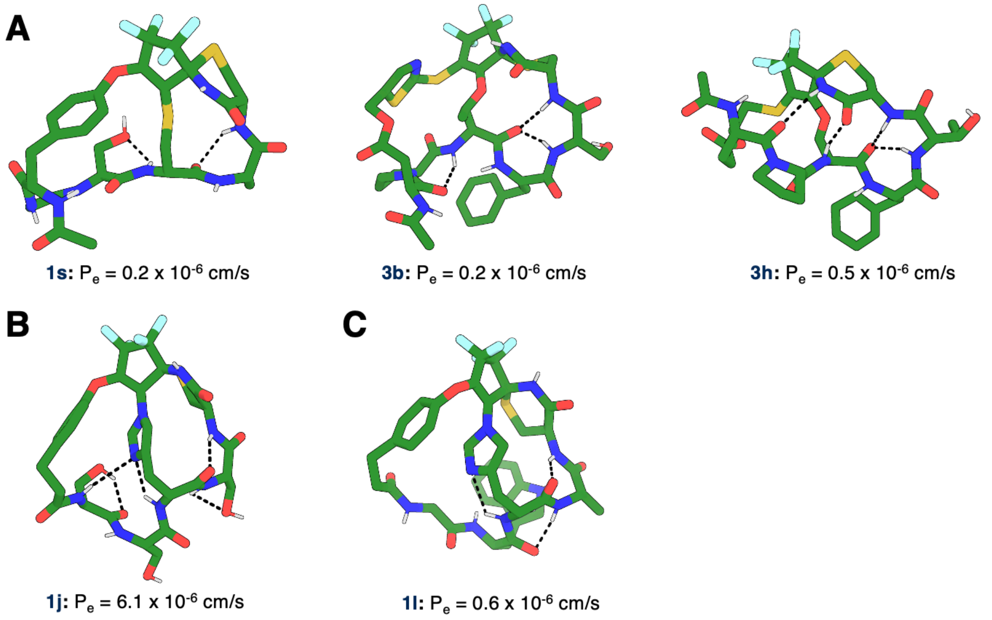

Figure S6. MD predicted top conformations for some derivatives of **1** in chloroform. **A**. The internal H-bonds between different bridging residues and others on the scaffold. **B**. The extensive THB netwok of the “closed” conformation of **1j**. **C**. The disrupted H-bonding network of **1l**. H-bonds are shown in black dash lines.

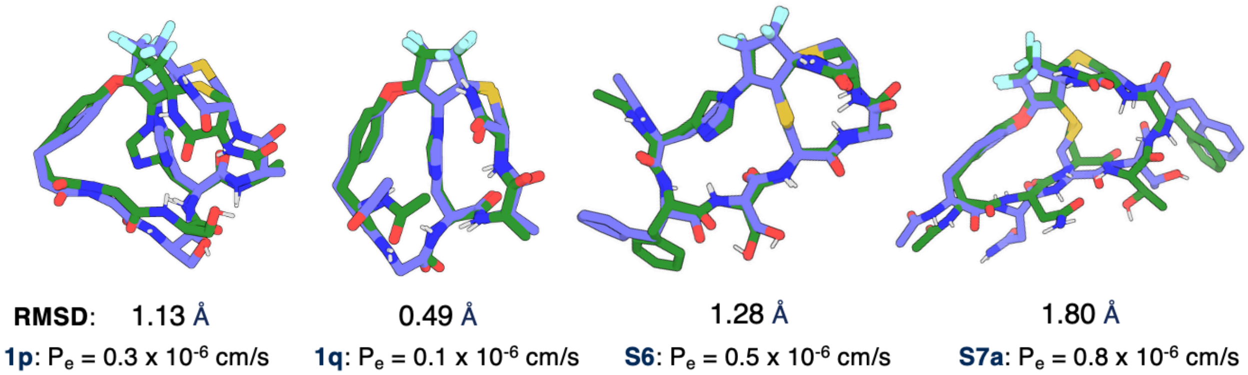

Figure S7. Poorly permeable polycycles have stabilized open conformations. Analogs **1p**, **1q**, **S6**, and **S7a** all exhibit poor permeability and are locked in a single “open” conformation in both aqueous and chloroform. All-heavy-atom RMSDs between the top models in aqueous and chloroform and PAMPA values are listed below each structure.

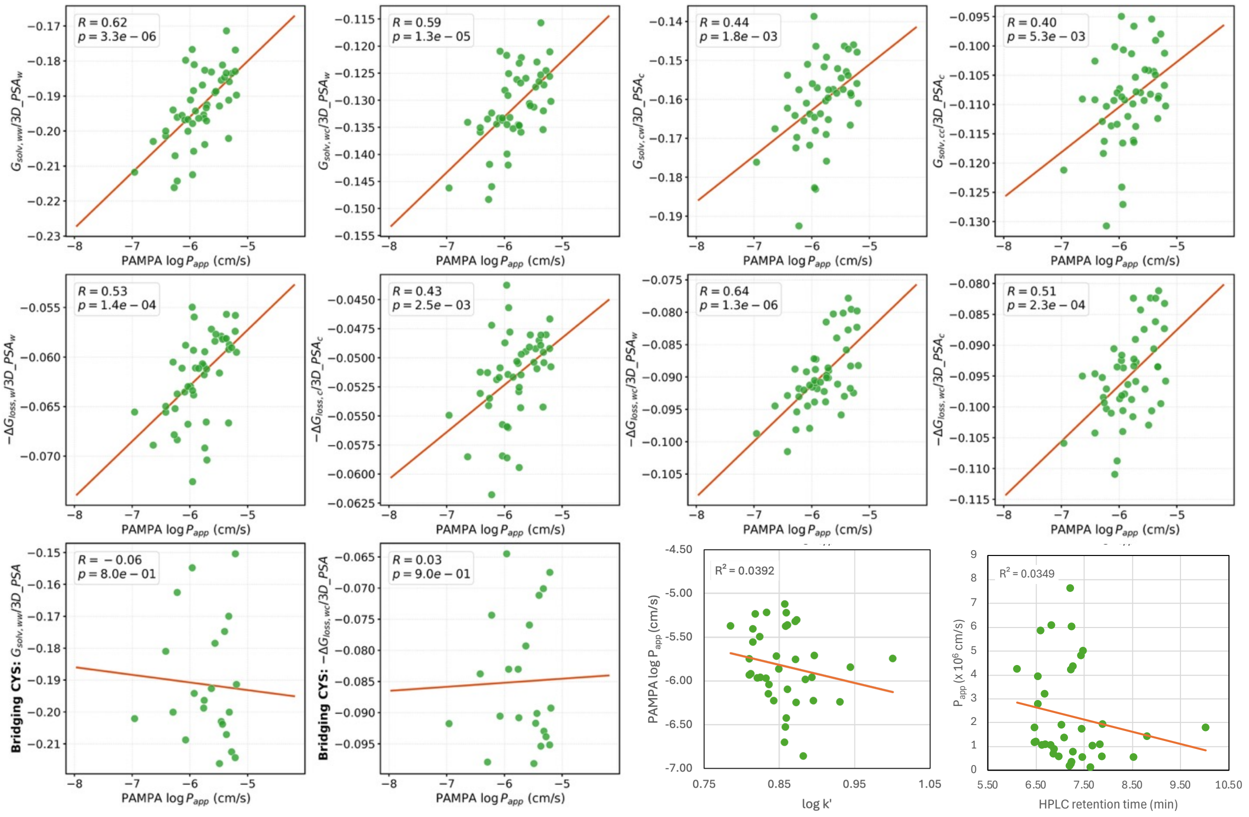

Figure S8. Linear correlations between tested physicochemical descriptors and the experimental PAMPA permeability. The notation "w/c" denotes the solvent environments, where the first letter represents the explicit solvent used for MD simulation and the second represents the implicit solvent model used for MM/PBSA calculations ("w" = water, "c" = chloroform). All computational values represent averages over the entire trajectory. Log k' represents the capacity factor derived from HPLC retention time.

A

Scaffold	Linear Sequence	Loop Type (PDB Code)	Predicted P_e ($\times 10^6$ cm/s)
H1H5C	Ac-H(A)$_1$H(A)$_5$C-NH$_2$	Asx-Motif (3B8I)	**0.9**
H5H2C	Ac-H(A)$_5$H(A)$_2$C-NH$_2$	γ-Turn (2WAM)	<0.1
C2H3C	Ac-C(A)$_2$H(A)$_3$C-NH$_2$	Asx-Turn (4HN1)	**1.2**
H4H4C	Ac-H(A)$_4$H(A)$_4$C-NH$_2$	S/T-Turn (4J07)	<0.1

B

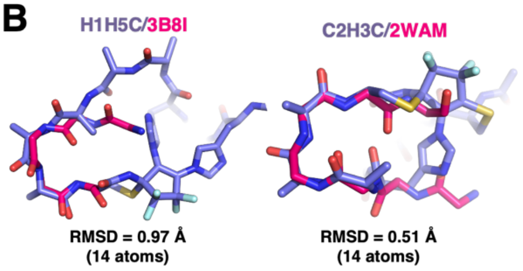

Figure S9. Exploration of predictive metrics and application in the prediction of potential permeable loop mimetics. **A.** PAMPA prediction of hypothetical "hot loop" mimetics. The table lists polyalanine-based bicyclic scaffolds designed to mimic Kritzer's protein loop structural motifs. Hypothetical scaffolds were defined as Ac-X–(Ala)$_m$–Y–(Ala)$_n$–Cys-NH$_2$ sequences and abbreviated as XmYnC: where X = Cys, His, and Tyr, Y = Cys and His and m, n = 1–5. Permeability is predicted using the linear model that combines ΔG_{loss} and ΔG_{conf}. Scaffolds with predicted high permeability are highlighted in green. **B.** Superposition of MD-predicted top conformations of H1H5C and C2H3C (purple sticks) with their respective target PDB loop structures (red sticks, PDB: 3B8I and 2WAM). Low RMSD values (calculated on the 14 aligned backbone atoms) indicate high structural similarity to the native biological loops.

Table S1. Calculated physicochemical descriptors for different bicyclic compounds.

Compound[a] No.	$G_{solv,ww}$[b] (kcal/mol)	$G_{solv,wc}$[b] (kcal/mol)	$G_{solv,cw}$[b] (kcal/mol)	$G_{solv,cc}$[b] (kcal/mol)	$clus_{open}$[c] (%)	$clus_{close}$[c] (%)	ΔG_{conf} (kcal/mol)	$3D\_PSA_w$[d] (Å²)	$3D\_PSA_c$[d] (Å²)	$SASA_w$[d] (Å²)
1	-59.86	-41.17	-49.02	-33.62	61.6	25.3	0.53	326.9	311.4	880.4
1a	-65.87	-45.35	-49.95	-34.23	41.2	8.5	0.94	337.2	311.5	910.3
1b	-63.08	-43.29	-49.29	-33.75	78.2	11.5	1.14	332.5	306.1	895.5
1c	-61.14	-34.77	-49.13	-33.63	67.1	8.1	1.26	334.2	315.1	899.6
1d	-58.11	-39.75	-48.69	-33.25	68.6	68.6	0	312.6	306.3	897.1
1e	-58.55	-41.96	-47.14	-32.15	88.5	88.5	0	319.2	306.4	919.0
1f	-53.28	-36.65	-44.02	-30.26	48.0	48.0	0	287.3	279.6	812.5
1g	-58.73	-40.0	-46.29	-31.87	65.9	65.9	0	298.0	280.2	833.9
1h	-58.19	-39.87	-45.63	-31.15	74.6	5.0	1.61	315.1	298.5	835.9
1i	-61.89	-42.77	-49.83	-34.39	54.3	54.3	0	327.5	314.6	831.6
1j	-62.26	-42.62	-49.92	-34.17	44.1	23.3	0.38	352.1	337.6	836.7
1k	-60.75	-41.93	-47.55	-32.69	66.5	16.2	0.84	317.8	300.1	851.9
1l	-55.24	-37.62	-46.03	-31.26	59.3	11.9	0.96	257.8	239.1	887.3
1m	-63.21	-43.67	-47.6	-32.74	63.3	12.7	0.96	349.2	325.3	858.3
1n	-69.92	-48.17	-51.74	-35.42	37.6	28.8	0.16	395.8	373.1	873.3
1o	-51.8	-35.5	-44.11	-30.3	5.5	69.4	-1.51[e]	263.8	261.0	861.9
1p	-56.62	-38.96	-44.66	-30.71	88.8	88.8	0	291.9	272.1	825.7
1q	-55.18	-38.1	-42.8	-29.45	97.1	97.1	0	260.6	243.0	781.8
2	-73.77	-50.07	-55.07	-35.9	56.6	1.4	2.20	407.3	376.4	1060.1
2a	-70.87	-48.23	-52.88	-35.64	53.5	13.0	0.84	367.5	342.2	993.4
2b	-71.99	-48.6	-58.37	-39.3	59.7	31.7	0.38	420.1	396.9	1038.5
S1	-78.77	-53.0	-56.35	-37.37	86.1	1.1	2.60	438.2	373.2	1070.0
S1b	-78.56	-52.99	-58.43	-38.97	50.9	1.4	2.14	413.2	379.9	1125.9
3	-62.51	-40.99	-51.33	-33.87	47.0	16.7	0.62	323.3	321.6	
3a	-63.69	-43.0	-51.14	-34.72	66.8	27.5	0.53	322.6	317.7	
3b	-66.38	-44.82	52.84	-35.56	54.5	12.4	0.88	331.9	325.7	
3c	-64.24	-43.67	-52.2	-34.43	50.3	50.3	0	326.6	318.8	
3d	-57.47	-38.76	-45.12	-29.23	92.1	27.2	0.73	314.7	302.5	
3e	-63.97	-43.48	-52.57	-34.71	78.7	78.7	0	323.3	319.4	
3f	-59.47	-40.16	-45.06	-30.11	74.9	24.9	0.66	318.3	297.2	
3h	-61.35	-41.41	-46.59	-32.63	51.3	46.8	0.05	312.9	295.8	
3j	-61.31	-38.89	-48.15	-32.96	42.9	17.8	0.52	318.5	305.7	
3k	-62.03	-42.52	-47.02	-32.71	76.5	22.9	0.72	319.3	299.5	
3l	-59.26	-41.14	-44.8	-30.65	62.6	0.2	3.42	314.1	294.6	
3m	-57.25	-38.39	-43.36	-29.82	5.4	93.6	-1.70[e]	299.6	278.0	
3n	-59.75	-40.94	-45.51	-31.58	58.0	23.6	0.54	317.1	270.9	
3o	-61.37	-40.54	-50.4	-32.8	82.5	14.9	1.02	302.4	300.8	
3p	-63.56	-43.69	-49.01	-33.65	18.0	69.8	-0.81[e]	325.1	296.1	
3q	-65.77	-45.37	-52.89	-36.71	62.8	35.2	0.35	319.6	288.9	
S2	-49.95	-34.44	-42.27	-29.39				259.3	245.8	
S2a	-50.46	-34.83	-42.39	-29.38				253.9	239.7	
S2b	-49.46	-32.12	-40.56	-26.39				258.5	244.5	
S2c	-52.84	-35.76	-41.74	-28.29				264.1	249.0	
S2d	-54.21	-37.09	-43.39	-29.72				267.1	251.9	
S2e	-53.54	-36.65	-46.19	-31.74				306.4	268.7	
S2f	-54.70	-36.56	-46.87	-31.43				306.5	293.9	
S2g	-64.78	-44.16	-52.44	-35.71				430.7	399.6	
S3	-40.81	-28.73	-33.89	-23.98				240.1	223.1	
S3a	-39.15	-25.79	-32.28	-21.24				240.9	226.1	
S3b	-45.37	-31.22	-37.56	-25.97				233.7	219.0	
S3c	-42.25	-29.42	-35.46	-24.64				273.0	234.2	
S4	-49.05	-33.78	-38.53	-26.37	49.1	27.4	0.35	249.8	235.4	
S4b	-59.19	-39.43	-45.78	-30.21	81.4	7.4	1.43	295.9	266.5	
S5	-42.96	-28.75	-34.72	-23.42	56.7	56.7	0	212.3	208.4	
S5a	-43.54	-28.67	-35.79	-24.31	73.3	73.3	0	204.9	195.9	
S6	-67.87	-46.57	-53.99	-37.05	25.3	43.0	-0.32[e]	314.0	313.1	
S6a	-66.4	-45.49	-52.28	-35.81	61.9	18.1	0.73	320.7	308.0	
S7	-44.67	-29.60	-38.06	-25.29				214.0	222.6	
S7a	-66.41	-45.07	-52.54	-35.67	99.0	99.0	0	367.0	352.9	
S7b	-51.17	-34.77	-41.63	-28.44				238.8	247.4	
H1H5C	-79.22			-40.34	93.4	93.4	0	420.9		
H5H2C	-84.30			-41.41	32.1	<0.1		441.2		
C2H3C	-68.37			-33.93	48.7	26.3	0.37	381.1		
H4H4C	-91.15			-42.85	82.1	<0.1		467.4		

[a]The compounds used for linear fitting are colored in green.

[b]The notation "w/c" denotes the solvent environments, where the first letter represents the explicit solvent used for MD simulation and the second represents the implicit solvent model used for MM/PBSA calculations ("w" = water, "c" = chloroform).

[c]The population of the open state and closed state in aqueous, see SI for more details of the clustering methods.

[d]The calculated average values for different compounds for the whole trajectory in aqueous.

[e]For these compounds, the closed conformation was not observed in the aqueous-phase MD trajectory. Consequently, the population of the open conformation was estimated based on backbone RMSD < 1.5 Å in the chloroform-phase trajectory.

Synthetic Methods

General Information

Reagents were purchased from commercial vendors and used as received unless otherwise stated. Tetrahydrofuran (THF), diethyl ether (Et_2O), acetonitrile (MeCN) and toluene (PhMe) were passed through a Glass Contour solvent drying system. Anhydrous N,N-dimethylformamide (DMF) was purchased from Sigma Aldrich. All reactions were run under an argon atmosphere using anhydrous solvents unless otherwise stated. Yields refer to chromatographically (Agilent 6545 LC-QTOF) homogeneous materials, unless otherwise stated. Thin-layer chromatography (TLC) was conducted on precoated plates (Sorbent Technologies, silica gel 60 PF254, 0.25 mm) visualized with UV 254 nm. Column chromatography was performed on silica gel 60 (SiliCycle, 240−400 mesh). Purification of peptides was performed using an Agilent 1200 HPLC system equipped with G1361A preparative pumps, a G1314A auto sampler, a G1314A VWD, a G1364B automated fraction collector, and a Waters Sunfire C18 column (5 μm, 19 mm × 250 mm), using 0.1% TFA in MeCN/H_2O solvent system unless otherwise noted. Analytical HPLC was performed using the same system, but with a G1312A binary pump. High-resolution mass spectra were recorded on Agilent 6545 LC-QTOF and Thermo Scientific Exactive® Mass Spectrometer with DART ID-CUBE Waters GST Premier. NMR spectra were recorded on Bruker Advance (300, 400, 500, or 600 MHz) spectrometers. HSQC, HMBC, and NOESY NMR experiments were used to aid assignment of NMR peaks when required. All ^{19}F NMR spectrums used $CFCl_3$ as a calibration standard.

Synthesis of all compounds described was performed in accordance with the general protocols outlined in our previous publications.[1, 2] The synthesis and characterization of compounds **1**, **1a**, **1q**, **2**, **3**, **3b**, **3f-i**, **3m-p**, **3r**, **S1**, **S1a-c**, **S2**, **S2a**, **S2f-g**, **S3**, **S4**, **S4a-c**, **S5**, **S6**, **S6a**, **S7**, and **S7a-b** were previously reported.[1, 2] Mass limited samples were characterized for identity and purity by the HRMS and LC chromatogram produced by Agilent QTOF 6545.

General Procedures

General Procedure A. Solid-Phase Peptide Synthesis (SPPS)

Primary C-terminal carboxamide peptides were synthesized manually using standard Fmoc-SPPS protocols (unless otherwise specified) on Fmoc-Rink Amide MBHA resin (Chem-Impex, 200-400 mesh, 0.73 mmol/g, 1% DVB, 1.0 eq) using a fritted glass reaction vessel and a Burrell Wrist Action Shaker. Resin was swollen by covering with DMF and shaking for 20 mins, then drained prior to first deprotection.

i. **Fmoc-deprotection**: Covered resin with 2%/5% DBU/piperazine in DMF (shake 1 x 15 min). The reaction vessel was then washed with DMF (2x), DCM (2x), and DMF (2x).
ii. **Coupling**: Added the appropriate Fmoc-amino acid (3.0 equiv.) and HBTU (3.0 equiv.) followed by DMF (to cover resin) and DIPEA (10.0 equiv.) to the reaction vessel. The resin was shaken for 45 minutes, drained, and washed with DMF (2x), DCM (2x), and DMF (2x).
iii. **N-Terminus capping**: After the final Fmoc-deprotection, the resin was covered with DMF and treated with DIPEA (5.5 eq) and Ac_2O (5.0 eq). The resin was shaken for 20 minutes, drained, and washed with DMF (2x), DCM (2x), and DMF (2x).
iv. **Cleavage**: Resin was shaken with a solution of 18:1:1 TFA/H_2O/TIPS (to cover resin) for 90 minutes. The cleaved resin was filtered and rinsed with Et_2O until ~200 mL of volume. The peptide was then triturated and centrifuged, and this process repeated with Et_2O (2x) to afford a crude peptide pellet. This peptide pellet was then stripped with anhydrous PhMe (3x) to afford the desired linear peptide. Linear peptides of ≥80% purity (HPLC analysis) were carried forward

without further purification. When necessary, analytically pure linear peptides were obtained by preparative HPLC (0.1% TFA in MeCN/H_2O).

Secondary C-terminal carboxamide peptides were synthesized analogously to the above procedure but utilizing 2-chlorotrityl chloride resin (Chem-Impex, 100–200 mesh, 1.02 mmol/g, 1% DVB). After all couplings were completed, the resin was cleaved by shaking with a solution of 1:99 TFA:CH_2Cl_2 (3 x 33 mL, total of 100 mL). Filtered onto a solution filled with 1:9 pyridine:MeOH (100 mL). Concentration under reduced pressure afforded desired crude C-terminal carboxylate peptide. This crude peptide was then capped with $MeNH_2$. C-terminus capping was achieved following general EDC•HCl/HOBt coupling protocol. After capping, the crude peptide was purified via preparative HPLC.

Branched linear peptides were synthesized on Fmoc-Rink Amide MBHA resin and elongated to the desired branching site using **General Procedure A** described above. Then, either **D*** or **E*** (see **Fig. S10**) was coupled using the standard coupling procedure. The sequenced was elongated to the desired length and the N-terminus was capped, with the sidechain acid CSY protection intact. Then, the acid was liberated oxidatively with NCS, as described by Bode *et al.*[3] The resin was covered with 90:8:2 DMF/H_2O/HFIP and NCS (0.5 equiv.) was added and shaken for 2 minutes then drained. This process was repeated 3x for a total of 1.5 equiv. NCS. The resin was washed with DMF (2x), DCM (2x), and DMF (2x). Then, the liberated free acid was coupled with H-Cys(Trt)-NH_2 using the standard coupling procedure, then the branched peptide was cleaved from the resin as described above.

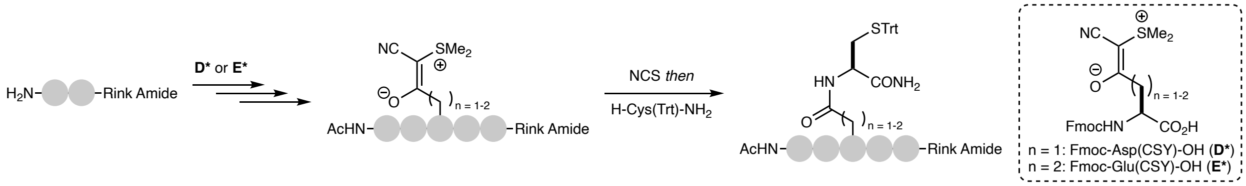

Figure S10. Representative synthesis of branched linear peptides. **D*** and **E*** were synthesized in accordance with the published procedure.[3] H-Cys(Trt)-NH2 was obtained commercially (Aapptec).

Linear peptides with synthetic 1,2,4-triazoyl analog of histidine (**H*** and **h***) were synthesized on Fmoc-Rink Amide MBHA resin and elongated to the desired residue using **General Procedure A** described above. **H*** and **h*** were coupled to the free N-terminus using standard conditions as Trt-Trz(Trt)-OH (See **Fig. S11**). The N_α-trityl was selectively deprotected by covering the resin with 95.5:3:1.5 DCE/MeOH/TFA and shaking for 5 mins. The resin was filtered quickly and washed with DMF (2x), DCM (2x), and DMF (2x). The subsequent couplings and completion of the linear peptide were preformed using the standard procedures described above.

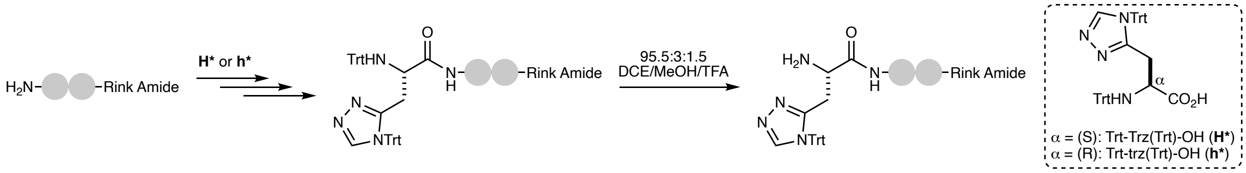

Figure S11. Representative scheme depicting incorporation of 1,2,4-triazoyl analog of histidine. See page S24 for preparation of **H*** and **h***.

General Procedure B. Standard OFCP Processing (Direct Macrobicyclization)

To a flame-dried round bottom flask equipped with a stir bar was charged with linear peptide (1.0 equiv), diluted with anhydrous DMF (5.0 mM), and then allowed to stir at 0 °C. Then OFCP (1.5 equiv, 1.0 M in MeCN) was added followed by NEt_3 (2.5 equiv.). The reaction mixture was allowed to stir at 0 °C for 30 min.

When HPLC indicated macrocycle intermediate formation, the reaction mixture was concentrated under reduced pressure to remove volatiles (excess OFCP). The reaction residue was then diluted with DMF (5.0 mM) and stirred at 0 °C. Then Cs_2CO_3 (4.0 equiv.) was added, and the reaction mixture allowed to gradually warm up to 23 °C for 3 h. When HPLC indicated reaction completion, the reaction mixture was quenched with AcOH (10.0 equiv.) and then concentrated under reduced pressure to afford crude product. The crude product was then purified via preparative HPLC or by silica gel flash column chromatography (FCC) as specified below.

General Procedure C. Vinyl Fluoride Trapping with Exogenous Nucleophiles

To a flame-dried round bottom flask equipped with a stir bar was charged with linear peptide (1.0 equiv), diluted with anhydrous DMF (10.0 mM), and then allowed to stir at 0 °C. Then OFCP (1.5 equiv, 1.0 M in MeCN) was added followed by NEt_3 (2.5 equiv.). The reaction mixture was allowed to stir at 0 °C for 30 min. When HPLC indicated macrocycle intermediate formation, the reaction mixture was concentrated under reduced pressure to dryness. The reaction residue was then added 1:4 DMF:THF (30 mM) and cooled to 0 °C. Then Cs_2CO_3 (5.0 equiv.) was added, and the reaction mixture allowed to stir at 0 °C for 1 h. When HPLC indicated full conversion to the desired vinyl fluoride then the indicated exogenous nucleophile (1.2 eq) was added and the reaction mixture allowed to stir at 0 °C for 1 h. When HPLC indicated reaction completion, the reaction mixture was quenched with AcOH (10.0 equiv.) and then concentrated under reduced pressure to afford crude product. The crude product was then telescoped to the next step.

General Procedure D. Free Acid Liberation

Assumed a 50% yield from **General Procedure C** when calculating stoichiometry. To the same round bottom flask used for **General Procedure C**, the crude product was diluted with a solution 2:8 MeCN:aqueous buffer pH 4.5 (NaOAc/AcOH, 0.1 M). The reaction mixture was then allowed to stir at 23 °C and then was added NCS in MeCN (1.0 M) portion wise (0.5 equiv.). Total NCS varied from 1.5–3.0 equiv. depending on substrate. When HPLC indicated full conversion to the desired free acid, the reaction mixture was quenched with $Na_2S_2O_3{\bullet}5H_2O$ (6.0 equiv.). The reaction mixture was concentrated under reduced pressure to afford crude product. The crude product was then purified via preparative HPLC. Yields shown refer to yields from their linear peptides.

General Procedure E. Macrobicyclization via lactonization

To a flame-dried round bottom flask equipped with a stir bar was charged with the incipient seco acid (1.0 equiv.), EDC•HCl (2.5 equiv.), HOBt (1.5 equiv.), and then diluted with DMF (5.0 mM). The reaction mixture was then allowed to stir at 0 °C and then added NEt_3 (5.0 equiv.). The reaction mixture was then allowed to warm up to 23 °C and stirred for 44–64 h. After HPLC indicated reaction completion, the reaction mixture was quenched with AcOH (5.0 equiv.) and then concentrated under reduced pressure to afford crude product. The crude product was then purified via preparative HPLC.

General Procedure F. Macrobicyclization via lactamization

To a flame-dried round bottom flask equipped with a stir bar was charged with the incipient amino acid (1.0 equiv.) and diluted with anhydrous DMF (5.0 mM). The solution was added $iPrNEt_2$ (3.5 equiv.) and the reaction mixture allowed to cool to 0 °C. Then propanephosphonic acid anhydride in EtOAc (1.5 equiv., 50% wt.) was added and the reaction mixture allowed to stir at 0 °C for 1 h. After HPLC indicated reaction completion, the reaction mixture was quenched with AcOH (5 equiv.) and then concentrated under reduced pressure to afford crude product. The crude product was then purified via preparative HPLC.

Polycycle Synthesis

Linear precursors are written following standard convention with N→C-terminus. Upper-case letters refer to L-amino acids, and lower-case letters refer to D-amino acids. Abbreviations: Dat = Des-amino tyrosine.

Polycycle 1 Analogs

Compound	Linear Precursor	Structure
**1**<br>(Compound 13, *J. Am. Chem. Soc.*, **2023**, *145* (29), 15888-15895.)	Ac-YGShAC-NH_2	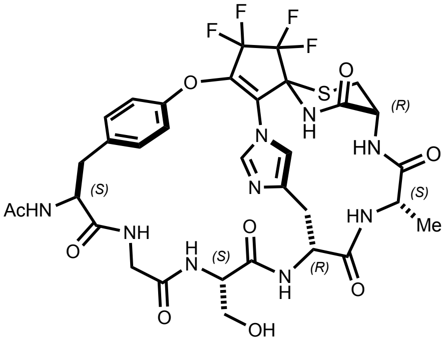
**1a**<br>(Compound 4, *J. Am. Chem. Soc.*, **2023**, *145* (29), 15888-15895.)	Ac-YGSHAC-NH_2	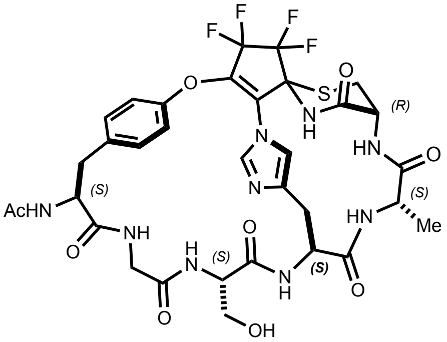
**1b**	Ac-YGsHAC-NH_2	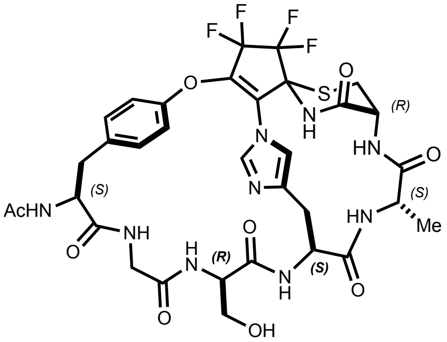
**1c**	Ac-YGshAC-NH_2	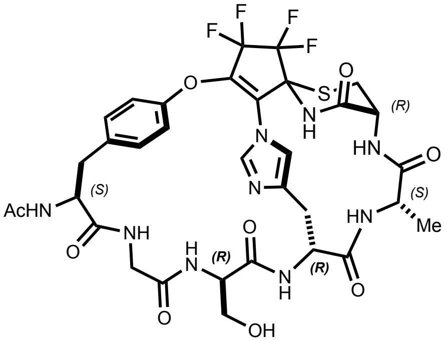

**1d**	Ac-YG(N-Me)ShAC-NH$_2$	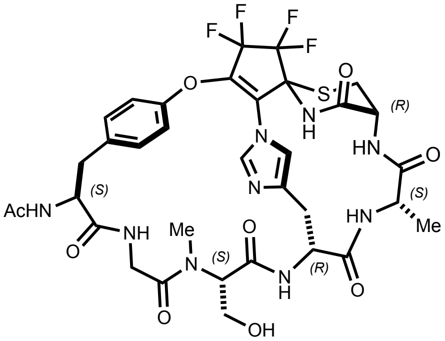
**1e**	Ac-YG(N-Me)SHAC-NH$_2$	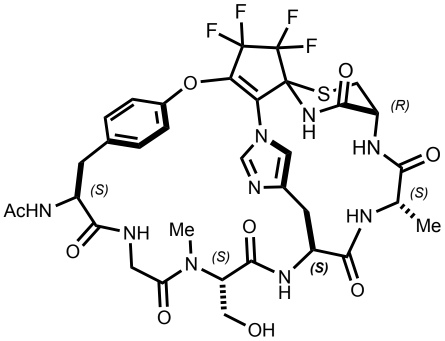
**1f**	Dat-GShAC-NH$_2$	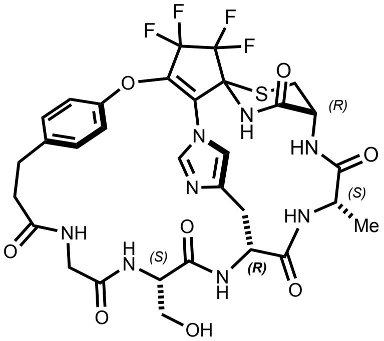
**1g**	Dat-GSHAC-NH$_2$	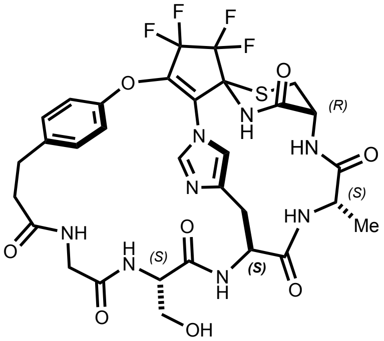

**1h**	Dat-SShAC-NH_2	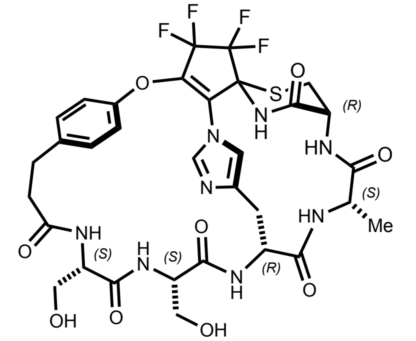
**1i**	Dat-GShSC-NH_2	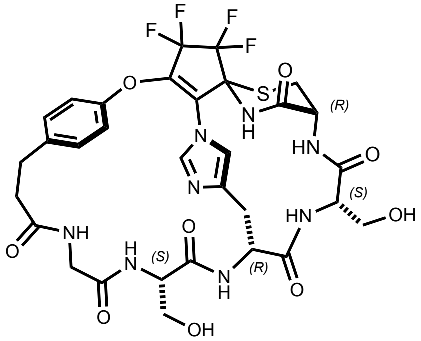
**1j**	Dat-SShSC-NH_2	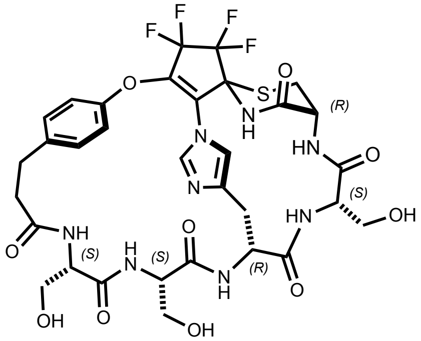
**1k**	Dat-GD*hAC-NH_2	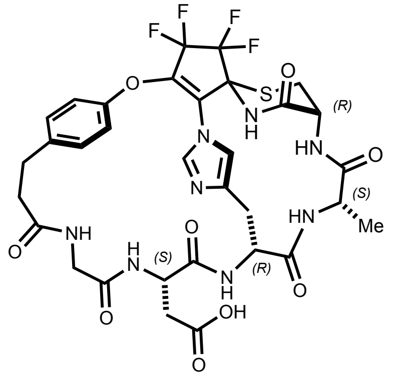

**1l**	Dat-GWhAC-NH$_2$	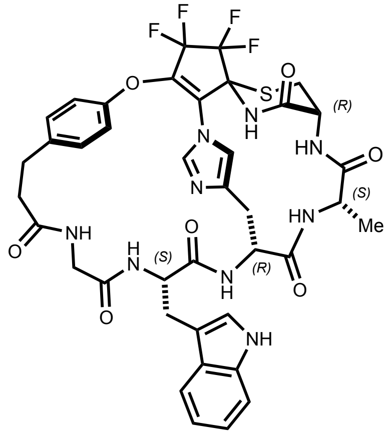
**1m**	Dat-GShNC-NH$_2$	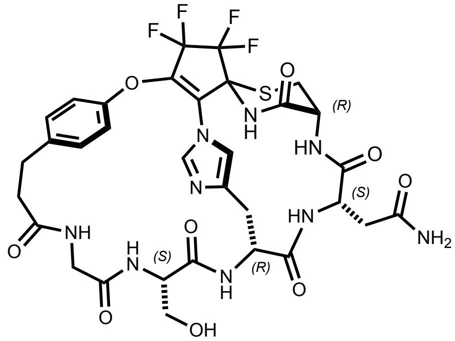
**1n**	Dat-NShNC-NH$_2$	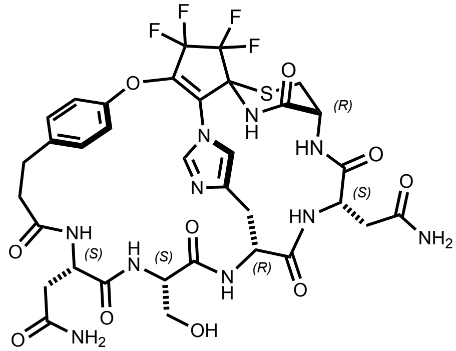
**1o**	Dat-PShAC-NH$_2$	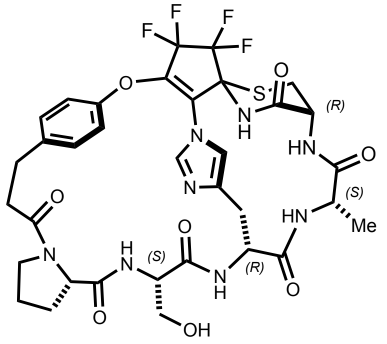

**1p**	Dat-GsHAC-NH_2	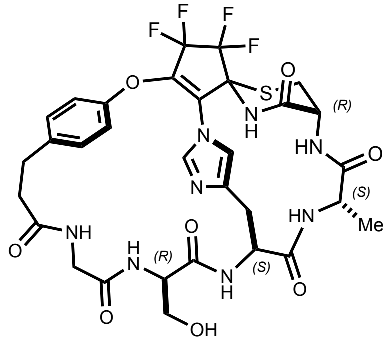
**1q**<br>(Compound 12, *J. Am. Chem. Soc.*, **2023**, *145* (29), 15888-15895.)	Ac-YGHAC-NH_2	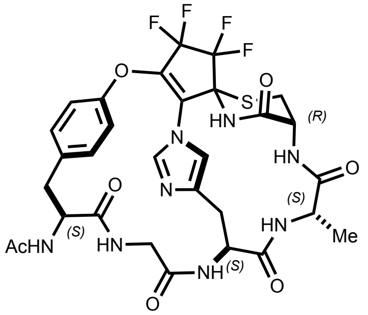
**1r**	Ac-HAC-NH_2	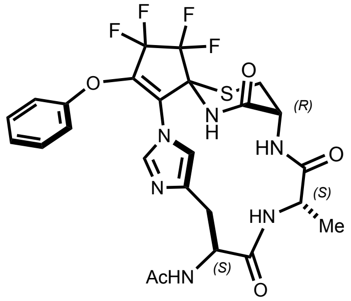
**1s**	Ac-YGScAC-NH_2	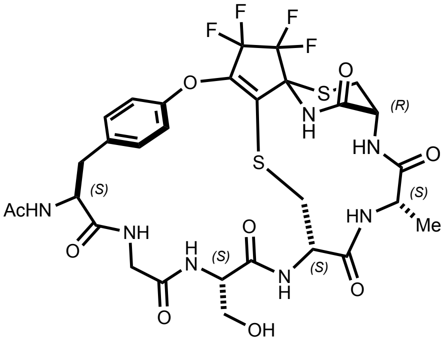
**1t**	Ac-YGSh*AC-NH_2	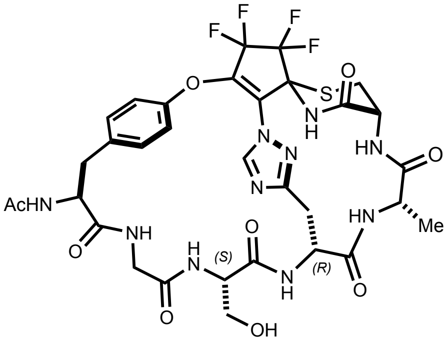

**1u**	Ac-YGSH*AC-NH_2	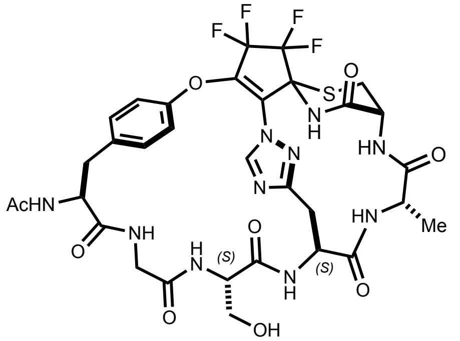

Polycycle 1b.

Synthesized according to **General Procedure B** with analytically pure peptide Ac-YGsHAC-NH_2•TFA (62.5 mg, 1.0 equiv., 78.9 µmol). Purification by silica gel FCC (10-15% MeOH/$CHCl_3$) afforded the title compound (61.4 mg, 96% isolated yield) as an amorphous white solid. **^{1}H NMR** (500 MHz, DMSO) δ 8.94 (bs, 1H), 8.82 (d, *J* = 6.5 Hz, 1H), 8.40 (d, *J* = 9.4 Hz, 1H), 8.26 (d, *J* = 7.6 Hz, 1H), 8.06 (d, *J* = 5.0 Hz, 1H), 7.25 (s, 1H), 7.23 (d, *J* = 1.3 Hz, 1H), 7.18 – 7.09 (m, 3H), 7.03 (d, *J* = 8.3 Hz, 2H), 6.07 (d, *J* = 9.3 Hz, 1H), 4.92 (t, *J* = 5.6 Hz, 1H), 4.70 (dt, *J* = 9.0, 4.1 Hz, 1H), 4.59 (dt, *J* = 9.4, 2.9 Hz, 1H), 4.49 (ddd, *J* = 11.7, 7.7, 4.5 Hz, 1H), 4.10 (td, *J* = 6.8, 3.5 Hz, 1H), 4.00 (dd, *J* = 17.7, 5.6 Hz, 1H), 3.96 – 3.84 (m, 1H), 3.76 – 3.57 (m, 3H), 3.41 (dd, *J* = 14.5, 2.6 Hz, 1H), 3.31 – 3.26 (m, 1H), 2.98 – 2.66 (m, 3H), 2.47 – 2.39 (m, 1H), 1.83 (s, 3H), 1.27 (d, *J* = 7.4 Hz, 3H). **^{13}C NMR** (126 MHz, DMSO) δ 171.3, 170.2, 169.6, 168.9, 168.8, 168.6, 166.9, 154.3, 138.4, 137.5, 134.2, 131.1, 125.9, 119.3, 118.5, 116.2, 116.1, 67.3, 60.7, 57.4, 54.0, 51.0, 50.6, 45.8, 41.8, 37.1, 30.1, 28.7, 22.4, 17.0. **^{19}F NMR** (376 MHz, DMSO) δ -106.75 (dd, *J* = 256.7, 14.8 Hz, 1F), -116.34 (dd, *J* = 224.6, 8.3 Hz, 1F), -122.79 (dd, *J* = 257.5, 9.1 Hz, 1F), -125.57 (dd, *J* = 225.8, 14.7 Hz, 1F). **HRMS** (QTOF) m/z: $[M+H]^+$ calc'd for $C_{33}H_{36}F_4N_9O_9S^+$ 810.2287, found 810.2289; 0.2 ppm mass defect.

Polycycle 1c.

Synthesized according to **General Procedure B** with crude peptide Ac-YGshAC-NH_2•TFA (330 mg, 1.0 equiv., 487 µmol). Purification by silica gel FCC (10-15% MeOH/$CHCl_3$) afforded the title compound (62.0 mg, 16% isolated yield) as an amorphous white solid. **^{1}H NMR** (500 MHz, MeOD) δ 7.24 (dt, *J* = 9.3, 2.9 Hz, 1H), 7.11 – 6.92 (m, 3H), 5.54 – 5.44 (m, 1H), 4.77 (t, *J* = 3.1 Hz, 1H), 4.64 – 4.54 (m, 2H), 4.55 – 4.48 (m, 2H), 4.32 – 4.18 (m, 2H), 3.99 – 3.89 (m, 2H), 3.79 (dd, *J* = 17.3, 3.1 Hz, 1H), 3.54 (dd, *J* = 14.7, 3.0 Hz, 1H), 3.27 (dd, *J* = 15.4, 3.3 Hz, 1H), 3.15 (dt, *J* = 14.0, 3.5 Hz, 1H), 3.00 (ddd, *J* = 14.0, 6.8, 3.0 Hz, 1H), 2.86 (ddt, *J* = 15.1, 4.8, 2.0 Hz, 1H), 2.77 (dt, *J* = 14.6, 3.3 Hz, 1H), 2.05 (s, 3H), 1.31 (dd, *J* = 7.4, 3.1 Hz, 3H). **^{13}C NMR** (126 MHz, MeOD) δ 174.9, 173.8, 173.4, 172.6, 172.5, 172.5, 169.3, 155.4, 141.6, 139.3, 135.4, 132.6, 118.7, 117.5, 61.6, 57.4, 56.6, 55.1, 54.8, 52.2, 48.0, 43.5, 37.1, 31.1, 30.7, 29.5, 22.8, 16.8. **^{19}F NMR** (376 MHz, MeOD) δ -108.64 (dd, *J* = 258.7, 9.6 Hz, 1F), -117.80 (dd, *J* = 231.2, 8.0 Hz, 1F), -123.18 (d, *J* = 258.1 Hz, 1F), -129.51 (d, *J* = 231.6 Hz, 1F). **HRMS** (QTOF) m/z: $[M+H]^+$ calc'd for $C_{33}H_{36}F_4N_9O_9S^+$ 810.2287, found 810.2327; 4.9 ppm mass defect.

Polycycle 1d.

Synthesized according to **General Procedure B** with crude peptide Ac-YGS(N-Me)hAC-NH_2•TFA (50.0 mg, 1.0 equiv., 62.1 µmol). Purification by preparative-reverse phase HPLC (gradient 60% MeCN/H_2O → 100% MeCN/H_2O) afforded the title compound (6.2 mg, 12% isolated yield) as an off-white powder. **HRMS** (QTOF) m/z: $[M+H]^+$ calc'd for $C_{34}H_{38}F_4N_9O_9S^+$ 824.2444, found 824.2349; 11.5 ppm mass defect.

Polycycle 1e.

Synthesized according to **General Procedure B** with crude peptide Ac-YGS(N-Me)HAC-NH_2•TFA (132.0 mg, 1.0 equiv., 191 µmol). Purification by preparative-reverse phase HPLC (gradient 60% MeCN/H_2O → 100% MeCN/H_2O) afforded the title compound (12.0 mg, 8% isolated yield) as an off-white powder. **^{1}H NMR** (500 MHz, MeOD) δ 7.32 (s, 1H), 7.12 (d, *J* = 8.7 Hz, 2H), 7.08 (d, *J* = 1.3 Hz, 1H), 7.00 (d, *J* = 8.6 Hz, 2H), 4.80 (t, *J* = 4.0 Hz, 1H), 4.77 (t, *J* = 3.0 Hz, 1H), 4.59 (bs, 1H), 4.45 (d, *J* = 16.6 Hz, 1H), 4.36 (dd, *J* = 11.4, 3.6 Hz, 1H), 4.28 (dd, *J* = 9.3, 5.2 Hz, 1H), 4.15 – 4.00 (m, 2H), 3.97 (q, *J* = 7.3 Hz, 1H), 3.76 (d, *J* = 16.6 Hz, 1H), 3.51 (dd, *J* = 14.7, 2.8 Hz, 1H), 3.20 (dd, *J* = 15.1, 3.9 Hz, 1H), 3.16 (s, 3H), 3.01 – 2.90 (m, 1H), 2.84 (ddd, *J* = 13.0, 7.7, 3.4 Hz, 2H), 2.77 (dd, *J* = 14.8, 4.3 Hz, 1H), 1.98 (s, 3H), 1.45 (d, *J* = 7.4 Hz, 3H). **^{13}C NMR** (126 MHz, MeOD) δ 174.3, 173.5, 172.7, 172.1, 170.8, 170.6, 169.5, 153.5, 140.7, 139.3, 136.9, 132.1, 120.6, 119.5, 65.8, 59.9, 56.9, 53.4, 53.3, 43.3, 38.7, 36.8, 31.1, 30.7, 29.4, 22.3, 16.7. **^{19}F NMR** (376 MHz, MeOD) δ -109.23 (d, *J* = 258.4 Hz, 1F), -119.14 (dd, *J* = 229.2, 8.3 Hz, 1F), -125.63 (dd, *J* = 258.8, 8.0 Hz, 1F), -130.08 (dd, *J* = 228.5, 12.4 Hz, 1F). **HRMS** (QTOF) m/z: $[M+H]^+$ calc'd for $C_{34}H_{38}F_4N_9O_9S^+$ 824.2444, found 824.2498; 6.6 ppm mass defect.

Polycycle 1f.

Synthesized according to **General Procedure B** with crude peptide Dat-GShAC-NH_2•TFA (167.0 mg, 1.0 equiv., 269 µmol). Purification by silica gel FCC (5-15% MeOH/$CHCl_3$) afforded the title compound (63.0 mg, 31% isolated yield) as an amorphous white solid. **^{1}H NMR** (500 MHz, MeOD) δ 7.10 – 7.00 (m, 3H), 6.98 – 6.89 (m, 3H), 4.78 (t, *J* = 3.0 Hz, 1H), 4.59 (bs, 1H), 4.54 (dd, *J* = 5.2, 2.9 Hz, 1H), 4.36 (d, *J* = 15.3 Hz, 1H), 4.19 (dd, *J* = 3.2, 2.1 Hz, 1H), 4.10 (q, *J* = 7.4 Hz, 1H), 4.00 (dd, *J* = 10.5, 3.2 Hz, 1H), 3.85 (dd, *J* = 10.5, 2.2 Hz, 1H), 3.64 (d, *J* = 15.3 Hz, 1H), 3.50 (dd, *J* = 14.6, 2.7 Hz, 1H), 3.24 (dd, *J* = 14.7, 3.0 Hz, 1H), 2.92 – 2.77 (m, 3H), 2.72 – 2.57 (m, 2H), 2.21 (ddd, *J* = 13.5, 10.8, 5.0 Hz, 1H), 1.21 (d, *J* = 7.4 Hz, 3H). **^{13}C NMR** (126 MHz, MeOD) δ 174.5, 173.6, 173.5, 172.9, 172.4, 169.1, 152.6, 142.2, 140.6, 139.2, 131.2, 119.1, 117.8, 116.5, 70.0, 62.3, 58.4, 54.7, 52.4, 48.5, 47.6, 43.3, 40.3, 32.4, 31.1, 28.6, 17.1. **^{19}F NMR** (376 MHz, MeOD) δ -109.22 – -114.65 (m, 1F), -117.38 (dd, *J* = 232.7, 8.4 Hz, 1F), -120.43 – -125.19 (m, 1F), -128.79 (d, *J* = 232.6 Hz, 1F). **HRMS** (QTOF) m/z: $[M+H]^+$ calc'd for $C_{31}H_{33}F_4N_8O_8S^+$ 753.2073, found 753.2075; 0.3 ppm mass defect.

Polycycle 1g.

Synthesized according to **General Procedure B** with crude peptide Dat-GSHAC-NH_2•TFA (156.0 mg, 1.0 equiv., 251 µmol). Purification by silica gel FCC (5-15% MeOH/$CHCl_3$) afforded the title compound (18.0 mg, 10% isolated yield) as an amorphous white solid. **^{1}H NMR** (500 MHz, MeOD) δ 7.26 (d, *J* = 1.2 Hz, 1H), 7.07 (d, *J* = 8.6 Hz, 2H), 6.99 – 6.92 (m, 3H), 4.78 (t, *J* = 3.0 Hz, 1H), 4.64 (dd, *J* = 6.4, 3.7 Hz, 1H), 4.59 (bs, 1H), 4.46 (dd, *J* = 6.8, 5.2 Hz, 1H), 4.33 (d, *J* = 16.5 Hz, 1H), 4.12 (q, *J* = 7.3 Hz, 1H), 3.92 (dd, *J* = 11.3, 5.2 Hz, 1H), 3.84 (dd, *J* = 11.3, 6.8 Hz, 1H), 3.60 (d, *J* = 16.6 Hz, 1H), 3.52 (dd, *J* = 14.6, 2.6 Hz, 1H), 3.08 (dd, *J* = 15.2, 3.7 Hz, 1H), 2.94 – 2.81 (m, 4H), 2.61 (ddd, *J* = 14.3, 6.7, 4.4 Hz, 1H), 2.39 (ddd, *J* = 14.5, 9.5, 5.2 Hz, 1H), 1.40 (d, *J* = 7.3 Hz, 3H). **^{13}C NMR** (126 MHz, MeOD) δ 175.1, 174.2, 172.6, 171.9, 171.9, 169.4, 153.4, 144.8, 141.3, 141.0, 138.9, 131.1, 119.9, 119.3, 118.2, 112.4, 69.1, 61.5, 56.2, 53.6, 52.5, 48.0, 44.0, 39.2, 31.9, 31.2, 30.2, 17.1. **^{19}F NMR** (376 MHz, MeOD) δ -108.98 (dd, *J* = 258.5, 13.1 Hz, 1F), -119.28 (dd, *J* = 228.6, 8.3 Hz, 1F), -125.49 (dd, *J* = 257.8, 8.0 Hz, 1F), -129.44 (dd, *J* = 228.5, 13.1 Hz, 1F). **HRMS** (QTOF) m/z: $[M+H]^+$ calc'd for $C_{31}H_{33}F_4N_8O_8S^+$ 753.2073, found 753.2075; 0.3 ppm mass defect.

Polycycle 1h.

Synthesized according to **General Procedure B** with crude peptide Dat-SShAC-NH_2•TFA (100.0 mg, 1.0 equiv., 154 µmol). Purification by silica gel FCC (5-15% MeOH/$CHCl_3$) afforded the title compound (3.4 mg, 3% isolated yield) as an amorphous white solid. **HRMS** (QTOF) m/z: $[M+H]^+$ calc'd for $C_{32}H_{35}F_4N_8O_9S^+$ 783.2178, found 783.2229; 6.5 ppm mass defect.

Polycycle 1i.

Synthesized according to **General Procedure B** with crude peptide Dat-GShSC-NH_2•TFA (100.0 mg, 1.0 equiv., 314 μmol). Purification by silica gel FCC (5-15% MeOH/$CHCl_3$) afforded the title compound (5.2 mg, 4% isolated yield) as an amorphous white powder. **HRMS** (QTOF) m/z: $[M+H]^+$ calc'd for $C_{31}H_{33}F_4N_8O_8S^+$ 769.2022, found 769.2030; 1.0 ppm mass defect.

Polycycle 1j.

Synthesized according to **General Procedure B** with crude peptide Dat-SShSC-NH_2•TFA (100.0 mg, 1.0 equiv., 150 μmol). Purification by silica gel FCC (5-15% MeOH/$CHCl_3$) afforded the title compound (3.6 mg, 3% isolated yield) as an off-white powder. **HRMS** (QTOF) m/z: $[M+H]^+$ calc'd for $C_{32}H_{35}F_4N_8O_{10}S^+$ 799.2127, found 799.2182; 6.9 ppm mass defect.

Polycycle 1k.

Synthesized according to **General Procedure B** with crude peptide Dat-GD*hAC-NH_2•TFA (49.5 mg, 1.0 equiv., 67.6 μmol). Post OFCP processing, the CSY was liberated as described by Bode *et al*.[3] Purification by preparative-reverse phase HPLC (gradient 10% MeCN/H_2O → 100% MeCN/H_2O) afforded the title compound (18.7 mg, 36% isolated yield) as an amorphous white powder. **^{1}H NMR** (600 MHz, DMSO) δ 9.28 (s, 1H), 8.68 (d, *J* = 7.3 Hz, 1H), 8.63 (d, *J* = 7.1 Hz, 1H), 7.28 (d, *J* = 5.7 Hz, 1H), 7.08 (d, *J* = 8.1 Hz, 2H), 7.03 (d, *J* = 5.8 Hz, 1H), 6.96 (s, 1H), 6.92 (d, *J* = 8.1 Hz, 2H), 6.86 (s, 1H), 6.10 (d, *J* = 9.0 Hz, 1H), 4.59 (dd, *J* = 7.7, 4.3 Hz, 1H), 4.38 (td, *J* = 6.9, 3.8 Hz, 1H), 4.28 (dt, *J* = 7.9, 4.0 Hz, 1H), 4.05 (dd, *J* = 16.3, 7.1 Hz, 1H), 4.01 – 3.94 (m, 1H), 3.57 (dd, *J* = 16.3, 2.7 Hz, 1H), 3.50 – 3.43 (m, 1H), 3.17 (s, 3H), 3.00 (dd, *J* = 14.8, 3.2 Hz, 1H), 2.93 – 2.85 (m, 1H), 2.82 – 2.72 (m, 2H), 2.67 (dd, *J* = 14.8, 5.0 Hz, 1H), 2.65 – 2.57 (m, 2H), 2.43 (dt, *J* = 9.0, 4.6 Hz, 1H), 2.34 (td, *J* = 14.5, 4.4 Hz, 1H), 1.13 (d, *J* = 7.5 Hz, 3H). **^{13}C NMR** (151 MHz, DMSO) δ 172.5, 171.4, 170.9, 170.8, 170.8, 169.4, 166.9, 158.0, 152.4, 139.4, 138.4, 138.0, 137.5, 130.3, 116.6, 116.3, 64.6, 53.4, 50.3, 50.1, 48.6, 45.8, 42.4, 40.1, 37.6, 35.1, 30.6, 29.9, 27.8, 16.7. **^{19}F NMR** (565 MHz, DMSO) δ -107.93 (d, *J* = 256.2 Hz, 1F), -114.79 (d, *J* = 228.7 Hz, 1F), -118.77 (d, *J* = 256.5 Hz, 1F), -124.87 (d, *J* = 228.9 Hz, 1F). **HRMS** (QTOF) m/z: $[M+H]^+$ calc'd for $C_{32}H_{33}F_4N_8O_9S^+$ 781.2022, found 781.2067; 5.8 ppm mass defect.

Polycycle 1l.

Synthesized according to **General Procedure B** with crude peptide Dat-GWhAC-NH_2•TFA (100.0 mg, 1.0 equiv., 119 μmol). Purification by silica gel FCC (5-10% MeOH/$CHCl_3$) afforded the title compound (23.5 mg, 23% isolated yield) as an amorphous white powder. **HRMS** (QTOF) m/z: $[M+H]^+$ calc'd for $C_{39}H_{38}F_4N_9O_7S^+$ 852.2546, found 852.2540; 0.7 ppm mass defect.

Polycycle 1m.

Synthesized according to **General Procedure B** with crude peptide Dat-GShNC-NH_2•TFA (100.0 mg, 1.0 equiv., 151 μmol). Purification by preparative-reverse phase HPLC (gradient 10% MeCN/H_2O → 100% MeCN/H_2O) afforded the title compound (1.5 mg, 1% isolated yield) as an amorphous white solid. **HRMS** (QTOF) m/z: $[M+H]^+$ calc'd for $C_{32}H_{34}F_4N_9O_9S^+$ 796.2131, found 796.2173; 5.3 ppm mass defect.

Polycycle 1n.

Synthesized according to **General Procedure B** with crude peptide Dat-NShNC-NH_2•TFA (100.0 mg, 1.0 equiv., 120 μmol). Purification by silica gel FCC (5-15% MeOH/$CHCl_3$) afforded the title compound (3.2 mg, 3% isolated yield) as an amorphous white solid. **HRMS** (QTOF) m/z: $[M+H]^+$ calc'd for $C_{34}H_{37}F_4N_{10}O_{10}S^+$ 853.2345, found 853.2385; 4.7 ppm mass defect.

Polycycle 1o.

Synthesized according to **General Procedure B** with crude peptide Dat-PShAC-NH_2•TFA (156.0 mg, 1.0 equiv., 251 μmol). Purification by silica gel FCC (5-15% MeOH/$CHCl_3$) afforded the title compound (18.0 mg, 10% isolated yield) as an amorphous white solid. **HRMS** (QTOF) m/z: $[M+H]^+$ calc'd for $C_{34}H_{37}F_4N_8O_8S^+$ 793.2386, found 793.2371; 1.9 ppm mass defect.

Polycycle 1p.

Synthesized according to **General Procedure B** with crude peptide Dat-GsHAC-NH_2•TFA (167.2 mg, 1.0 equiv., 227 μmol). Purification by silica gel FCC (5-12.5% MeOH/$CHCl_3$) afforded the title compound (5.2 mg, 3% isolated yield) as a white powder. **HRMS** (QTOF) m/z: $[M+H]^+$ calc'd for $C_{31}H_{33}F_4N_8O_8S^+$ 753.2073, found 753.2091; 2.4 ppm mass defect.

Polycycle 1r.

Synthesized according to **General Procedure B** with crude peptide Ac-HAC-NH_2•TFA (400.0 mg, 1.0 equiv., 826 μmol). After full conversion to vinyl fluoride was observed by HPLC, phenol (2.0 eq) was added. Reaction progress was monitored by HPLC. Purification by silica gel FCC (5-10% MeOH/$CHCl_3$) afforded the title compound (151 mg, 31% isolated yield) as an amorphous white powder, and vinyl fluoride intermediate (47.7 mg, 11% isolated yield) as an amorphous white powder. **^{1}H NMR** (500 MHz, DMSO) δ 9.38 (bs, 1H), 8.49 (d, *J* = 6.7 Hz, 1H), 8.27 (d, *J* = 7.8 Hz, 1H), 7.67 (d, *J* = 1.4 Hz, 1H), 7.43 – 7.15 (m, 2H), 7.23 – 7.08 (m, 3H), 6.95 (s, 1H), 6.67 (d, *J* = 9.3 Hz, 1H), 4.66 (dt, *J* = 9.4, 2.9 Hz, 1H), 4.51 (ddd, *J* = 11.3, 7.8, 2.9 Hz, 1H), 4.09 (p, *J* = 7.3 Hz, 1H), 3.54 (dd, *J* = 14.6, 2.6 Hz, 1H), 2.98 – 2.74 (m, 2H), 2.47 – 2.40 (m, 1H), 1.82 (s, 3H), 1.19 (d, *J* = 7.4 Hz, 3H). **^{13}C NMR** (151 MHz, DMSO) δ 170.9, 170.9, 168.8, 167.2, 162.3, 153.6, 129.9, 125.2, 116.7, 52.2, 49.6, 45.5, 35.8, 30.9, 30.8, 30.2, 22.4, 17.2. **^{19}F NMR** (565 MHz, DMSO) δ -105.14 (dd, *J* = 255.0, 12.2 Hz, 1F), -114.33 (d, *J* = 224.7 Hz, 1F), -119.59 (d, *J* = 254.0 Hz, 1F), -126.77 (dd, *J* = 226.0, 12.1 Hz, 1F). **HRMS** (QTOF) m/z: $[M+H]^+$ calc'd for $C_{25}H_{25}F_4N_6O_5S^+$ 597.1538, found 597.1597; 9.9 ppm mass defect.

Polycycle 1s.

Synthesized according to **General Procedure B** with crude peptide Ac-YGScAC-NH_2 (189.0 mg, 1.0 equiv., 294 μmol). Purification by silica gel FCC (5-15% MeOH/$CHCl_3$) afforded the title compound (19.0 mg, 10% isolated yield) as an off-white solid. **^{1}H NMR** (500 MHz, MeOD) δ 7.22 (d, *J* = 8.2 Hz, 2H), 6.98 (d, *J* = 8.1 Hz, 2H), 4.57 (td, *J* = 8.0, 4.9 Hz, 2H), 4.39 – 4.29 (m, 2H), 4.22 (t, *J* = 3.8 Hz, 1H), 3.93 (dd, *J* = 11.4, 4.4 Hz, 1H), 3.85 (dd, *J* = 11.3, 3.2 Hz, 1H), 3.79 (d, *J* = 4.4 Hz, 1H), 3.51 (dd, *J* = 13.8, 7.1 Hz, 1H), 3.44 – 3.36 (m, 2H), 3.37 – 3.33 (m, 1H), 3.28 (d, *J* = 4.8 Hz, 1H), 3.11 (dd, *J* = 13.2, 4.2 Hz, 1H), 2.98 (dd, *J* = 13.2, 10.5 Hz, 1H), 2.08 (s, 3H), 1.35 (d, *J* = 7.1 Hz, 3H). **^{13}C NMR** (126 MHz, MeOD) δ 174.1, 174.0, 174.0, 173.9, 173.4, 172.3, 171.7, 151.8, 137.0, 132.1, 123.6, 112.4, 99.9, 75.8, 62.6, 58.3, 58.1, 56.3, 52.9, 51.4, 44.6, 38.2, 36.8, 36.1, 30.7, 22.5, 16.0. **^{19}F NMR** (376 MHz, MeOD) δ -103.97 (dd, *J* = 257.3, 11.4 Hz), -118.03 – -119.39 (m), -119.90 (d, *J* = 257.3 Hz), -126.94 (dt, *J* = 228.6, 12.3 Hz), -139.52 (d, *J* = 226.0 Hz). **HRMS** (QTOF) m/z: $[M+H]^+$ calc'd for $C_{30}H_{34}F_4N_7O_9S_2^+$ 776.1790, found 776.1809; 2.4 ppm mass defect.

Polycycle 1t.

Synthesized according to **General Procedure B** with analytically pure peptide Ac-YGSh*AC-NH_2•TFA (7.2 mg, 1.0 equiv., 9.1 μmol). Purification by PTLC (7% MeOH/$CHCl_3$) afforded the title compound (4.3 mg, 58% isolated yield) as a sparkly white solid. **^{1}H NMR** (500 MHz, DMSO) δ 9.15 (s, 1H), 8.92 (d, *J* = 7.8 Hz, 1H), 8.84 (d, *J* = 8.4 Hz, 1H), 8.60 (td, *J* = 7.5, 2.7 Hz, 1H), 8.30 (d, *J* = 8.0 Hz, 1H), 8.08 (s, 1H), 7.65 (d, *J* = 7.5 Hz, 1H), 7.34 – 7.15 (m, 3H), 6.83 (d, *J* = 8.7 Hz, 2H), 5.00 (ddd, *J* = 10.6, 8.4, 2.1 Hz, 1H), 4.80 (t, *J* = 5.8 Hz, 1H), 4.68 (dt, *J* = 10.0, 2.8 Hz, 1H), 4.33 (ddd, *J* = 10.9, 8.0, 3.4 Hz, 1H), 4.29 – 4.18 (m, 2H),

3.86 (dd, J = 15.8, 7.9 Hz, 1H), 3.66 – 3.49 (m, 4H), 3.19 (dd, J = 16.3, 10.5 Hz, 1H), 2.78 (dd, J = 14.2, 3.3 Hz, 2H), 2.58 – 2.53 (m, 2H), 2.21 (d, J = 16.0 Hz, 1H), 2.05 – 1.91 (m, 1H), 1.75 (s, 3H), 1.26 (d, J = 7.3 Hz, 3H). **^{13}C NMR** (126 MHz, DMSO) δ 172.2, 170.5, 170.2, 169.4, 169.0, 169.0, 167.4, 162.7, 160.6, 139.9, 136.6, 135.5, 130.8, 129.6, 117.8, 117.3, 61.6, 54.8, 49.7, 48.4, 45.8, 42.9, 31.3, 28.7, 26.6, 24.5, 22.3, 17.0, 14.0. **^{19}F NMR** (565 MHz, DMSO) δ -105.15 (d, J = 254.0 Hz, 1F), -117.37 (d, J = 221.9 Hz, 1F), -123.34 (d, J = 258.0 Hz, 1F), -129.49 (d, J = 226.9 Hz, 1F). **HRMS** (QTOF) m/z: $[M+H]^+$ calc'd for $C_{32}H_{35}F_4N_{10}O_9S^+$ 811.2240, found 811.2249; 1.1 ppm mass defect.

Polycycle 1u.

Synthesized according to **General Procedure B** with crude peptide Ac-YGSH*AC-NH_2•TFA (150.0 mg, 1.0 equiv., 189 μmol). Purification by silica gel FCC (5-15% MeOH/$CHCl_3$) afforded the title compound (2.1 mg, 3% isolated yield) as a sparkly white solid. **HRMS** (QTOF) m/z: $[M+H]^+$ calc'd for $C_{32}H_{35}F_4N_{10}O_9S^+$ 811.2240, found 811.2255; 1.9 ppm mass defect.

Synthesis of 1,2,4-Triazoyl Analog of Histidine (H*)

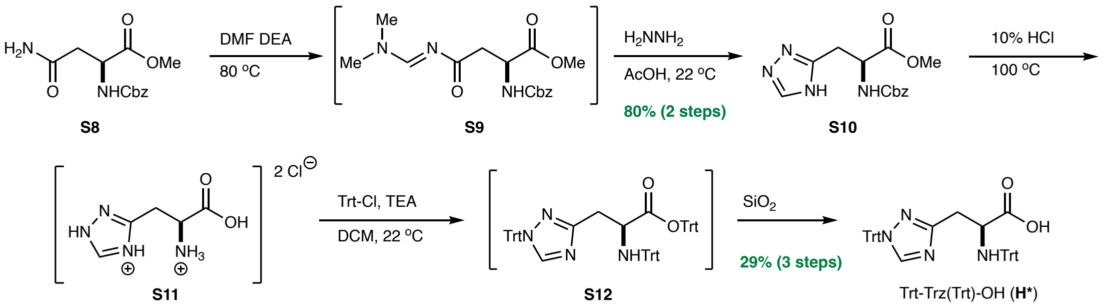

Compound S9.

Prepared analogously to the Boc-Trz-OBn reported by Zhao et al.[4] Cbz-Asn-OMe (**S8**, 5.00 g, 1.0 equiv., 17.8 mmol) was suspended in freshly distilled dimethylformamide diethylacetal (DMF DEA, 0.15 M, 119 mL) under inert atmosphere. The suspension was heated to 80 °C, which allowed the suspension to dissolve. After 1 hour, ^{1}H NMR of the crude reaction indicated full conversion to **S9**. The reaction was concentrated *in vacuo* and the residue was used directly in the next step. **^{1}H NMR** (600 MHz, $CDCl_3$) δ 8.39 (s, 1H), 7.41 – 7.28 (m, 5H), 6.09 (d, J = 9.1 Hz, 1H), 5.26 – 4.98 (m, 2H), 4.62 (dt, J = 8.9, 4.3 Hz, 1H), 3.72 (s, 3H), 3.16 (dd, J = 17.4, 4.4 Hz, 1H), 3.11 (s, 3H), 3.04 (s, 2H), 2.88 (dd, J = 17.3, 4.4 Hz, 1H).

Compound S10

The crude residue **S9** was dissolved in glacial acetic acid (0.06 M, 297 mL) and hydrazine monohydrate (1.73 mL, 2.0 equiv., 35.6 mmol) was added dropwise under inert atmosphere. The reaction was stirred at room temperature for 16 hours, then concentrated *in vacuo* (***Caution!*** H_2NNH_2 is an inhalation toxin. Proper ventilation must be utilized). The residue was diluted with EtOAc and washed with water. The aqueous layer was extracted 3x, then combined organic layers were washed with brine, dried over Na_2SO_4, filtered, and concentrated. The crude residue was purified by silica gel FCC 50-100% EtOAc/Hex, giving the title compound (4.32 g, 80%) as a white powder. **^{1}H NMR** (600 MHz, $CDCl_3$) δ 8.10 (s, 1H), 7.46 – 7.28 (m, 5H), 6.14 (d, J = 8.5 Hz, 1H), 5.17 – 5.04 (m, 2H), 4.81 (d, J = 8.4 Hz, 1H), 3.73 (s, 3H), 3.41 (dd, J = 15.5, 5.7 Hz, 1H), 3.36 (dd, J = 15.5, 4.7 Hz, 1H).

Compound S11

Compound **S10** (4.86 g, 1.0 equiv., 12.8 mmol) was suspended in 10% aq. HCl (100 mL, 0.16 M) then heated to reflux under inert atmosphere. After 16 hours, ^{1}H NMR of the crude reaction indicated full conversion to **S11**. The reaction was then cooled to room temperature and washed 3x with DCM. The aqueous layer was then concentrated to dryness, producing a white foam (***Note:*** this material is very hydroscopic, it can be dried by stripping with anhydrous toluene, but exposure to atmospheric moisture will melt the solid in minutes). This material was subjected directly to the next step without further purification. ^{1}**H NMR** (600 MHz, MeOD) δ 9.40 (s, 1H), 4.57 (dd, *J* = 7.0, 6.0 Hz, 1H), 3.64 (dd, *J* = 16.3, 6.0 Hz, 1H), 3.56 (dd, *J* = 16.3, 7.0 Hz, 1H).

Trt-Trz(Trt)-OH (H*)

Crude **S11** (3.66 g, 1.0 equiv., 16.0 mmol) was suspended in anhydrous DCM (40 mL, 0.4 M) and TEA (13.4 mL, 6.0 equiv., 96 mmol) was added slowly under argon. In a separate flask, TrtCl (13.4 g, 3.0 eq, 48 mmol) was diluted with anhydrous DCM (60 mL). The TrtCl solution was then added to the reaction flask containing **S11** via cannula over 5 minutes. The reaction was stirred at room temperature overnight. Then, the reaction was diluted with DCM, and washed 3x with 5% citric acid, then brine. The organic layer was dried with $MgSO_4$, filtered, and concentrated. The crude residue was then purified by silica gel FCC (0-75% EtOAc/Hex), which hydrolyzed the Trt-ester to yield **H*** as a sparkly white solid (2.98 g, 29% yield). ^{1}**H NMR** (500 MHz, $CDCl_3$) δ 7.83 (s, 1H), 7.47 – 7.27 (m, 15H), 7.22 – 7.11 (m, 9H), 7.10 – 7.06 (m, 6H), 3.70 (dd, *J* = 5.8, 3.2 Hz, 1H), 3.10 (dd, *J* = 15.4, 3.2 Hz, 1H), 2.11 – 2.05 (m, 1H). ^{13}**C NMR** (126 MHz, $CDCl_3$) δ 174.2, 160.6, 146.0, 145.3, 141.8, 130.2, 130.0, 128.6, 128.4, 128.3, 128.2, 126.9, 78.3, 71.8, 55.6, 30.3. $[\boldsymbol{\alpha}]_{\boldsymbol{D}}^{\boldsymbol{20}}$ = +184.00 (c = 0.53, $CHCl_3$). **HRMS** (DART) m/z: $[M\text{-}Trt]^-$ calc'd for $C_{24}H_{21}N_4O_2^-$ 397.1670, found 397.1669; 0.3 ppm mass defect.

Trt-trz(Trt)-OH (h*)

Prepared analogously to **H***, deriving from D-Asn. ^{1}**H NMR** (500 MHz, $CDCl_3$) δ 7.83 (s, 1H), 7.45 – 7.28 (m, 15H), 7.21 – 7.12 (m, 9H), 7.11 – 7.00 (m, 6H), 3.70 (dd, *J* = 5.7, 3.2 Hz, 1H), 3.10 (dd, *J* = 15.6, 3.2 Hz, 1H), 2.08 – 1.95 (m, 1H). ^{13}**C NMR** (126 MHz, $CDCl_3$) δ 174.2, 160.6, 146.0, 145.3, 141.8, 130.0, 130.0, 128.6, 128.4, 128.4, 128.3, 128.3, 128.2, 128.1, 127.0, 78.3, 71.9, 55.6, 30.1. $[\boldsymbol{\alpha}]_{\boldsymbol{D}}^{\boldsymbol{20}}$ = -126.00 (c = 0.54, $CHCl_3$). **HRMS** (DART) m/z: $[M+H]^+$ calc'd for $C_{43}H_{37}N_4O_2^+$ 641.2911, found 641.2910; 0.2 ppm mass defect.

Polycycle 2 Analogs

Compound	Linear Precursor	Structure
**2**<br>(Compound 15, *J. Am. Chem. Soc.*, **2023**, *145* (29), 15888-15895.)	Ac-D(C)VHSAY-NH_2	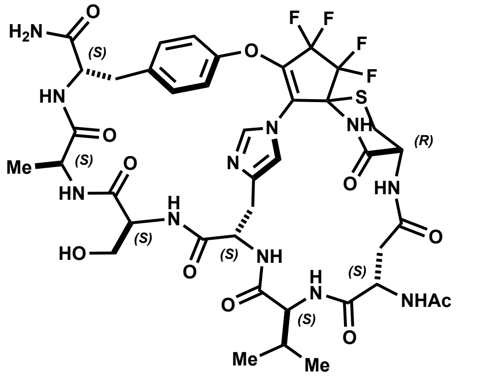
**2a**	H-D(C)VHSAY-NH_2	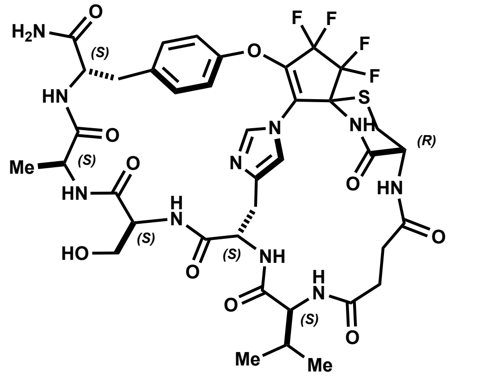
**2b**	Ac-D(C)VHSP*Y-NH_2	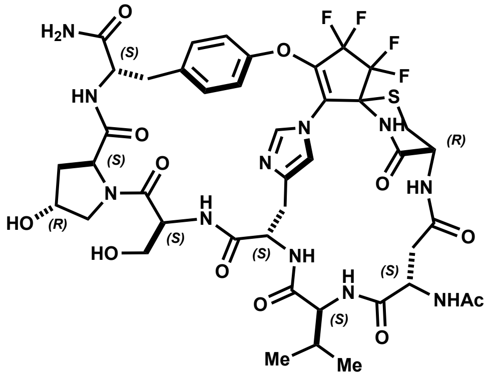

Polycycle 2a.

Synthesized according to **General Procedure B** with crude peptide Ac-D(C)VHSAY-NH_2•TFA (300 mg, 1.0 equiv., 386 μmol). Purification by preparative-reverse phase HPLC (gradient 10% MeCN/H_2O → 100% MeCN/H_2O) afforded the title compound (5.1 mg, 2% isolated yield) as an off-white powder. **HRMS** (QTOF) m/z: $[M+H]^+$ calc'd for $C_{38}H_{45}F_4N_{10}O_{10}S^+$ 909.2971, found 909.2957; 1.5 ppm mass defect.

Polycycle 2b.

Synthesized according to **General Procedure B** with crude peptide H-D(C)VHSAY-NH_2•TFA (130 mg, 1.0 equiv., 148 μmol). Purification by preparative-reverse phase HPLC (gradient 10% MeCN/H_2O → 100% MeCN/H_2O) afforded the title compound (1.7 mg, 1% isolated yield) as an off-white powder. **HRMS** (QTOF) m/z: $[M+H]^+$ calc'd for $C_{42}H_{50}F_4N_{11}O_{12}S^+$ 1008.3292, found 1008.3323; 3.1 ppm mass defect.

Polycycle 3 Analogs

Compound	Linear Precursor	Nucleophile	Structure
**3** (Compound 46, *J. Am. Chem. Soc.*, **2023**, *145* (29), 15888-15895.)	Ac-D*PTFSC-NH_2	Cysteamine	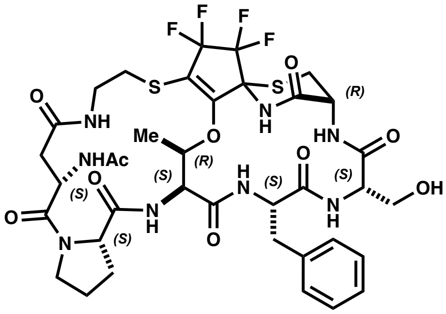
**3a**	Ac-D*PTFSC-NH_2	2-Mercaptoethanol	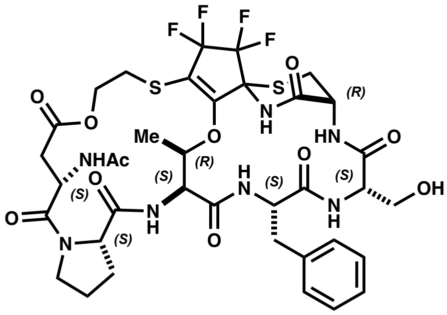
**3b** (Compound 49, *J. Am. Chem. Soc.*, **2023**, *145* (29), 15888-15895.)	Ac-D*PTFSC-NH_2	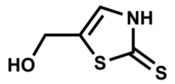 (*J. Am. Chem. Soc.*, **2023**, *145* (29), 15888-15895.)	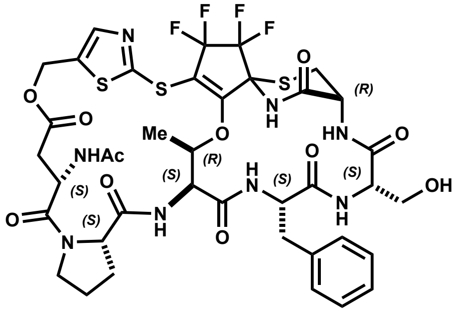
**3c**	Ac-D*A(N-Me)TFSC-NH_2	Cysteamine	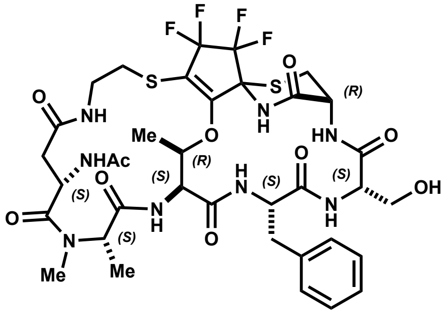
**3d**	Ac-D*P-alloT-FSC-NH_2	Cysteamine	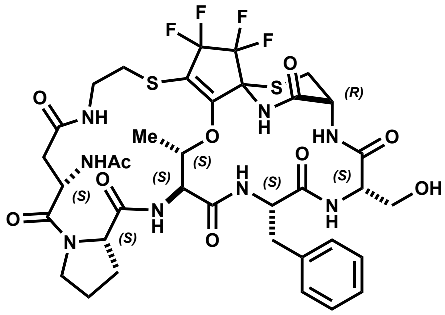

**3e**	Ac-D*PTFS-homoC-NH_2	Cysteamine	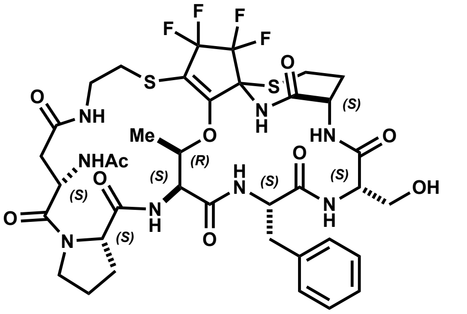
**3f** (Compound 44, *J. Am. Chem. Soc.*, **2023**, *145* (29), 15888-15895.)	Ac-E*PSFTC-NH_2	Cysteamine	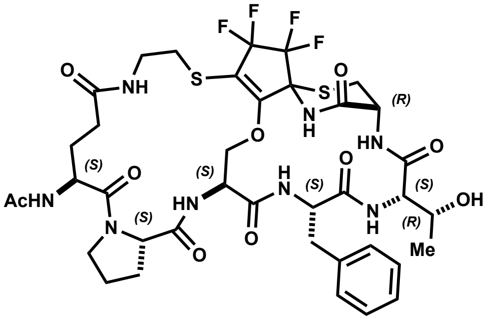
**3g** (*J. Am. Chem. Soc.*, **2023**, *145* (29), 15888-15895.)	Ac-E*PSFTC-NH_2	Cysteamine	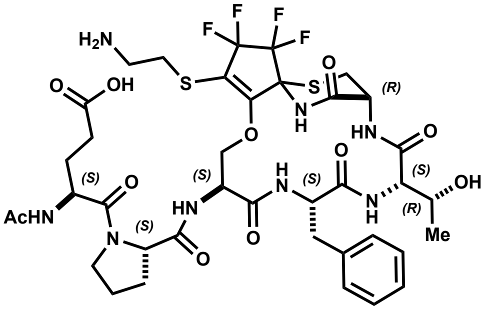
**3h** (Compound 47, *J. Am. Chem. Soc.*, **2023**, *145* (29), 15888-15895.)	Ac-E*PSFTC-NH_2	2-Mercaptoethanol	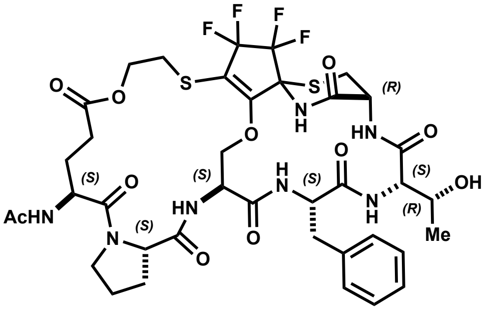
**3i** (*J. Am. Chem. Soc.*, **2023**, *145* (29), 15888-15895.)	Ac-E*PSFTC-NH_2	2-Mercaptoethanol	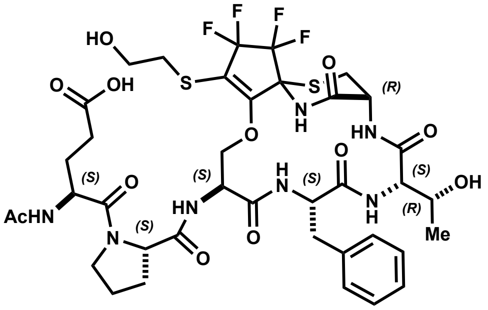

**3j**	Ac-E*PSFTC-NH2	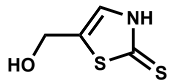 (*J. Am. Chem. Soc.*, **2023**, *145* (29), 15888-15895.)	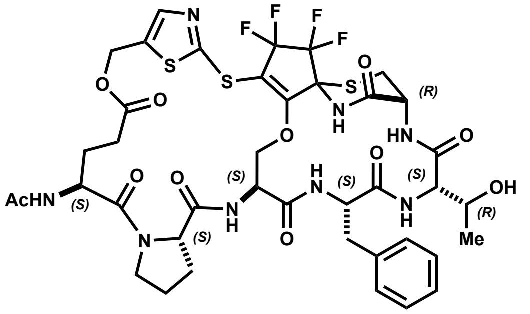
**3k**	Ac-E*A(N-Me)SFTC-NH2	Cysteamine	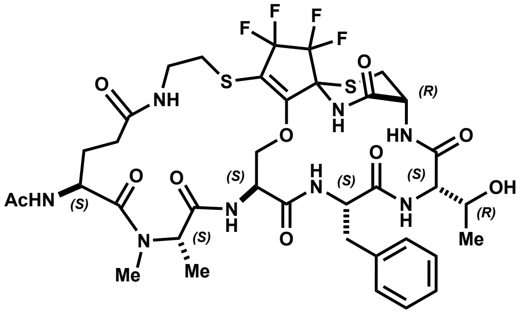
**3l**	Ac-E*PSFT-homoC-NH2	Cysteamine	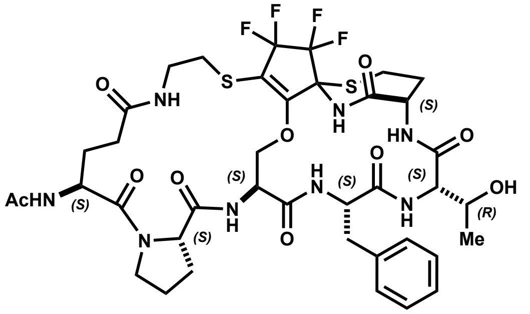
**3m** (Compound 45, *J. Am. Chem. Soc.*, **2023**, *145* (29), 15888-15895.)	Ac-E*VSFAC-NH2	Cysteamine	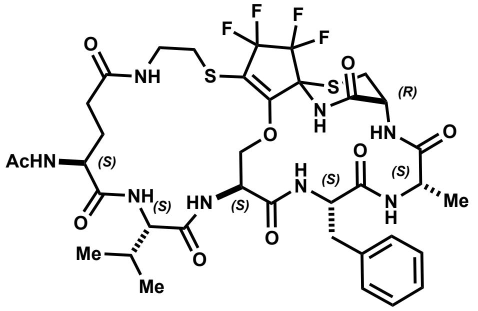
**3n** (Compound 41, *J. Am. Chem. Soc.*, **2023**, *145* (29), 15888-15895.)	Ac-E*A(N-Me)SFAC-NH2	Cysteamine	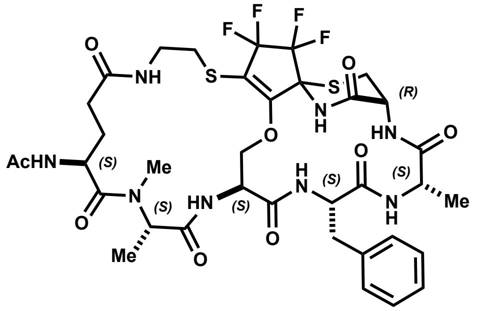

**3o** (Compound 42, *J. Am. Chem. Soc.*, **2023**, *145* (29), 15888-15895.)	Ac-E*VTFAC-NH_2	Cysteamine	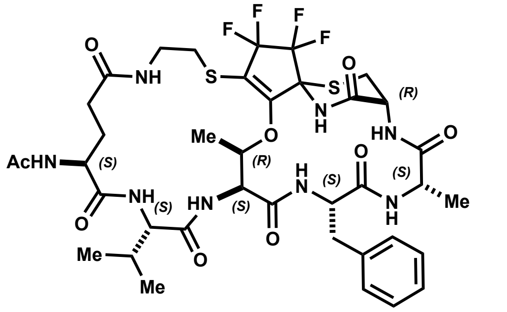
**3p** (Compound 40, *J. Am. Chem. Soc.*, **2023**, *145* (29), 15888-15895.)	Ac-D*GSFAC-NH_2	Cysteamine	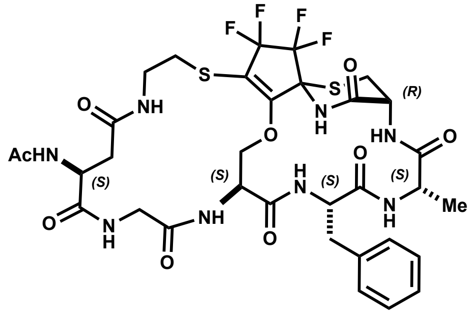
**3q**	Ac-D*GSFAC-NH_2	Ethylenediamine	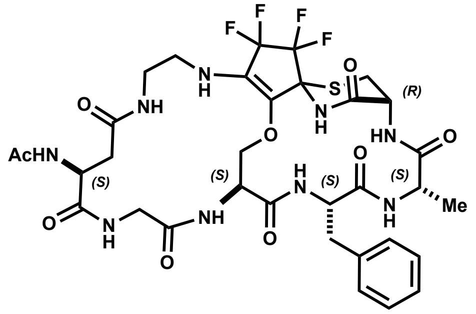
**3r** (Compound S20, *J. Am. Chem. Soc.*, **2023**, *145* (29), 15888-15895.)	Ac-D*ASFAC-NH_2	-	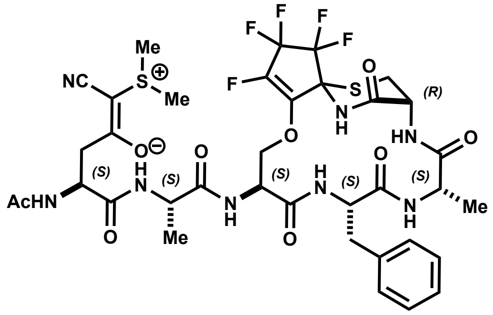

Polycycle 3a.

Synthesized according to **General Procedure C-D** with crude peptide Ac-D*PTFSC-NH_2 (260 mg, 1.0 equiv., 328 μmol) and 2-mercaptoethanol (27.6 μL, 1.2 equiv., 393 μmol). Purification by preparative-reverse phase HPLC (gradient 10% $MeCN/H_2O$ → 70% $MeCN/H_2O$) afforded the free acid intermediate (60.0 mg, 20% isolated yield) as a slightly yellow powder. Then subjection of the free acid intermediate (20.0 mg, 1.0 equiv., 21.7 μmol) to **General Procedure E** followed by purification by preparative-reverse phase HPLC (gradient 35% $MeCN/H_2O$ → 100% $MeCN/H_2O$) afforded the title compound (1.5 mg, 8% isolated yield) as an off-white powder. **HRMS** (QTOF) m/z: $[M+H]^+$ calc'd for $C_{37}H_{44}F_4N_7O_{11}S_2^+$ 902.2471, found 902.2488; 1.9 ppm mass defect.

Polycycle 3c.

Synthesized according to **General Procedure C-D** with crude peptide Ac-D*A(N-Me)TFSC-NH_2 (118.0 mg, 1.0 equiv., 151 μmol) and cysteamine (14.0 mg, 1.2 equiv., 181 μmol). Filtered crude reaction through short celite plug to remove buffer salts to obtain crude free acid intermediate as a slightly yellow powder. Then subjection of the crude free acid intermediate to **General Procedure F** followed by purification by silica gel FCC (10-15% MeOH/DCM) afforded the title compound (3.9 mg, 3% isolated yield) as an off-white powder. **HRMS** (QTOF) m/z: $[M+H]^+$ calc'd for $C_{36}H_{45}F_4N_8O_{10}S_2^+$ 889.2631, found 889.2630; 0.1 ppm mass defect.

Polycycle 3d.

Synthesized according to **General Procedure C-D** with crude peptide Ac-D*P-alloT-FSC-NH_2 (100.0 mg, 1.0 equiv., 126 μmol) and cysteamine (11.7 mg, 1.2 equiv., 151 μmol). Filtered crude reaction through short celite plug to remove buffer salts to obtain crude free acid intermediate as a slightly yellow powder. Then subjection of the crude free acid intermediate to **General Procedure F** followed by purification by silica gel FCC (10-15% MeOH/DCM) afforded the title compound (8.4 mg, 6% isolated yield) as an off-white powder. **HRMS** (QTOF) m/z: $[M+H]^+$ calc'd for $C_{37}H_{45}F_4N_8O_{10}S_2^+$ 901.2631, found 901.2656; 2.8 ppm mass defect.

Polycycle 3e.

Synthesized according to **General Procedure C-D** with crude peptide Ac-D*PTFS-homoC-NH_2 (150.0 mg, 1.0 equiv., 185.9 μmol) and cysteamine (17.2 mg, 1.2 equiv., 223 μmol). Purification by preparative-reverse phase HPLC (gradient 10% MeCN/H_2O → 65% MeCN/H_2O) afforded the free acid intermediate (35.0 mg, 29% isolated yield) as a slightly yellow powder. Then subjection of the free acid intermediate (35.0 mg, 1.0 equiv., 37.5 μmol) to **General Procedure F** followed by purification by preparative-reverse phase HPLC (gradient 40% MeCN/H_2O → 100% MeCN/H_2O) afforded the title compound (4.8 mg, 14% isolated yield) as an off-white powder. **HRMS** (QTOF) m/z: $[M+H]^+$ calc'd for $C_{38}H_{47}F_4N_8O_{10}S_2^+$ 915.2787, found 915.2776; 1.2 ppm mass defect.

Polycycle 3j.

Synthesized according to **General Procedure C-D** with crude peptide Ac-E*PSFTC-NH_2 (250 mg, 1.0 equiv., 310 μmol) and exogenous nucleophile hydroxymethylated thiazole thione (54.7 mg, 1.2 equiv., 372 μmol). Purification by preparative-reverse phase HPLC (gradient 10% MeCN/H_2O → 100% MeCN/H_2O) afforded the free acid intermediate (15.7 mg, 6% isolated yield) as a slightly yellow powder. Then subjection of the free acid intermediate (10.0 mg, 1.0 equiv., 10.0 μmol) to **General Procedure E** followed by purification by preparative-reverse phase HPLC (gradient 35% MeCN/H_2O → 100% MeCN/H_2O) afforded the title compound (1.2 mg, 12% isolated yield) as an off-white powder. **HRMS** (QTOF) m/z: $[M+H]^+$ calc'd for $C_{40}H_{45}F_4N_8O_{11}S_3^+$ 985.2301, found 985.2283; 1.8 ppm mass defect.

Polycycle 3k.

Synthesized according to **General Procedure C-D** with crude peptide Ac-E*A(N-Me)SFTC-NH_2 (100.0 mg, 1.0 equiv., 125.8 μmol) and exogenous nucleophile cysteamine (11.7 mg, 1.2 equiv., 151.0 μmol). Filtered crude reaction through short celite plug to remove buffer salts to obtain crude free acid intermediate as a slightly yellow powder. Then subjection of the crude free acid intermediate to **General Procedure F** followed by purification by preparative-reverse phase HPLC (gradient 35% MeCN/H_2O → 100% MeCN/H_2O) then pTLC (10% MeOH/DCM) afforded the title compound (3.1 mg, 3% isolated yield) as a white film. **HRMS** (QTOF) m/z: $[M+H]^+$ calc'd for $C_{37}H_{47}F_4N_8O_{10}S_2^+$ 903.2787, found 903.2805; 2.0 ppm mass defect.

Polycycle 3l.

Synthesized according to **General Procedure C-D** with crude peptide Ac-E*PSFT-homoC-NH_2 (100.0 mg, 1.0 equiv., 121.8 μmol) and exogenous nucleophile cysteamine (11.3 mg, 1.2 equiv., 146.2 μmol). Purification by preparative-reverse phase HPLC (gradient 10% MeCN/H_2O → 100% MeCN/H_2O) afforded the free acid intermediate (45.9 mg, 40% isolated yield) as a slightly yellow powder. Then subjection of the crude free acid intermediate to **General Procedure F** followed by purification by preparative-reverse phase HPLC (gradient 40% MeCN/H_2O → 100% MeCN/H_2O) then pTLC (10% MeOH/DCM) afforded the title compound (4.4 mg, 11% isolated yield) as an off-white powder. **HRMS** (QTOF) m/z: $[M+H]^+$ calc'd for $C_{39}H_{49}F_4N_8O_{10}S_2^+$ 929.2944, found 929.2901; 4.6 ppm mass defect.

Polycycle 3q.

Synthesized according to **General Procedure C-D** with analytically pure **3r** (17.9 mg, 1.0 equiv., 20.5 μmol) and exogenous nucleophile ethylenediamine (1.8 mg, 1.5 equiv., 30.7 μmol). Purification by preparative-reverse phase HPLC (gradient 10% MeCN/H_2O → 65% MeCN/H_2O) afforded the free acid intermediate (15.2 mg, 79% isolated yield) as a slightly yellow powder. Then subjection of the free acid intermediate (15.2 mg, 1.0 equiv., 16.1 μmol) to **General Procedure F** followed by purification by silica gel FCC (5-10% MeOH/$CHCl_3$) afforded the title compound (10.1 mg, 77% isolated yield) as an off-white powder. **HRMS** (QTOF) m/z: $[M+H]^+$ calc'd for $C_{33}H_{40}F_4N_9O_9S^+$ 814.2600, found 814.2593; 0.9 ppm mass defect.

Polycycle S1 Analogs

Compound	Linear Precursor	Nucleophile	Structure
**S1**<br>(Compound 43, *J. Am. Chem. Soc.*, **2023**, *145* (29), 15888-15895.)	Ac-D*PNHFTC-NH_2	Cysteamine	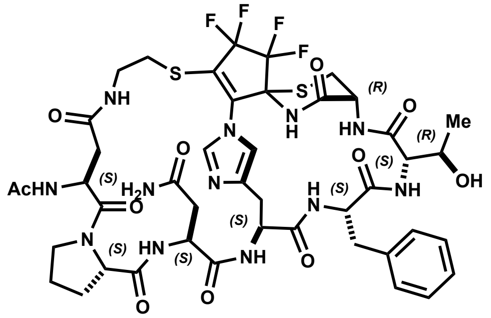
**S1a**<br>(*J. Am. Chem. Soc.*, **2023**, *145* (29), 15888-15895.)	Ac-D*PNHFTC-NH_2	Cysteamine	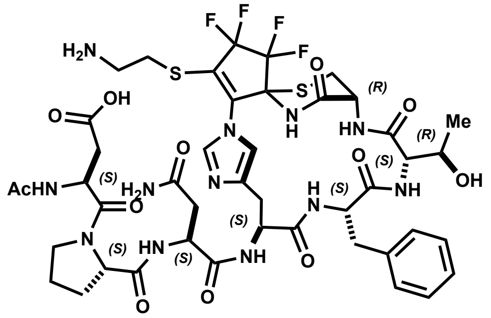
**S1b**<br>(Compound 48, *J. Am. Chem. Soc.*, **2023**, *145* (29), 15888-15895.)	Ac-D*PNHFTC-NH_2	2-Mercaptoethanol	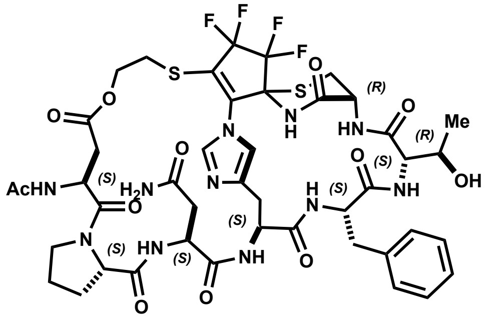
**S1c**<br>(*J. Am. Chem. Soc.*, **2023**, *145* (29), 15888-15895.)	Ac-D*PNHFTC-NH_2	2-Mercaptoethanol	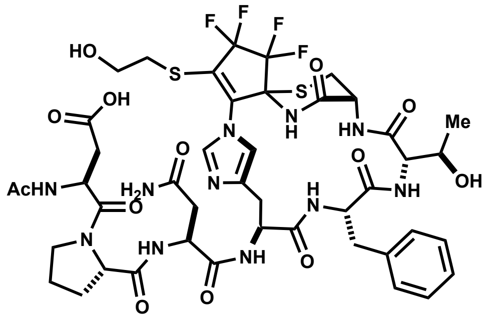

Polycycle S2 Analogs

Compound	Linear Precursor	Nucleophile	Structure
**S2** (Compound 17, *J. Am. Chem. Soc.*, **2023**, *145* (29), 15888-15895.)	Ac-CWSC-NH_2	-	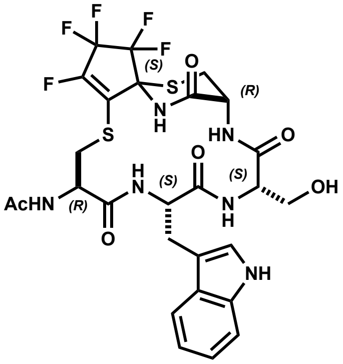
**S2a** (Compound 26, *J. Am. Chem. Soc.*, **2023**, *145* (29), 15888-15895.)	Ac-CWS-homoC-NH_2	-	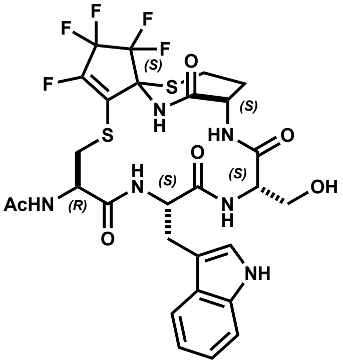
**S2b**	Ac-CWSC-NH_2	Oleylamine	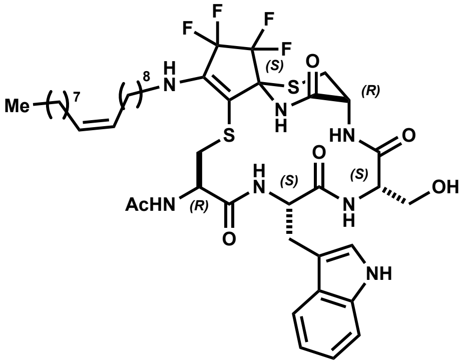
**S2c**	Ac-CWSC-NH_2	Hexylamine	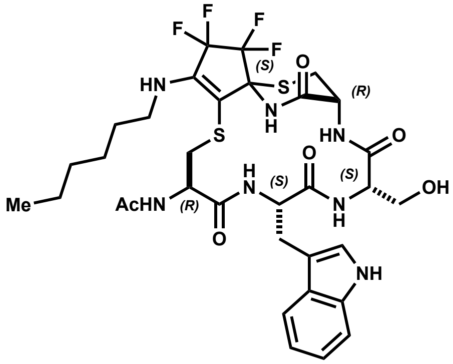

**S2d**	Ac-CWSC-NH_2	Propylamine	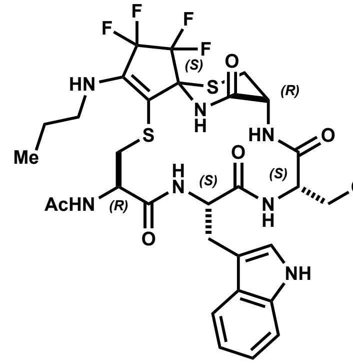
**S2e**	Ac-CWSC-NH_2	2-mercaptopyrimidine	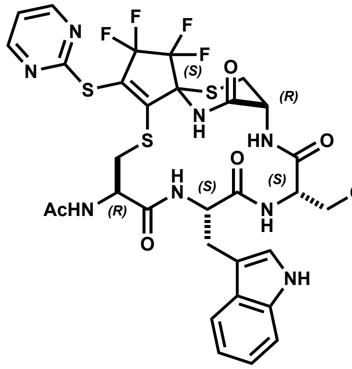
**S2f**<br>(Compound 20, *J. Am. Chem. Soc.*, **2023**, *145* (29), 15888-15895.)	Ac-CWSC-NH_2	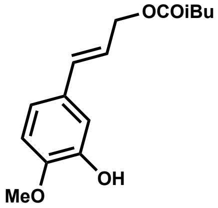<br>(*J. Am. Chem. Soc.*, **2023**, *145* (29), 15888-15895.)	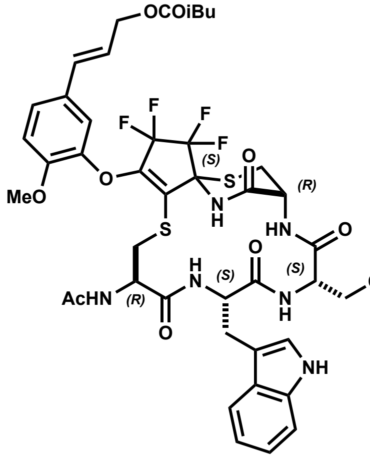
**S2g**<br>(Compound 19, *J. Am. Chem. Soc.*, **2023**, *145* (29), 15888-15895.)	Ac-CWSC-NH_2	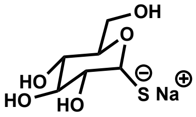<br>(AA Blocks)	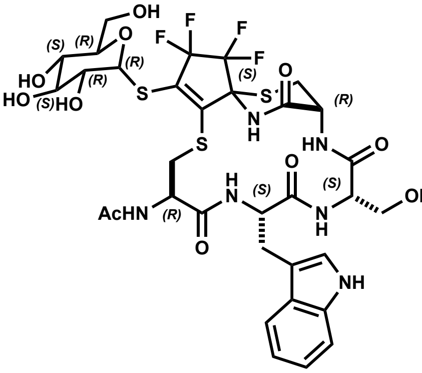

Polycycle S2b.

Synthesized according to **General Procedure C** with analytically pure **S2** (5.0 mg, 1.0 equiv., 7.2 µmol) and exogenous nucleophile oleylamine (3.6 µL, 1.5 equiv., 11.0 µmol). Purification by pTLC (5% MeOH/DCM) afforded the title compound (4.7 mg, 69% isolated yield) as a white film. **HRMS** (QTOF) m/z: $[M+H]^+$ calc'd for $C_{45}H_{64}F_4N_7O_6S_2^+$ 938.4290, found 938.4298; 0.9 ppm mass defect.

Polycycle S2c.

Synthesized according to **General Procedure C** with analytically pure **S2** (5.0 mg, 1.0 equiv., 7.2 µmol) and exogenous nucleophile propylamine (2.0 µL, 3.4 equiv., 25.0 µmol). Purification by pTLC (5% MeOH/DCM) afforded the title compound (5.2 mg, 98% isolated yield) as a white film. **HRMS** (QTOF) m/z: $[M+H]^+$ calc'd for $C_{33}H_{42}F_4N_7O_6S_2^+$ 772.2569, found 772.2565; 0.5 ppm mass defect.

Polycycle S2d.

Synthesized according to **General Procedure C** with analytically pure **S2** (5.0 mg, 1.0 equiv., 7.2 µmol) and exogenous nucleophile hexylamine (2.0 µL, 2.1 equiv., 15.0 µmol). Purification by pTLC (7% MeOH/DCM) afforded the title compound (5.0 mg, 89% isolated yield) as a white film. **HRMS** (QTOF) m/z: $[M+H]^+$ calc'd for $C_{30}H_{36}F_4N_7O_6S_2^+$ 730.2099, found 730.2124; 3.4 ppm mass defect.

Polycycle S2e.

Synthesized according to **General Procedure C** with analytically pure **S2** (5.0 mg, 1.0 equiv., 7.2 µmol) and exogenous nucleophile 2-mercaptopyrimidine (1.2 mg, 1.5 equiv., 11.0 µmol). Purification by pTLC (10% MeOH/DCM) afforded the title compound (5.6 mg, 98% isolated yield) as a white powder. **HRMS** (QTOF) m/z: $[M+H]^+$ calc'd for $C_{31}H_{32}F_4N_9O_6S_2^+$ 783.1459, found 783.1477; 2.3 ppm mass defect.

Polycycle S3 Analogs

Compound	Linear Precursor	Nucleophile	Structure
**S3** (Compound 24, *J. Am. Chem. Soc.*, **2023**, *145* (29), 15888-15895.)	Ac-CSC-NH_2	-	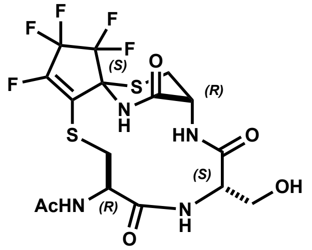
**S3a**	Ac-CSC-NH_2	Oleylamine	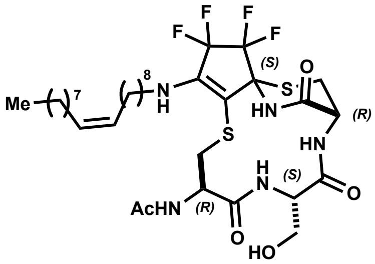
**S3b**	Ac-CSC-NH_2	N-methylbenzylamine	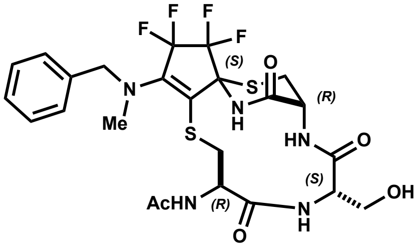
**S3c**	Ac-CSC-NH_2	2-mercaptopyridine	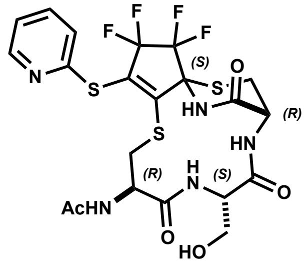

Polycycle S3a.

Synthesized according to **General Procedure C** with analytically pure **S3** (5.0 mg, 1.0 equiv., 9.9 µmol) and exogenous nucleophile oleylamine (4.9 µL, 1.5 equiv., 15.0 µmol). Purification by pTLC (5% MeOH/DCM) afforded the title compound (4.3 mg, 58% isolated yield) as a white film. **HRMS** (QTOF) m/z: $[M+H]^+$ calc'd for $C_{34}H_{54}F_4N_5O_5S_2^+$ 752.3497, found 752.3502; 0.7 ppm mass defect.

Polycycle S3b.

Synthesized according to **General Procedure C** with analytically pure **S3** (5.0 mg, 1.0 equiv., 9.9 µmol) and exogenous nucleophile N-methylbenzylamine (2.0 µL, 1.5 equiv., 15.0 µmol). Purification by pTLC (10% MeOH/DCM) afforded the title compound (1.7 mg, 28% isolated yield) as a white powder. **HRMS** (QTOF) m/z: $[M+H]^+$ calc'd for $C_{24}H_{28}F_4N_5O_5S_2^+$ 606.1463, found 606.1490; 4.5 ppm mass defect.

Polycycle S3c.

Synthesized according to **General Procedure C** with analytically pure **S3** (5.0 mg, 1.0 equiv., 9.9 µmol) and exogenous nucleophile 2-mercaptopyridine (1.7 mg, 1.5 equiv., 15.0 µmol). Purification by pTLC (10% MeOH/DCM) afforded the title compound (1.6 mg, 27% isolated yield) as a white powder. **HRMS** (QTOF) m/z: $[M+H]^+$ calc'd for $C_{21}H_{22}F_4N_5O_5S_3^+$ 596.0714, found 596.0718; 0.7ppm mass defect.

Polycycle S4 Analogs

Compound	Linear Precursor	Nucleophile	Structure
**S4** (Compound 32, *J. Am. Chem. Soc.*, **2023**, *145* (29), 15888-15895.)	Ac-YCAC-NH_2	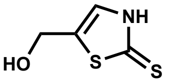 (*J. Am. Chem. Soc.*, **2023**, *145* (29), 15888-15895.)	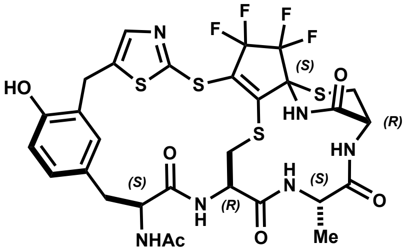
**S4a** (Compound S22, *J. Am. Chem. Soc.*, **2023**, *145* (29), 15888-15895.)	Ac-YCAC-NH_2	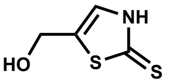 (*J. Am. Chem. Soc.*, **2023**, *145* (29), 15888-15895.)	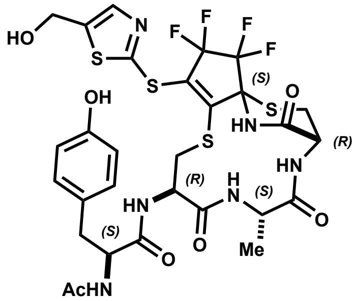
**S4b** (Compound 31, *J. Am. Chem. Soc.*, **2023**, *145* (29), 15888-15895.)	Ac-YACFAC-NH_2	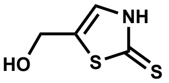 (*J. Am. Chem. Soc.*, **2023**, *145* (29), 15888-15895.)	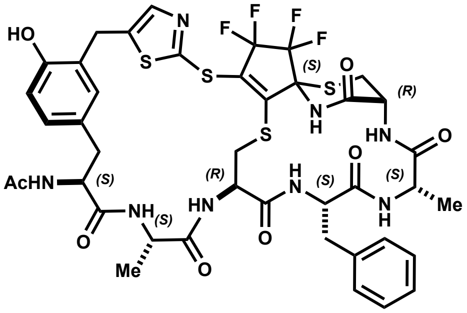
**S4c** (Compound 30, *J. Am. Chem. Soc.*, **2023**, *145* (29), 15888-15895.)	Ac-YACFAC-NH_2	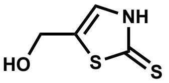 (*J. Am. Chem. Soc.*, **2023**, *145* (29), 15888-15895.)	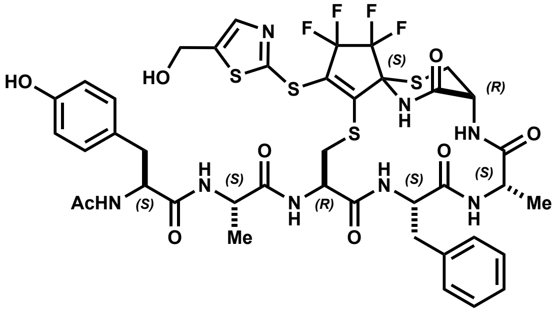

Polycycle S5 Analogs

Compound	Linear Precursor	Structure
**S5**<br>(Compound 50, *J. Am. Chem. Soc.*, **2023**, *145* (29), 15888-15895.)	Dat-PCVAC-NHMe	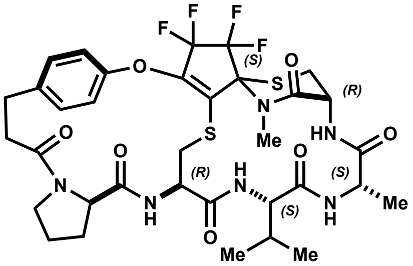
**S5a**	Dat-PCVAC-NH_2	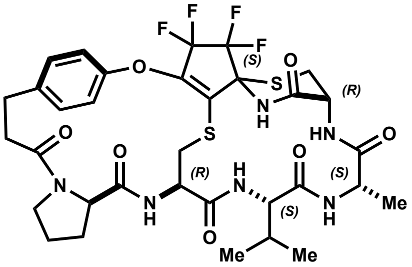

Polycycle S5a.

Synthesized according to **General Procedure B** with crude peptide Dat-PCVAC-NH_2 (50.0 mg, 1.0 equiv., 78.3 μmol). Purification by preparative-reverse phase HPLC (gradient 10% $MeCN/H_2O$ → 60% $MeCN/H_2O$) afforded the title compound (14.5 mg, 24% isolated yield) as an off-white powder. **HRMS** (QTOF) m/z: $[M+H]^+$ calc'd for $C_{33}H_{39}F_4N_6O_7S_2^+$ 771.2252, found 771.2264; 1.6 ppm mass defect.

Polycycle S6 Analogs

Compound	Linear Precursor	Structure
**S6**<br>(Compound 11, *J. Am. Chem. Soc.*, **2023**, *145* (29), 15888-15895.)	Ac-HFSCAC-NH_2	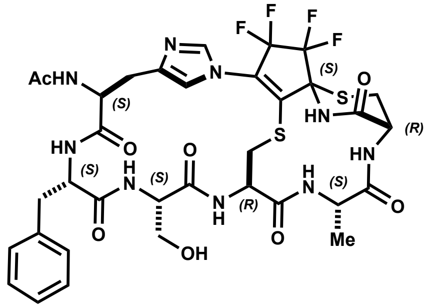
**S6a**<br>(Compound 10*J. Am. Chem. Soc.*, **2023**, *145* (29), 15888-15895.)	Ac-HFCASC-NH_2	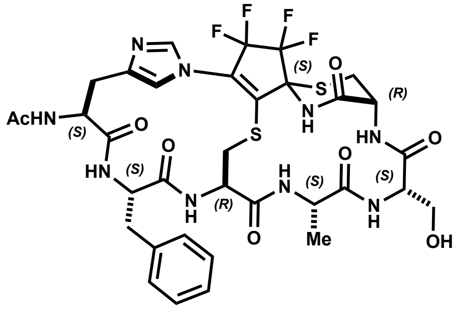

Polycycle S7 Analogs

Compound	Linear Precursor	Nucleophile	Structure
**S7**<br>(Compound S3, *J. Am. Chem. Soc.*, **2023**, *145* (29), 15888-15895.)	Ac-CTFC-NHMe	Phenol	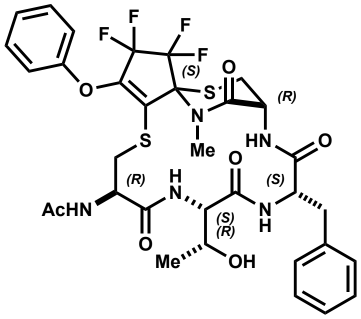
**S7a**<br>(Compound 9, *J. Am. Chem. Soc.*, **2023**, *145* (29), 15888-15895.)	Ac-YNCTFC-NH_2	-	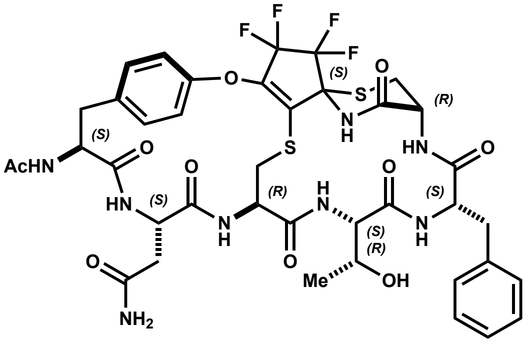
**S7b**<br>(Compound S2, *J. Am. Chem. Soc.*, **2023**, *145* (29), 15888-15895.)	Ac-CTFC-NH_2	Imidazole	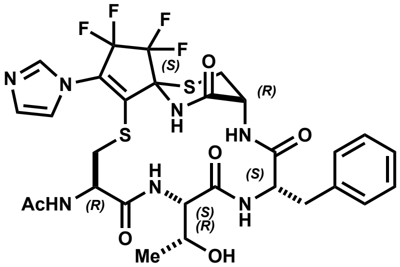

Computational Methods

MD simulations

All MD simulations for compounds **1**–**S7b** were performed using the Amber24 package[5] with the General Amber Force Field (GAFF2[6]). Initial structures were generated as the lowest energy conformers from ETKDGv3 calculations[7] using RDKit[8]. Atomic charges were assigned via the AM1-BCC method as implemented in Antechamber.[9] Using the *tleap* module, macrocycles were solvated in either a TIP3P water box or a chloroform box (CHCL3BOX), maintaining a minimum buffer distance of 20 Å between the solute and the box edges. The entire solvated system underwent energy minimization consisting of 10000 steps of steepest descent followed by 10000 steps of conjugate gradient algorithms. Subsequently, the system was heated from 0 to 300 K at constant volume for 200 ps. Following thermalization, a 1 ns equilibration was performed at constant temperature (300 K) and pressure (1 bar) using the Berendsen coupling algorithm.[10] Finally, production runs were executed for a total of 1000 ns (two independent 500 ns replicas) at 700 K using the leapfrog "middle" scheme (Langevin thermostat with friction coefficient = 5 ps^{-1}).[11] The SHAKE algorithm was applied to constrain all bonds involving hydrogen atoms.[12] Trajectories were sampled every 50 ps, after discarding the first 100 ns of each replica, a total of 16000 snapshots per compound per solvent were retained for analysis.

Clustering analysis

For each compound of high-T MD simulations, we did a cluster analysis of 16000 structures from the 1 μs trajectory. The density peaks advanced[13] (DPA) algorithm was used to cluster all generated conformations. To recover the conformational distributions from 700 K to 300 K, we calculated the density of each conformation by using the point-adaptive k-nearest neighbors (PAk) estimator.[14] Then, the Boltzmann weight was assigned to each conformation according to our PDR approach. Finally, to obtain the probability of clusters, we grouped all conformations by DPA and summed the weights within the same cluster. The probability of a cluster is directly proportional to the sum of the weights of all conformations. The clusters were sorted based on their probabilities after reweighting. The conformation with the highest density in each cluster was identified as the cluster center. Notably, we used the Cartesian coordinates of backbone atoms to estimate the density of each conformation (**Fig. S8**). Python scripts for clustering can be obtained from our previous work.[15]

Calculation of conformational transition penalty

To quantify the energetic cost of the transition from the aqueous "open" state to the membrane "close" state, we calculated the conformational transition penalty ΔG_{conf}. The parameters P_{open} and P_{close} were defined based on the reweighted populations derived from the clustering analysis:

(1) The central conformation of the most populated cluster from the aqueous simulation was defined as the representative "open" state. Its corresponding reweighted population occupancy was assigned as P_{open}.
(2) The center of the most populated cluster from the chloroform simulation was defined as the reference "close" state. To determine the population of this state in water (P_{close}), we first screened the top-ranked clusters from the aqueous simulation. If a cluster center was found within a backbone RMSD of 1.5 Å of the reference "close" conformation, its population was assigned as P_{close}.
(3) If no such cluster was identified among the top aqueous clusters, we searched the entire aqueous trajectory for snapshots within 1.5 Å backbone RMSD of the reference chloroform "close" conformation. P_{close} was then calculated by summing the Boltzmann weights of these specific snapshots.

(4) For compounds (**1o**, **3m**, **3p**, and **S6**) where the "close" form was not sampled in the aqueous simulation, but the "open" conformation was successfully captured in the chloroform simulation, we utilized the conformational ensemble from the chloroform trajectory to estimate the ratio. In these specific cases, the free energy penalty was approximated using the populations of the open and close states within the chloroform ensemble.

Hydrogen bonding analysis

Intramolecular hydrogen bonds (IMHBs) analysis was performed using the *hbond* command within the CPPTRAJ module of AmberTools24.[16] A hydrogen bond was defined by a donor-acceptor distance cutoff of 3.2 Å and a donor-hydrogen-acceptor angle cutoff of 120°. The occupancy of each IMHB was calculated as the percentage of frames in which these criteria were met over the final 800 ns of the simulation trajectories. Only hydrogen bonds with an occupancy greater than 1% were considered significant for further analysis.

Surface area analysis

Solvent accessible surface area (SASA) and polar surface area (PSA) were computed using the *surf* command within the CPPTRAJ module. A standard solvent probe radius of 1.4 Å was applied for all calculations. SASA was defined as the total surface area derived from all constituent atoms. Standard PSA calculations were restricted to contributions from **N** and **O** atoms. Notably, for the bridging Cys series and analogs bearing sulfur-containing N-terminal modifications (e.g., **S1**, **S4**), the surface area contributions of **S** atoms were explicitly included in the PSA definition to account for their polarity.

Parallel artificial membrane permeability assay (PAMPA)

This assay was performed as outlined in the protocol of the parallel artificial permeability assay kit (BioAssay Systems Cat. # PAMPA-096). Incubation was performed at room temperature for 18 hours. Data analysis was performed using a Tecan M1000 plate reader and 96-well UV plates purchased from BioAssay Systems (Cat. # P96 UV). UV absorbance was measured from 230 to 500 nm in 10 nm intervals to determine peak absorbance of test compounds and controls. The following equation used to determined Permeability Rate (P_e):

$$P_e = C \times -ln\left(1 - \frac{OD_A}{OD_E}\right)\ cm/s$$

Where OD_A is the absorbance of Acceptor Solution minus Blank, OD_E is the absorbance of Equilibrium Standard minus Blank, and, using an 18-hour incubation, C = 7.72 x 10^{-6}. When a different length incubation time was used, the following equation was used to determine C:

$$C = \frac{V_D \times V_A}{(V_D + V_A) \times Area \times time}\ cm/s$$

Where V_D is the donor volume (0.2 cm^3), V_A is the acceptor volume (0.3 cm^3), Area is the membrane area (0.24 cm^2).

A total of seven PAMPA assays were run, each in respective technical replicates. Permeability data was z-score filtered, where values outside of one standard deviation from the average were excluded to remove outliers. Z-scores were determined using the following equation:

$$z = \frac{x - \mu}{\sigma}$$

Where x is the data point, μ is the average, and σ is the standard deviation.

Table S2. Averaged and z-score filtered permeability data from seven independent PAMPA experiments.

Compound Number	Avg. Permeability Rate (x10^6 cm/s)	Standard Deviation (x10^6 cm/s)
**Theophylline**	0.59	0.081
**Diclofenac**	3.66	0.416
**Chloramphenicol**	4.66	0.856
**1**	1.33	0.697
**1a**	0.81	0.363
**1b**	7.65	1.211
**1c**	6.06	0.874
**1d**	5.03	0.628
**1e**	4.25	0.423
**1f**	4.38	0.821
**1g**	1.93	0.061
**1h**	3.97	0.669
**1i**	1.22	0.613
**1j**	6.11	1.529
**1k**	5.87	1.328
**1l**	0.60	0.020
**1m**	1.18	0.037
**1n**	1.09	0.034
**1o**	1.77	0.037

**1p**	0.30	0.373
**1q**	0.11	0.023
**1r**	4.82	1.157
**1s**	0.20	0.028
**1t**	0.60	0.418
**1u**	0.42	0.222
**2**	2.31	1.465
**2a**	3.23	2.189
**2b**	4.28	0.981
**S1**	0.99	0.154
**S1a**	0.31	0.150
**S1b**	0.38	0.163
**S1c**	0.31	0.147
**3**	1.91	0.721
**3a**	0.81	0.243
**3b**	0.19	0.091
**3c**	0.92	0.086
**3d**	1.80	0.317
**3e**	1.11	0.020
**3f**	1.59	0.327
**3g**	0.56	0.039
**3h**	0.53	0.134
**3i**	0.40	0.004
**3j**	1.96	0.047
**3k**	1.38	0.081
**3l**	2.80	0.123
**3m**	0.76	0.801
**3n**	1.29	0.187
**3o**	0.23	0.056
**3p**	0.51	0.247
**3q**	1.05	0.131
**3r**	0.19	0.049
**S2**	0.33	0.123
**S2a**	0.45	0.134
**S2b**	1.82	1.050
**S2c**	0.58	0.555
**S2d**	0.57	0.246
**S2e**	0.38	0.019
**S2f**	0.86	0.283
**S2g**	0.15	0.030
**S3**	1.44	0.500
**S3a**	1.45	0.560
**S3b**	0.14	0.026
**S3c**	1.08	0.784
**S4**	1.76	0.507
**S4a**	0.29	0.043
**S4b**	0.60	0.186
**S4c**	0.34	0.050
**S5**	2.40	0.600
**S5a**	1.11	0.008
**S6**	0.47	0.019
**S6a**	0.56	0.219
**S7**	1.31	0.085

**S7a**	0.81	0.037
**S7b**	0.90	0.581

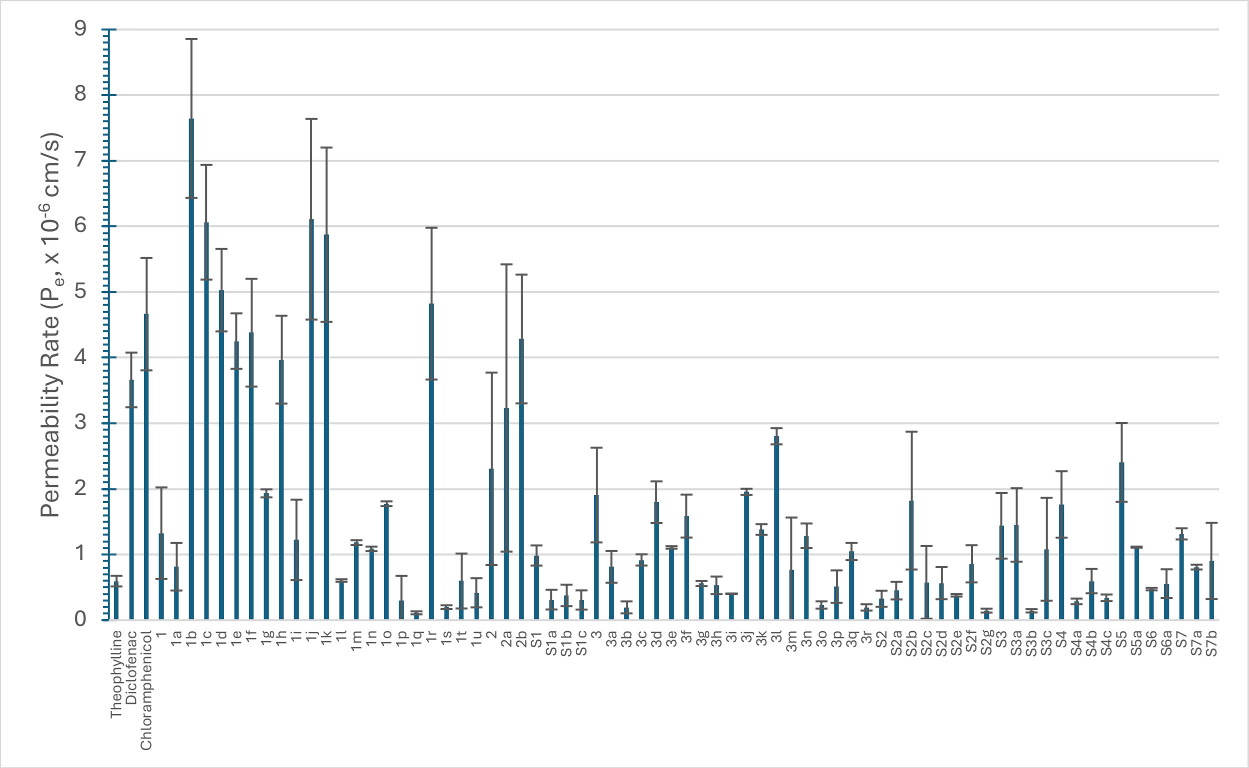

Figure S12. Bar graph with averaged and z-score filtered permeability data from seven independent PAMPA experiments.

Table S3. Z-score filtered full PAMPA dataset from seven independent experiments.

Compound Number	JACS ' 23 DATA				PAMPA 3 DATA		PAMPA 4 DATA		PAMPA 5 DATA		PAMPA 6 DATA		PAMPA 7 DATA		Avg. Permeability Rate (x106cm/s)	Standard Deviation (x10^{6}cm/s)
	Assay 1 Well 1	Assay 1 Well 2	Assay 2 Well 1	Assay 2 Well 2	Well 1	Well 2	Well 1	Well 2	Well 1	Well 2	Well 1	Well 2	Well 1	Well 2		
**Theophylline**	5.50506E-07	4.83665E-07	5.966E-07	6.18784E-07	-	-	7.35795E-07	-	7.28546E-07	6.31331E-07	5.19384E-07	5.4789E-07	5.36589E-07	5.91455E-07	0.59	0.08
**Diclofenac**	3.42111E-06	3.00558E-06	4.0553E-06	-	-	-	4.12122E-06	3.78044E-06	3.63919E-06	4.00387E-06	3.94708E-06	4.0089E-06	3.11836E-06	3.16371E-06	3.66	0.42
**Chloramphenicol**	4.4116E-06	3.77755E-06	-	6.06656E-06	-	-	5.4364E-06	3.2316E-06	4.22661E-06	4.70024E-06	5.0842E-06	5.75794E-06	4.16034E-06	4.44309E-06	4.66	0.86
**1**	-	-	7.24281E-07	5.36741E-07	-	-	2.50511E-06	1.20556E-06	1.48841E-06	1.46622E-06	-	-	2.0347E-06	6.46281E-07	1.33	0.70
**1a**	1.19846E-06	1.02366E-06	4.28999E-07	3.74052E-07	-	-	1.07271E-06	3.85011E-07	7.31862E-07	7.88727E-07	-	-	-	1.32536E-06	0.81	0.36
**1b**	-	-	-	-	-	-	-	-	8.81656E-06	-	-	-	6.39851E-06	7.72173E-06	7.65	1.21
**1c**	-	-	-	-	-	-	-	-	-	-	-	-	6.68137E-06	5.4452E-06	6.06	0.87
**1d**	-	-	-	-	-	-	-	-	4.58463E-06	-	5.47319E-06	-	-	-	5.03	0.63
**1e**	-	-	-	-	-	-	-	-	-	-	-	-	3.95354E-06	4.55136E-06	4.25	0.42
**1f**	-	-	-	-	-	-	-	-	4.39741E-06	5.19275E-06	-	-	-	3.55044E-06	4.38	0.82
**1g**	-	-	-	-	-	-	-	-	-	-	-	-	1.97533E-06	1.88923E-06	1.93	0.06
**1h**	-	-	-	-	-	-	-	-	-	-	3.49592E-06	4.44227E-06	-	-	3.97	0.67
**1i**	-	-	-	-	-	-	-	-	-	-	-	-	1.65622E-06	7.89928E-07	1.22	0.61
**1j**	-	-	-	-	-	-	-	-	-	-	7.19042E-06	5.02783E-06	-	-	6.11	1.53
**1k**	-	-	-	-	-	-	-	-	-	-	6.8272E-06	6.43657E-06	-	4.35676E-06	5.87	1.33
**1l**	-	-	-	-	-	-	-	-	-	-	6.18121E-07	5.90382E-07	-	-	0.60	0.02
**1m**	-	-	-	-	-	-	-	-	-	-	1.20745E-06	1.15516E-06	-	-	1.18	0.04
**1n**	-	-	-	-	-	-	-	-	-	-	1.06211E-06	1.11019E-06	-	-	1.09	0.03
**1o**	-	-	-	-	-	-	-	-	-	-	1.79999E-06	1.74835E-06	-	-	1.77	0.04
**1p**	-	-	-	-	-	-	-	-	-	-	7.32569E-07	-	8.98038E-08	8.24301E-08	0.30	0.37
**1q**	-	-	1.28136E-07	9.59019E-08	-	-	-	-	-	-	-	-	-	-	0.11	0.02
**1r**	-	-	-	-	-	-	-	-	-	-	4.00512E-06	5.64072E-06	-	-	4.82	1.16
**1s**	-	-	-	-	-	-	-	-	-	-	-	-	2.1872E-07	1.7865E-07	0.20	0.03
**1t**	-	-	-	-	-	-	-	-	-	-	-	-	3.00308E-07	8.9203E-07	0.60	0.42
**1u**	-	-	-	-	-	-	-	-	-	-	-	-	2.60356E-07	5.74564E-07	0.42	0.22
**2**	-	-	1.84053E-06	5.12063E-06	2.59998E-06	1.77893E-06	-	-	-	-	1.23748E-06	1.26055E-06	-	-	2.31	1.47
**2a**	-	-	-	-	-	-	-	-	5.08513E-06	5.17126E-06	1.36221E-06	1.31292E-06	-	-	3.23	2.19
**2b**	-	-	-	-	-	-	-	-	4.9779E-06	3.59108E-06	-	-	-	-	4.28	0.98
**S1**	-	-	1.07582E-06	-	9.28609E-07	-	1.13975E-06	7.96582E-07	-	-	-	-	-	-	0.99	0.15
**S1a**	-	-	4.18909E-07	-	-	2.06798E-07	-	-	-	-	-	-	-	-	0.31	0.15
**S1b**	-	-	2.61088E-07	4.92127E-07	-	-	-	-	-	-	-	-	-	-	0.38	0.16
**S1c**	-	-	-	3.87156E-07	3.97183E-07	1.37861E-07	-	-	-	-	-	-	-	-	0.31	0.15
**3**	-	-	-	-	1.94015E-06	1.07182E-06	3.04094E-06	-	1.88317E-06	1.59524E-06	-	-	-	-	1.91	0.72
**3a**	-	-	-	-	-	-	-	-	9.83754E-07	6.40683E-07	-	-	-	-	0.81	0.24
**3b**	-	-	2.05331E-07	1.43156E-07	2.44582E-07	9.6905E-08	3.46592E-07	1.32943E-07	-	-	-	-	-	-	0.19	0.09
**3c**	-	-	-	-	-	-	-	-	8.57232E-07	9.78647E-07	-	-	-	-	0.92	0.09
**3d**	-	-	-	-	-	-	-	-	1.57352E-06	2.02226E-06	-	-	-	-	1.80	0.32
**3e**	-	-	-	-	-	-	-	-	1.09453E-06	1.12212E-06	-	-	-	-	1.11	0.02
**3f**	-	-	-	-	-	-	-	-	1.81675E-06	1.35413E-06	-	-	-	-	1.59	0.33
**3g**	-	-	5.14857E-07	5.91294E-07	5.693E-07	-	-	-	-	-	-	-	-	-	0.56	0.04
**3h**	-	-	5.03657E-07	3.74629E-07	5.23123E-07	-	-	5.06411E-07	7.44553E-07	-	-	-	-	-	0.53	0.13
**3i**	-	-	4.05224E-07	-	-	3.98908E-07	-	-	-	-	-	-	-	-	0.40	0.00
**3j**	-	-	-	-	-	-	-	-	1.92231E-06	1.98838E-06	-	-	-	-	1.96	0.05
**3k**	-	-	-	-	1.28966E-06	-	-	-	1.41018E-06	1.44438E-06	-	-	-	-	1.38	0.08
**3l**	-	-	-	-	-	-	-	-	2.88956E-06	2.71518E-06	-	-	-	-	2.80	0.12
**3m**	-	-	1.94224E-07	1.61512E-07	1.76971E-07	-	1.61419E-06	-	-	1.66527E-06	-	-	-	-	0.76	0.80
**3n**	-	-	1.07896E-06	1.34068E-06	-	-	1.44044E-06	-	-	-	-	-	-	-	1.29	0.19
**3o**	-	-	1.71775E-07	1.91099E-07	2.18505E-07	-	2.66651E-07	-	-	3.07926E-07	-	-	-	-	0.23	0.06
**3p**	7.72644E-07	6.71728E-07	3.16438E-07	2.84204E-07	-	-	-	-	-	-	-	-	-	-	0.51	0.25
**3q**	-	-	-	-	-	-	9.7094E-07	9.7094E-07	-	1.19753E-06	-	-	-	-	1.05	0.13
**3r**	2.50116E-07	1.70253E-07	-	1.6248E-07	-	-	-	-	-	-	-	-	-	-	0.19	0.05
**S2**	-	4.12442E-07	-	2.39193E-07	-	-	-	-	-	-	-	-	-	-	0.33	0.12
**S2a**	-	5.67439E-07	4.78383E-07	3.04109E-07	-	-	-	-	-	-	-	-	-	-	0.45	0.13
**S2b**	-	-	-	-	-	-	2.56407E-06	1.07896E-06	-	-	-	-	-	-	1.82	1.05
**S2c**	-	-	-	-	-	-	9.68688E-07	1.84257E-07	-	-	-	-	-	-	0.58	0.55
**S2d**	-	-	-	-	-	-	7.38731E-07	3.91351E-07	-	-	-	-	-	-	0.57	0.25
**S2e**	-	-	-	-	-	-	3.91962E-07	3.65203E-07	-	-	-	-	-	-	0.38	0.02
**S2f**	6.58694E-07	-	1.05919E-06	-	-	-	-	-	-	-	-	-	-	-	0.86	0.28
**S2g**	-	-	1.66028E-07	1.24185E-07	-	-	-	-	-	-	-	-	-	-	0.15	0.03
**S3**	1.21507E-06	1.12784E-06	9.09285E-07	-	-	-	2.05123E-06	1.8852E-06	-	-	-	-	-	-	1.44	0.50
**S3a**	-	-	-	-	-	-	1.84685E-06	1.05436E-06	-	-	-	-	-	-	1.45	0.56
**S3b**	-	-	-	-	-	-	1.61026E-07	1.24951E-07	-	-	-	-	-	-	0.14	0.03
**S3c**	-	-	-	-	-	-	5.25373E-07	1.63429E-06	-	-	-	-	-	-	1.08	0.78
**S4**	-	-	-	-	-	-	2.22091E-06	-	1.84991E-06	1.2187E-06	-	-	-	-	1.76	0.51
**S4a**	-	-	2.55718E-07	3.15952E-07	-	-	-	-	-	-	-	-	-	-	0.29	0.04
**S4b**	-	-	7.63816E-07	3.43168E-07	7.49193E-07	3.18865E-07	-	6.71728E-07	6.74863E-07	6.47678E-07	-	-	-	-	0.60	0.19
**S4c**	-	-	3.5365E-07	3.93963E-07	-	-	3.42406E-07	2.72699E-07	-	-	-	-	-	-	0.34	0.05
**S5**	3.21103E-06	2.66217E-06	2.36361E-06	2.71726E-06	-	-	1.94015E-06	1.52721E-06	-	-	-	-	-	-	2.40	0.60
**S5a**	-	-	-	-	-	-	-	-	1.10571E-06	1.11769E-06	-	-	-	-	1.11	0.01
**S6**	4.60269E-07	4.86662E-07	-	-	-	-	-	-	-	-	-	-	-	-	0.47	0.02
**S6a**	-	7.11556E-07	4.01875E-07	-	-	-	-	-	-	-	-	-	-	-	0.56	0.22
**S7**	1.37509E-06	-	-	1.25465E-06	-	-	-	-	-	-	-	-	-	-	1.31	0.09
**S7a**	8.3408E-07	7.82441E-07	-	-	-	-	-	-	-	-	-	-	-	-	0.81	0.04
**S7b**	6.01863E-07	5.32625E-07	1.57178E-06	-	-	-	-	-	-	-	-	-	-	-	0.90	0.58

Table S4. Full PAMPA dataset from seven independent experiments and Z-score calculations.

Compound Number	JACS '23 DATA				PAMPA 3		PAMPA 4		PAMPA 5		PAMPA 6		PAMPA 7		Avg. Permeability Rate (x106cm/s)	Standard Deviation ($x10^6$cm/s)
	Well 1	Well 2	Well 1	Well 2	Well 1	Well 2	Well 1	Well 2	Well 1	Well 2	Well 1	Well 2	Well 1	Well 2		
Theophylline	5.505E-07	4.837E-07	5.966E-07	6.188E-07	3.389E-07	2.344E-07	7.358E-07	1.056E-06	7.285E-07	6.313E-07	5.194E-07	5.479E-07	5.366E-07	5.915E-07	0.58	0.19
Diclofenac	3.421E-06	3.006E-06	4.055E-06	5.298E-06	1.437E-06	1.370E-06	4.121E-06	3.780E-06	3.639E-06	4.004E-06	3.947E-06	4.009E-06	3.118E-06	3.164E-06	3.45	1.04
Chloramphenicol	4.412E-06	3.778E-06	9.606E-06	6.067E-06	1.982E-06	1.974E-06	5.436E-06	3.232E-06	4.227E-06	4.700E-06	5.084E-06	5.758E-06	4.160E-06	4.443E-06	4.63	1.88
1	6.709E-06	6.137E-06	7.243E-07	5.367E-07	-	-	2.505E-06	1.206E-06	1.488E-06	1.466E-06	-	-	2.035E-06	6.463E-07	2.35	2.24
1a	1.198E-06	1.024E-06	4.290E-07	3.741E-07	-	-	1.073E-06	3.850E-07	7.319E-07	7.887E-07	-	-	9.943E-06	1.325E-06	1.73	2.91
1b	-	-	-	-	-	-	-	-	8.817E-06	9.977E-06	2.439E-06	2.984E-06	6.399E-06	7.722E-06	6.39	3.09
1c	-	-	-	-	-	-	-	-	-	-	-	-	6.681E-06	5.445E-06	6.06	0.87
1d	-	-	-	-	-	-	-	-	4.585E-06	3.004E-06	5.473E-06	6.170E-06	-	-	4.81	1.37
1e	-	-	-	-	-	-	-	-	-	-	-	-	3.954E-06	4.551E-06	4.25	0.42
1f	-	-	-	-	-	-	-	-	4.397E-06	5.193E-06	9.616E-07	1.039E-06	5.717E-06	3.550E-06	3.48	2.05
1g	-	-	-	-	-	-	-	-	-	-	-	-	1.975E-06	1.889E-06	1.93	0.06
1h	-	-	-	-	-	-	-	-	-	-	3.496E-06	4.442E-06	-	-	3.97	0.67
1i	-	-	-	-	-	-	-	-	-	-	-	5.654E-06	1.656E-06	7.899E-07	2.70	2.59
1j	-	-	-	-	-	-	-	-	-	-	7.190E-06	5.028E-06	-	-	6.11	1.53
1k	-	-	-	-	-	-	-	-	-	-	6.827E-06	6.437E-06	1.206E-06	4.357E-06	4.71	2.57
1l	-	-	-	-	-	-	-	-	-	-	6.181E-07	5.904E-07	-	-	0.60	0.02
1m	-	-	-	-	-	-	-	-	-	-	1.207E-06	1.155E-06	-	-	1.18	0.04
1n	-	-	-	-	-	-	-	-	-	-	1.062E-06	1.110E-06	-	-	1.09	0.03
1o	-	-	-	-	-	-	-	-	-	-	1.800E-06	1.748E-06	-	-	1.77	0.04
1p	-	-	-	-	-	-	-	-	-	-	7.326E-07	1.131E-06	8.980E-08	8.243E-08	0.51	0.51
1q	-	-	1.281E-07	9.590E-08	-	-	-	-	-	-	-	-	-	-	0.11	0.02
1r	-	-	-	-	-	-	-	-	-	-	4.005E-06	5.641E-06	-	-	4.82	1.16
1s	-	-	-	-	-	-	-	-	-	-	-	-	2.187E-07	1.786E-07	0.20	0.03
1t	-	-	-	-	-	-	-	-	-	-	-	-	3.003E-07	8.920E-07	0.60	0.42
1u	-	-	-	-	-	-	-	-	-	-	-	-	2.604E-07	5.746E-07	0.42	0.22
2	-	-	1.841E-06	5.121E-06	2.600E-06	1.779E-06	-	-	8.610E-06	6.856E-06	1.237E-06	1.261E-06	-	-	3.66	2.84
2a	-	-	-	-	-	-	-	-	5.085E-06	5.171E-06	1.362E-06	1.313E-06	-	-	3.23	2.19
2b	-	-	-	-	-	-	-	-	4.978E-06	3.591E-06	-	-	-	-	4.28	0.98
S1	-	-	1.076E-06	2.552E-07	9.286E-07	-	1.140E-06	7.966E-07	-	-	-	-	-	-	0.84	0.35
S1a	-	-	4.189E-07	5.456E-08	7.870E-07	2.068E-07	-	-	-	-	-	-	-	-	0.37	0.32
S1b	-	-	2.611E-07	4.921E-07	-	-	-	-	-	-	-	-	-	-	0.38	0.16
S1c	-	-	1.031E-06	3.872E-07	3.972E-07	1.379E-07	-	-	-	-	-	-	-	-	0.49	0.38
3	-	-	-	-	1.940E-06	1.072E-06	3.041E-06	2.904E-05	1.883E-06	1.595E-06	-	-	-	-	6.43	11.09
3a	-	-	-	-	-	-	-	-	9.838E-07	6.407E-07	-	-	-	-	0.81	0.24
3b	-	-	2.053E-07	1.432E-07	2.446E-07	9.691E-08	3.466E-07	1.329E-07	1.038E-06	8.058E-07	-	-	-	-	0.38	0.35
3c	-	-	-	-	-	-	-	-	8.572E-07	9.786E-07	-	-	-	-	0.92	0.09
3d	-	-	-	-	-	-	-	-	1.574E-06	2.022E-06	-	-	-	-	1.80	0.32
3e	-	-	-	-	-	-	-	-	1.095E-06	1.122E-06	-	-	-	-	1.11	0.02
3f	-	-	2.284E-06	2.284E-06	1.039E-06	1.049E-06	-	-	1.817E-06	1.354E-06	-	-	-	-	1.64	0.57
3g	-	-	5.149E-07	5.913E-07	5.693E-07	7.352E-08	-	-	-	-	-	-	-	-	0.44	0.24
3h	-	-	5.037E-07	3.746E-07	5.231E-07	2.160E-07	1.048E-06	5.064E-07	7.446E-07	9.053E-07	-	-	-	-	0.60	0.28
3i	-	-	4.052E-07	1.775E-07	5.168E-07	3.989E-07	-	-	-	-	-	-	-	-	0.37	0.14
3j	-	-	-	-	-	-	-	-	1.922E-06	1.988E-06	-	-	-	-	1.96	0.05
3k	-	-	-	-	1.290E-06	8.040E-07	-	-	1.410E-06	1.444E-06	-	-	-	-	1.24	0.30
3l	-	-	-	-	-	-	-	-	2.890E-06	2.715E-06	-	-	-	-	2.80	0.12
3m	-	-	1.942E-07	1.615E-07	1.770E-07	1.094E-08	1.614E-06	2.173E-06	2.202E-06	1.665E-06	-	-	-	-	1.02	0.97
3n	6.238E-07	5.477E-07	1.079E-06	1.341E-06	-	-	1.440E-06	5.092E-07	1.905E-06	1.766E-06	-	-	-	-	1.15	0.55
3o	-	-	1.718E-07	1.911E-07	2.185E-07	4.803E-08	2.667E-07	-	3.719E-07	3.079E-07	-	-	-	-	0.23	0.10
3p	7.726E-07	6.717E-07	3.164E-07	2.842E-07	-	-	1.591E-06	-	-	-	-	-	-	-	0.73	0.53
3q	-	-	-	-	-	-	9.709E-07	9.709E-07	1.323E-06	1.198E-06	-	-	-	-	1.12	0.17
3r	2.501E-07	1.703E-07	2.651E-07	1.625E-07	-	-	-	-	-	-	-	-	-	-	0.21	0.05
S2	4.834E-07	4.124E-07	1.054E-07	2.392E-07	-	-	4.982E-07	1.280E-07	-	-	-	-	-	-	0.31	0.18
S2a	1.040E-06	5.674E-07	4.784E-07	3.041E-07	-	-	-	-	-	-	-	-	-	-	0.60	0.31
S2b	-	-	-	-	-	-	2.564E-06	1.079E-06	-	-	-	-	-	-	1.82	1.05
S2c	-	-	-	-	-	-	9.687E-07	1.843E-07	-	-	-	-	-	-	0.58	0.55
S2d	-	-	-	-	-	-	7.387E-07	3.914E-07	-	-	-	-	-	-	0.57	0.25
S2e	-	-	-	-	-	-	3.920E-07	3.652E-07	-	-	-	-	-	-	0.38	0.02
S2f	6.587E-07	4.430E-07	1.059E-06	1.527E-06	-	-	-	-	-	-	-	-	-	-	0.92	0.48
S2g	3.732E-07	3.477E-08	1.660E-07	1.242E-07	-	-	-	-	-	-	-	-	-	-	0.17	0.14
S3	1.215E-06	1.128E-06	9.093E-07	3.507E-06	-	-	2.051E-06	1.885E-06	-	-	-	-	-	-	1.78	0.96
S3a	-	-	-	-	-	-	1.847E-06	1.054E-06	-	-	-	-	-	-	1.45	0.56
S3b	-	-	-	-	-	-	1.610E-07	1.250E-07	-	-	-	-	-	-	0.14	0.03
S3c	-	-	-	-	-	-	5.254E-07	1.634E-06	-	-	-	-	-	-	1.08	0.78
S4	-	-	-	-	4.777E-07	3.151E-07	2.221E-06	2.365E-06	1.850E-06	1.219E-06	-	-	-	-	1.41	0.88
S4a	-	-	2.557E-07	3.160E-07	4.657E-07	1.521E-07	-	-	-	-	-	-	-	-	0.30	0.13
S4b	-	-	7.638E-07	3.432E-07	7.492E-07	3.189E-07	3.199E-06	6.717E-07	6.749E-07	6.477E-07	-	-	-	-	0.92	0.94
S4c	-	-	3.537E-07	3.940E-07	4.064E-07	2.162E-07	3.424E-07	2.727E-07	-	-	-	-	-	-	0.33	0.07
S5	3.211E-06	2.662E-06	2.364E-06	2.717E-06	-	-	1.940E-06	1.527E-06	1.816E-05	-	-	-	-	-	4.65	5.98
S5a	-	-	-	-	-	-	-	-	1.106E-06	1.118E-06	-	-	-	-	1.11	0.01
S6	4.603E-07	4.867E-07	8.924E-07	2.745E-07	-	-	-	-	-	-	-	-	-	-	0.53	0.26
S6a	8.188E-07	7.116E-07	4.019E-07	2.849E-07	-	-	-	-	-	-	-	-	-	-	0.55	0.25
S7	1.375E-06	1.136E-06	1.438E-06	1.255E-06	-	-	-	-	-	-	-	-	-	-	1.30	0.13
S7a	8.341E-07	7.824E-07	1.094E-06	2.216E-07	-	-	-	-	-	-	-	-	-	-	0.73	0.37
S7b	6.019E-07	5.326E-07	1.572E-06	1.955E-06	-	-	-	-	-	-	-	-	-	-	1.17	0.71

Compound Number	JACS '23 DATA Z Score				PAMPA 3 Z Score		PAMPA 4 Z Score		PAMPA 5 Z Score		PAMPA 6 Z score		PAMPA 7 Z score	
	Well 1	Well 2	Well 1	Well 2	Well 1	Well 2	Well 1	Well 2	Well 1	Well 2	Well 1	Well 2	Well 1	Well 2
Theophylline	-0.17	-0.52	0.07	0.18	-1.28	-1.83	0.80	2.48	0.76	0.25	-0.34	-0.19	-0.25	0.04
Diclofenac	-0.03	-0.43	0.58	1.78	-1.94	-2.01	0.64	0.31	0.18	0.53	0.47	0.53	-0.32	-0.28
Chloramphenicol	-0.12	-0.45	2.64	0.76	-1.41	-1.41	0.43	-0.74	-0.22	0.04	0.24	0.60	-0.25	-0.10
1	1.95	1.69	-0.72	-0.81	#VALUE!	#VALUE!	0.07	-0.51	-0.38	-0.39	#VALUE!	#VALUE!	-0.14	-0.76
1a	-0.18	-0.24	-0.45	-0.47	#VALUE!	#VALUE!	-0.23	-0.46	-0.34	-0.32	#VALUE!	#VALUE!	2.83	-0.14
1b	#VALUE!	#VALUE!	#VALUE!	#VALUE!	#VALUE!	#VALUE!	#VALUE!	#VALUE!	0.79	1.16	-1.28	-1.10	0.00	0.43
1c	#VALUE!	#VALUE!	#VALUE!	#VALUE!	#VALUE!	#VALUE!	#VALUE!	#VALUE!	#VALUE!	#VALUE!	#VALUE!	#VALUE!	0.71	-0.71
1d	#VALUE!	#VALUE!	#VALUE!	#VALUE!	#VALUE!	#VALUE!	#VALUE!	#VALUE!	-0.16	-1.32	0.49	1.00	#VALUE!	#VALUE!
1e	#VALUE!	#VALUE!	#VALUE!	#VALUE!	#VALUE!	#VALUE!	#VALUE!	#VALUE!	#VALUE!	#VALUE!	#VALUE!	#VALUE!	-0.71	0.71
1f	#VALUE!	#VALUE!	#VALUE!	#VALUE!	#VALUE!	#VALUE!	#VALUE!	#VALUE!	0.45	0.84	-1.22	-1.19	1.09	0.04
1g	#VALUE!	#VALUE!	#VALUE!	#VALUE!	#VALUE!	#VALUE!	#VALUE!	#VALUE!	#VALUE!	#VALUE!	#VALUE!	#VALUE!	0.71	-0.71
1h	#VALUE!	#VALUE!	#VALUE!	#VALUE!	#VALUE!	#VALUE!	#VALUE!	#VALUE!	#VALUE!	#VALUE!	-0.71	0.71	#VALUE!	#VALUE!
1i	#VALUE!	#VALUE!	#VALUE!	#VALUE!	#VALUE!	#VALUE!	#VALUE!	#VALUE!	#VALUE!	#VALUE!	#VALUE!	1.14	-0.40	-0.74
1j	#VALUE!	#VALUE!	#VALUE!	#VALUE!	#VALUE!	#VALUE!	#VALUE!	#VALUE!	#VALUE!	#VALUE!	0.71	-0.71	#VALUE!	#VALUE!
1k	#VALUE!	#VALUE!	#VALUE!	#VALUE!	#VALUE!	#VALUE!	#VALUE!	#VALUE!	#VALUE!	#VALUE!	0.82	0.67	-1.36	-0.14
1l	#VALUE!	#VALUE!	#VALUE!	#VALUE!	#VALUE!	#VALUE!	#VALUE!	#VALUE!	#VALUE!	#VALUE!	0.71	-0.71	#VALUE!	#VALUE!
1m	#VALUE!	#VALUE!	#VALUE!	#VALUE!	#VALUE!	#VALUE!	#VALUE!	#VALUE!	#VALUE!	#VALUE!	0.71	-0.71	#VALUE!	#VALUE!
1n	#VALUE!	#VALUE!	#VALUE!	#VALUE!	#VALUE!	#VALUE!	#VALUE!	#VALUE!	#VALUE!	#VALUE!	-0.71	0.71	#VALUE!	#VALUE!
1o	#VALUE!	#VALUE!	#VALUE!	#VALUE!	#VALUE!	#VALUE!	#VALUE!	#VALUE!	#VALUE!	#VALUE!	0.71	-0.71	#VALUE!	#VALUE!
1p	#VALUE!	#VALUE!	#VALUE!	#VALUE!	#VALUE!	#VALUE!	#VALUE!	#VALUE!	#VALUE!	#VALUE!	0.43	1.21	-0.81	-0.83
1q	#VALUE!	#VALUE!	0.71	-0.71	#VALUE!	#VALUE!	#VALUE!	#VALUE!	#VALUE!	#VALUE!	#VALUE!	#VALUE!	#VALUE!	#VALUE!
1r	#VALUE!	#VALUE!	#VALUE!	#VALUE!	#VALUE!	#VALUE!	#VALUE!	#VALUE!	#VALUE!	#VALUE!	-0.71	0.71	#VALUE!	#VALUE!
1s	#VALUE!	#VALUE!	#VALUE!	#VALUE!	#VALUE!	#VALUE!	#VALUE!	#VALUE!	#VALUE!	#VALUE!	#VALUE!	#VALUE!	0.71	-0.71
1t	#VALUE!	#VALUE!	#VALUE!	#VALUE!	#VALUE!	#VALUE!	#VALUE!	#VALUE!	#VALUE!	#VALUE!	#VALUE!	#VALUE!	-0.71	0.71
1u	#VALUE!	#VALUE!	#VALUE!	#VALUE!	#VALUE!	#VALUE!	#VALUE!	#VALUE!	#VALUE!	#VALUE!	#VALUE!	#VALUE!	-0.71	0.71
2	#VALUE!	#VALUE!	-0.64	0.51	-0.37	-0.66	#VALUE!	#VALUE!	1.74	1.12	-0.85	-0.85	#VALUE!	#VALUE!
2a	#VALUE!	#VALUE!	#VALUE!	#VALUE!	#VALUE!	#VALUE!	#VALUE!	#VALUE!	0.85	0.89	-0.85	-0.88	#VALUE!	#VALUE!
2b	#VALUE!	#VALUE!	#VALUE!	#VALUE!	#VALUE!	#VALUE!	#VALUE!	#VALUE!	0.71	-0.71	#VALUE!	#VALUE!	#VALUE!	#VALUE!
S1	#VALUE!	#VALUE!	0.67	-1.66	0.25	#VALUE!	0.85	-0.12	#VALUE!	#VALUE!	#VALUE!	#VALUE!	#VALUE!	#VALUE!
S1a	#VALUE!	#VALUE!	0.16	-0.98	1.32	-0.50	#VALUE!	#VALUE!	#VALUE!	#VALUE!	#VALUE!	#VALUE!	#VALUE!	#VALUE!
S1b	#VALUE!	#VALUE!	-0.71	0.71	#VALUE!	#VALUE!	#VALUE!	#VALUE!	#VALUE!	#VALUE!	#VALUE!	#VALUE!	#VALUE!	#VALUE!
S1c	#VALUE!	#VALUE!	1.42	-0.27	-0.24	-0.92	#VALUE!	#VALUE!	#VALUE!	#VALUE!	#VALUE!	#VALUE!	#VALUE!	#VALUE!
3	#VALUE!	#VALUE!	#VALUE!	#VALUE!	-0.40	-0.48	-0.31	2.04	-0.41	-0.44	#VALUE!	#VALUE!	#VALUE!	#VALUE!
3a	#VALUE!	#VALUE!	#VALUE!	#VALUE!	#VALUE!	#VALUE!	#VALUE!	#VALUE!	0.71	-0.71	#VALUE!	#VALUE!	#VALUE!	#VALUE!
3b	#VALUE!	#VALUE!	-0.49	-0.67	-0.38	-0.80	-0.09	-0.69	1.89	1.22	#VALUE!	#VALUE!	#VALUE!	#VALUE!
3c	#VALUE!	#VALUE!	#VALUE!	#VALUE!	#VALUE!	#VALUE!	#VALUE!	#VALUE!	-0.71	0.71	#VALUE!	#VALUE!	#VALUE!	#VALUE!
3d	#VALUE!	#VALUE!	#VALUE!	#VALUE!	#VALUE!	#VALUE!	#VALUE!	#VALUE!	-0.71	0.71	#VALUE!	#VALUE!	#VALUE!	#VALUE!
3e	#VALUE!	#VALUE!	#VALUE!	#VALUE!	#VALUE!	#VALUE!	#VALUE!	#VALUE!	-0.71	0.71	#VALUE!	#VALUE!	#VALUE!	#VALUE!
3f	#VALUE!	#VALUE!	1.12	1.12	-1.04	-1.02	#VALUE!	#VALUE!	0.31	-0.49	#VALUE!	#VALUE!	#VALUE!	#VALUE!
3g	#VALUE!	#VALUE!	0.32	0.63	0.54	-1.49	#VALUE!	#VALUE!	#VALUE!	#VALUE!	#VALUE!	#VALUE!	#VALUE!	#VALUE!
3h	#VALUE!	#VALUE!	-0.36	-0.82	-0.29	-1.40	1.61	-0.35	0.51	1.09	#VALUE!	#VALUE!	#VALUE!	#VALUE!
3i	#VALUE!	#VALUE!	0.22	-1.39	1.00	0.17	#VALUE!	#VALUE!	#VALUE!	#VALUE!	#VALUE!	#VALUE!	#VALUE!	#VALUE!
3j	#VALUE!	#VALUE!	#VALUE!	#VALUE!	#VALUE!	#VALUE!	#VALUE!	#VALUE!	-0.71	0.71	#VALUE!	#VALUE!	#VALUE!	#VALUE!
3k	#VALUE!	#VALUE!	#VALUE!	#VALUE!	0.18	-1.46	#VALUE!	#VALUE!	0.58	0.70	#VALUE!	#VALUE!	#VALUE!	#VALUE!
3l	#VALUE!	#VALUE!	#VALUE!	#VALUE!	#VALUE!	#VALUE!	#VALUE!	#VALUE!	0.71	-0.71	#VALUE!	#VALUE!	#VALUE!	#VALUE!
3m	#VALUE!	#VALUE!	-0.85	-0.89	-0.87	-1.04	0.61	1.18	1.21	0.66	#VALUE!	#VALUE!	#VALUE!	#VALUE!
3n	-0.96	-1.10	-0.13	0.34	#VALUE!	#VALUE!	0.52	-1.17	1.37	1.12	#VALUE!	#VALUE!	#VALUE!	#VALUE!
3o	#VALUE!	#VALUE!	-0.51	-0.33	-0.06	-1.69	0.40	#VALUE!	1.40	0.79	#VALUE!	#VALUE!	#VALUE!	#VALUE!
3p	0.09	-0.10	-0.78	-0.84	#VALUE!	#VALUE!	1.64	#VALUE!	#VALUE!	#VALUE!	#VALUE!	#VALUE!	#VALUE!	#VALUE!
3q	#VALUE!	#VALUE!	#VALUE!	#VALUE!	#VALUE!	#VALUE!	-0.83	-0.83	1.19	0.47	#VALUE!	#VALUE!	#VALUE!	#VALUE!
3r	0.72	-0.79	1.00	-0.93	#VALUE!	#VALUE!	#VALUE!	#VALUE!	#VALUE!	#VALUE!	#VALUE!	#VALUE!	#VALUE!	#VALUE!
S2	0.98	0.57	-1.16	-0.41	#VALUE!	#VALUE!	1.06	-1.04	#VALUE!	#VALUE!	#VALUE!	#VALUE!	#VALUE!	#VALUE!
S2a	1.41	-0.10	-0.38	-0.93	#VALUE!	#VALUE!	#VALUE!	#VALUE!	#VALUE!	#VALUE!	#VALUE!	#VALUE!	#VALUE!	#VALUE!
S2b	#VALUE!	#VALUE!	#VALUE!	#VALUE!	#VALUE!	#VALUE!	0.71	-0.71	#VALUE!	#VALUE!	#VALUE!	#VALUE!	#VALUE!	#VALUE!
S2c	#VALUE!	#VALUE!	#VALUE!	#VALUE!	#VALUE!	#VALUE!	0.71	-0.71	#VALUE!	#VALUE!	#VALUE!	#VALUE!	#VALUE!	#VALUE!
S2d	#VALUE!	#VALUE!	#VALUE!	#VALUE!	#VALUE!	#VALUE!	0.71	-0.71	#VALUE!	#VALUE!	#VALUE!	#VALUE!	#VALUE!	#VALUE!
S2e	#VALUE!	#VALUE!	#VALUE!	#VALUE!	#VALUE!	#VALUE!	0.71	-0.71	#VALUE!	#VALUE!	#VALUE!	#VALUE!	#VALUE!	#VALUE!
S2f	-0.55	-1.00	0.29	1.27	#VALUE!	#VALUE!	#VALUE!	#VALUE!	#VALUE!	#VALUE!	#VALUE!	#VALUE!	#VALUE!	#VALUE!
S2g	1.39	-0.98	-0.06	-0.35	#VALUE!	#VALUE!	#VALUE!	#VALUE!	#VALUE!	#VALUE!	#VALUE!	#VALUE!	#VALUE!	#VALUE!
S3	-0.59	-0.68	-0.91	1.80	#VALUE!	#VALUE!	0.28	0.11	#VALUE!	#VALUE!	#VALUE!	#VALUE!	#VALUE!	#VALUE!
S3a	#VALUE!	#VALUE!	#VALUE!	#VALUE!	#VALUE!	#VALUE!	0.71	-0.71	#VALUE!	#VALUE!	#VALUE!	#VALUE!	#VALUE!	#VALUE!
S3b	#VALUE!	#VALUE!	#VALUE!	#VALUE!	#VALUE!	#VALUE!	0.71	-0.71	#VALUE!	#VALUE!	#VALUE!	#VALUE!	#VALUE!	#VALUE!
S3c	#VALUE!	#VALUE!	#VALUE!	#VALUE!	#VALUE!	#VALUE!	-0.71	0.71	#VALUE!	#VALUE!	#VALUE!	#VALUE!	#VALUE!	#VALUE!
S4	#VALUE!	#VALUE!	#VALUE!	#VALUE!	-1.06	-1.24	0.92	1.09	0.50	-0.22	#VALUE!	#VALUE!	#VALUE!	#VALUE!
S4a	#VALUE!	#VALUE!	-0.32	0.14	1.28	-1.11	#VALUE!	#VALUE!	#VALUE!	#VALUE!	#VALUE!	#VALUE!	#VALUE!	#VALUE!
S4b	#VALUE!	#VALUE!	-0.17	-0.62	-0.18	-0.64	2.43	-0.27	-0.26	-0.29	#VALUE!	#VALUE!	#VALUE!	#VALUE!
S4c	#VALUE!	#VALUE!	0.31	0.86	1.03	-1.56	0.16	-0.79	#VALUE!	#VALUE!	#VALUE!	#VALUE!	#VALUE!	#VALUE!
S5	-0.24	-0.33	-0.38	-0.32	#VALUE!	#VALUE!	-0.45	-0.52	2.26	#VALUE!	#VALUE!	#VALUE!	#VALUE!	#VALUE!
S5a	#VALUE!	#VALUE!	#VALUE!	#VALUE!	#VALUE!	#VALUE!	#VALUE!	#VALUE!	-0.71	0.71	#VALUE!	#VALUE!	#VALUE!	#VALUE!
S6	-0.26	-0.16	1.40	-0.98	#VALUE!	#VALUE!	#VALUE!	#VALUE!	#VALUE!	#VALUE!	#VALUE!	#VALUE!	#VALUE!	#VALUE!
S6a	1.05	0.62	-0.60	-1.07	#VALUE!	#VALUE!	#VALUE!	#VALUE!	#VALUE!	#VALUE!	#VALUE!	#VALUE!	#VALUE!	#VALUE!
S7	0.55	-1.23	1.03	-0.35	#VALUE!	#VALUE!	#VALUE!	#VALUE!	#VALUE!	#VALUE!	#VALUE!	#VALUE!	#VALUE!	#VALUE!
S7a	0.28	0.13	0.98	-1.39	#VALUE!	#VALUE!	#VALUE!	#VALUE!	#VALUE!	#VALUE!	#VALUE!	#VALUE!	#VALUE!	#VALUE!
S7b	-0.80	-0.89	0.57	1.11	#VALUE!	#VALUE!	#VALUE!	#VALUE!	#VALUE!	#VALUE!	#VALUE!	#VALUE!	#VALUE!	#VALUE!

Cells highlighted in red represent data values where z ≥ 2 and cells highlighted in yellow represent data values where z ≥ 1.

Solution-Phase Structural Determination

The solution conformation of **1b** was determined from NMR spectra of **1b** acquired in DMSO-d_6, 5:1 $CDCl_3$:DMSO-d_6, and 9:1 MeOD-d_3:D_2O. Proton signals were assigned by analysis of standard HSQC, HMBC, and NOESY experiments. Distance restraints were obtained from pairs of NOE correlations from a ^1H-^1H NOESY experiment using a 100ms mixing time and 4s interscan delay. Integration of NOESY cross-peaks was performed with MestreNova (version 14.2.3). Internuclear distances *r* were calculated according to the following equation, using cross-peak intensities *I* :

$$r_{unk} = r_{ref}\left(\frac{I_{ref}}{I_{unk}}\right)^{\frac{1}{6}}$$

The reference values I_{ref} and r_{ref} were acquired by integration of an NOE cross-peak belonging to a pair of protons with a known internuclear distance. In the DMSO-d_6 structure, the fixed reference distance H21-H21' (1.8Å) was corroborated by distance H12-H12', suggesting sufficiently linear NOE buildup for stratification of the larger dataset. In the 5:1 $CDCl_3$:DMSO-d_6 structure, the fixed reference distance H12-H12' (1.8Å) was corroborated by distance H16-H16', suggesting sufficiently linear NOE buildup for stratification of the larger dataset. In the 9:1 MeOD-d_3:D_2O structure, the fixed reference distance H34-H34' (1.8Å) was corroborated by distance H21-H21', suggesting sufficiently linear NOE buildup for stratification of the larger dataset. Attempts to determine distance restraints in $CDCl_3$, 9:1 $CDCl_3$:DMSO-d_6, and 5:1 DMSO-d_6:D_2O were unsuccessful due to poor solubility, limiting signal resolution and/or aggregation effects obscuring true NOESY correlations.

To identify the solution-state conformer corresponding to the experimental NOE data, we performed a structural screen across the full chloroform MD trajectory. Pairwise interproton distances were calculated for three critical atom pairs: H12'-H15, H16'-H17, and H16'-H23. Applying a deviation tolerance of 0.7 Å from the experimental restraints yielded only four conformations. The distance triplets ($d_{12'-15}$, $d_{16'-17}$, and$d_{16'-23}$) for these candidates were $[3.5, 3.8, 2.7]$, $[3.7, 4.3, 2.5]$, $[3.6, 3.0, 2.2]$, and $[3.7, 4.1, 2.9]$ Å, respectively. Consequently, the first conformation was selected as the final representative NMR structure due to its minimal aggregate deviation from the experimental values.

Polycycle 1b in DMSO-d_6

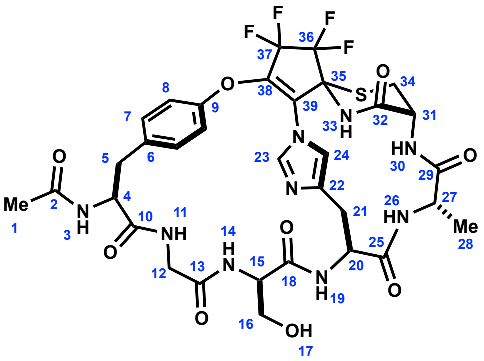

Chemical Formula: $C_{33}H_{35}F_4N_9O_9S$

^{1}H NMR (500 MHz, DMSO) δ 8.94 (bs, 1H), 8.82 (d, *J* = 6.5 Hz, 1H), 8.40 (d, *J* = 9.4 Hz, 1H), 8.26 (d, *J* = 7.6 Hz, 1H), 8.06 (d, *J* = 5.0 Hz, 1H), 7.25 (s, 1H), 7.23 (d, *J* = 1.3 Hz, 1H), 7.18 – 7.09 (m, 3H), 7.03 (d, *J* = 8.3 Hz, 2H), 6.07 (d, *J* = 9.3 Hz, 1H), 4.92 (t, *J* = 5.6 Hz, 1H), 4.70 (dt, *J* = 9.0, 4.1 Hz, 1H), 4.59 (dt, *J* = 9.4, 2.9 Hz, 1H), 4.49 (ddd, *J* = 11.7, 7.7, 4.5 Hz, 1H), 4.10 (td, *J* = 6.8, 3.5 Hz, 1H), 4.00 (dd, *J* = 17.7, 5.6 Hz, 1H), 3.96 – 3.84 (m, 1H), 3.76 – 3.57 (m, 3H), 3.41 (dd, *J* = 14.5, 2.6 Hz, 1H), 3.31 – 3.26 (m, 1H), 2.98 – 2.66 (m, 3H), 2.47 – 2.39 (m, 1H), 1.83 (s, 3H), 1.27 (d, *J* = 7.4 Hz, 3H). **^{13}C NMR** (126 MHz, DMSO) δ 171.3, 170.2, 169.6, 168.9, 168.8, 168.6, 166.9, 154.3, 141.2, 138.4, 137.5, 134.2, 131.1, 125.9, 119.3, 118.5, 116.2, 116.1, 67.3, 60.7, 57.4, 54.0, 51.0, 50.6, 45.8, 41.8, 37.1, 30.1, 28.7, 22.4, 17.0.

Table S5. Tabulated chemical shifts and correlations for polycycle **1b** identified in DMSO-*d*$_6$.

	^{13}C NMR	^{1}H NMR	HMBC	NOESY
**1**	22.4	1.83 (s, 3H)	→ 2	→ 3
**2**	168.9	-	→ 1, 3	
**3**	-	8.26 (d, *J* = 7.6 Hz, 1H)	→ 2	→ 1, 4, 5, 11
**4**	54.0	4.49 (ddd, *J* = 11.7, 7.7, 4.5 Hz, 1H)	→ 5’, 5”	→ 3, 5, 7
**5’**	37.1	2.98 – 2.66 (m, 3H, overlap)	→ 4, 6, 7	→ 3, 4, 7
**5’’**	37.1	2.98 – 2.66 (m, 3H, overlap)	→ 4, 6, 7, 10	→ 3, 4, 7
**6**	134.2	-	→ 5’, 5”, 8	
**7**	131.1	7.18 – 7.09 (m, 3H, overlap)	→ 5’, 5”, 8, 9	→ 4, 5
**8**	116.2	7.03 (d, *J* = 8.3 Hz, 2H)	→ 6, 7, 9	→ 5
**9**	154.3	-	→ 7, 8	
**10**	169.6	-	→ 5”	
**11**	-	7.18 – 7.09 (m, 3H, overlap)		→ 12
**12’**	41.8	3.76 – 3.57 (m, 3H, overlap)	→ 13	→ 11, 14
**12’’**	41.8	4.00 (dd, *J* = 17.7, 5.6 Hz, 1H)	→ 13	→ 11, 14
**13**	168.8	-	→ 12’, 12”, 14	
**14**	-	8.82 (d, *J* = 6.5 Hz, 1H)	→ 13	→ 12, 15
**15**	57.4	4.10 (td, *J* = 6.8, 3.5 Hz, 1H)		→ 14, 16
**16’**	60.7	3.76 – 3.57 (m, 3H, overlap)		→ 14
**16’’**	60.7	3.76 – 3.57 (m, 3H, overlap)		→ 14
**17**	-	4.92 (t, *J* = 5.6 Hz, 1H)		→ H_2O
**18**	168.6	-	→ 19	
**19**	-	8.40 (d, *J* = 9.4 Hz, 1H)	→ 18	→ 16, 19, 20, 21
**20**	51.0	4.70 (dt, *J* = 9.0, 4.1 Hz, 1H)	→ 23	→ 19, 21, 26
**21’**	28.7	2.47 – 2.39 (m, 1H)		→ 19, 20, 26

**21''**	28.7	3.31 – 3.26 (m, 1H)		→ 19, 20, 26
**22**	138.4	-	→ 23	
**23**	137.5	7.23 (d, *J* = 1.3 Hz, 1H)	→ 20, 22, 24	
**24**	119.3	7.25 (s, 1H)	→ 23, 39	→ 21, 30
**25**	170.2	-	→ 26	
**26**	-	8.06 (d, *J* = 5.0 Hz, 1H)	→ 25	→ 20, 21, 27, 28, 30
**27**	50.6	3.96 – 3.84 (m, 1H)	→ 28, 29	→ 26, 28
**28**	17.0	1.27 (d, *J* = 7.4 Hz, 3H)	→ 27, 29	→ 26
**29**	171.3	-	→ 27, 28, 30	
**30**	-	6.07 (d, *J* = 9.3 Hz, 1H)	→ 29, 31	→ 26, 28, 31, 34,
**31**	45.8	4.59 (dt, *J* = 9.4, 2.9 Hz, 1H)	→ 32, 34'	→ 30, 34
**32**	166.9	-	→ 31, 34'	
**33**	-	8.94 (bs, 1H)		→ H_2O
**34'**	30.1	2.98 – 2.66 (m, 3H overlap)	→ 31, 32, 35	→ 34
**34''**	30.1	3.41 (dd, *J* = 14.5, 2.6 Hz, 1H)		→ 34
**35**	67.3 (m, 1C)	-	→ 34'	
**36**	118.5 (m, 1C)	-		
**37**	116.1 (m, 1C)	-		
**38**	141.2 (m, 1C)	-		
**39**	125.9 (m, 1C)	-		

Table S6. Tabulated distance restraints for polycycle **1b** identified in DMSO-*d*$_6$.

NOE Correlation	I_{unk} (above diagonal)	I_{unk} (below diagonal)	I_{unk} (average)	r_{unk} (Å)
1↔3	0.06	0.07	0.07	2.8
3↔4	0.02	0.04	0.03	3.3
12'↔12"	0.71	0.7	0.71	1.9
12'↔14	0.04	0.02	0.03	3.3
14↔15	0.05	0.06	0.06	2.9
14↔19	0.04	0.04	0.04	3.1
19↔20	0.10	0.12	0.11	2.6
19↔21"	0.05	0.05	0.05	3.0
20↔26	0.08	0.06	0.07	2.8
21'↔21"	-	1.00	-	1.8
21'↔26	0.17	0.26	0.22	2.3
24↔30	0.02	0.03	0.03	3.3
26↔27	0.04	0.05	0.05	3.0
26↔28	0.09	0.11	0.10	2.6
26↔30	0.05	0.04	0.05	3.0
27↔28	0.15	0.10	0.13	2.6
28↔30	0.01	0.02	0.02	3.7
30↔31	0.11	0.07	0.09	2.7
31↔34'	0.07	-	-	2.8
31↔34"	0.12	-	-	2.6

Indicated geminal protons (blue) were used as references distances where I_{ref} = 1.00 and r_{ref} = 1.8 Å.

HSQC

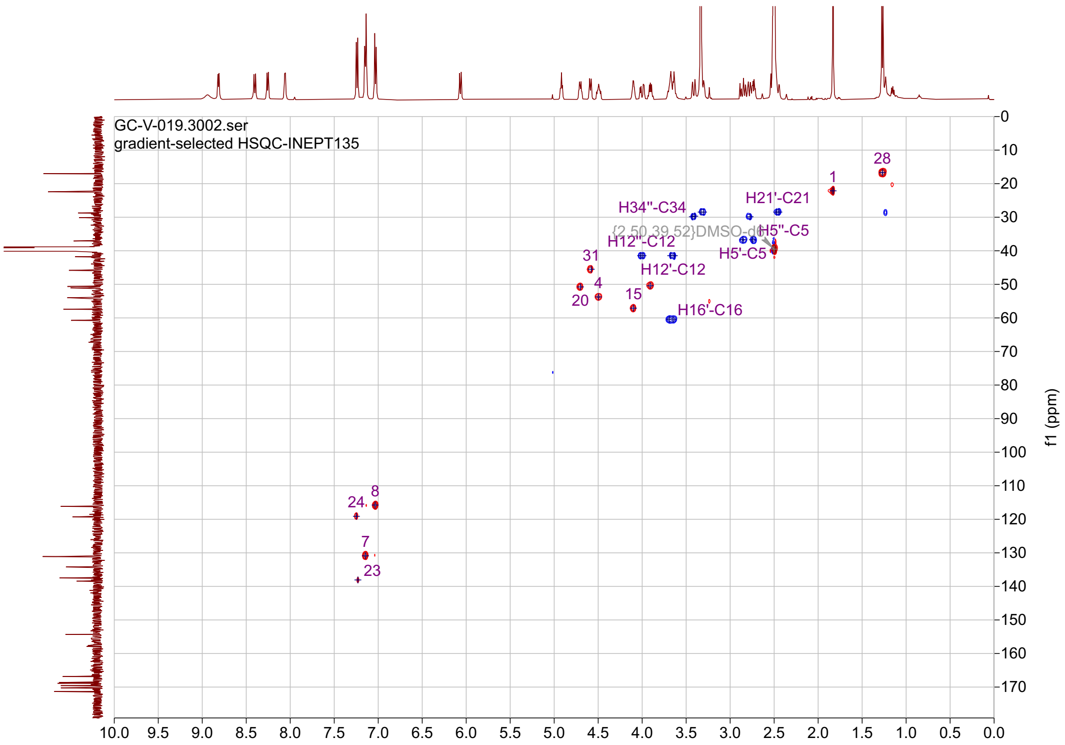
GC-V-019.3002.ser
gradient-selected HSQC-INEPT135
28
1
H21'-C21
H34''-C34
{2.50,39.52}DMSO-d6
H5''-C5
H12''-C12
H5'-C5
31
H12'-C12
4
20
15
H16'-C16
8
24
7
23
f1 (ppm)

HMBC

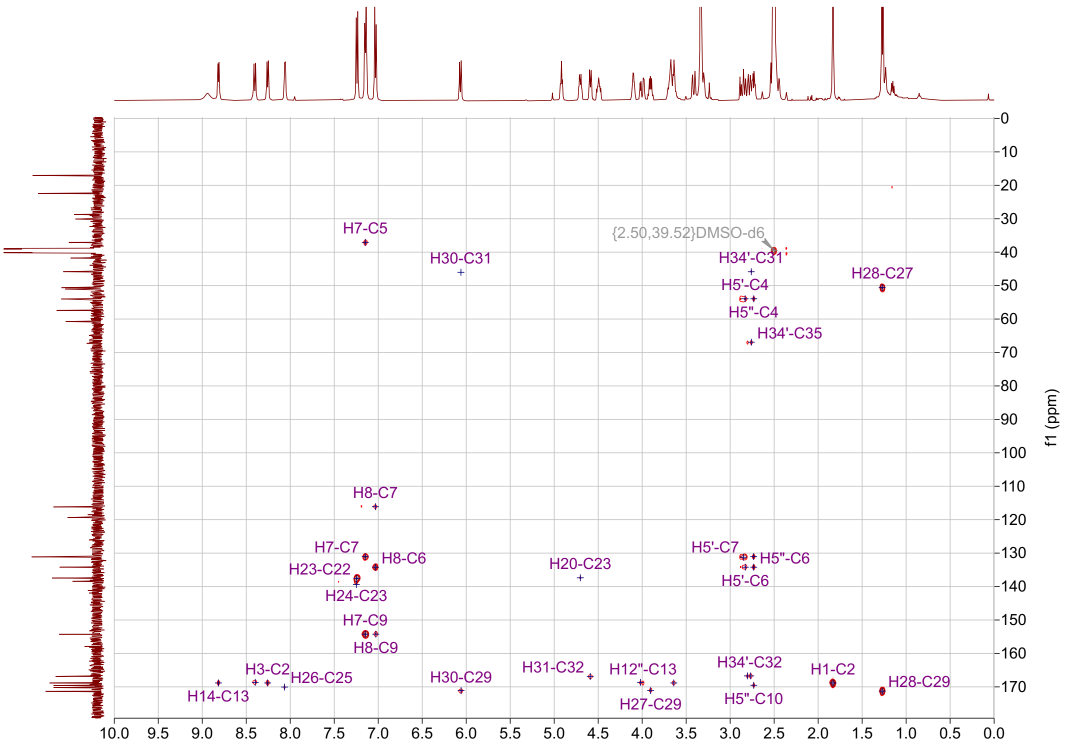
H7-C5
H30-C31
{2.50,39.52}DMSO-d6
H34'-C31
H28-C27
H5'-C4
H5''-C4
H34'-C35
H8-C7
H7-C7
H8-C6
H20-C23
H5'-C7
H5''-C6
H23-C22
H5'-C6
H24-C23
H7-C9
H8-C9
H3-C2
H26-C25
H14-C13
H30-C29
H31-C32
H12''-C13
H27-C29
H34'-C32
H5''-C10
H1-C2
H28-C29
f1 (ppm)

NOESY

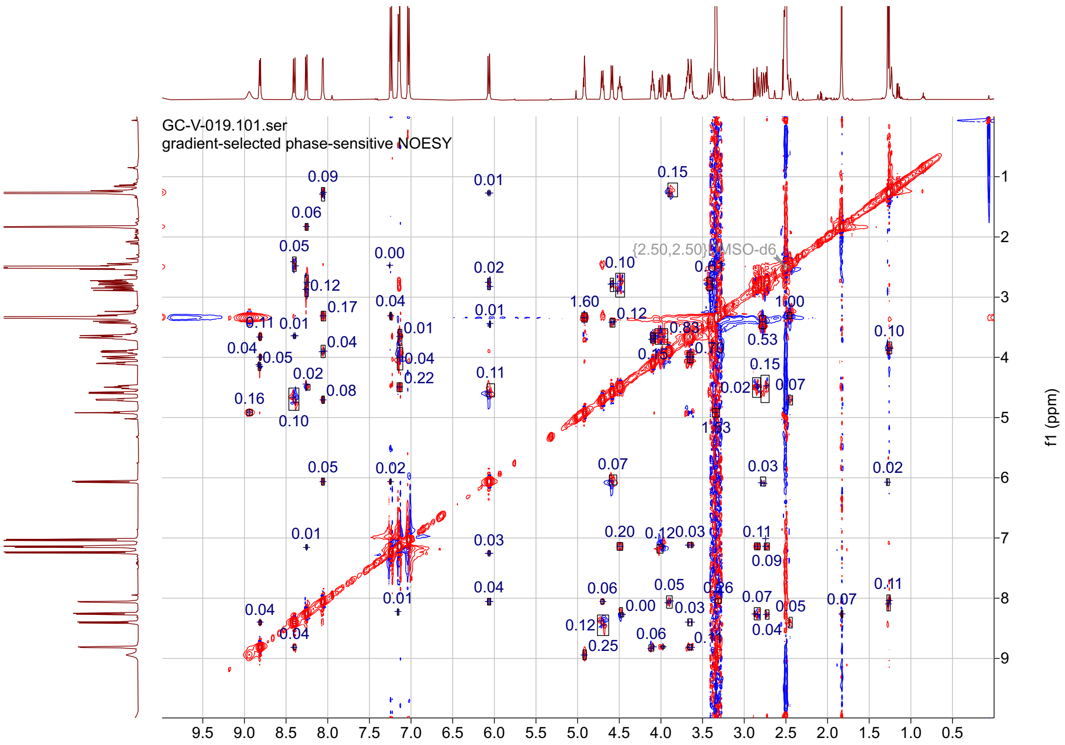

GC-V-019.101.ser
gradient-selected phase-sensitive NOESY
{2.50,2.50} DMSO-d6
f1 (ppm)

Polycycle 1b in 5:1 $CDCl_3$: DMSO-d6

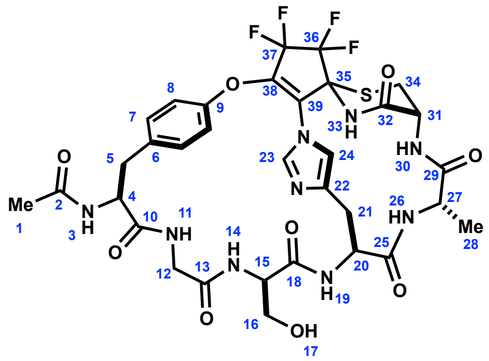

Chemical Formula: $C_{33}H_{35}F_4N_9O_9S$

^{1}H NMR (500 MHz, 5:1 $CDCl_3$: DMSO) δ 8.98 (bs, 1H), 8.65 (d, *J* = 6.2 Hz, 1H), 8.41 (d, *J* = 8.9 Hz, 1H), 8.05 (d, *J* = 5.1 Hz, 1H), 7.64 (d, *J* = 7.2 Hz, 1H), 7.10 – 7.00 (m, 3H), 6.94 (s, 1H), 6.88 (d, *J* = 7.8 Hz, 2H), 6.67 (d, *J* = 6.7 Hz, 1H), 6.33 (d, *J* = 8.6 Hz, 1H), 4.91 (bs, 1H), 4.70 (dt, *J* = 8.4, 3.9 Hz, 1H), 4.65 (dd, *J* = 8.3, 4.0 Hz, 1H), 4.28 (q, *J* = 7.3 Hz, 1H), 4.20 (dd, *J* = 16.7, 6.4 Hz, 1H), 4.14 (dd, *J* = 6.6, 3.5 Hz, 1H), 3.93 (d, *J* = 11.4 Hz, 1H), 3.80 (q, *J* = 6.7 Hz, 1H), 3.69 (s, 1H), 3.66 (s, 1H), 3.31 (td, *J* = 14.8, 3.4 Hz, 2H), 2.85 – 2.75 (m, 2H), 2.67 (dd, *J* = 14.6, 3.4 Hz, 1H), 2.61 – 2.53 (m, 1H), 1.89 (s, 3H), 1.35 (d, *J* = 7.1 Hz, 3H).

Table S7. Tabulated chemical shifts and correlations for polycycle **1b** identified in 5:1 $CDCl_3$:DMSO-d_6.

	^{1}H NMR	NOESY
**1**	1.89 (s, 3H)	→ 3
**3**	7.64 (d, *J* = 7.2 Hz, 1H)	→ 1, 4
**4**	4.28 (q, *J* = 7.3 Hz, 1H)	→ 3, 11
**5**	2.85 – 2.75 (m, 2H, overlap)	
**5'**	2.85 – 2.75 (m, 2H, overlap)	
**7**	7.10 – 7.00 (m, 3H, overlap)	
**8**	6.88 (d, *J* = 7.8 Hz, 2H)	
**11**	6.67 (d, *J* = 6.7 Hz, 1H)	→ 4, 12
**12**	4.20 (dd, *J* = 16.7, 6.4 Hz, 1H)	→ 11, 12'
**12'**	3.66 (s, 1H)	→ 14, 15, 23
**14**	8.65 (d, *J* = 6.2 Hz, 1H)	→ 12', 15, 16', 19
**15**	4.14 (dd, *J* = 6.6, 3.5 Hz, 1H)	→ 12', 14, 16
**16**	3.93 (d, *J* = 11.4 Hz, 1H)	→ 15, 16'
**16'**	3.69 (s, 1H)	→ 14, 16, 17, 23
**17**	4.91 (bs, 1H)	→ 16'
**19**	8.41 (d, *J* = 8.9 Hz, 1H)	→ 14, 20
**20**	4.70 (dt, *J* = 8.4, 3.9 Hz, 1H)	→ 19, 21'
**21**	3.31 (m, 2H, overlap)	→ 21'
**21'**	2.61 – 2.53 (m, 1H)	→ 20, 21
**23**	6.94 (s, 1H)	→ 12', 16'
**24**	7.10 – 7.00 (m, 3H, overlap)	
**26**	8.05 (d, *J* = 5.1 Hz, 1H)	→ 27, 28
**27**	3.80 (q, *J* = 6.7 Hz, 1H)	→ 26, 28
**28**	1.35 (d, *J* = 7.1 Hz, 3H)	→ 26, 27
**30**	6.33 (d, *J* = 8.6 Hz, 1H)	→ 31

31	4.65 (dd, J = 8.3, 4.0 Hz, 1H)	→ 30, 34’
33	8.98 (bs, 1H)	
34	3.31 (m, 2H, overlap)	→ 34’
34’	2.67 (dd, J = 14.6, 3.4 Hz, 1H)	→ 31, 34

Table S8. Tabulated distance restraints for polycycle **1b** identified in 5:1 $CDCl_3$:DMSO-d_6.

NOE Correlation	I_{unk} (above diagonal)	I_{unk} (below diagonal)	I_{unk} (average)	r_{unk} (Å)
1↔3	0.03	-	0.03	3.2
3↔4	0.09	0.04	0.07	2.9
4↔11	0.13	0.08	0.11	2.6
11↔12	0.17	0.07	0.12	2.6
12↔12’	1.00	-	1.00	1.8
12’↔14	0.13	0.06	0.10	2.7
12’↔23	0.03	0.02	0.03	3.3
12’↔15	-	0.04	0.04	3.1
14↔15	0.15	0.10	0.13	2.6
14↔16’	-	0.03	0.03	3.3
14↔19	0.08	0.05	0.07	2.9
15↔16	0.17	0.19	0.18	2.4
16↔16’	1.01	0.97	0.99	1.8
16’↔17	0.01	0.01	0.01	3.9
16’↔23	0.01	0.03	0.02	3.6
19↔20	0.07	0.12	0.10	2.7
20↔21’	0.07	0.04	0.06	2.9
20↔26	0.04	0.09	0.07	2.9
21↔21’	1.71	1.38	1.55	1.7
26↔27	0.10	0.11	0.11	2.6
26↔28	0.03	0.04	0.04	3.2
27↔28	0.12	0.16	0.14	2.5
30↔31	0.05	0.08	0.07	2.9
31↔34’	0.04	0.04	0.04	3.1
34↔34’	1.56	1.17	1.37	1.7

Indicated geminal protons (blue) were used as references distances where I_{ref} = 1.00 and r_{ref} = 1.8 Å.

^{1}H NMR (500 MHz, 5:1 $CDCl_3$:DMSO)

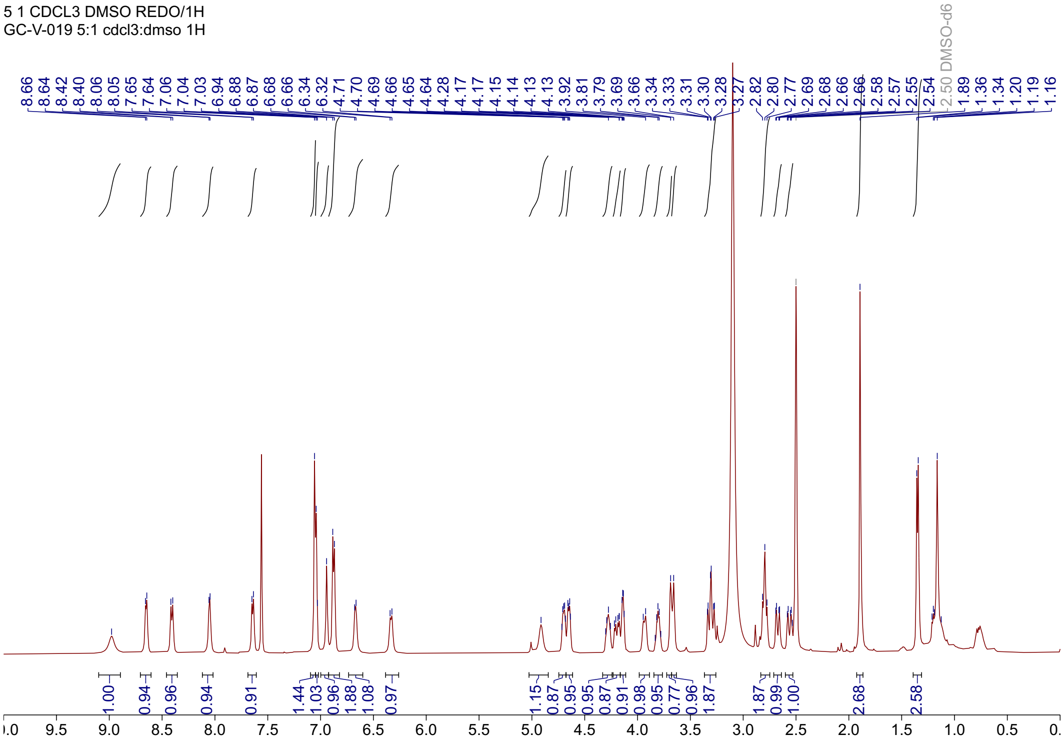

NOESY

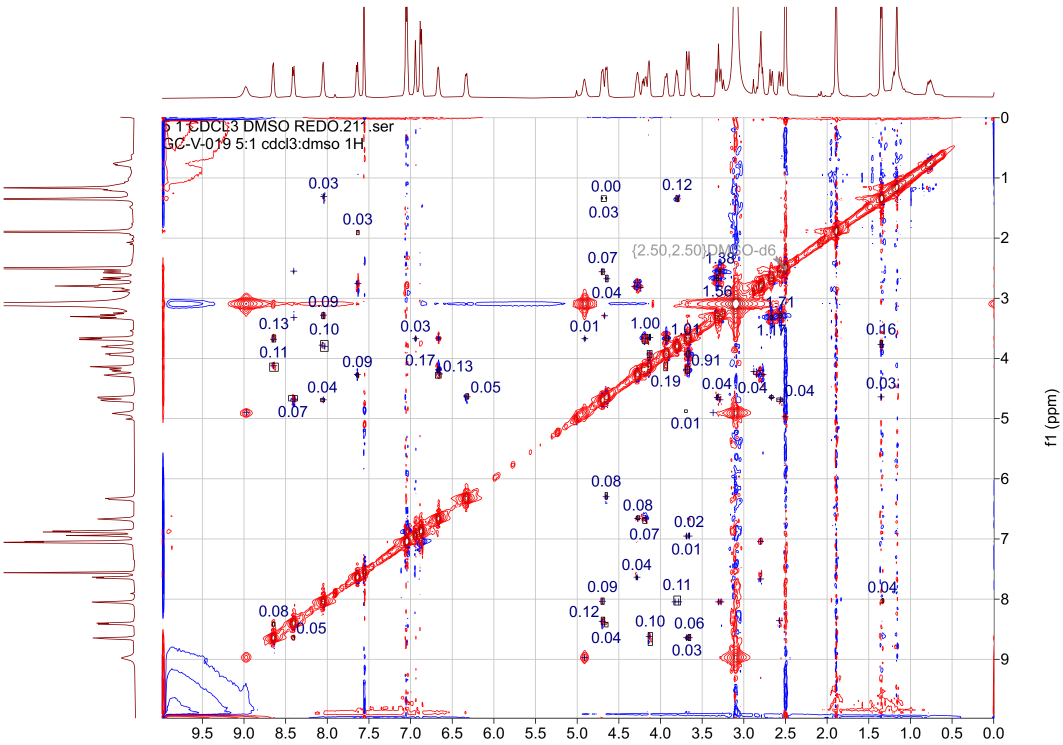

Polycycle 1b in 9:1 MeOD-d_3:D_2O

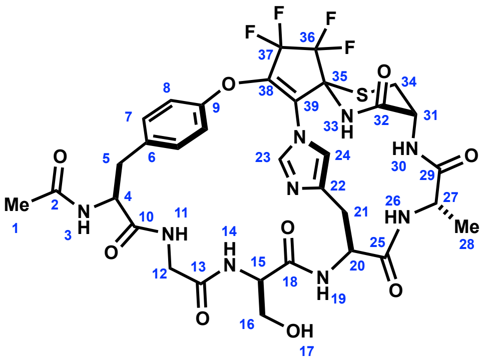

Chemical Formula: $C_{33}H_{35}F_4N_9O_9S$

^{1}H NMR (500 MHz, 9:1 MeOD:D_2O) δ 7.37 (s, 1H), 7.19 (d, *J* = 8.5 Hz, 2H), 7.06 – 6.91 (m, 3H), 4.80 (t, *J* = 3.0 Hz, 1H), 4.76 (t, *J* = 4.0 Hz, 1H), 4.50 (dd, *J* = 11.2, 4.1 Hz, 1H), 4.34 – 4.23 (m, 2H), 4.00 (q, *J* = 7.3 Hz, 1H), 3.92 (dd, *J* = 11.7, 5.7 Hz, 1H), 3.87 – 3.80 (m, 2H), 3.50 (dd, *J* = 14.7, 2.8 Hz, 1H), 3.35 (d, *J* = 4.5 Hz, 1H), 3.04 – 2.69 (m, 4H), 2.01 (s, 3H), 1.43 (d, *J* = 7.4 Hz, 3H).

Table S9. Tabulated chemical shifts and correlations for polycycle **1b** identified in 9:1 MeOD-*d*$_3$:D_2O.

	**^{1}H NMR**	**NOESY**
**1**	2.01 (s, 3H)	
**3**	Not observed	
**4**	4.50 (dd, *J* = 11.2, 4.1 Hz, 1H)	→ 5, 5’
**5**	3.04 – 2.69 (m, 4H, overlap)	→ 4
**5’**	3.04 – 2.69 (m, 4H, overlap)	→ 4
**7**	7.19 (d, *J* = 8.5 Hz, 2H)	→ 8
**8**	7.06 – 6.91 (m, 3H)	→ 7
**11**	Not observed	
**12**	4.34 – 4.23 (m, 2H, overlap)	→ 12’
**12’**	3.87 – 3.80 (m, 2H, overlap)	→ 12
**14**	Not observed	
**15**	4.34 – 4.23 (m, 2H, overlap)	→ 16, 16’
**16**	3.92 (dd, *J* = 11.7, 5.7 Hz, 1H)	→ 15
**16’**	3.87 – 3.80 (m, 2H, overlap)	→ 15
**17**	Not observed	
**19**	Not observed	
**20**	4.76 (t, *J* = 4.0 Hz, 1H)	→ 21, 21’
**21**	3.35 (d, *J* = 4.5 Hz, 1H)	→ 20, 21’
**21’**	3.04 – 2.69 (m, 4H, overlap)	→ 20, 21
**23**	7.06 – 6.91 (m, 3H)	
**24**	7.37 (s, 1H)	
**26**	Not observed	
**27**	4.00 (q, *J* = 7.3 Hz, 1H)	→ 28
**28**	1.43 (d, *J* = 7.4 Hz, 3H)	→ 27
**30**	Not observed	
**31**	4.80 (t, *J* = 3.0 Hz, 1H)	→ 34, 34’
**33**	Not observed	
**34**	3.50 (dd, *J* = 14.7, 2.8 Hz, 1H)	→ 31, 34’

34’	3.04 – 2.69 (m, 4H, overlap)	→ 31, 34

Table S10. Tabulated distance restraints for polycycle **1b** identified in 9:1 MeOD-d_3:D_2O.

NOE Correlation	I_{unk} (above diagonal)	I_{unk} (below diagonal)	I_{unk} (average)	r_{unk} (Å)
4↔5	0.17	0.45	0.31	2.2
4↔5’	0.21	0.14	0.18	2.4
20↔21	0.04	0.03	0.04	3.2
20↔21’	0.04	0.03	0.04	3.2
21↔21’	0.75	0.99	0.87	1.8
27↔28	0.29	0.11	0.20	2.4
31↔34’	0.02	0.03	0.03	3.3
34↔34’	1.00	-	-	1.8

Indicated geminal protons (blue) were used as references distances where I_{ref} = 1.00 and r_{ref} = 1.8 Å.

^{1}H NMR (500 MHz, 9:1 MeOD:D_2O)

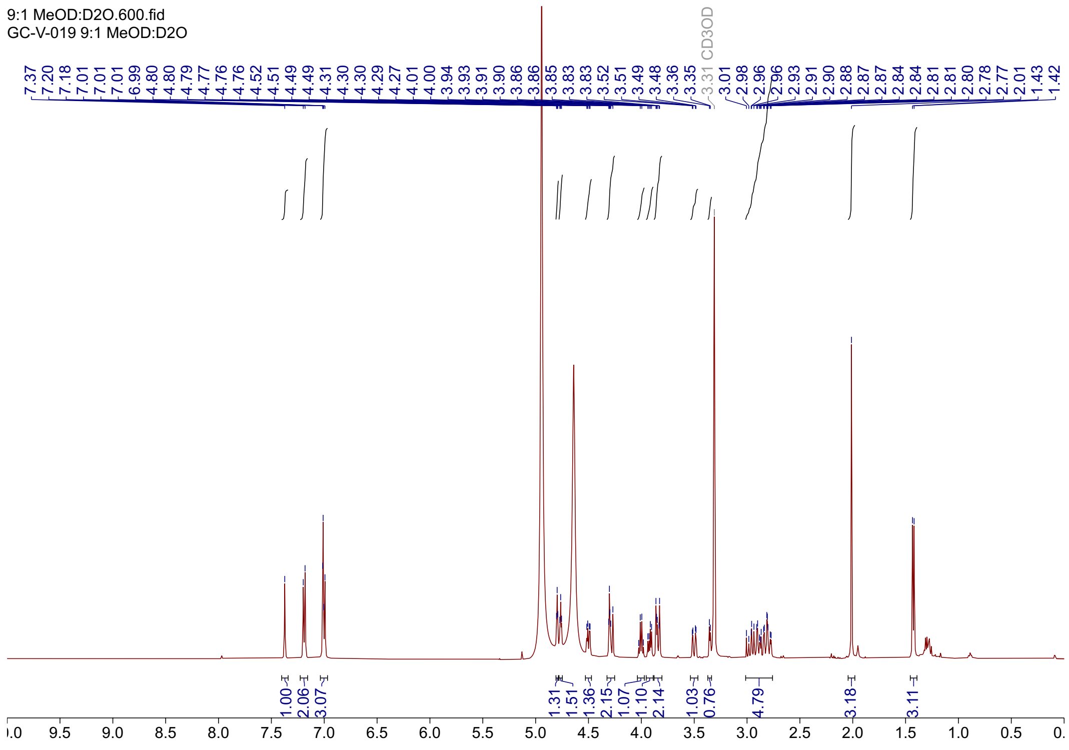

NOESY

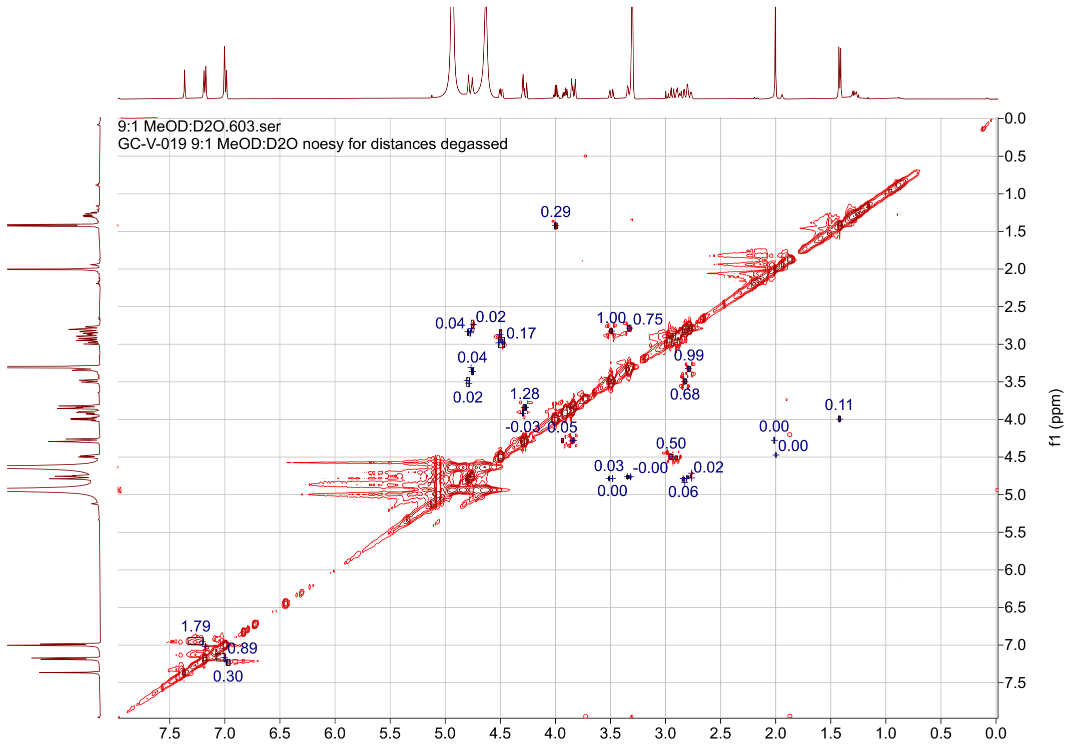

Crystallographic Data

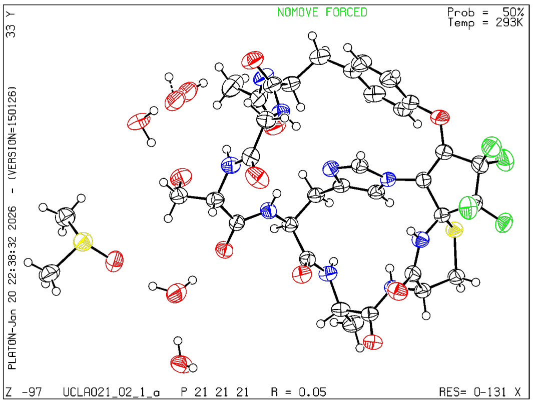

A colorless, block-shaped crystal was mounted on the goniometer. Data for **1a** were collected from a shock-cooled single crystal at 100K at the ALS 5.0.2 beamline. The diffractometer used synchrotron radiation (λ = 0.7748 Å). All data were integrated with XDS and a multi-scan absorption correction using was applied.[16] The structure was solved by direct methods with SHELXT 2018/2 and refined by full-matrix least-squares methods against F^2 using SHELXL-2018/3.[17, 18] All non-hydrogen atoms were refined with anisotropic displacement parameters. All C-bound hydrogen atoms were refined with isotropic displacement parameters. Some of their coordinates were refined freely and some on calculated positions using a riding model with their U_{iso} values constrained to 1.5 times the U_{eq} of their pivot atoms for terminal sp^3 carbon atoms and 1.2 times for all other carbon atoms. Disordered moieties were refined using bond lengths restraints and displacement parameter restraints. Crystallographic data for the structures reported in this paper have been deposited with the Cambridge Crystallographic Data Centre.[19] CCDC 2524526 contain the supplementary crystallographic data for this paper. These data can be obtained free of charge from The Cambridge Crystallographic Data Centre via www.ccdc.cam.ac.uk/structures. This report and the CIF file were generated using FinalCif (https://dkratzert.de/finalcif.html.).

Table S11. Crystal data and structure refinement for **1a**.

CCDC number	2524526
Empirical formula	$C_{140}H_{194.37}F_{16}N_{36}O_{55.19}S_8$
Formula weight	3825.14
Temperature [K]	293(2)
Crystal system	orthorhombic
Space group (number)	$P2_12_12_1$ (19)
a [Å]	13.270(3)
b [Å]	15.800(3)
c [Å]	20.680(4)
α [°]	90
β [°]	90
γ [°]	90
Volume [Å^3]	4335.9(15)
Z	1
ρ_{calc} [gcm^{-3}]	1.465
μ [mm^{-1}]	0.266
F(000)	2000
Crystal size [mm^3]	0.01×0.01×0.01
Crystal colour	colorless
Crystal shape	block
Radiation	synchrotron (λ=0.7748 Å)
2θ range [°]	3.54 to 58.02 (0.80 Å)
Index ranges	−16 ≤ h ≤ 16<br>−19 ≤ k ≤ 19<br>−25 ≤ l ≤ 25
Reflections collected	29896
Independent reflections	8511<br>R_{int} = 0.0625<br>R_{sigma} = 0.0574
Completeness to θ = 27.702°	96.7 %
Data / Restraints / Parameters	8511 / 10 / 617
Absorption correction T_{min}/T_{max} (method)	0.9950 / 0.9990 (multi-scan)
Goodness-of-fit on F^2	1.017
Final *R* indexes [$I \geq 2\sigma(I)$]	R_1 = 0.0549<br>wR_2 = 0.1510
Final *R* indexes [all data]	R_1 = 0.0640<br>wR_2 = 0.1602
Largest peak/hole [eÅ^{-3}]	0.41/−0.52
Flack X parameter	0.04(3)

Table S12. Atomic coordinates and U_{eq} [Å^2] for **1a**.

Atom	*x*	*y*	*z*	U_{eq}
S1	0.39264(8)	0.74431(8)	0.69523(5)	0.0401(3)
O2	0.5768(3)	0.2461(3)	0.6235(2)	0.0640(11)
O3	0.8997(3)	0.3146(3)	0.5417(2)	0.0610(10)
O4	0.7235(4)	0.5200(3)	0.3819(2)	0.0670(12)
O5	0.5807(3)	0.2286(3)	0.3923(2)	0.0555(10)
H5A	0.509(6)	0.212(4)	0.398(2)	0.083
O6	0.4131(2)	0.3758(2)	0.37806(17)	0.0461(8)
O7	0.3515(3)	0.5454(2)	0.44590(17)	0.0481(8)
O8	0.1591(3)	0.7080(2)	0.48951(16)	0.0463(8)
O9	0.3836(3)	0.8187(2)	0.49026(16)	0.0502(9)
N1	0.7456(3)	0.2363(3)	0.6161(2)	0.0520(11)
H1	0.796668	0.203225	0.612005	0.062
N2	0.7846(3)	0.4189(3)	0.52546(19)	0.0427(9)
H2	0.733180	0.443872	0.542097	0.051
N3	0.6816(3)	0.3877(3)	0.4097(2)	0.0447(10)
H3	0.697710	0.344868	0.433130	0.054
N4	0.5009(3)	0.4259(3)	0.46282(18)	0.0407(9)
H4	0.558342	0.445096	0.475240	0.049
N5	0.2649(3)	0.5081(3)	0.5342(2)	0.0408(9)
H5	0.264272	0.480408	0.569981	0.049
N6	0.2679(3)	0.6820(3)	0.57073(18)	0.0383(8)
H6	0.296971	0.644404	0.594348	0.046
N7	0.4760(3)	0.7918(3)	0.57855(19)	0.0405(9)
H7	0.527840	0.797232	0.553918	0.049
N8	0.6157(3)	0.5301(3)	0.55662(18)	0.0386(8)
N9	0.5765(3)	0.6374(2)	0.62042(18)	0.0357(8)
C1	0.5827(3)	0.7149(3)	0.6541(2)	0.0380(10)
C2	0.4991(3)	0.7797(3)	0.6460(2)	0.0387(10)
O1	0.7399(3)	0.7054(3)	0.7183(2)	0.0584(11)
F1	0.5965(3)	0.9045(2)	0.62763(15)	0.0573(8)
F2	0.4867(2)	0.9138(2)	0.70404(16)	0.0561(8)
F3	0.7164(2)	0.8770(2)	0.7201(2)	0.0681(10)
F4	0.5969(3)	0.8256(2)	0.78214(17)	0.0691(9)
C3	0.5510(4)	0.8595(3)	0.6752(2)	0.0427(11)
C4	0.6325(4)	0.8266(3)	0.7205(3)	0.0496(12)
C5	0.6563(3)	0.7402(3)	0.6954(2)	0.0439(11)
C6	0.7578(4)	0.6177(4)	0.7100(3)	0.0499(13)
C7	0.6901(4)	0.5576(4)	0.7305(2)	0.0480(12)
H7A	0.630241	0.573447	0.750431	0.058
C8	0.7123(4)	0.4750(4)	0.7210(2)	0.0495(13)
H8	0.665973	0.434201	0.734007	0.059
C9	0.8029(4)	0.4486(3)	0.6922(2)	0.0446(11)
C10	0.8715(4)	0.5102(4)	0.6739(3)	0.0532(13)
H10	0.932696	0.494219	0.655771	0.064
C11	0.8501(4)	0.5951(4)	0.6823(3)	0.0561(14)
H11	0.896203	0.636298	0.669825	0.067
C12	0.8188(4)	0.3563(3)	0.6783(3)	0.0498(13)
H12A	0.795397	0.323276	0.714929	0.060
H12B	0.890213	0.345498	0.672915	0.060
C13	0.7628(4)	0.3283(3)	0.6177(3)	0.0452(11)
H13	0.697190	0.356748	0.616783	0.054
C14	0.6533(4)	0.2019(4)	0.6206(3)	0.0550(14)
C15	0.6488(5)	0.1083(4)	0.6242(4)	0.0734(19)

H15A	0.598063	0.087801	0.595058	0.110
H15B	0.632417	0.091374	0.667537	0.110
H15C	0.713043	0.085066	0.612340	0.110
C16	0.8209(4)	0.3534(4)	0.5573(3)	0.0486(12)
C17	0.8230(4)	0.4532(4)	0.4648(2)	0.0463(11)
H17A	0.877582	0.417987	0.449050	0.056
H17B	0.849212	0.509752	0.471976	0.056
C18	0.7388(4)	0.4563(3)	0.4146(2)	0.0446(11)
C19	0.5936(4)	0.3788(3)	0.3679(2)	0.0432(11)
H19	0.599666	0.418542	0.331731	0.052
C20	0.5888(4)	0.2886(3)	0.3412(3)	0.0483(12)
H20A	0.531106	0.283307	0.312655	0.058
H20B	0.649078	0.277117	0.316103	0.058
C21	0.4944(4)	0.3954(3)	0.4031(2)	0.0396(10)
C22	0.4177(3)	0.4293(3)	0.5088(2)	0.0384(10)
H22	0.381016	0.375667	0.505619	0.046
C23	0.3435(4)	0.5001(3)	0.4938(2)	0.0391(10)
C24	0.1810(3)	0.5630(3)	0.5181(2)	0.0425(11)
H24	0.163728	0.552269	0.472782	0.051
C25	0.0880(4)	0.5410(4)	0.5586(3)	0.0509(13)
H25A	0.032421	0.575763	0.545304	0.076
H25B	0.102221	0.551009	0.603467	0.076
H25C	0.071184	0.482495	0.552379	0.076
C26	0.2040(3)	0.6572(3)	0.5242(2)	0.0390(10)
C27	0.2893(4)	0.7719(3)	0.5822(2)	0.0395(10)
H27	0.234780	0.804240	0.561920	0.047
C28	0.3862(4)	0.7959(3)	0.5474(2)	0.0406(10)
C29	0.2893(4)	0.7943(3)	0.6530(2)	0.0417(11)
H29A	0.226059	0.776544	0.672237	0.050
H29B	0.294498	0.855274	0.657704	0.050
C30	0.4641(3)	0.4338(3)	0.5773(2)	0.0392(10)
H30A	0.410468	0.430938	0.609079	0.047
H30B	0.507525	0.385142	0.583759	0.047
C31	0.5241(3)	0.5129(3)	0.5884(2)	0.0371(10)
C32	0.5001(3)	0.5782(3)	0.6276(2)	0.0380(10)
H32	0.443795	0.582626	0.654166	0.046
C33	0.6435(4)	0.6048(3)	0.5773(2)	0.0383(10)
H33	0.701955	0.632359	0.564034	0.046
O11	0.8732(4)	0.2600(4)	0.4102(3)	0.0689(15)
H11A	0.864(8)	0.254(6)	0.4559(9)	0.103
H11B	0.870(9)	0.273(6)	0.3650(10)	0.103
O12	0.7389(3)	0.1251(3)	0.3751(3)	0.0553(14)
H12C	0.676(2)	0.154(4)	0.380(4)	0.083
H12D	0.787(4)	0.168(3)	0.386(4)	0.083
O11B	0.9269(15)	0.3167(13)	0.3405(12)	0.0689(15)
H11C	0.876(5)	0.277(4)	0.353(8)	0.103
H11D	0.993(3)	0.295(7)	0.347(12)	0.103
O13	0.3822(4)	0.3879(3)	0.2412(2)	0.0800(14)
H13A	0.397(7)	0.334(2)	0.222(3)	0.120
H13B	0.387(7)	0.383(6)	0.2872(5)	0.120
O14	0.2188(3)	0.4607(3)	0.1848(2)	0.0662(11)
H14A	0.241(5)	0.5166(17)	0.174(4)	0.099
H14B	0.264(4)	0.424(3)	0.207(3)	0.099
S2	0.53345(10)	0.19151(9)	0.16079(7)	0.0533(4)
O10	0.4547(3)	0.2373(3)	0.2008(2)	0.0655(11)

C34	0.4714(5)	0.1640(5)	0.0887(3)	0.0634(16)
H34D	0.451795	0.214491	0.066185	0.095
H34E	0.412651	0.131068	0.098640	0.095
H34F	0.516027	0.131403	0.061949	0.095
C35	0.5433(5)	0.0883(4)	0.1941(3)	0.0559(13)
H35A	0.477260	0.064204	0.198550	0.084
H35B	0.574896	0.091413	0.235822	0.084
H35C	0.583179	0.053362	0.166014	0.084

U_{eq} is defined as 1/3 of the trace of the orthogonalized U_{ij} tensor.

Table S13. Anisotropic displacement parameters [Å^2] for **1a**. The anisotropic displacement factor exponent takes the form: $-2\pi^2[\, h^2(a^*)^2U_{11} + k^2(b^*)^2U_{22} + \ldots + 2hka^*b^*U_{12}\,]$.

**Atom**	U_{11}	U_{22}	U_{33}	U_{23}	U_{13}	U_{12}
S1	0.0332(5)	0.0485(7)	0.0385(5)	0.0005(5)	0.0018(4)	0.0028(5)
O2	0.044(2)	0.059(3)	0.089(3)	−0.002(2)	−0.001(2)	0.007(2)
O3	0.047(2)	0.072(3)	0.064(2)	0.007(2)	0.0077(18)	0.018(2)
O4	0.082(3)	0.062(3)	0.058(2)	0.0177(19)	−0.014(2)	−0.020(2)
O5	0.0439(19)	0.056(2)	0.067(2)	0.0087(18)	0.0058(17)	0.0012(18)
O6	0.0363(17)	0.050(2)	0.0518(19)	−0.0063(16)	−0.0031(15)	−0.0052(15)
O7	0.0452(18)	0.052(2)	0.0465(19)	0.0087(16)	0.0044(15)	0.0012(17)
O8	0.0392(17)	0.052(2)	0.0482(18)	0.0049(15)	−0.0093(15)	0.0027(16)
O9	0.0476(18)	0.063(2)	0.0406(17)	0.0076(16)	0.0016(15)	−0.0034(19)
N1	0.035(2)	0.045(3)	0.076(3)	0.003(2)	0.005(2)	0.0063(19)
N2	0.0334(19)	0.050(2)	0.045(2)	0.0036(18)	0.0041(16)	0.0040(19)
N3	0.038(2)	0.046(2)	0.051(2)	0.0063(18)	−0.0002(18)	−0.0028(19)
N4	0.0308(18)	0.049(2)	0.042(2)	−0.0066(17)	0.0016(16)	−0.0051(18)
N5	0.034(2)	0.046(2)	0.042(2)	0.0069(17)	0.0009(16)	0.0035(18)
N6	0.0323(18)	0.043(2)	0.039(2)	0.0016(16)	−0.0033(15)	0.0041(17)
N7	0.0316(18)	0.051(2)	0.0387(19)	0.0015(17)	0.0047(15)	0.0008(17)
N8	0.0335(18)	0.041(2)	0.0410(19)	0.0010(16)	0.0035(16)	0.0013(18)
N9	0.0302(17)	0.038(2)	0.0389(19)	−0.0023(15)	−0.0009(15)	0.0020(16)
C1	0.033(2)	0.041(3)	0.040(2)	−0.0044(19)	0.0014(18)	−0.0012(19)
C2	0.032(2)	0.041(3)	0.043(2)	−0.0026(19)	−0.0019(19)	0.003(2)
O1	0.0390(18)	0.057(2)	0.079(3)	−0.016(2)	−0.0222(18)	0.0072(17)
F1	0.065(2)	0.0507(18)	0.0562(17)	−0.0042(14)	0.0109(15)	−0.0158(16)
F2	0.0435(16)	0.0573(19)	0.0675(19)	−0.0170(15)	0.0088(14)	0.0043(14)
F3	0.0447(17)	0.061(2)	0.098(3)	−0.0243(19)	−0.0073(18)	−0.0096(16)
F4	0.073(2)	0.084(2)	0.0502(18)	−0.0149(17)	0.0001(16)	0.002(2)
C3	0.038(2)	0.042(3)	0.048(3)	−0.008(2)	0.008(2)	−0.001(2)
C4	0.039(3)	0.053(3)	0.057(3)	−0.012(2)	−0.005(2)	−0.006(2)
C5	0.034(2)	0.048(3)	0.050(3)	−0.004(2)	−0.0059(19)	0.000(2)
C6	0.038(3)	0.054(3)	0.057(3)	−0.012(2)	−0.014(2)	0.009(2)
C7	0.037(2)	0.068(4)	0.038(2)	0.006(2)	−0.0026(19)	0.010(3)
C8	0.039(2)	0.065(4)	0.045(3)	0.018(2)	0.004(2)	0.001(3)
C9	0.036(2)	0.052(3)	0.045(2)	0.006(2)	−0.003(2)	0.002(2)
C10	0.032(2)	0.061(3)	0.067(3)	0.001(3)	0.003(2)	0.000(2)
C11	0.036(2)	0.054(3)	0.078(4)	0.000(3)	−0.007(2)	−0.004(3)
C12	0.039(3)	0.057(3)	0.054(3)	0.010(2)	−0.001(2)	0.007(2)
C13	0.033(2)	0.049(3)	0.053(3)	0.008(2)	0.003(2)	0.005(2)
C14	0.046(3)	0.047(3)	0.072(4)	−0.007(3)	0.003(3)	0.005(3)
C15	0.051(3)	0.052(4)	0.118(6)	−0.014(4)	0.009(4)	−0.001(3)
C16	0.040(3)	0.053(3)	0.053(3)	0.002(2)	0.001(2)	0.009(2)
C17	0.037(2)	0.050(3)	0.052(3)	0.004(2)	0.000(2)	−0.007(2)
C18	0.046(3)	0.044(3)	0.044(3)	0.004(2)	0.007(2)	−0.003(2)
C19	0.037(2)	0.050(3)	0.043(2)	0.004(2)	0.0010(19)	0.002(2)
C20	0.041(3)	0.058(3)	0.047(3)	−0.003(2)	−0.001(2)	0.004(2)
C21	0.036(2)	0.037(3)	0.046(2)	−0.0053(19)	−0.001(2)	0.001(2)

C22	0.034(2)	0.039(3)	0.042(2)	−0.0007(19)	−0.0016(18)	0.000(2)
C23	0.035(2)	0.041(3)	0.041(2)	−0.0026(19)	0.0006(19)	−0.004(2)
C24	0.030(2)	0.049(3)	0.049(3)	0.005(2)	−0.0040(19)	0.001(2)
C25	0.034(2)	0.051(3)	0.068(3)	0.009(3)	0.001(2)	−0.002(2)
C26	0.030(2)	0.044(3)	0.043(2)	0.003(2)	0.0000(18)	0.002(2)
C27	0.033(2)	0.044(3)	0.041(2)	0.000(2)	−0.0009(18)	0.004(2)
C28	0.043(2)	0.041(3)	0.038(2)	0.0003(18)	−0.002(2)	0.001(2)
C29	0.033(2)	0.050(3)	0.042(2)	−0.002(2)	0.0002(19)	0.002(2)
C30	0.032(2)	0.040(3)	0.045(2)	0.0014(19)	−0.0025(19)	0.001(2)
C31	0.028(2)	0.039(3)	0.044(2)	0.0033(19)	−0.0014(18)	0.0012(19)
C32	0.0273(19)	0.047(3)	0.040(2)	−0.0003(19)	−0.0010(17)	0.001(2)
C33	0.032(2)	0.043(3)	0.039(2)	0.0017(19)	−0.0005(18)	−0.001(2)
O11	0.061(3)	0.061(3)	0.085(4)	−0.008(3)	0.002(3)	0.017(3)
O12	0.042(3)	0.051(3)	0.073(3)	0.018(2)	0.006(2)	0.013(2)
O11B	0.061(3)	0.061(3)	0.085(4)	−0.008(3)	0.002(3)	0.017(3)
O13	0.093(3)	0.086(3)	0.062(3)	−0.011(2)	−0.014(3)	0.041(3)
O14	0.049(2)	0.082(3)	0.067(3)	−0.004(2)	−0.0145(19)	0.001(2)
S2	0.0472(7)	0.0502(8)	0.0626(8)	−0.0015(6)	−0.0006(6)	−0.0021(6)
O10	0.064(2)	0.060(3)	0.072(3)	−0.019(2)	0.001(2)	0.008(2)
C34	0.051(3)	0.075(4)	0.064(4)	−0.001(3)	0.002(3)	0.021(3)
C35	0.055(3)	0.060(3)	0.054(3)	0.003(3)	0.002(3)	0.005(3)

Table S14. Bond lengths and angles for **1a**.

Atom–Atom	Length [Å]
S1–C29	1.808(5)
S1–C2	1.829(5)
O2–C14	1.233(7)
O3–C16	1.255(6)
O4–C18	1.229(6)
O5–C20	1.425(7)
O5–H5A	0.99(8)
O6–C21	1.237(6)
O7–C23	1.227(6)
O8–C26	1.232(6)
O9–C28	1.236(6)
N1–C14	1.344(7)
N1–C13	1.472(7)
N1–H1	0.8600
N2–C16	1.318(7)
N2–C17	1.458(6)
N2–H2	0.8600
N3–C18	1.328(7)
N3–C19	1.459(6)
N3–H3	0.8600
N4–C21	1.328(6)
N4–C22	1.458(6)
N4–H4	0.8600
N5–C23	1.343(6)
N5–C24	1.450(6)
N5–H5	0.8600
N6–C26	1.341(6)
N6–C27	1.469(6)
N6–H6	0.8600
N7–C28	1.357(6)
N7–C2	1.440(6)
N7–H7	0.8600
N8–C33	1.309(6)
N8–C31	1.409(6)
N9–C33	1.361(6)
N9–C32	1.387(6)
N9–C1	1.412(6)
C1–C5	1.357(6)
C1–C2	1.518(7)
C2–C3	1.559(7)
O1–C5	1.326(6)
O1–C6	1.417(7)
F1–C3	1.356(6)
F2–C3	1.348(6)
F3–C4	1.368(6)
F4–C4	1.359(6)
C3–C4	1.522(7)
C4–C5	1.495(7)
C6–C7	1.374(8)
C6–C11	1.398(8)
C7–C8	1.353(8)
C7–H7A	0.9300
C8–C9	1.406(7)
C8–H8	0.9300
C9–C10	1.384(7)
C9–C12	1.501(8)
C10–C11	1.381(9)
C10–H10	0.9300
C11–H11	0.9300
C12–C13	1.524(7)
C12–H12A	0.9700
C12–H12B	0.9700
C13–C16	1.520(7)
C13–H13	0.9800
C14–C15	1.481(8)
C15–H15A	0.9600
C15–H15B	0.9600
C15–H15C	0.9600
C17–C18	1.526(7)

C17–H17A	0.9700
C17–H17B	0.9700
C19–C21	1.528(7)
C19–C20	1.529(8)
C19–H19	0.9800
C20–H20A	0.9700
C20–H20B	0.9700
C22–C23	1.523(7)
C22–C30	1.546(6)
C22–H22	0.9800
C24–C26	1.524(7)
C24–C25	1.531(7)
C24–H24	0.9800
C25–H25A	0.9600
C25–H25B	0.9600
C25–H25C	0.9600
C27–C29	1.507(7)
C27–C28	1.520(7)
C27–H27	0.9800
C29–H29A	0.9700
C29–H29B	0.9700
C30–C31	1.499(7)
C30–H30A	0.9700
C30–H30B	0.9700
C31–C32	1.351(7)
C32–H32	0.9300
C33–H33	0.9300
O11–H11A	0.9571(15)
O11–H11B	0.9571(15)
O12–H12C	0.9570(15)
O12–H12D	0.9571(15)
O11B–H11C	0.9570(15)
O11B–H11D	0.9570(17)
O13–H13A	0.9570(15)
O13–H13B	0.9570(15)
O14–H14A	0.9570(15)
O14–H14B	0.9570(15)
S2–O10	1.517(4)
S2–C34	1.757(6)
S2–C35	1.776(6)
C34–H34D	0.9600
C34–H34E	0.9600
C34–H34F	0.9600
C35–H35A	0.9600
C35–H35B	0.9600
C35–H35C	0.9600
**Atom–Atom–Atom**	**Angle [°]**
C29–S1–C2	100.6(2)
C20–O5–H5A	109.5
C14–N1–C13	122.7(4)
C14–N1–H1	118.7
C13–N1–H1	118.7
C16–N2–C17	126.4(4)
C16–N2–H2	116.8
C17–N2–H2	116.8
C18–N3–C19	125.6(4)
C18–N3–H3	117.2

C19–N3–H3	117.2
C21–N4–C22	124.8(4)
C21–N4–H4	117.6
C22–N4–H4	117.6
C23–N5–C24	120.7(4)
C23–N5–H5	119.7
C24–N5–H5	119.7
C26–N6–C27	121.4(4)
C26–N6–H6	119.3
C27–N6–H6	119.3
C28–N7–C2	130.7(4)
C28–N7–H7	114.6
C2–N7–H7	114.6
C33–N8–C31	105.4(4)
C33–N9–C32	107.1(4)
C33–N9–C1	127.9(4)
C32–N9–C1	125.0(4)
C5–C1–N9	127.4(4)
C5–C1–C2	113.4(4)
N9–C1–C2	119.2(4)
N7–C2–C1	110.6(4)
N7–C2–C3	111.3(4)
C1–C2–C3	100.3(4)
N7–C2–S1	114.5(3)
C1–C2–S1	107.2(3)
C3–C2–S1	111.9(3)
C5–O1–C6	120.2(4)
F2–C3–F1	105.6(4)
F2–C3–C4	113.3(4)
F1–C3–C4	108.1(4)
F2–C3–C2	113.9(4)
F1–C3–C2	109.8(4)
C4–C3–C2	106.1(4)
F4–C4–F3	107.2(4)
F4–C4–C5	112.8(5)
F3–C4–C5	111.0(4)
F4–C4–C3	109.5(4)
F3–C4–C3	112.1(4)
C5–C4–C3	104.4(4)
O1–C5–C1	134.8(5)
O1–C5–C4	115.5(4)
C1–C5–C4	109.6(4)
C7–C6–C11	121.5(5)
C7–C6–O1	121.9(5)
C11–C6–O1	116.6(5)
C8–C7–C6	118.7(5)
C8–C7–H7A	120.6
C6–C7–H7A	120.6
C7–C8–C9	122.2(5)
C7–C8–H8	118.9
C9–C8–H8	118.9
C10–C9–C8	118.0(5)
C10–C9–C12	122.6(5)
C8–C9–C12	119.2(5)
C11–C10–C9	120.9(5)
C11–C10–H10	119.6
C9–C10–H10	119.6
C10–C11–C6	118.6(5)

C10–C11–H11	120.7
C6–C11–H11	120.7
C9–C12–C13	111.8(4)
C9–C12–H12A	109.3
C13–C12–H12A	109.3
C9–C12–H12B	109.3
C13–C12–H12B	109.3
H12A–C12–H12B	107.9
N1–C13–C16	108.5(4)
N1–C13–C12	112.4(4)
C16–C13–C12	110.7(4)
N1–C13–H13	108.4
C16–C13–H13	108.4
C12–C13–H13	108.4
O2–C14–N1	121.6(5)
O2–C14–C15	122.0(5)
N1–C14–C15	116.3(5)
C14–C15–H15A	109.5
C14–C15–H15B	109.5
H15A–C15–H15B	109.5
C14–C15–H15C	109.5
H15A–C15–H15C	109.5
H15B–C15–H15C	109.5
O3–C16–N2	124.0(5)
O3–C16–C13	120.5(5)
N2–C16–C13	115.5(4)
N2–C17–C18	110.0(4)
N2–C17–H17A	109.7
C18–C17–H17A	109.7
N2–C17–H17B	109.7
C18–C17–H17B	109.7
H17A–C17–H17B	108.2
O4–C18–N3	122.1(5)
O4–C18–C17	121.5(5)
N3–C18–C17	116.4(4)
N3–C19–C21	113.0(4)
N3–C19–C20	109.7(4)
C21–C19–C20	107.3(4)
N3–C19–H19	108.9
C21–C19–H19	108.9
C20–C19–H19	108.9
O5–C20–C19	110.8(4)
O5–C20–H20A	109.5
C19–C20–H20A	109.5
O5–C20–H20B	109.5
C19–C20–H20B	109.5
H20A–C20–H20B	108.1
O6–C21–N4	122.5(4)
O6–C21–C19	120.6(4)
N4–C21–C19	116.7(4)
N4–C22–C23	112.5(4)
N4–C22–C30	107.4(4)
C23–C22–C30	114.3(4)
N4–C22–H22	107.5
C23–C22–H22	107.5
C30–C22–H22	107.5
O7–C23–N5	121.0(4)
O7–C23–C22	122.5(4)

N5–C23–C22	116.4(4)
N5–C24–C26	114.3(4)
N5–C24–C25	111.0(4)
C26–C24–C25	109.7(4)
N5–C24–H24	107.2
C26–C24–H24	107.2
C25–C24–H24	107.2
C24–C25–H25A	109.5
C24–C25–H25B	109.5
H25A–C25–H25B	109.5
C24–C25–H25C	109.5
H25A–C25–H25C	109.5
H25B–C25–H25C	109.5
O8–C26–N6	122.2(5)
O8–C26–C24	119.5(4)
N6–C26–C24	118.1(4)
N6–C27–C29	112.6(4)
N6–C27–C28	109.2(4)
C29–C27–C28	113.7(4)
N6–C27–H27	107.0
C29–C27–H27	107.0
C28–C27–H27	107.0
O9–C28–N7	119.5(4)
O9–C28–C27	120.1(4)
N7–C28–C27	120.4(4)
C27–C29–S1	111.5(3)
C27–C29–H29A	109.3
S1–C29–H29A	109.3
C27–C29–H29B	109.3
S1–C29–H29B	109.3
H29A–C29–H29B	108.0
C31–C30–C22	112.9(4)
C31–C30–H30A	109.0
C22–C30–H30A	109.0
C31–C30–H30B	109.0
C22–C30–H30B	109.0
H30A–C30–H30B	107.8
C32–C31–N8	109.6(4)
C32–C31–C30	127.2(4)
N8–C31–C30	123.2(4)
C31–C32–N9	106.2(4)
C31–C32–H32	126.9
N9–C32–H32	126.9
N8–C33–N9	111.7(4)
N8–C33–H33	124.1
N9–C33–H33	124.1
H11A–O11–H11B	168(9)
H12C–O12–H12D	103(5)
H11C–O11B–H11D	112(9)
H13A–O13–H13B	108(7)
H14A–O14–H14B	118(6)
O10–S2–C34	105.0(3)
O10–S2–C35	106.1(3)
C34–S2–C35	97.9(3)
S2–C34–H34D	109.5
S2–C34–H34E	109.5
H34D–C34–H34E	109.5
S2–C34–H34F	109.5

H34D–C34–H34F	109.5
H34E–C34–H34F	109.5
S2–C35–H35A	109.5
S2–C35–H35B	109.5
H35A–C35–H35B	109.5
S2–C35–H35C	109.5
H35A–C35–H35C	109.5
H35B–C35–H35C	109.5

Table S15. Torsion angles for **1a**.

**Atom–Atom–Atom–Atom**	**Torsion Angle [°]**
C33–N9–C1–C5	−59.4(7)
C32–N9–C1–C5	118.2(5)
C33–N9–C1–C2	121.4(5)
C32–N9–C1–C2	−60.9(6)
C28–N7–C2–C1	134.5(5)
C28–N7–C2–C3	−114.8(6)
C28–N7–C2–S1	13.2(7)
C5–C1–C2–N7	132.8(4)
N9–C1–C2–N7	−48.0(6)
C5–C1–C2–C3	15.2(5)
N9–C1–C2–C3	−165.5(4)
C5–C1–C2–S1	−101.7(4)
N9–C1–C2–S1	77.6(5)
C29–S1–C2–N7	−31.5(4)
C29–S1–C2–C1	−154.6(3)
C29–S1–C2–C3	96.3(4)
N7–C2–C3–F2	94.1(5)
C1–C2–C3–F2	−148.8(4)
S1–C2–C3–F2	−35.4(5)
N7–C2–C3–F1	−24.1(5)
C1–C2–C3–F1	93.0(4)
S1–C2–C3–F1	−153.6(3)
N7–C2–C3–C4	−140.6(4)
C1–C2–C3–C4	−23.5(5)
S1–C2–C3–C4	89.9(4)
F2–C3–C4–F4	28.9(6)
F1–C3–C4–F4	145.5(4)
C2–C3–C4–F4	−96.8(5)
F2–C3–C4–F3	−90.0(5)
F1–C3–C4–F3	26.6(6)
C2–C3–C4–F3	144.3(4)
F2–C3–C4–C5	149.9(4)
F1–C3–C4–C5	−93.5(5)
C2–C3–C4–C5	24.2(5)
C6–O1–C5–C1	−12.8(9)
C6–O1–C5–C4	165.4(5)
N9–C1–C5–O1	−1.4(9)
C2–C1–C5–O1	177.8(6)
N9–C1–C5–C4	−179.7(5)
C2–C1–C5–C4	−0.5(6)
F4–C4–C5–O1	−74.9(6)
F3–C4–C5–O1	45.3(6)
C3–C4–C5–O1	166.3(4)
F4–C4–C5–C1	103.8(5)
F3–C4–C5–C1	−136.0(5)
C3–C4–C5–C1	−15.1(6)
C5–O1–C6–C7	−55.4(7)
C5–O1–C6–C11	127.2(5)
C11–C6–C7–C8	−2.7(8)
O1–C6–C7–C8	−179.9(5)
C6–C7–C8–C9	1.3(8)
C7–C8–C9–C10	0.9(8)
C7–C8–C9–C12	−175.1(5)
C8–C9–C10–C11	−1.7(8)
C12–C9–C10–C11	174.2(5)
C9–C10–C11–C6	0.3(9)
C7–C6–C11–C10	1.9(9)
O1–C6–C11–C10	179.3(5)
C10–C9–C12–C13	−97.4(6)
C8–C9–C12–C13	78.4(6)
C14–N1–C13–C16	−124.7(6)
C14–N1–C13–C12	112.5(6)
C9–C12–C13–N1	−158.9(4)
C9–C12–C13–C16	79.6(5)
C13–N1–C14–O2	3.4(9)
C13–N1–C14–C15	−174.7(5)
C17–N2–C16–O3	5.5(9)
C17–N2–C16–C13	−177.0(5)
N1–C13–C16–O3	−50.5(6)
C12–C13–C16–O3	73.3(7)
N1–C13–C16–N2	131.9(5)
C12–C13–C16–N2	−104.3(5)
C16–N2–C17–C18	124.7(5)
C19–N3–C18–O4	−2.0(8)
C19–N3–C18–C17	176.1(4)
N2–C17–C18–O4	132.6(5)
N2–C17–C18–N3	−45.6(6)
C18–N3–C19–C21	−96.8(6)
C18–N3–C19–C20	143.5(5)
N3–C19–C20–O5	60.4(5)
C21–C19–C20–O5	−62.7(5)
C22–N4–C21–O6	8.9(8)
C22–N4–C21–C19	−166.5(4)
N3–C19–C21–O6	−166.9(5)
C20–C19–C21–O6	−45.9(6)
N3–C19–C21–N4	8.6(7)
C20–C19–C21–N4	129.6(5)
C21–N4–C22–C23	−76.0(6)
C21–N4–C22–C30	157.5(4)
C24–N5–C23–O7	7.8(7)
C24–N5–C23–C22	−169.0(4)
N4–C22–C23–O7	3.7(6)
C30–C22–C23–O7	126.5(5)
N4–C22–C23–N5	−179.5(4)
C30–C22–C23–N5	−56.8(6)
C23–N5–C24–C26	−73.6(6)

C23–N5–C24–C25	161.7(5)
C27–N6–C26–O8	−1.0(7)
C27–N6–C26–C24	−176.4(4)
N5–C24–C26–O8	150.9(4)
C25–C24–C26–O8	−83.8(5)
N5–C24–C26–N6	−33.6(6)
C25–C24–C26–N6	91.7(5)
C26–N6–C27–C29	135.8(4)
C26–N6–C27–C28	−97.0(5)
C2–N7–C28–O9	169.7(5)
C2–N7–C28–C27	−10.9(8)
N6–C27–C28–O9	87.0(5)
C29–C27–C28–O9	−146.4(5)
N6–C27–C28–N7	−92.4(5)
C29–C27–C28–N7	34.3(6)
N6–C27–C29–S1	66.8(5)
C28–C27–C29–S1	−58.1(5)
C2–S1–C29–C27	54.1(4)
N4–C22–C30–C31	63.9(5)
C23–C22–C30–C31	−61.7(5)
C33–N8–C31–C32	−0.4(5)
C33–N8–C31–C30	178.4(4)
C22–C30–C31–C32	110.5(5)
C22–C30–C31–N8	−68.1(6)
N8–C31–C32–N9	0.4(5)
C30–C31–C32–N9	−178.3(4)
C33–N9–C32–C31	−0.3(5)
C1–N9–C32–C31	−178.3(4)
C31–N8–C33–N9	0.2(5)
C32–N9–C33–N8	0.0(5)
C1–N9–C33–N8	178.0(4)

Spectroscopic Data

LC-HRMS Spectra

Polycycle 1b. **HRMS** (QTOF) m/z: $[M+H]^+$ calc'd for $C_{33}H_{36}F_4N_9O_9S^+$ 810.2287, found 810.2289; 0.2 ppm mass defect.

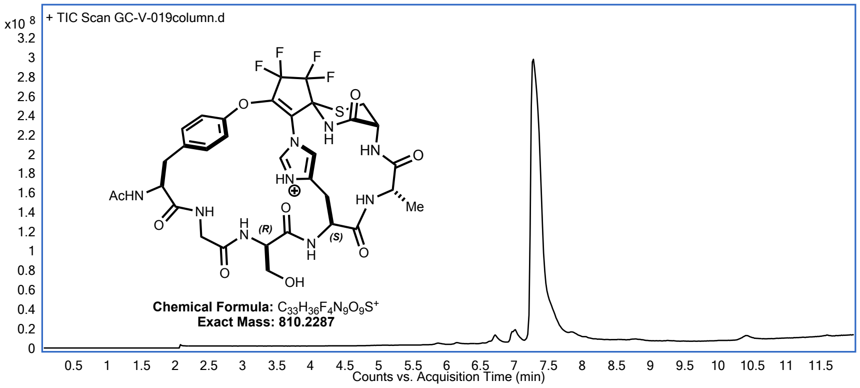

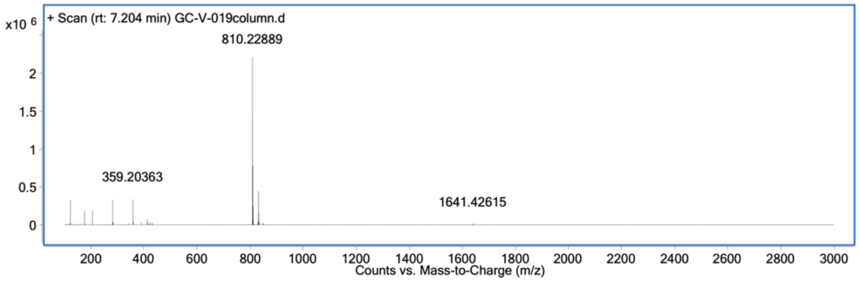

Polycycle 1c. **HRMS** (QTOF) m/z: $[M+H]^+$ calc'd for $C_{33}H_{36}F_4N_9O_9S^+$ 810.2287, found 810.2327; 4.9 ppm mass defect.

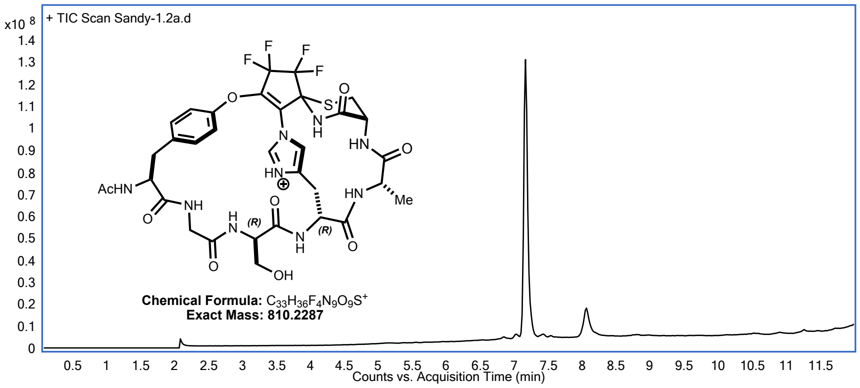

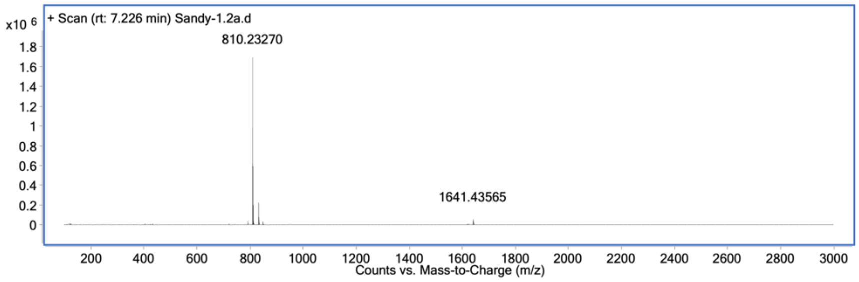

Polycycle 1d. **HRMS** (QTOF) m/z: $[M+H]^+$ calc'd for $C_{34}H_{38}F_4N_9O_9S^+$ 824.2444, found 824.2349; 11.5 ppm mass defect.

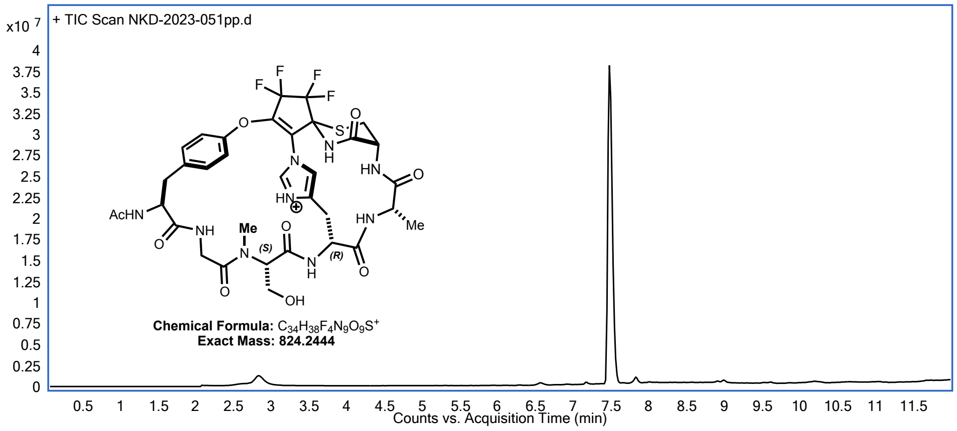

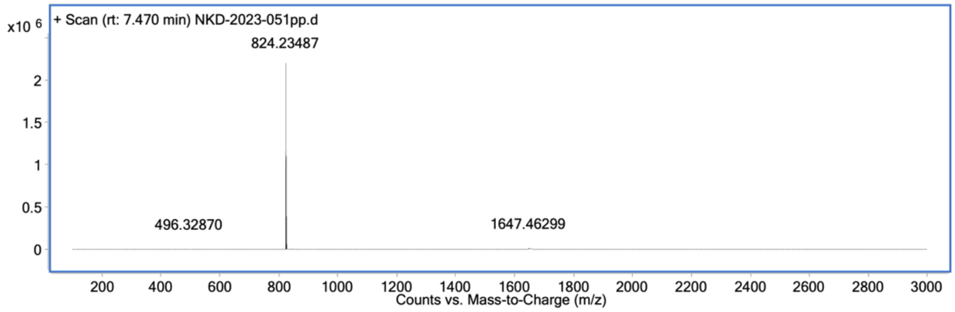

Polycycle 1e. **HRMS** (QTOF) m/z: $[M+H]^+$ calc'd for $C_{34}H_{38}F_4N_9O_9S^+$ 824.2444, found 824.2498; 6.6 ppm mass defect.

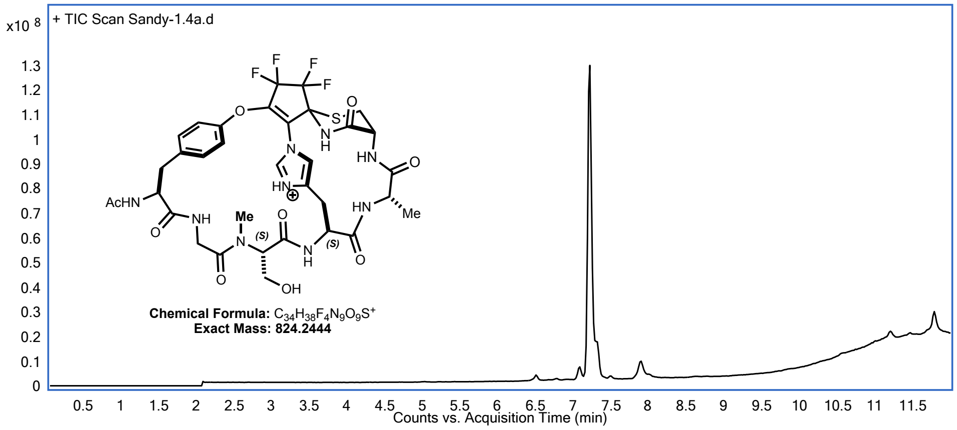

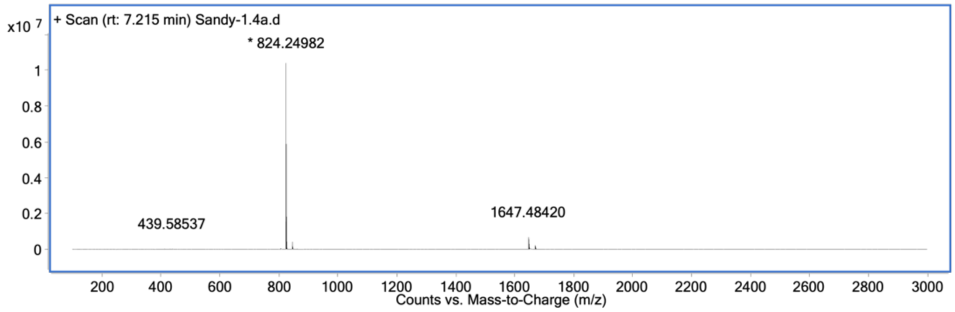

Polycycle 1f. **HRMS** (QTOF) m/z: [M+H]$^+$ calc'd for $C_{31}H_{33}F_4N_8O_8S^+$ 753.2073, found 753.2075; 0.3 ppm mass defect.

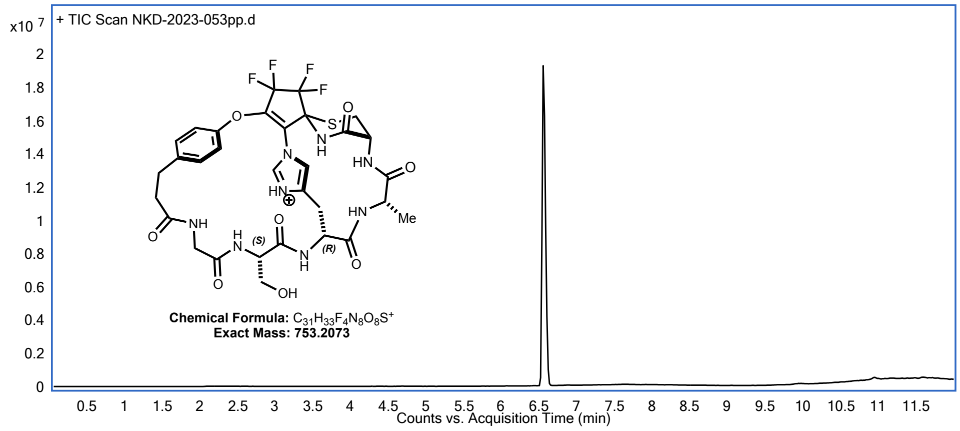

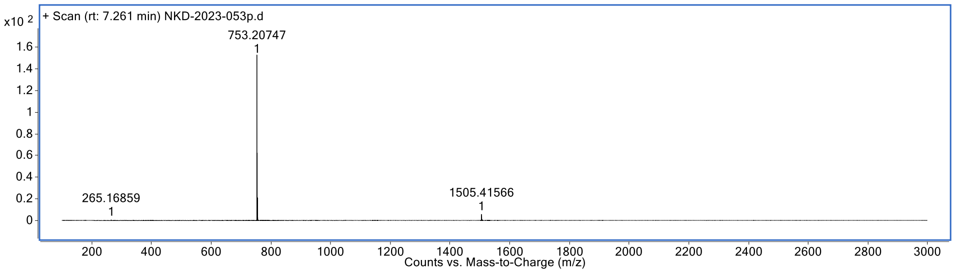

Polycycle 1g. **HRMS** (QTOF) m/z: [M+H]$^+$ calc'd for $C_{31}H_{33}F_4N_8O_8S^+$ 753.2073, found 753.2075; 0.3 ppm mass defect.

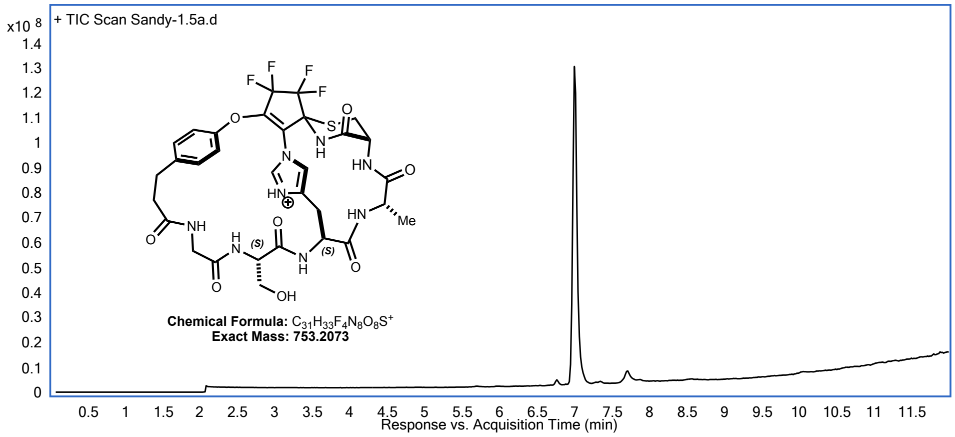

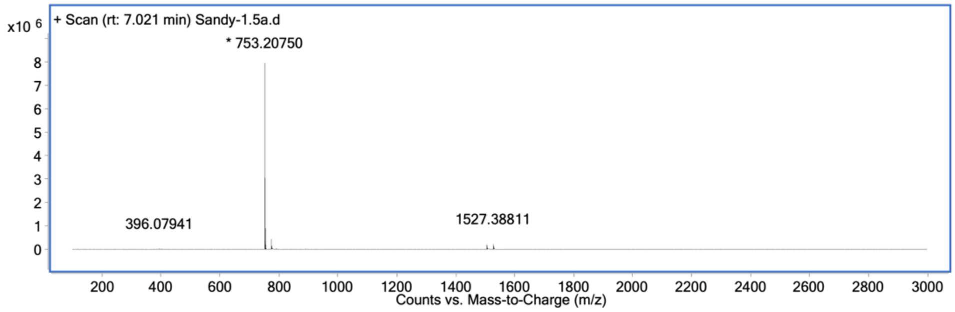

Polycycle 1h. **HRMS** (QTOF) m/z: $[M+H]^+$ calc'd for $C_{32}H_{35}F_4N_8O_9S^+$ 783.2178, found 783.2229; 6.5 ppm mass defect.

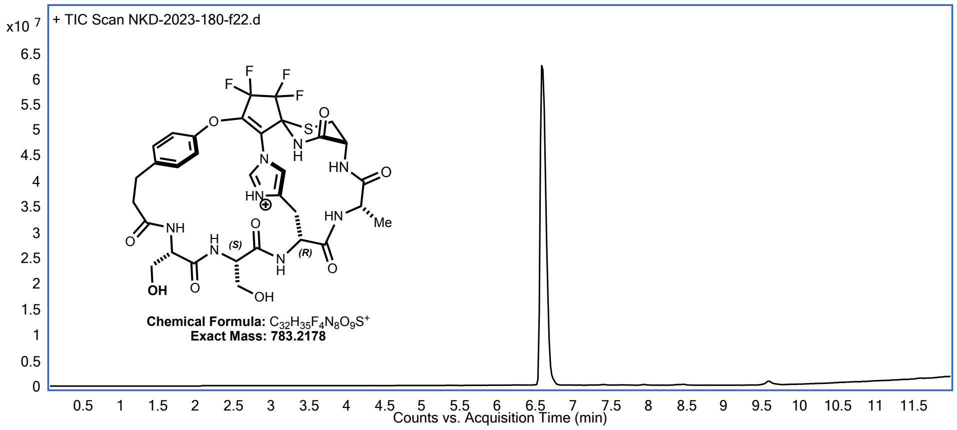

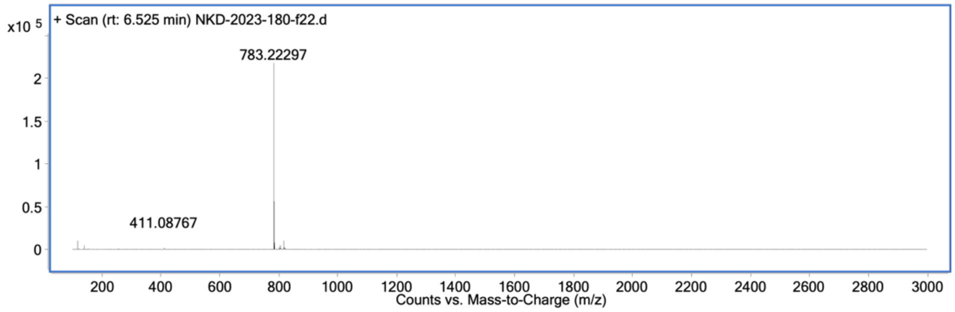

Polycycle 1i. **HRMS** (QTOF) m/z: $[M+H]^+$ calc'd for $C_{31}H_{33}F_4N_8O_8S^+$ 769.2022, found 769.2030; 1.0 ppm mass defect.

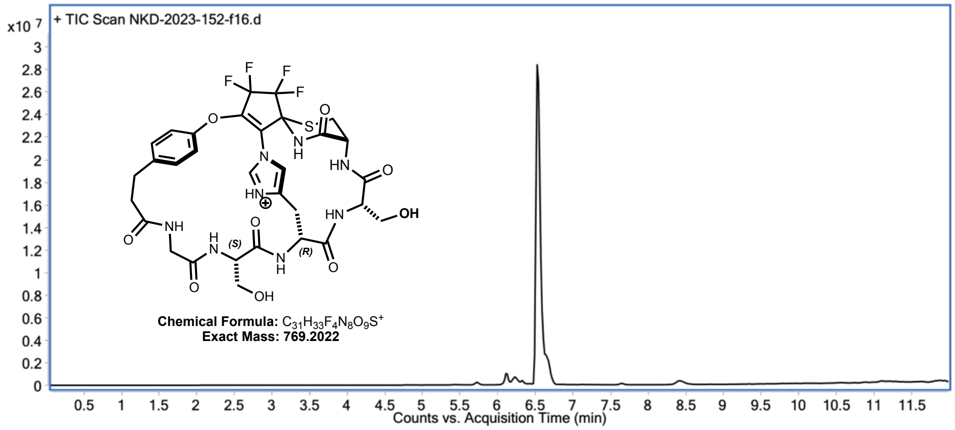

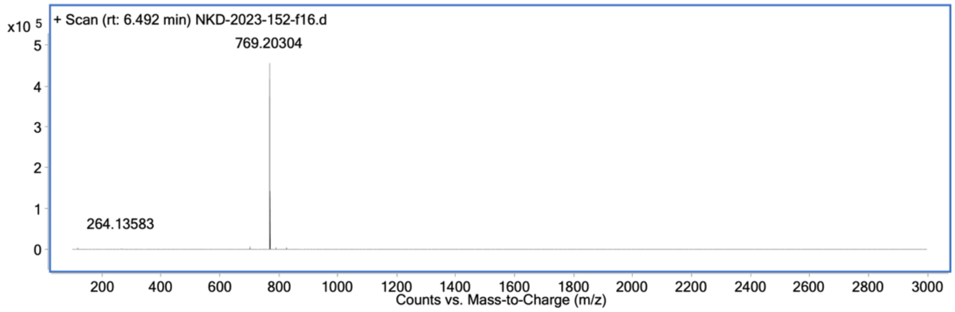

Polycycle 1j. **HRMS** (QTOF) m/z: $[M+H]^+$ calc'd for $C_{32}H_{35}F_4N_8O_{10}S^+$ 799.2127, found 799.2182; 6.9 ppm mass defect.

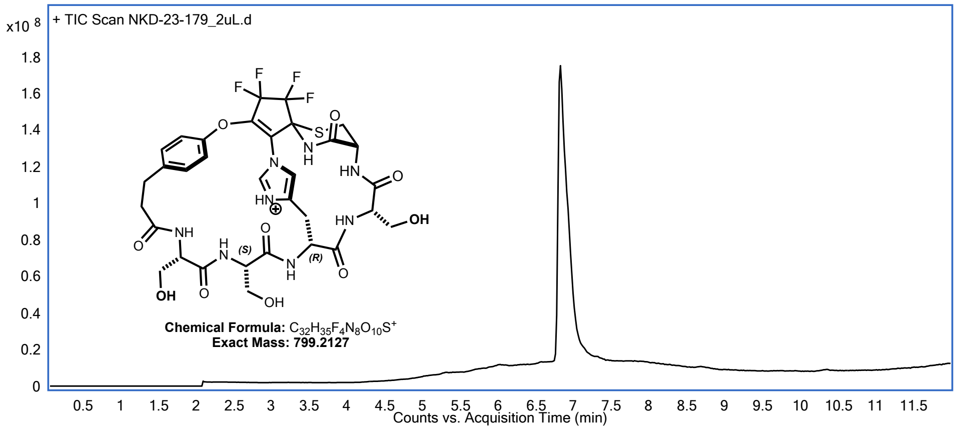

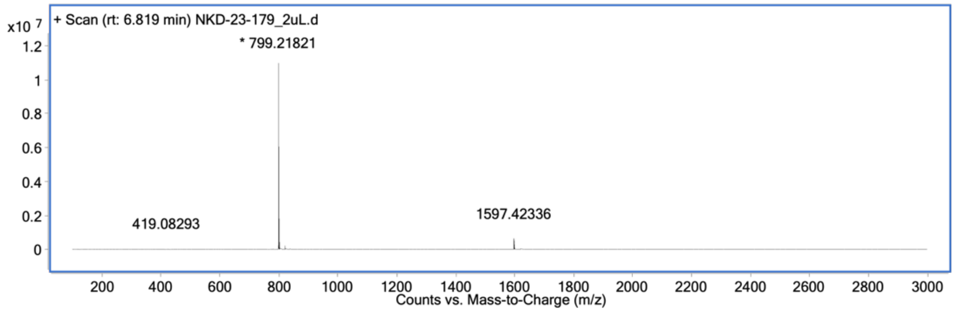

Polycycle 1k. **HRMS** (QTOF) m/z: $[M+H]^+$ calc'd for $C_{32}H_{33}F_4N_8O_9S^+$ 781.2022, found 781.2067; 5.8 ppm mass defect.

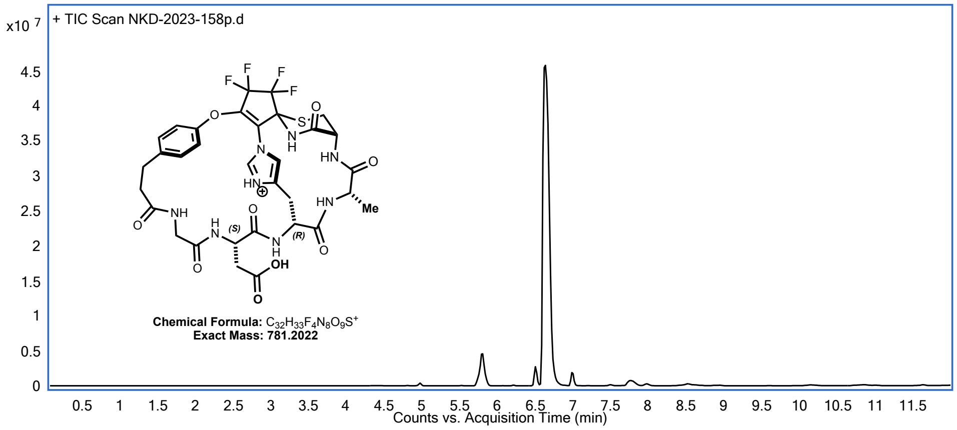

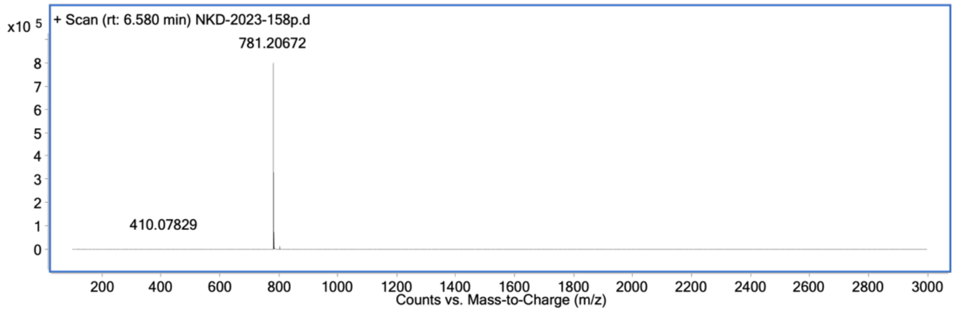

Polycycle 1l. **HRMS** (QTOF) m/z: $[M+H]^+$ calc'd for $C_{39}H_{38}F_4N_9O_7S^+$ 852.2546, found 852.2540; 0.7 ppm mass defect.

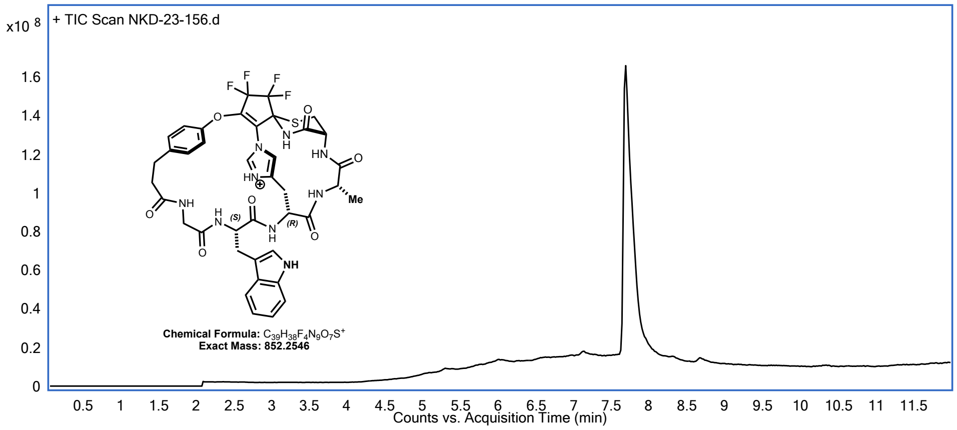

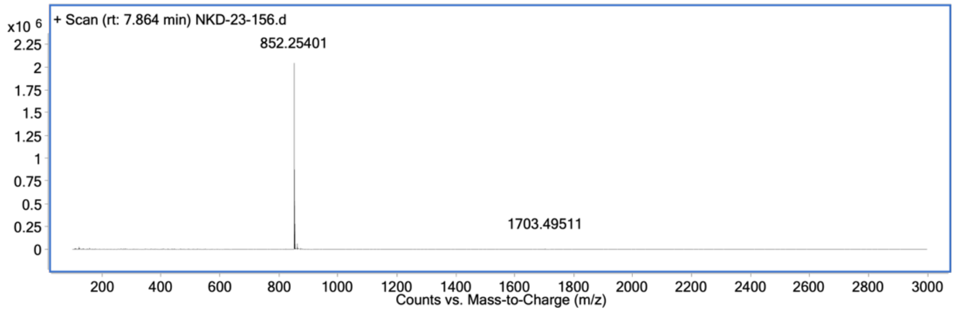

Polycycle 1m. **HRMS** (QTOF) m/z: $[M+H]^+$ calc'd for $C_{32}H_{34}F_4N_9O_9S^+$ 796.2131, found 796.2173; 5.3 ppm mass defect.

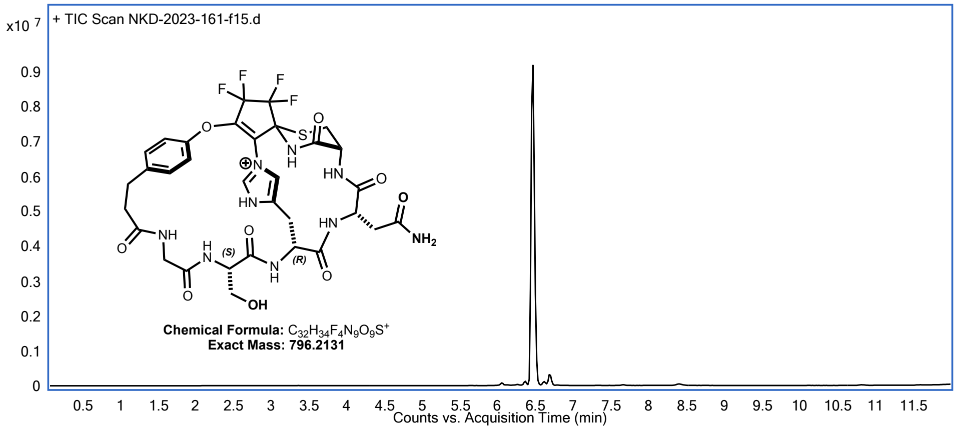

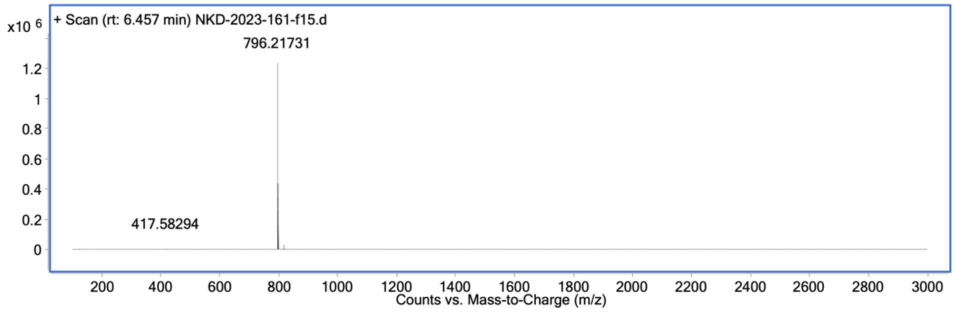

Polycycle 1n. **HRMS** (QTOF) m/z: $[M+H]^+$ calc'd for $C_{34}H_{37}F_4N_{10}O_{10}S^+$ 853.2345, found 853.2385; 4.7 ppm mass defect.

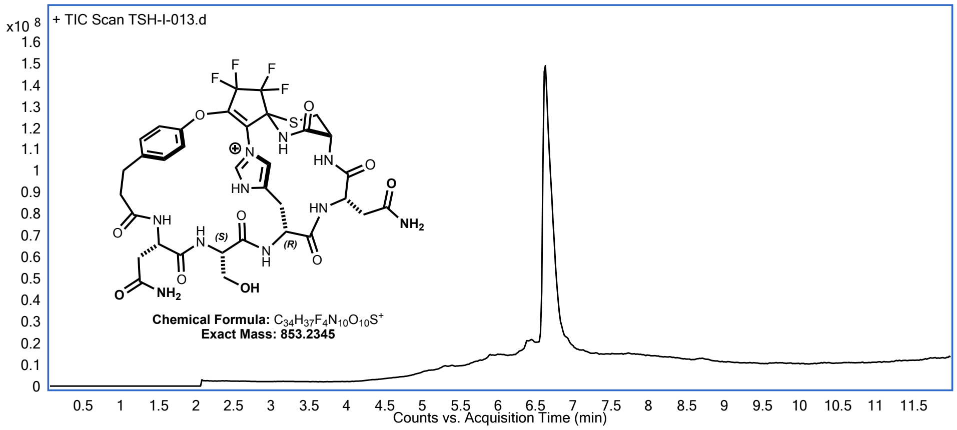

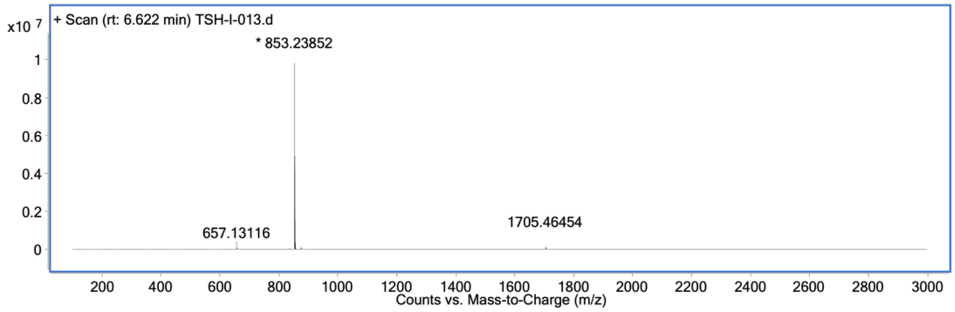

Polycycle 1o. **HRMS** (QTOF) m/z: $[M+H]^+$ calc'd for $C_{34}H_{37}F_4N_8O_8S^+$ 793.2386, found 793.2371; 1.9 ppm mass defect.

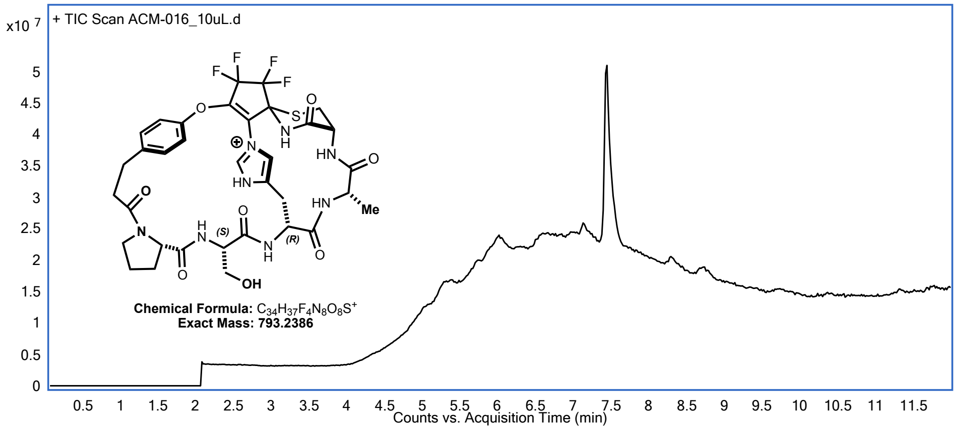

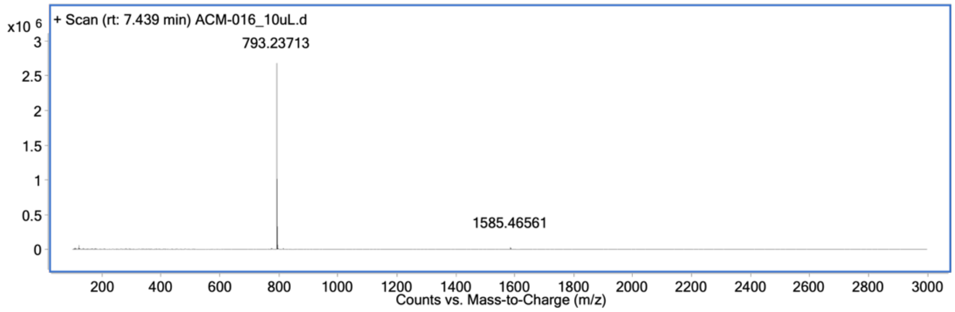

Polycycle 1p. **HRMS** (QTOF) m/z: $[M+H]^+$ calc'd for $C_{31}H_{33}F_4N_8O_8S^+$ 753.2073, found 753.2091; 2.4 ppm mass defect.

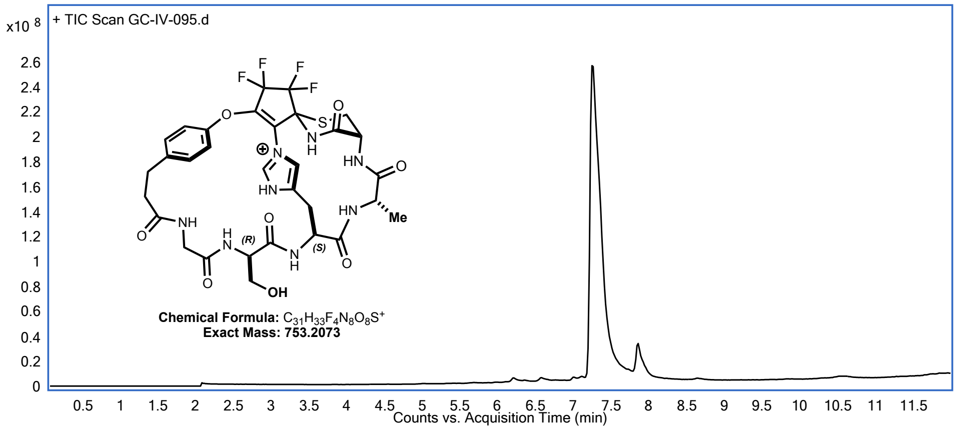

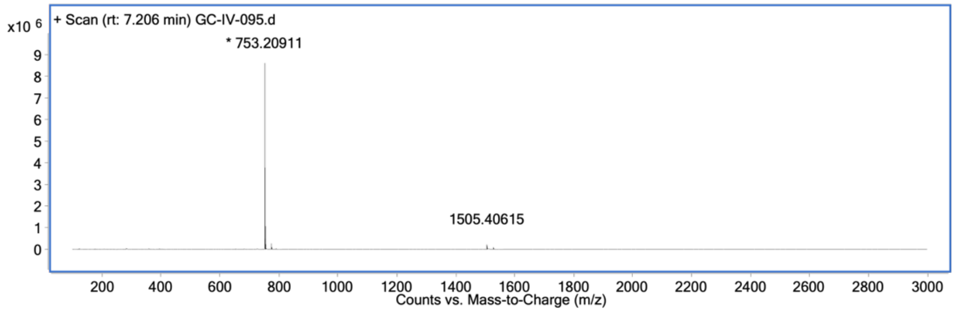

Polycycle 1r. **HRMS** (QTOF) m/z: $[M+H]^+$ calc'd for $C_{25}H_{25}F_4N_6O_5S^+$ 597.1538, found 597.1597; 9.9 ppm mass defect.

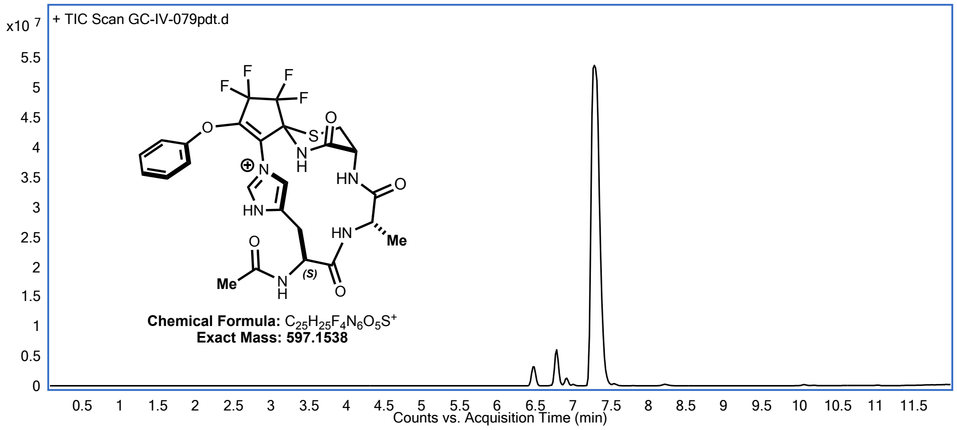

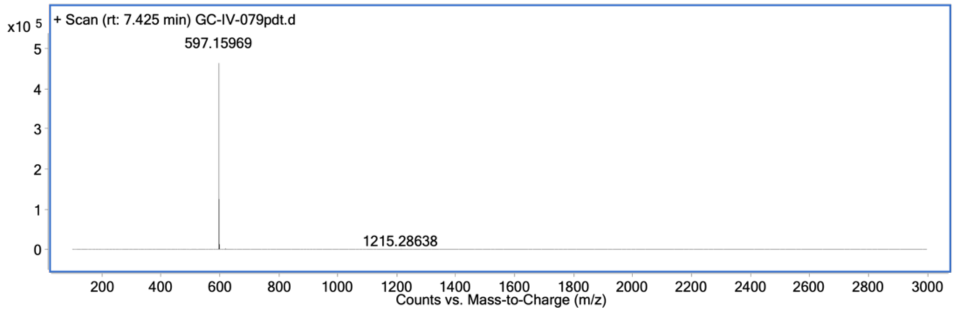

Polycycle 1s. **HRMS** (QTOF) m/z: $[M+H]^+$ calc'd for $C_{30}H_{34}F_4N_7O_9S_2^+$ 776.1790, found 776.1809; 2.4 ppm mass defect.

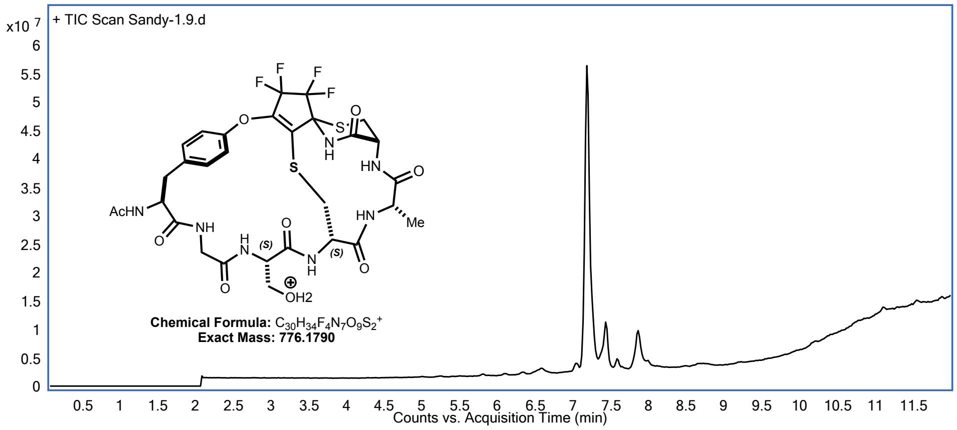

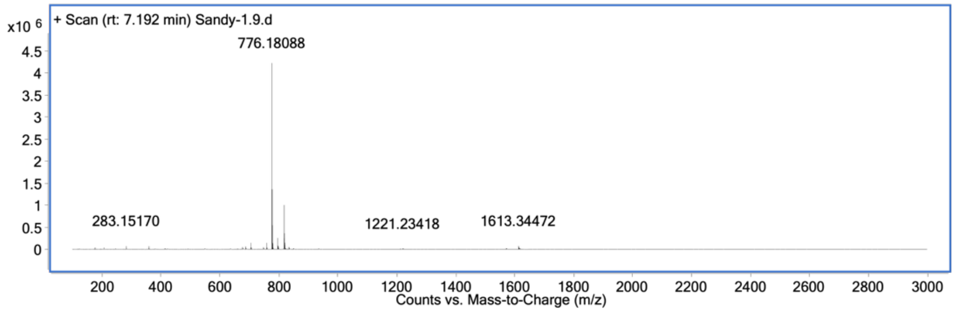

Polycycle 1t. **HRMS** (QTOF) m/z: $[M+H]^+$ calc'd for $C_{32}H_{35}F_4N_{10}O_9S^+$ 811.2240, found 811.2249; 1.1 ppm mass defect.

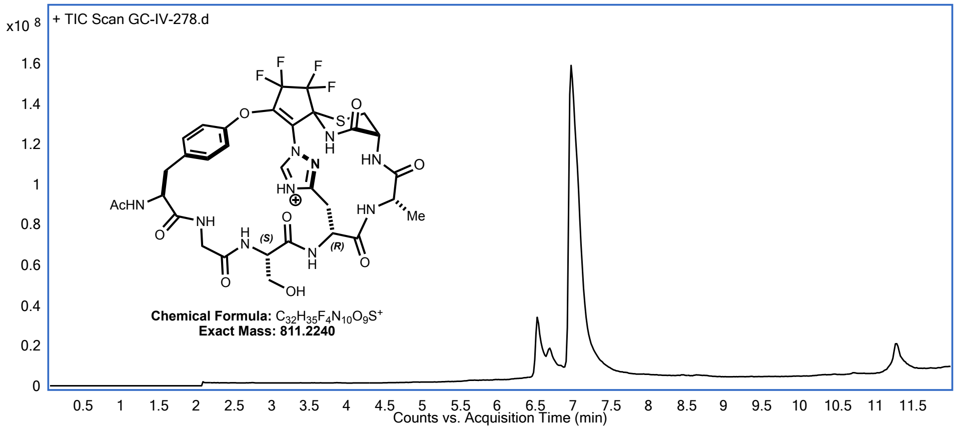

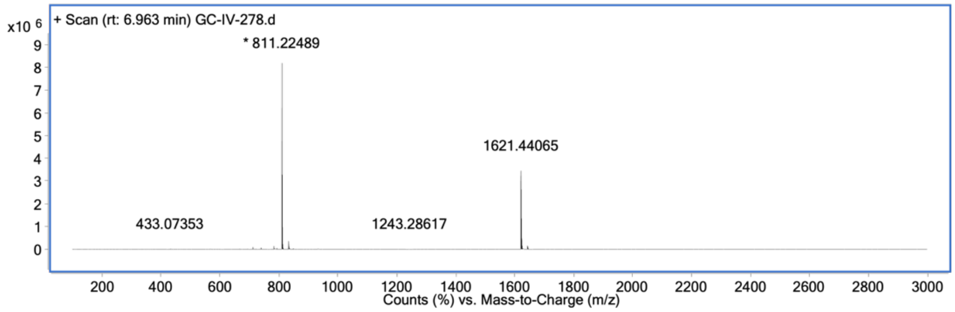

Polycycle 1u. **HRMS** (QTOF) m/z: $[M+H]^+$ calc'd for $C_{32}H_{35}F_4N_{10}O_9S^+$ 811.2240, found 811.2255; 1.9 ppm mass defect.

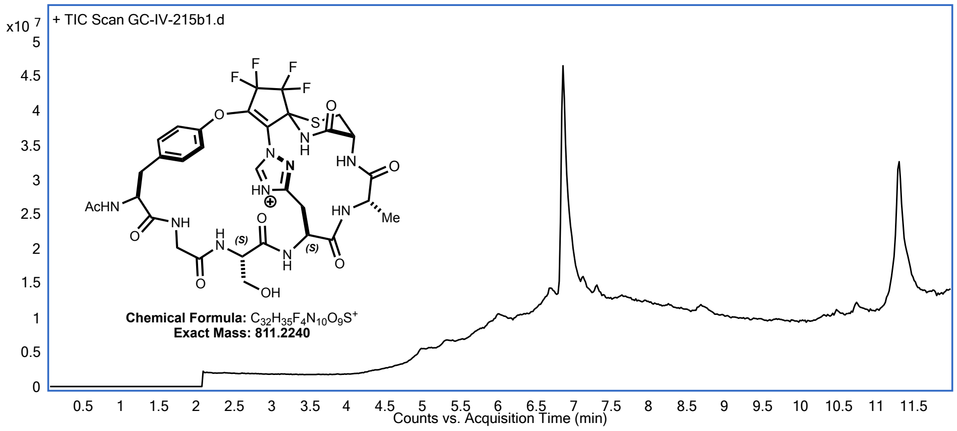

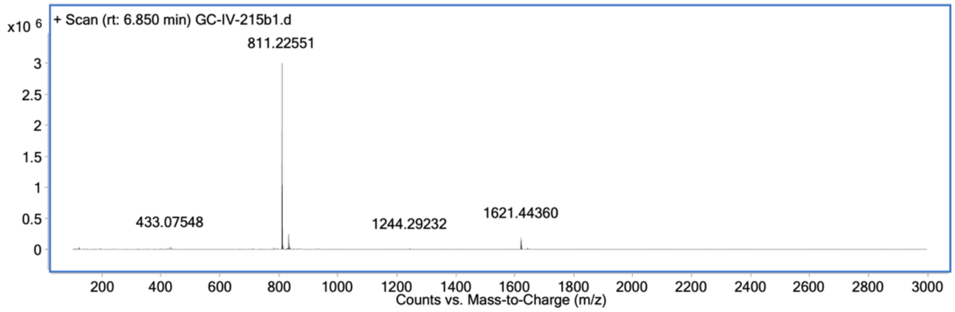

Polycycle 2a. HRMS (QTOF) m/z: $[M+H]^+$ calc'd for $C_{38}H_{45}F_4N_{10}O_{10}S^+$ 909.2971, found 909.2957; 1.5 ppm mass defect.

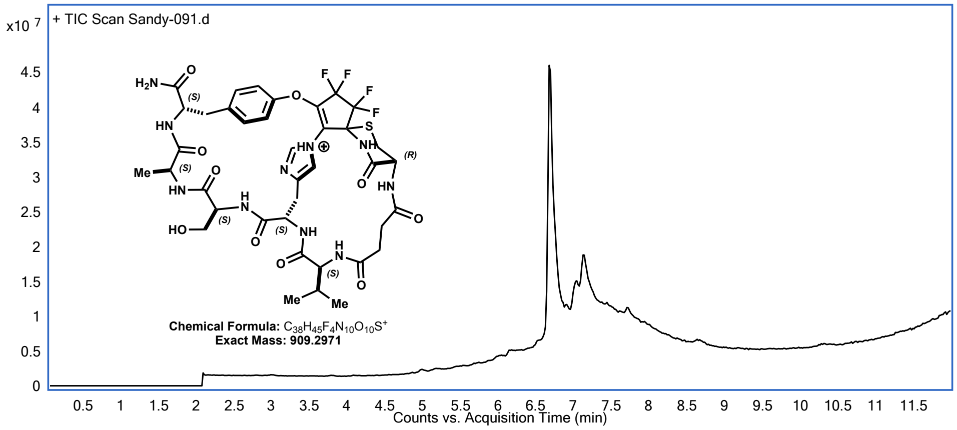

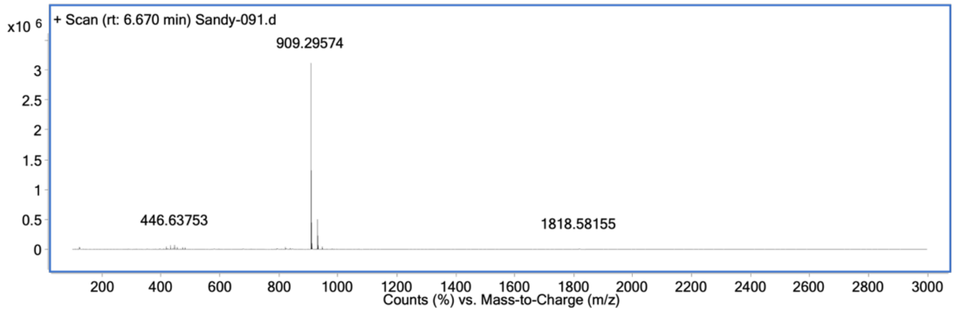

Polycycle 2b. HRMS (QTOF) m/z: $[M+H]^+$ calc'd for $C_{42}H_{50}F_4N_{11}O_{12}S^+$ 1008.3292, found 1008.3323; 3.1 ppm mass defect.

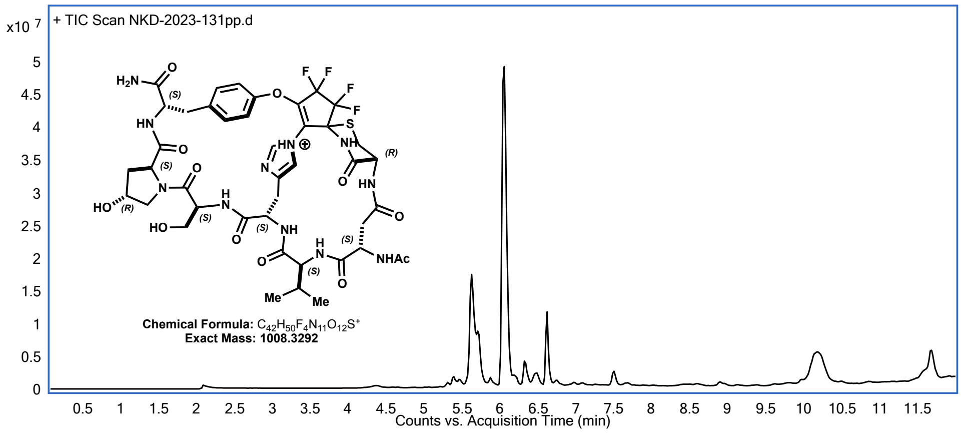

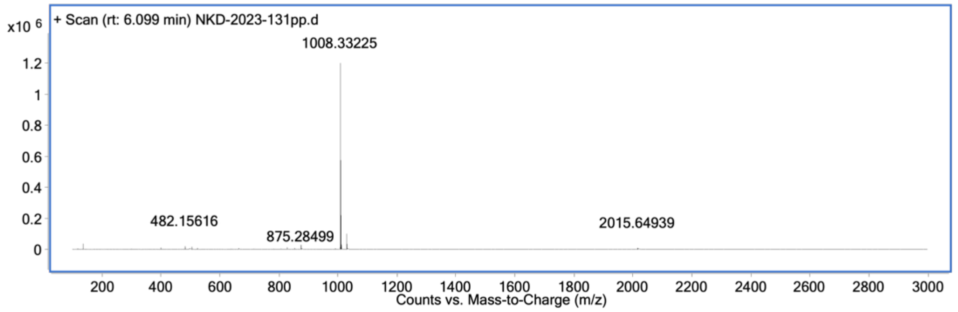

Polycycle 3a. HRMS (QTOF) m/z: $[M+H]^+$ calc'd for $C_{37}H_{44}F_4N_7O_{11}S_2^+$ 902.2471, found 902.2488; 1.9 ppm mass defect.

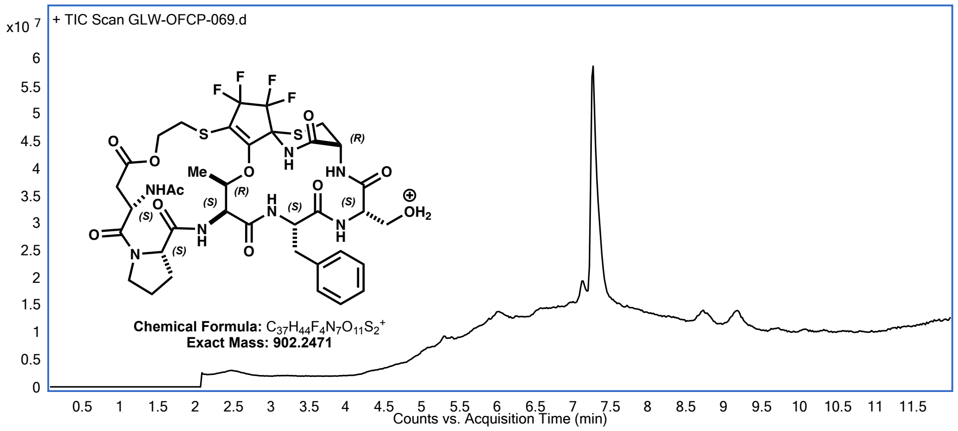

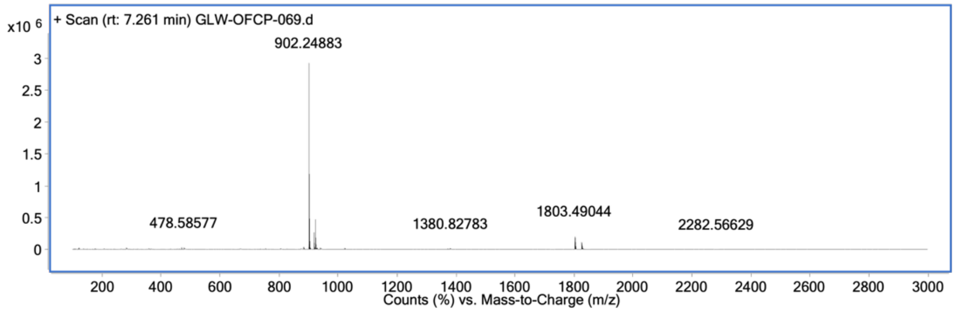

Polycycle 3c. HRMS (QTOF) m/z: [M+H]+ calc'd for $C_{36}H_{45}F_4N_8O_{10}S_2^+$ 889.2631, found 889.2630; 0.1 ppm mass defect.

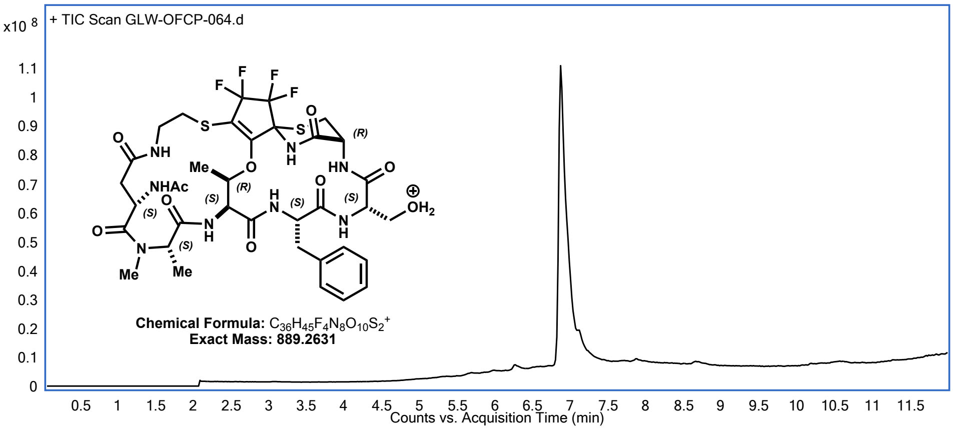

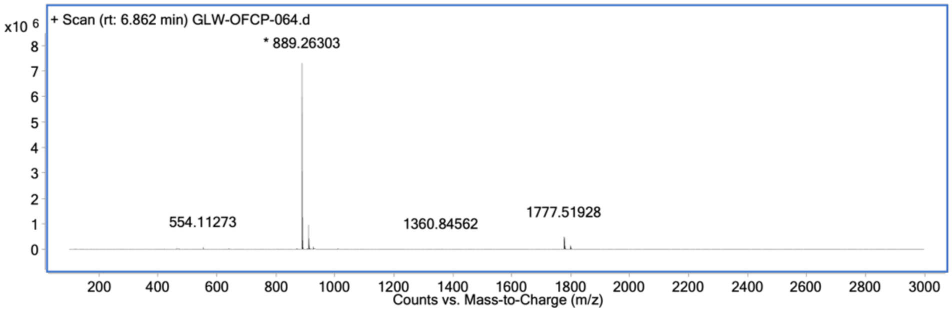

Polycycle 3d. HRMS (QTOF) m/z: [M+H]+ calc'd for C37H45F4N8O10S2+ 901.2631, found 901.2656; 2.8 ppm mass defect.

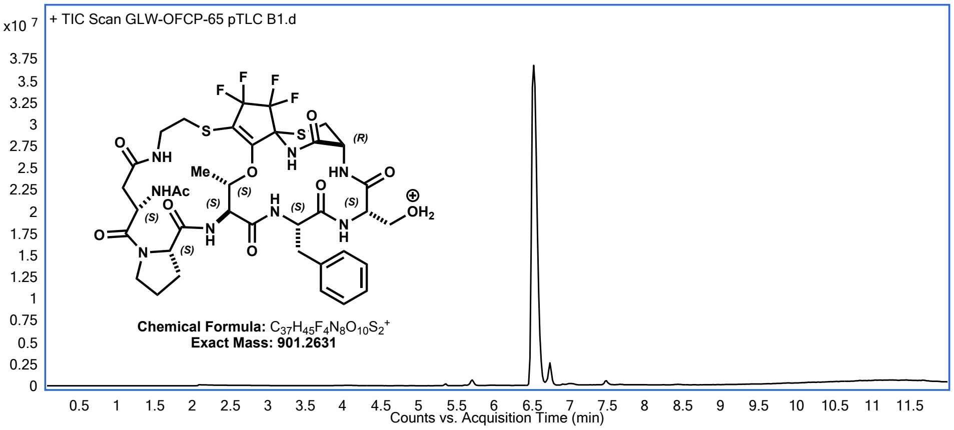

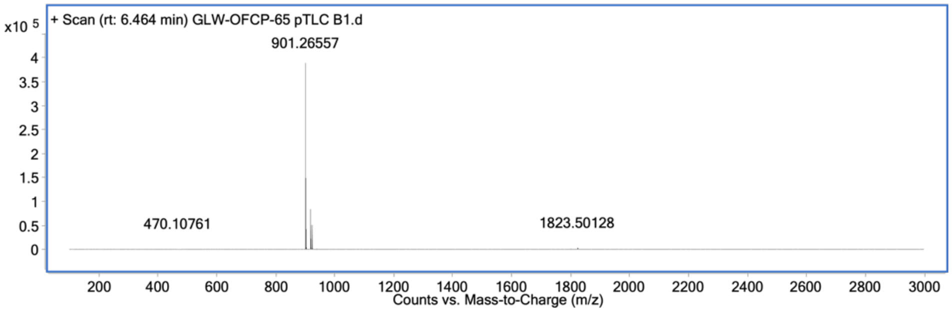

Polycycle 3e. HRMS (QTOF) m/z: $[M+H]^+$ calc'd for $C_{38}H_{47}F_4N_8O_{10}S_2^+$ 915.2787, found 915.2776; 1.2 ppm mass defect.

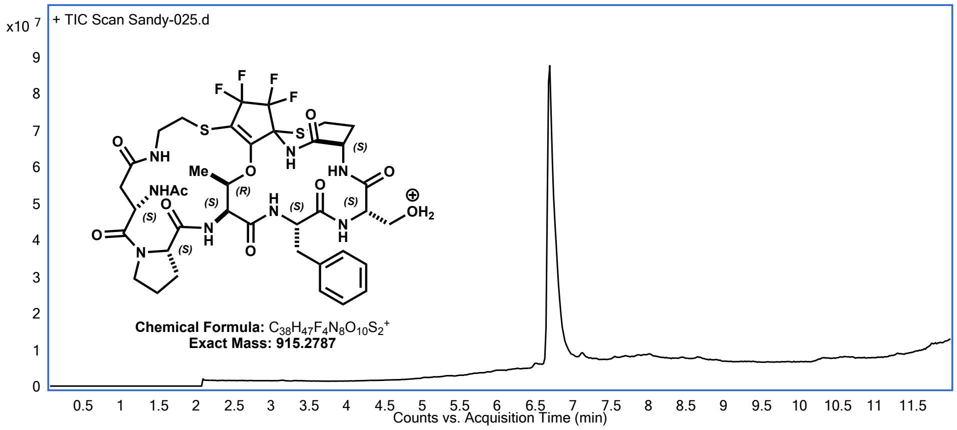

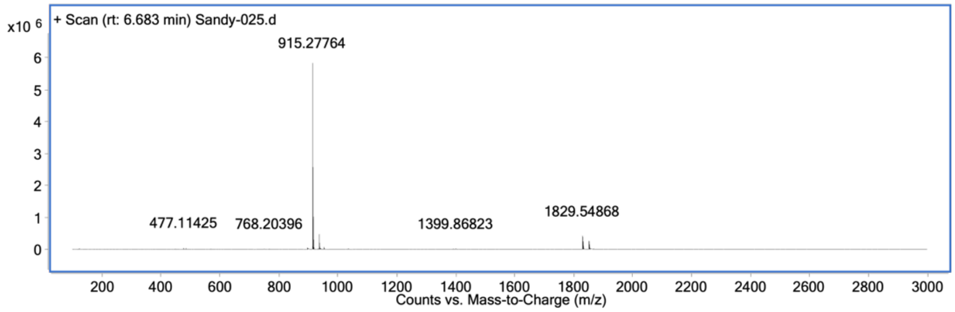

Polycycle 3j. HRMS (QTOF) m/z: $[M+H]^+$ calc'd for $C_{40}H_{45}F_4N_8O_{11}S_3^+$ 985.2301, found 985.2283; 1.8 ppm mass defect.

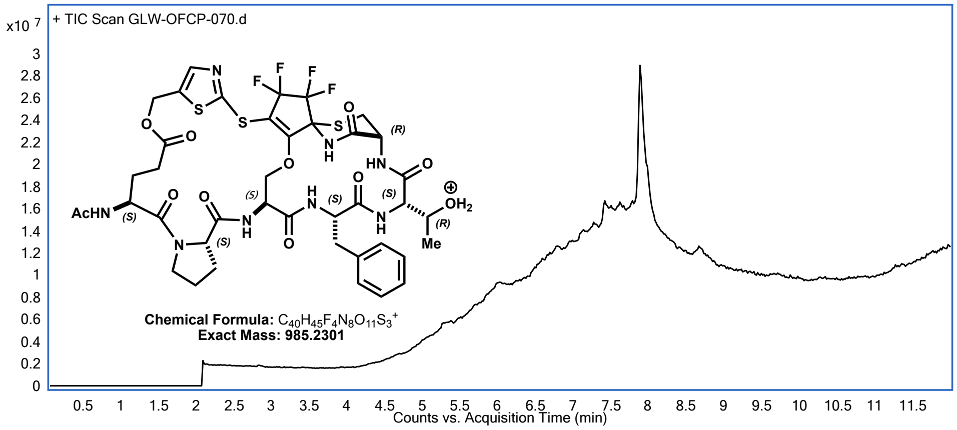

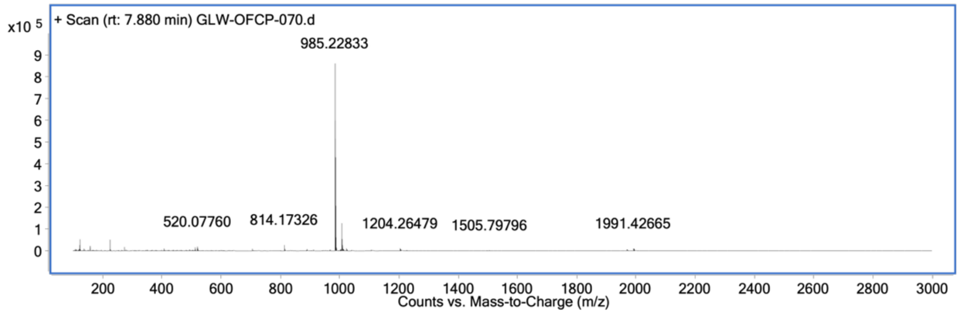

Polycycle 3k. HRMS (QTOF) m/z: $[M+H]^+$ calc'd for $C_{37}H_{47}F_4N_8O_{10}S_2^+$ 903.2787, found 903.2805; 2.0 ppm mass defect.

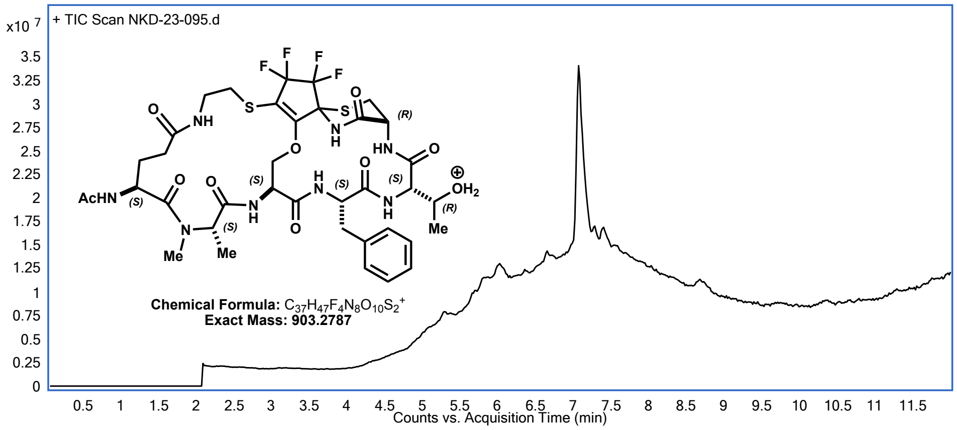

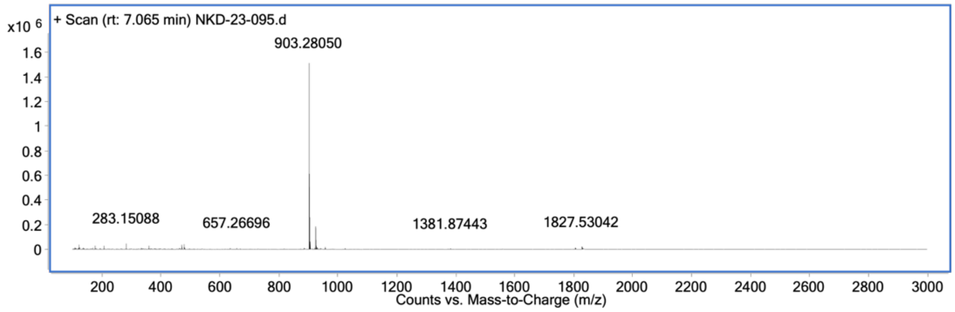

Polycycle 3l. HRMS (QTOF) m/z: $[M+H]^+$ calc'd for $C_{39}H_{49}F_4N_8O_{10}S_2^+$ 929.2944, found 929.2901; 4.6 ppm mass defect.

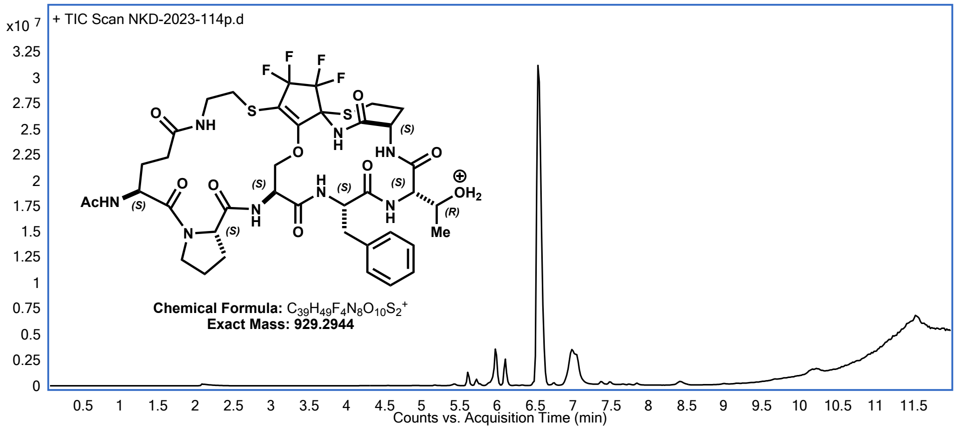

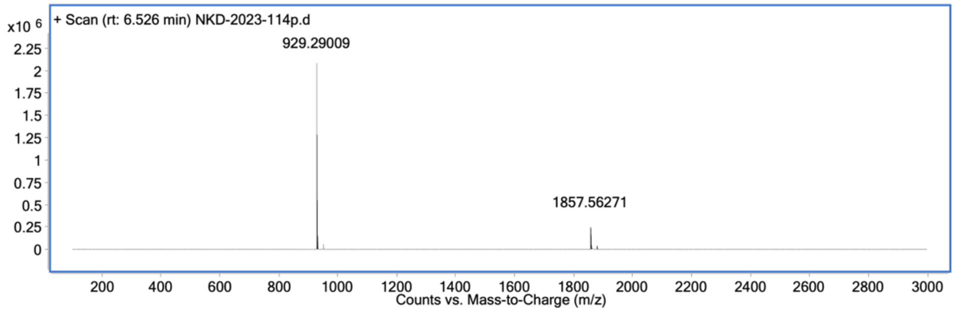

Polycycle 3q. HRMS (QTOF) m/z: $[M+H]^+$ calc'd for $C_{33}H_{40}F_4N_9O_9S^+$ 814.2600, found 814.2593; 0.9 ppm mass defect.

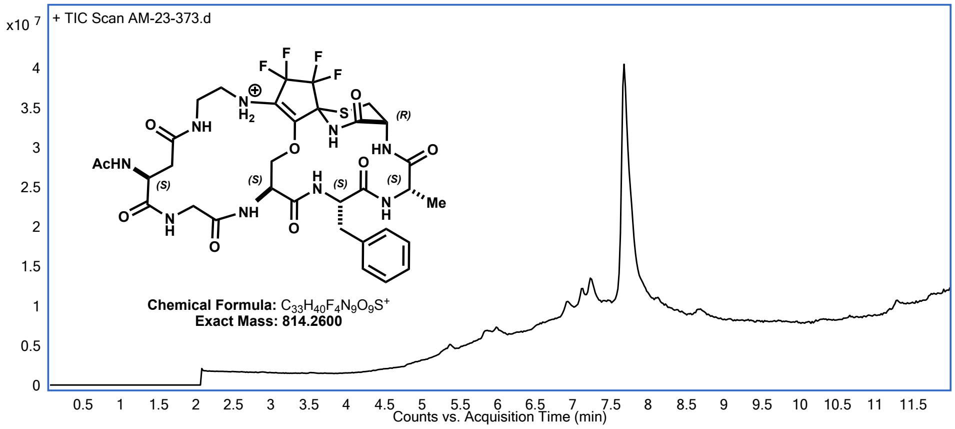

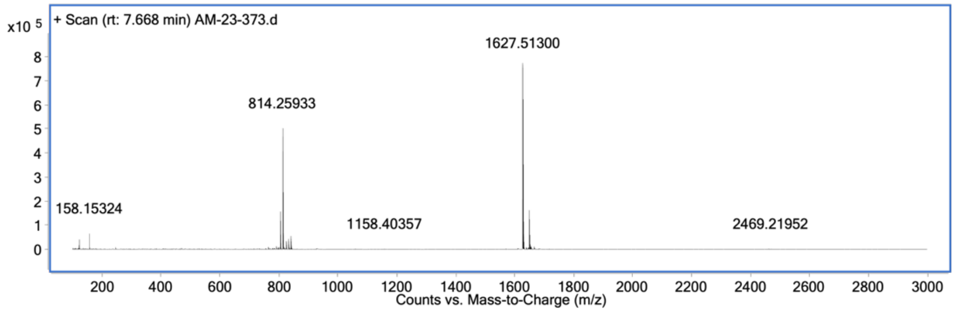

Polycycle S2b. HRMS (QTOF) m/z: $[M+H]^+$ calc'd for $C_{45}H_{64}F_4N_7O_6S_2^+$ 938.4290, found 938.4298; 0.9 ppm mass defect.

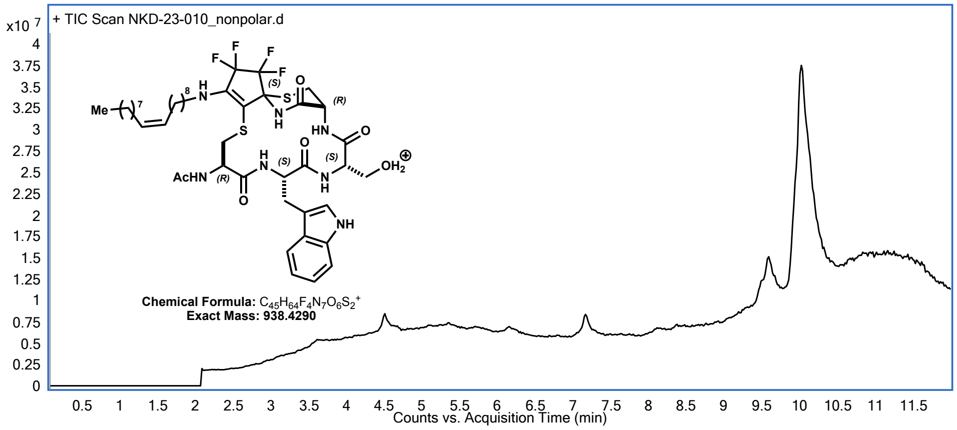

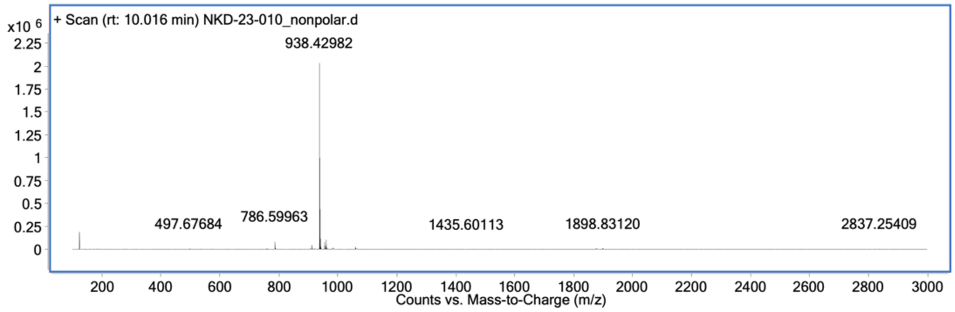

Polycycle S2c. HRMS (QTOF) m/z: [M+H]$^+$ calc'd for $C_{33}H_{42}F_4N_7O_6S_2^+$ 772.2569, found 772.2565; 0.5 ppm mass defect.

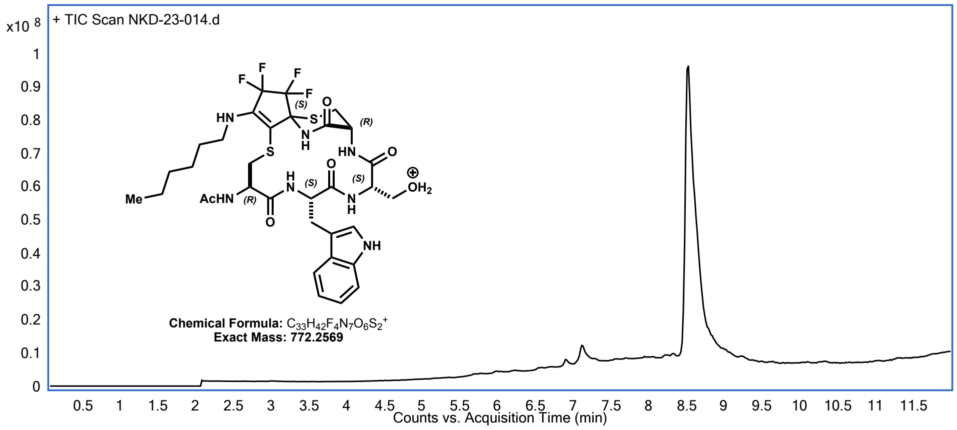

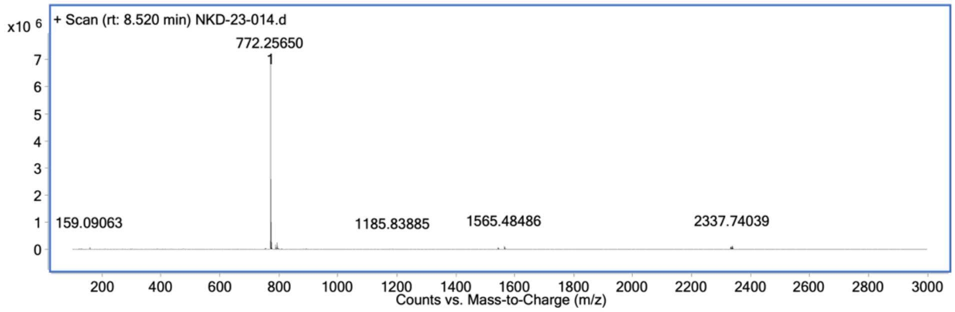

Polycycle S2d. HRMS (QTOF) m/z: [M+H]$^+$ calc'd for $C_{30}H_{36}F_4N_7O_6S_2^+$ 730.2099, found 730.2124; 3.4 ppm mass defect.

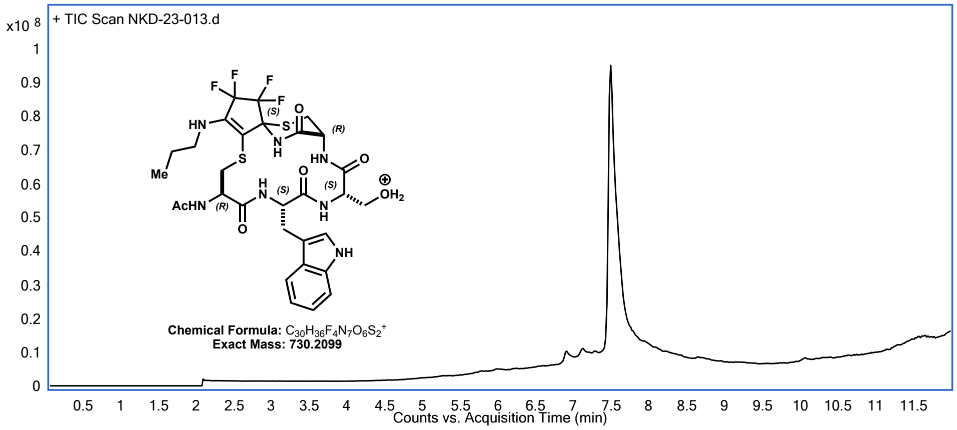

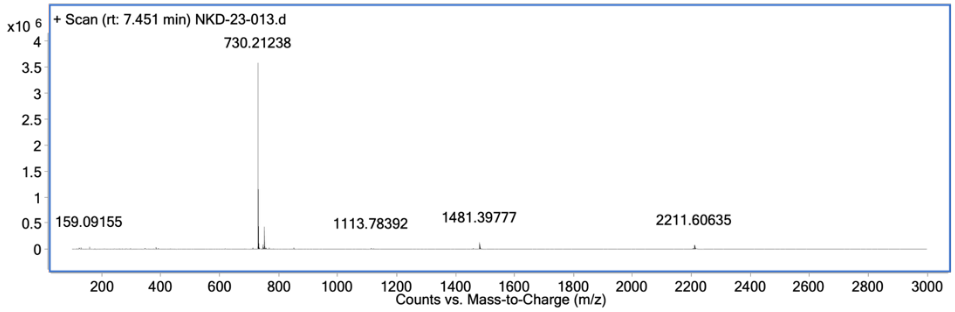

Polycycle S2e. HRMS (QTOF) m/z: $[M+H]^+$ calc'd for $C_{31}H_{32}F_4N_9O_6S_2^+$ 783.1459, found 783.1477; 2.3 ppm mass defect.

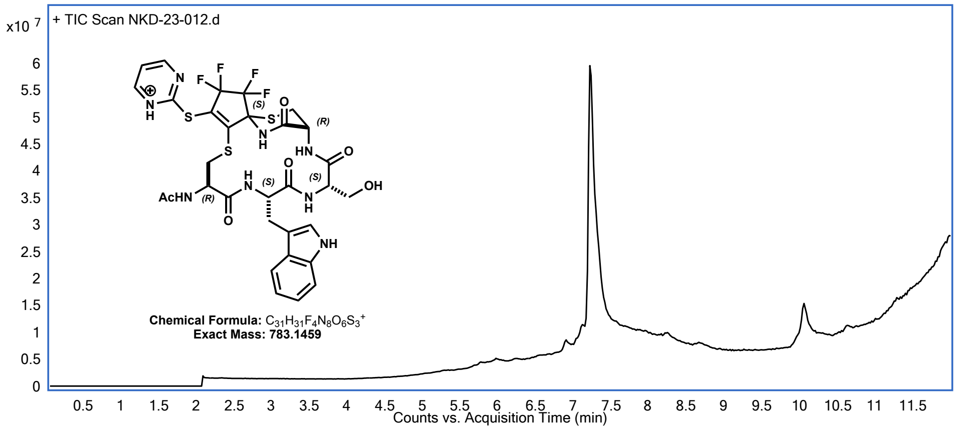

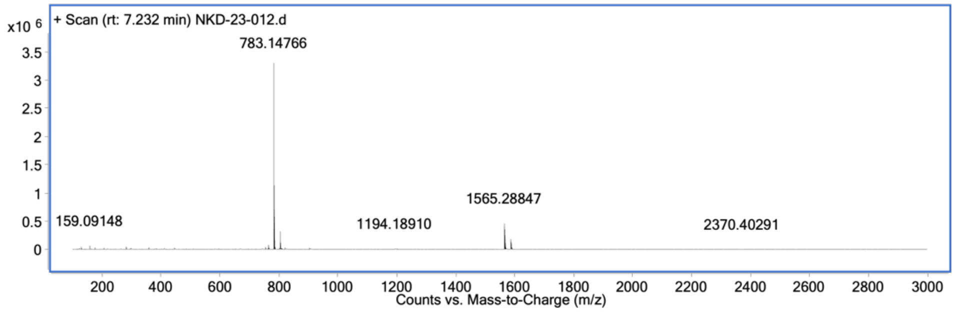

Polycycle S3a. HRMS (QTOF) m/z: $[M+H]^+$ calc'd for $C_{34}H_{54}F_4N_5O_5S_2^+$ 752.3497, found 752.3502; 0.7 ppm mass defect.

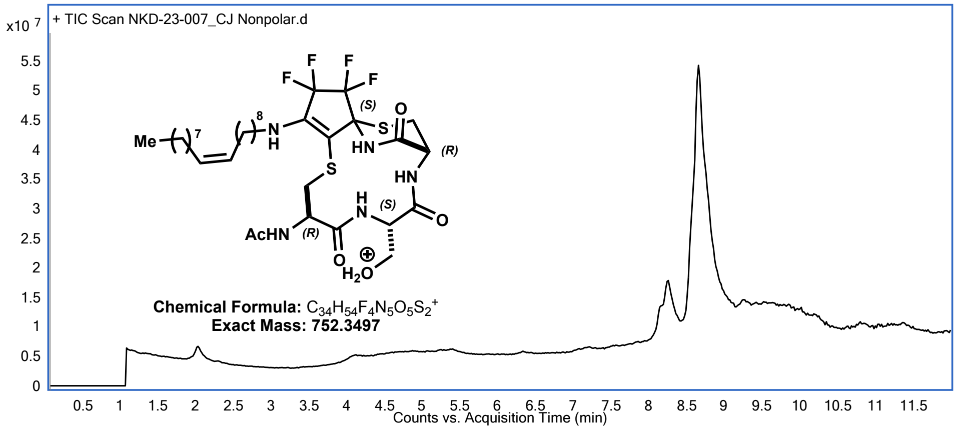

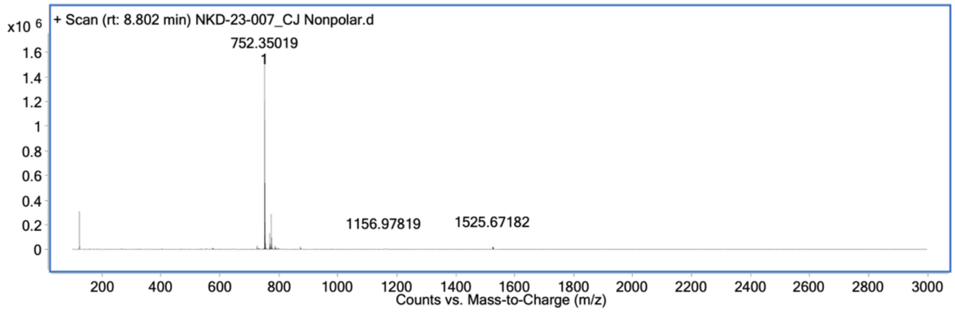

Polycycle S3b. HRMS (QTOF) m/z: $[M+H]^+$ calc'd for $C_{24}H_{28}F_4N_5O_5S_2^+$ 606.1463, found 606.1490; 4.5 ppm mass defect.

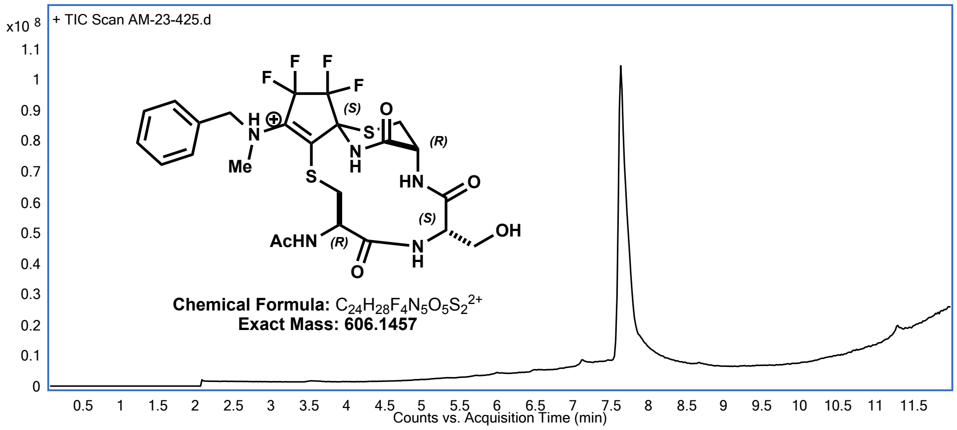

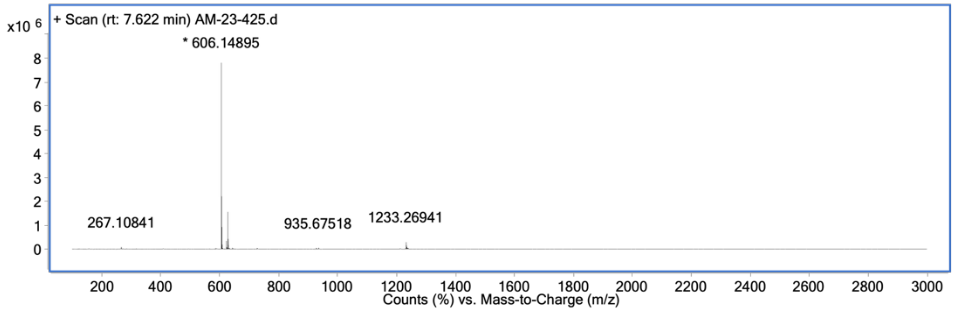

Polycycle S3c. HRMS (QTOF) m/z: $[M+H]^+$ calc'd for $C_{21}H_{22}F_4N_5O_5S_3^+$ 596.0714, found 596.0718; 0.7ppm mass defect.

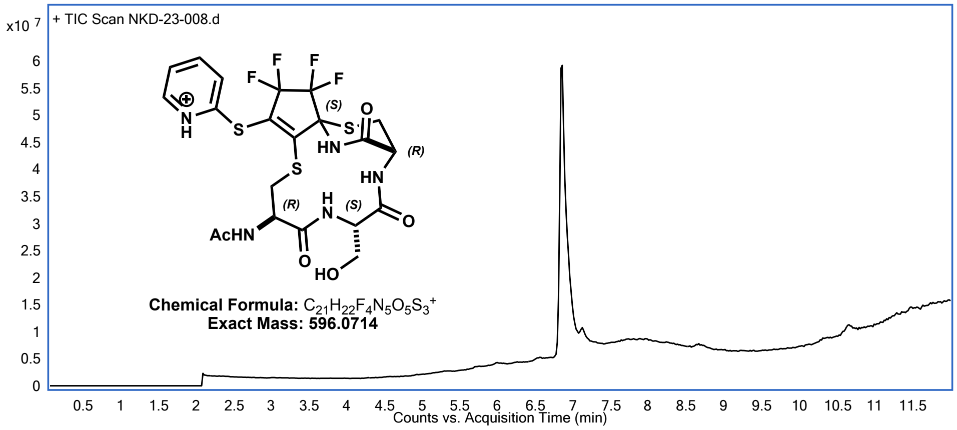

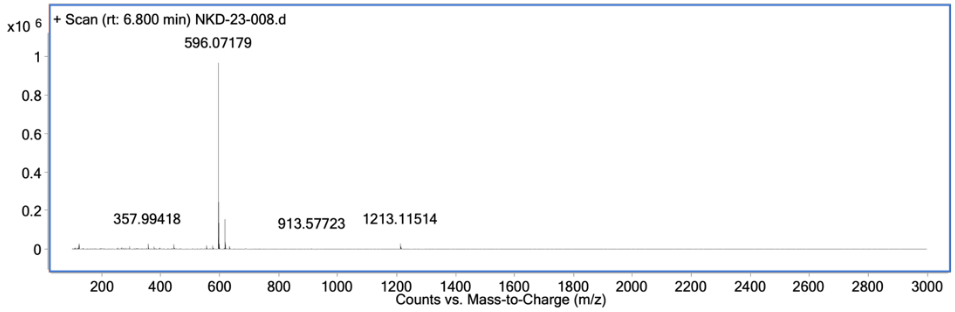

Polycycle S5a. HRMS (QTOF) m/z: $[M+H]^+$ calc'd for $C_{33}H_{39}F_4N_6O_7S_2^+$ 771.2252, found 771.2264; 1.6 ppm mass defect.

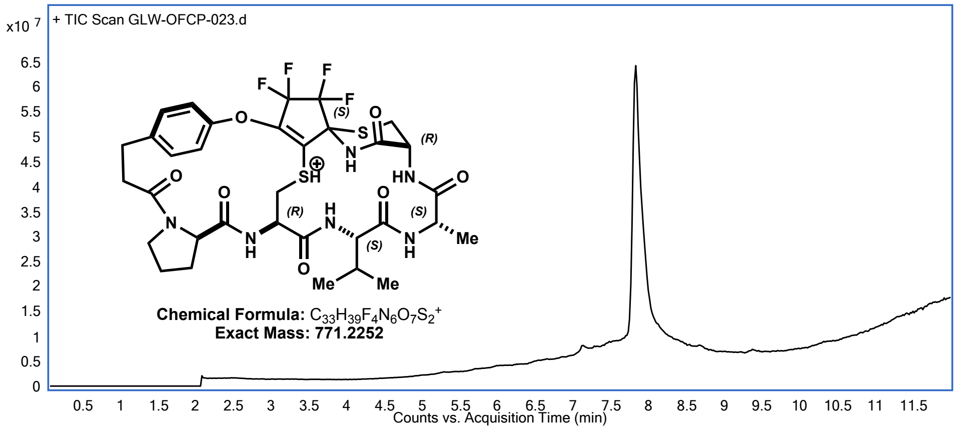

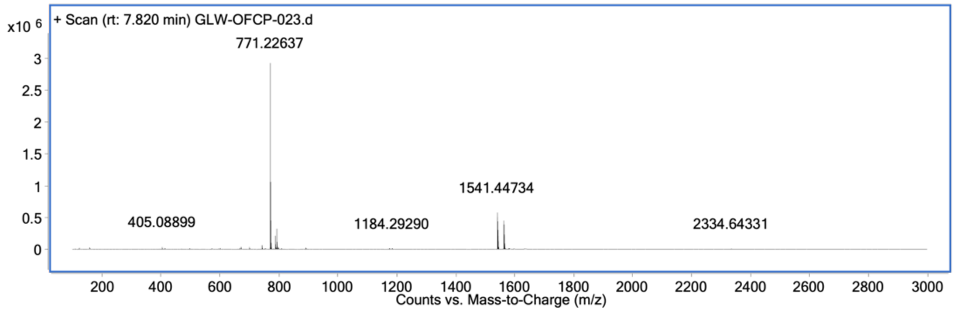

NMR Spectra

Polycycle 1b.

^{1}H NMR (500 MHz, DMSO)

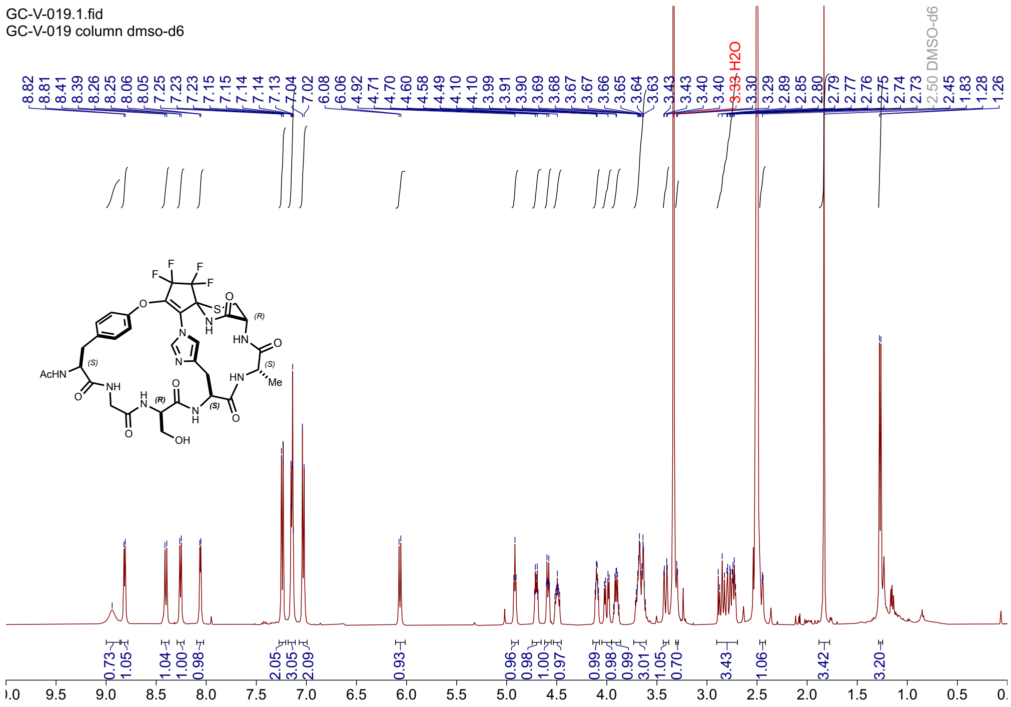

^{13}C NMR (126 MHz, DMSO)

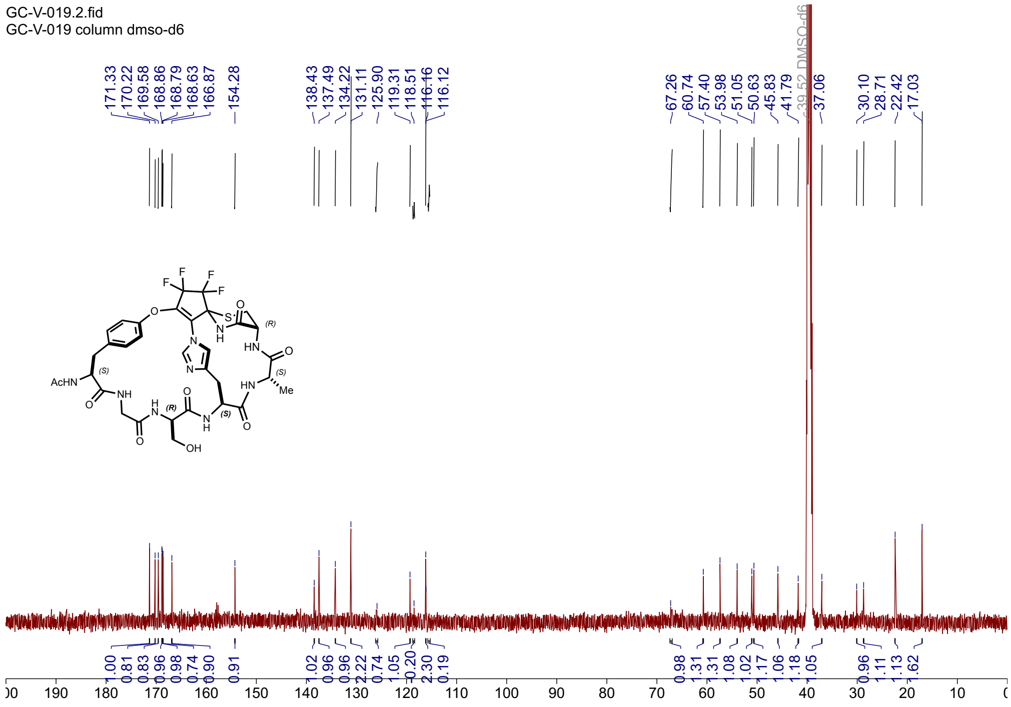

^{19}F NMR (376 MHz, DMSO)

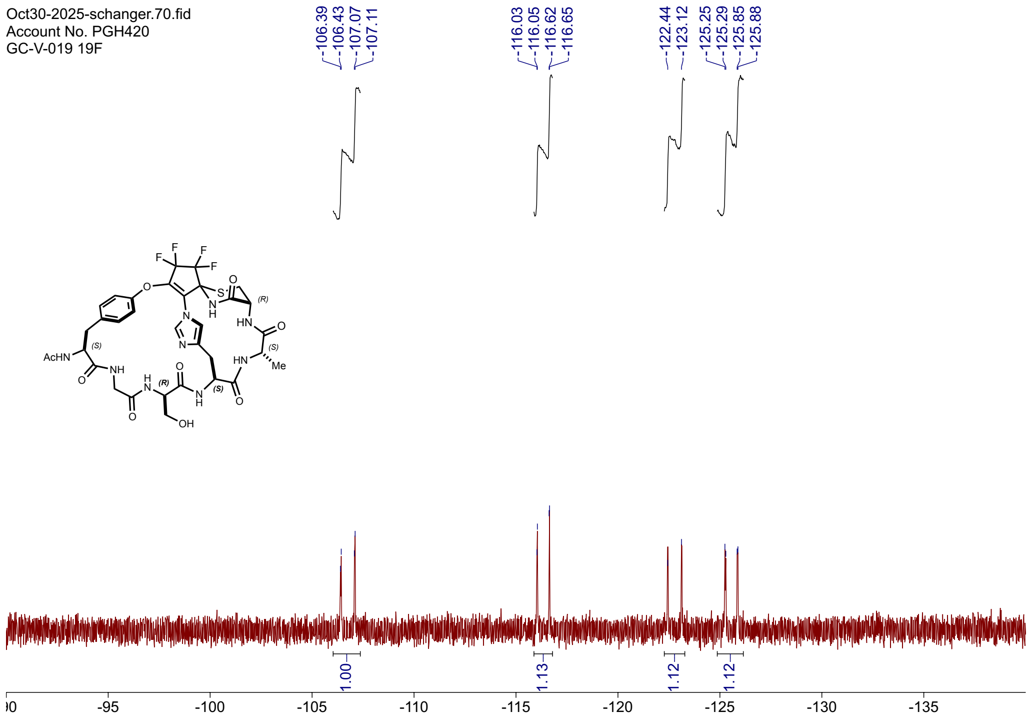

Polycycle 1c.

^{1}H NMR (500 MHz, MeOD)

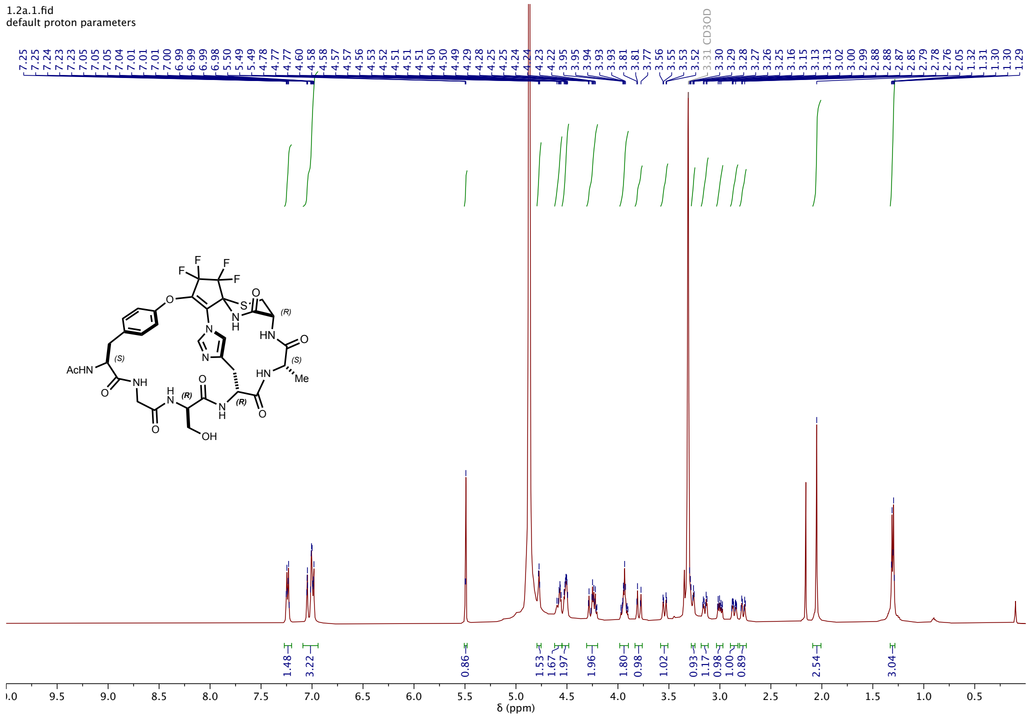

^{13}C NMR (126 MHz, MeOD)

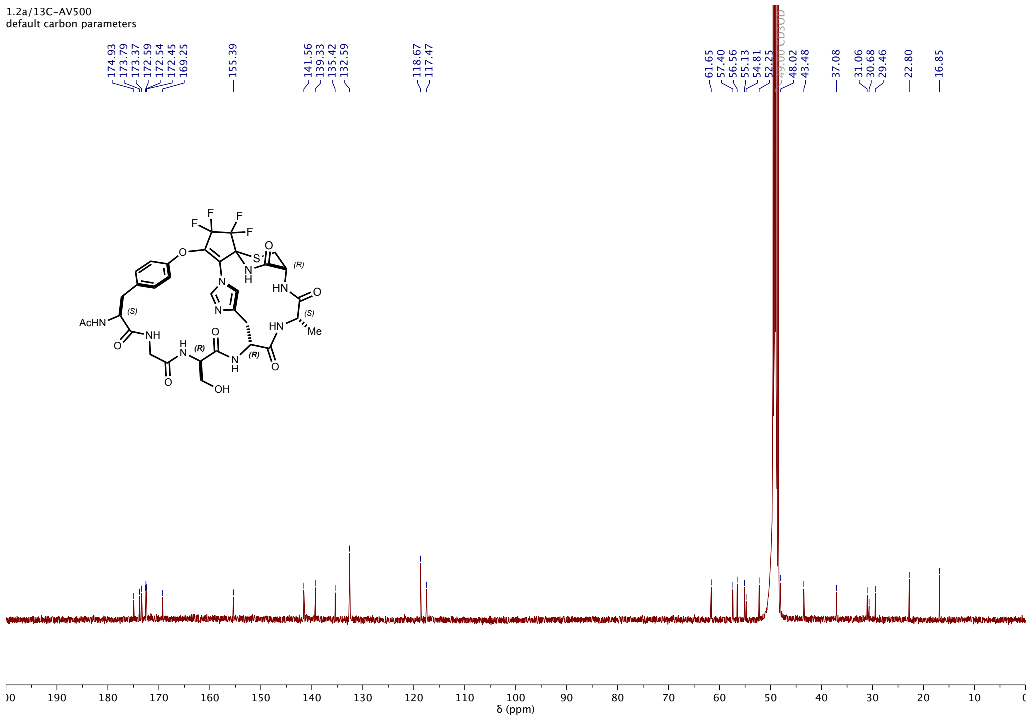

^{19}F NMR (376 MHz, MeOD)

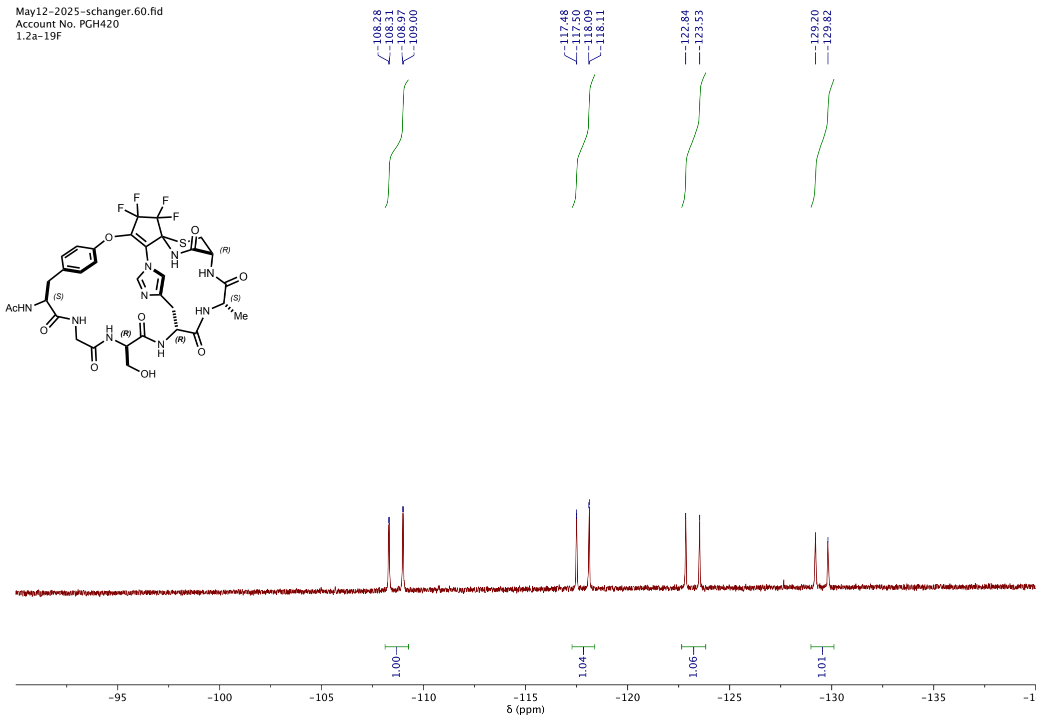

Polycycle 1e.

^{1}H NMR (500 MHz, MeOD)

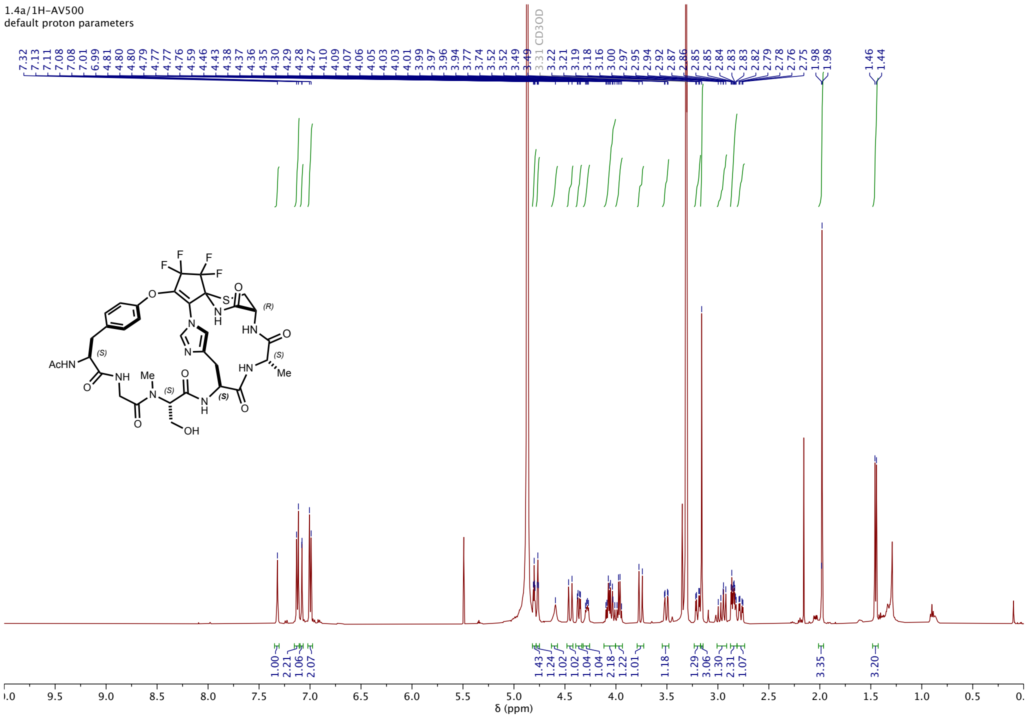

^{13}C NMR (126 MHz, MeOD)

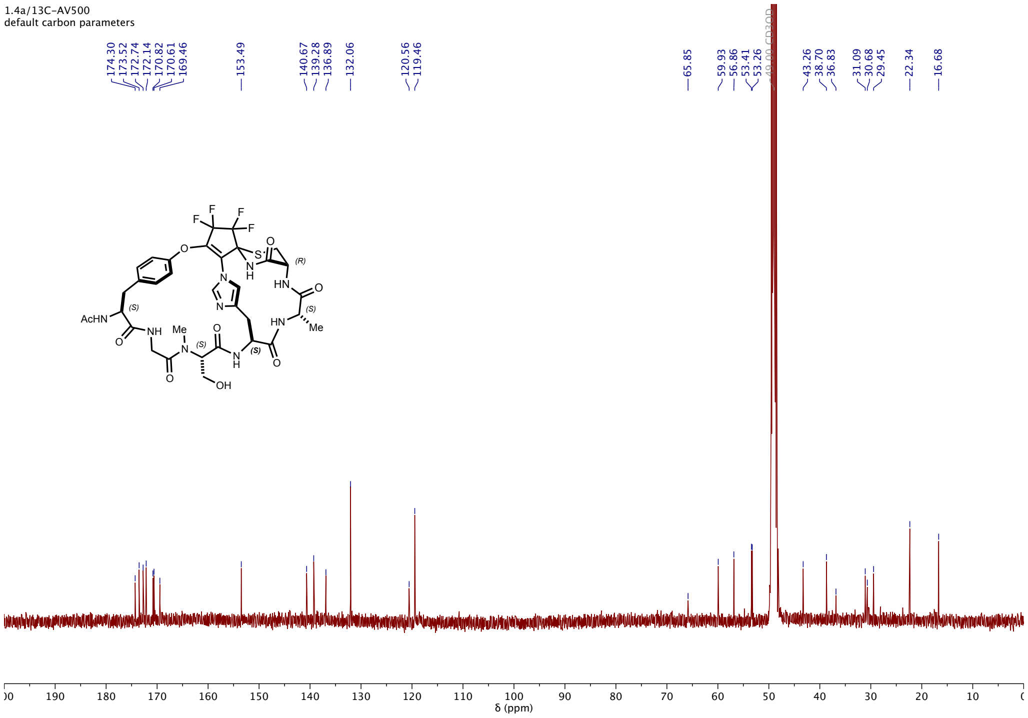

^{19}F NMR (376 MHz, MeOD)

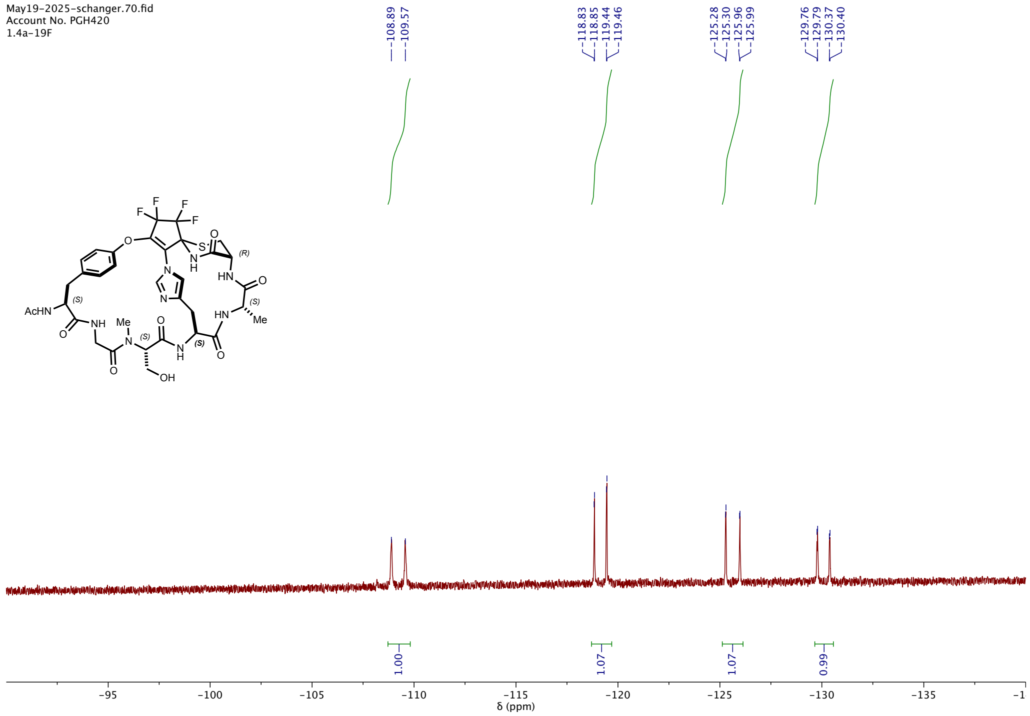

Polycycle 1f.

^{1}H NMR (500 MHz, MeOD)

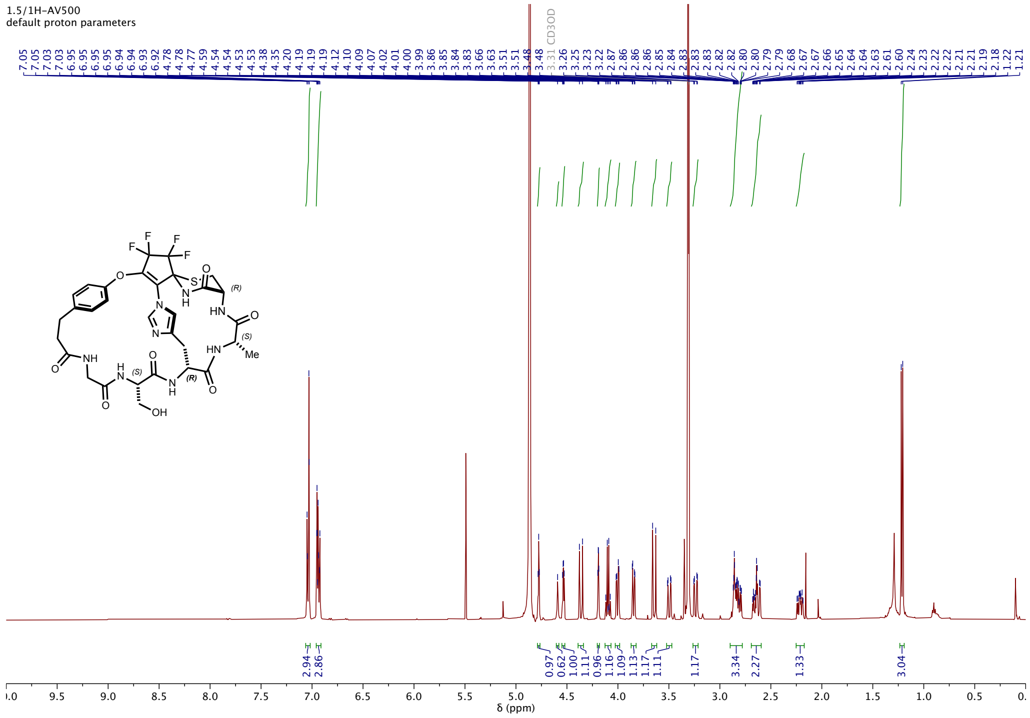

^{13}C NMR (126 MHz, MeOD)

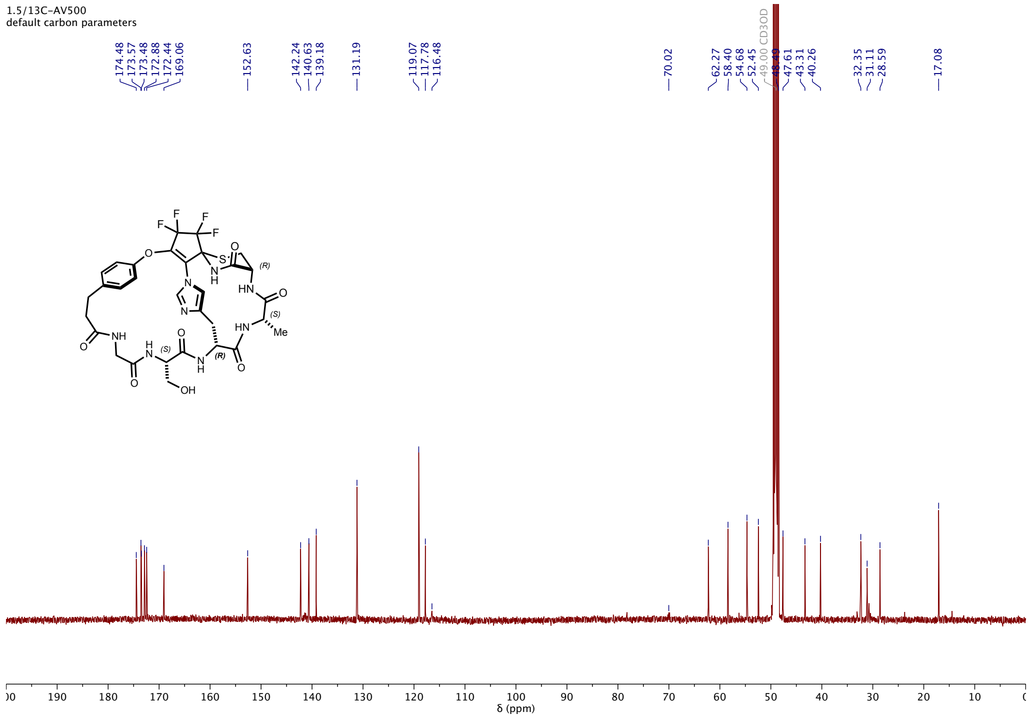

^{19}F NMR (376 MHz, MeOD)

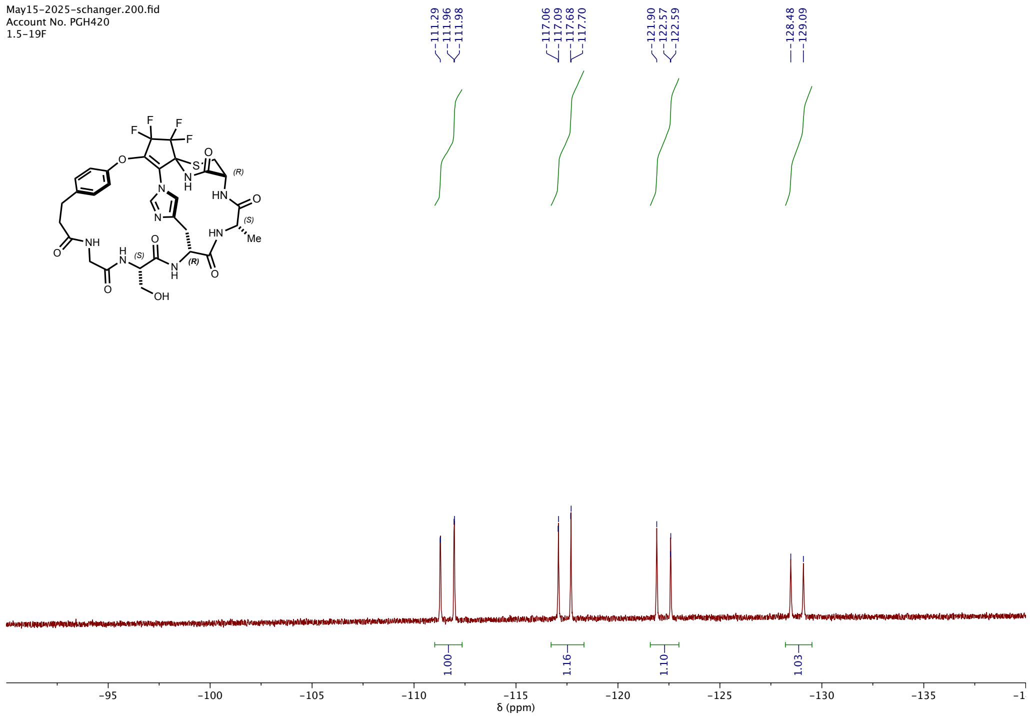

Polycycle 1g.

¹H NMR (500 MHz, MeOD)

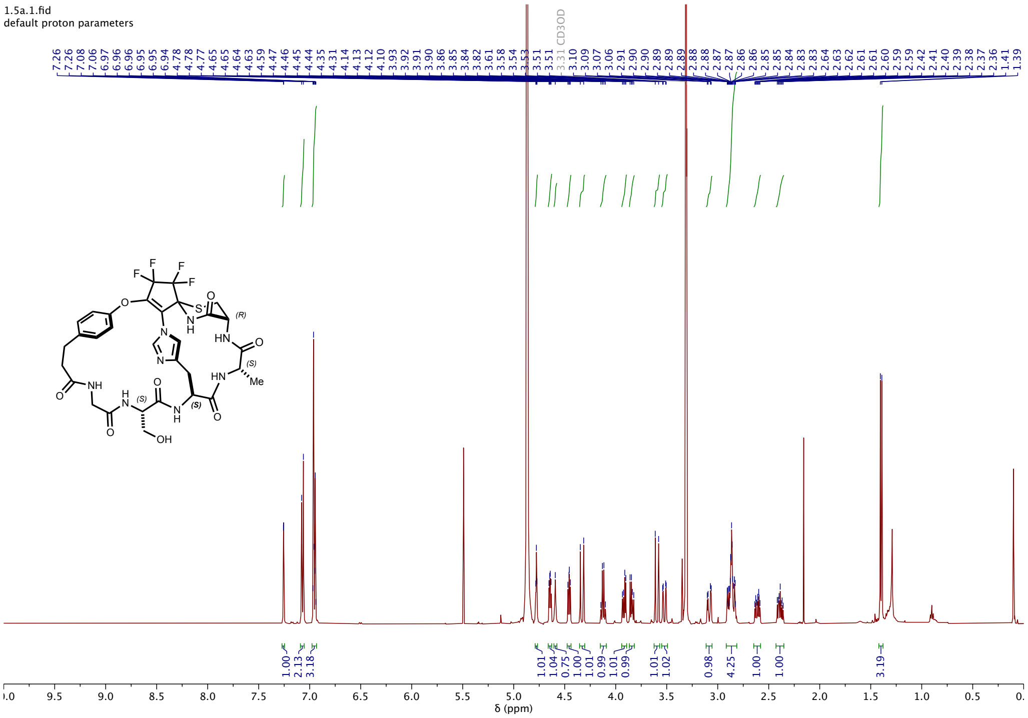

¹³C NMR (126 MHz, MeOD)

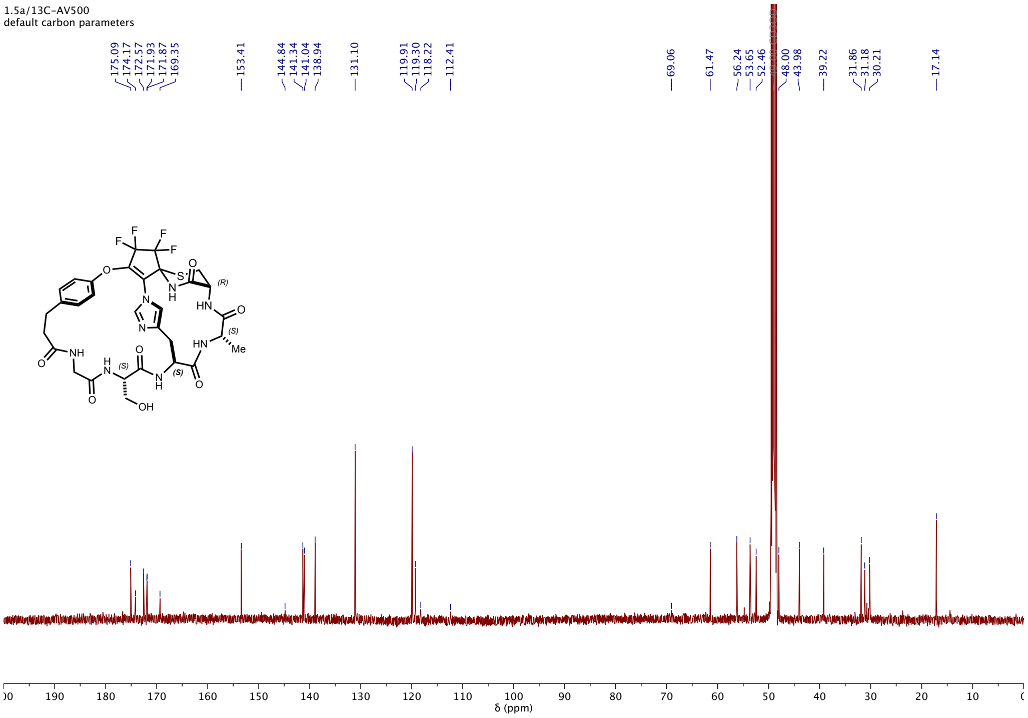

^{19}F NMR (376 MHz, MeOD)

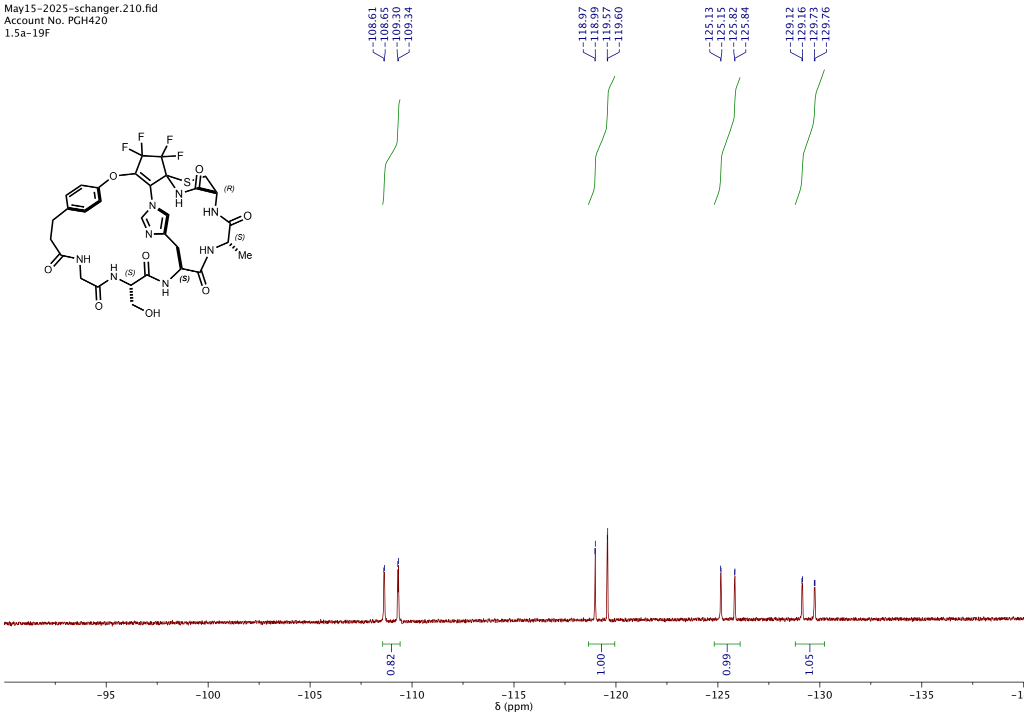

Polycycle 1k.

^{1}H NMR (600 MHz, DMSO)

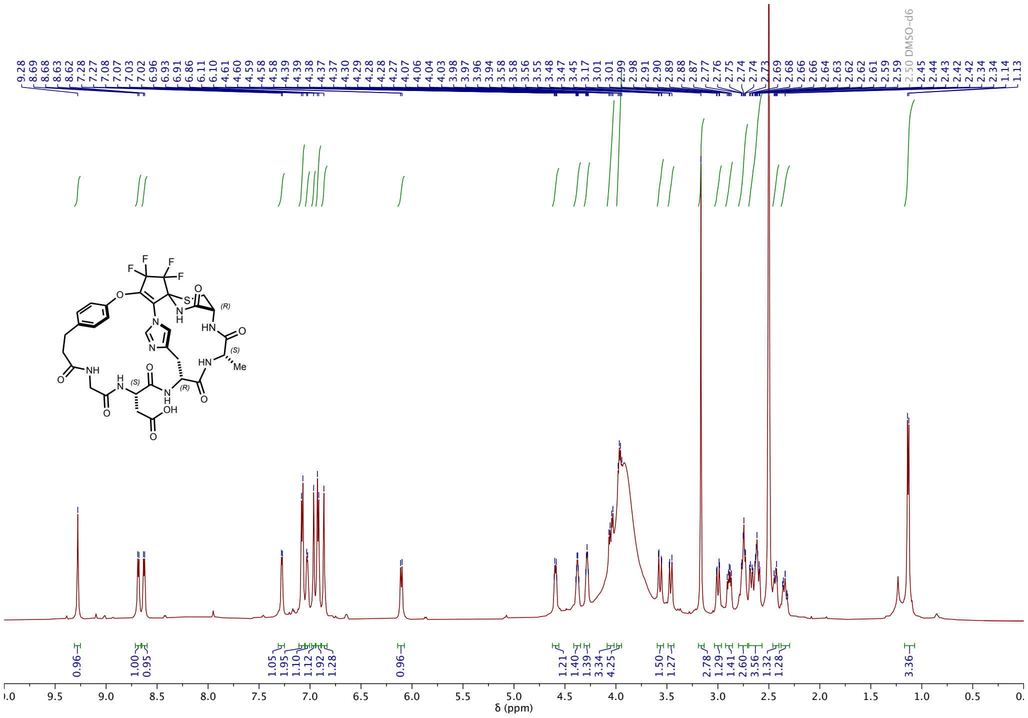

^{13}C NMR (151 MHz, DMSO)

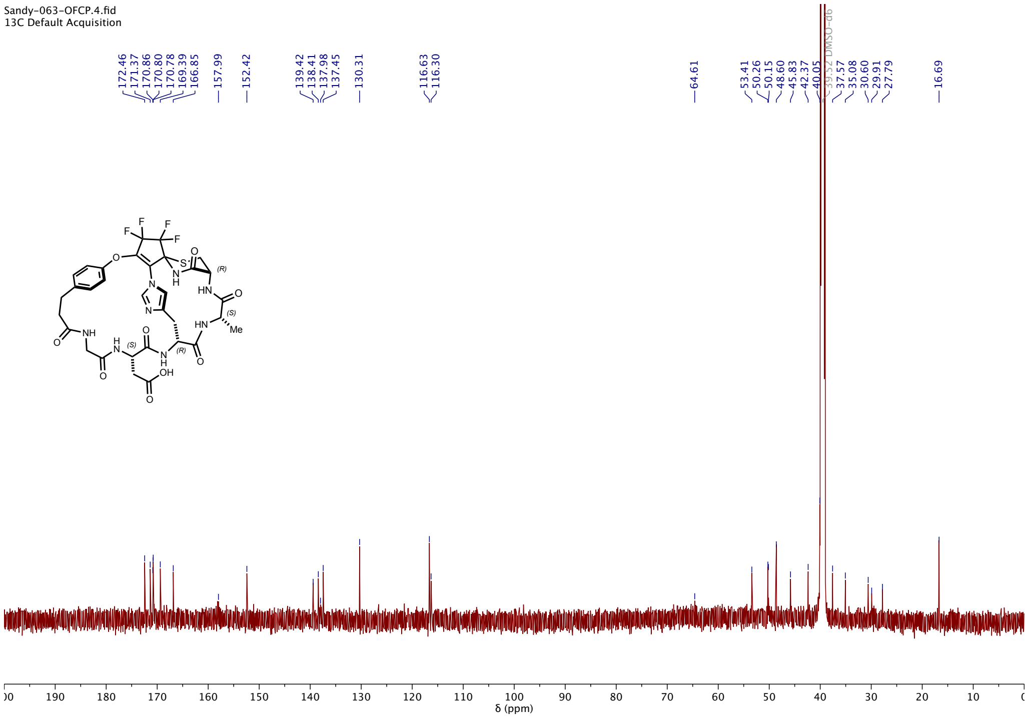

^{19}F NMR (565 MHz, DMSO)

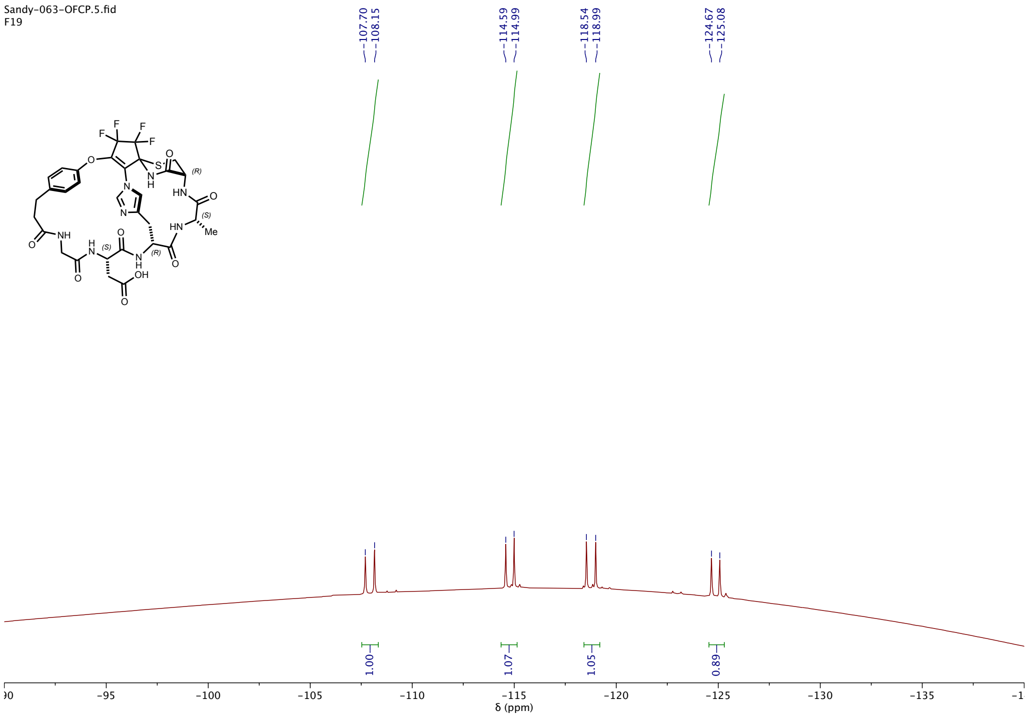

Polycycle 1r.

^{1}H NMR (500 MHz, DMSO)

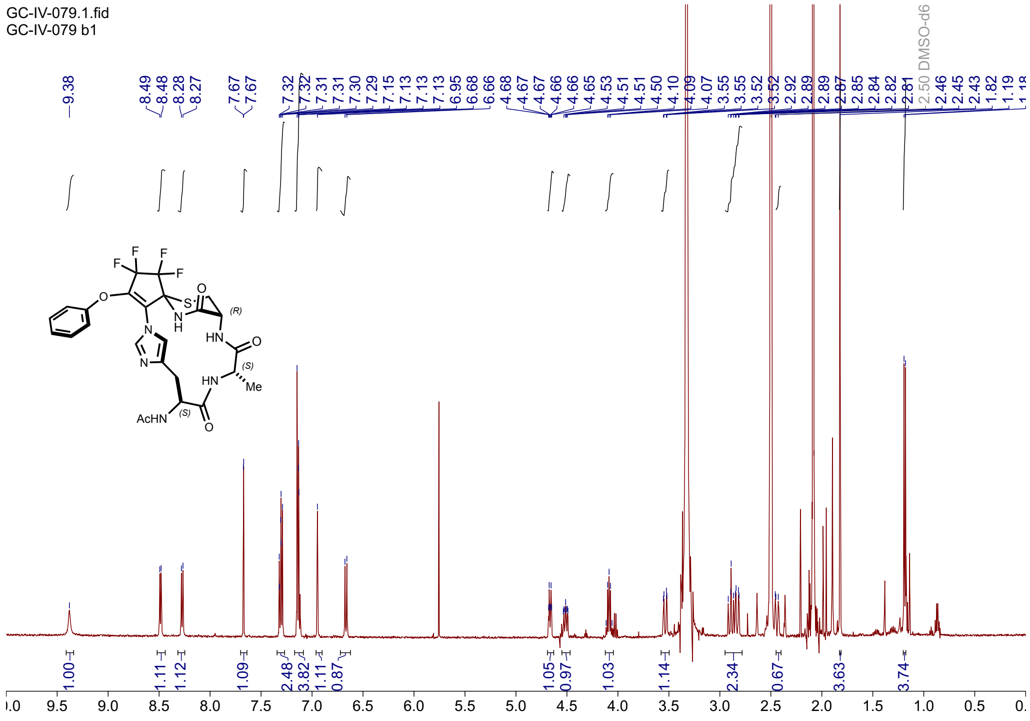

^{13}C NMR (151 MHz, DMSO)

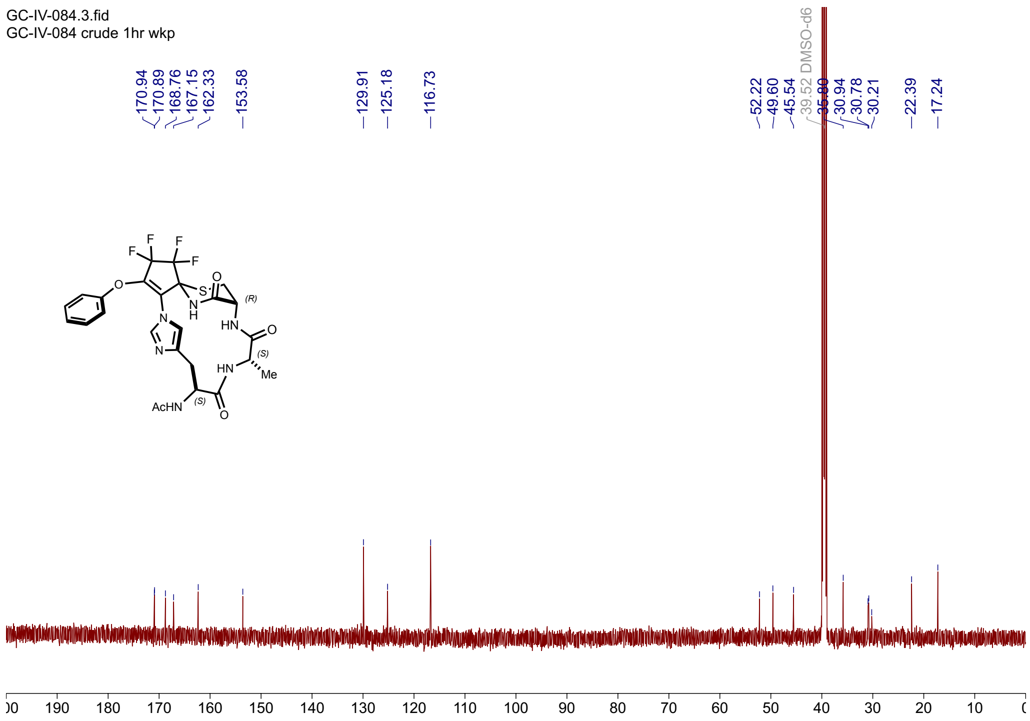

^{19}F NMR (565 MHz, DMSO)

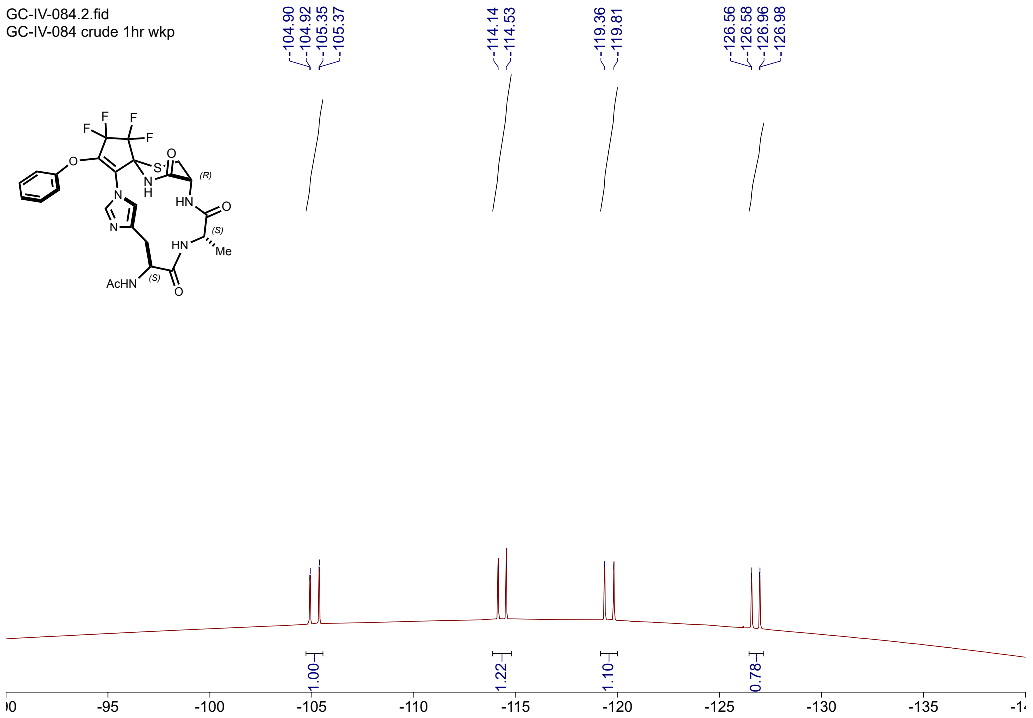

Polycycle 1s.

^{1}H NMR (500 MHz, MeOD)

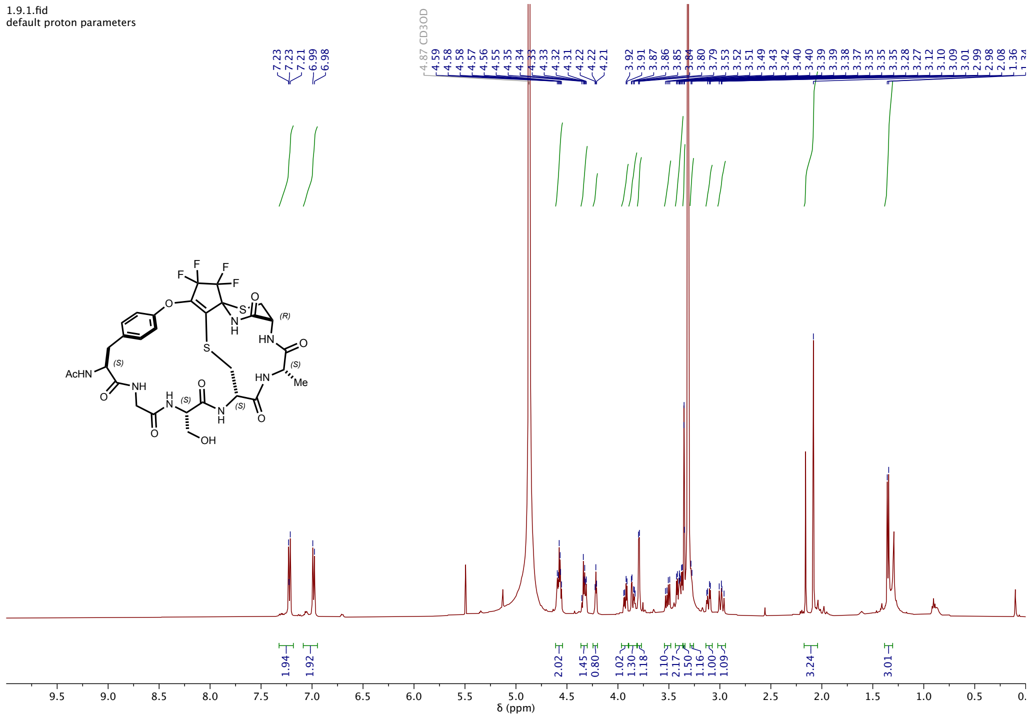

^{13}C NMR (126 MHz, MeOD)

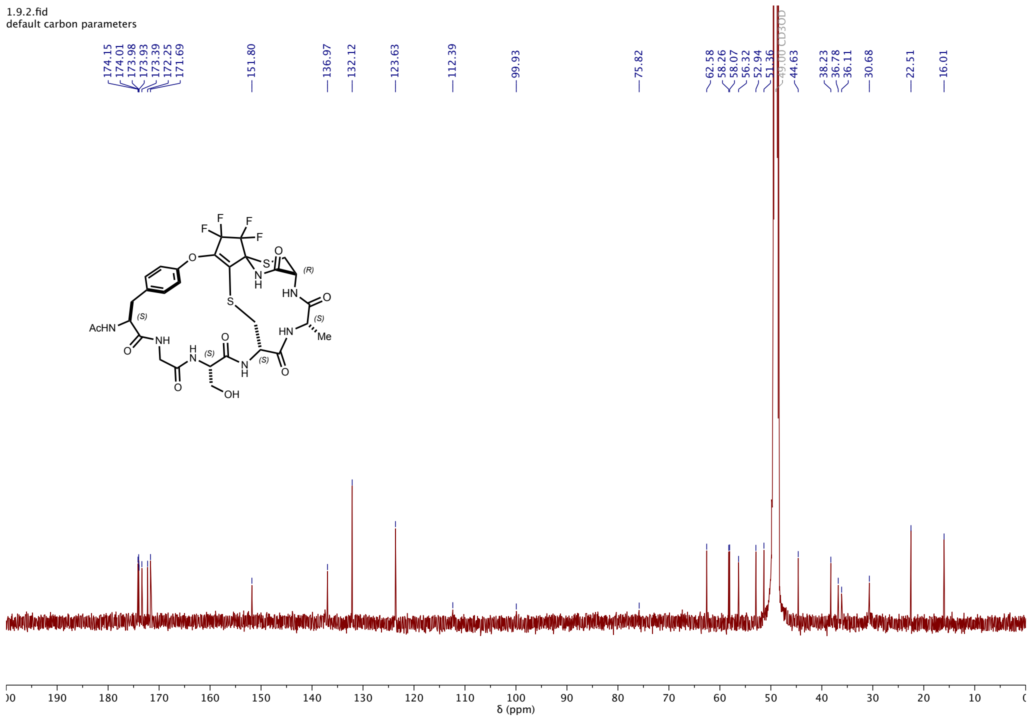

^{19}F NMR (376 MHz, MeOD)

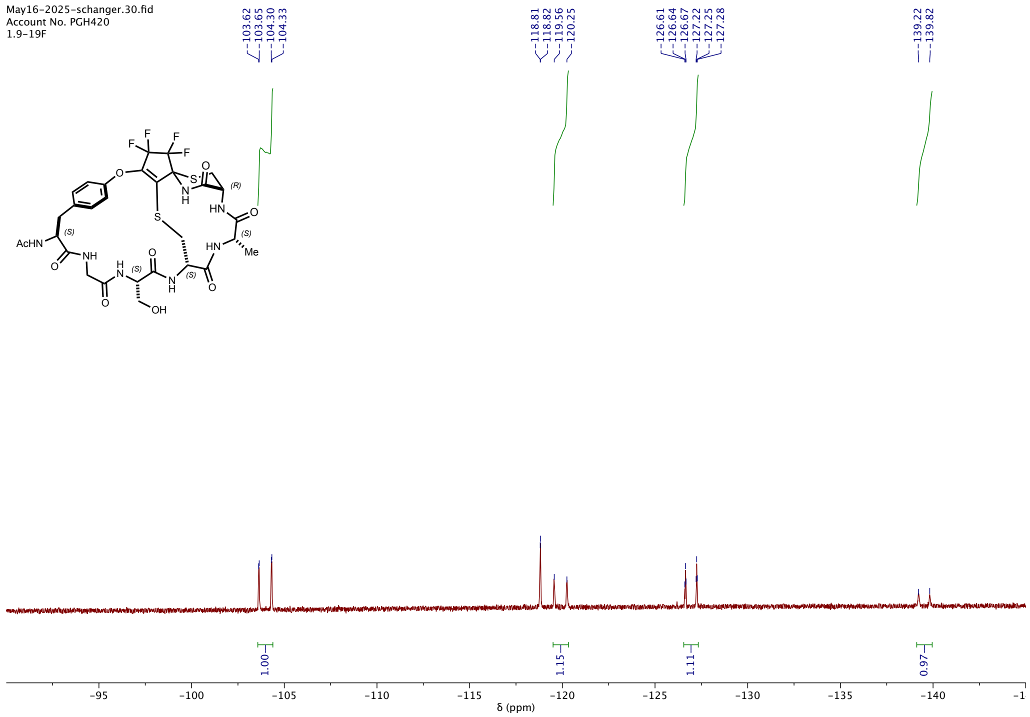

Polycycle 1t.

^{1}H NMR (500 MHz, DMSO)

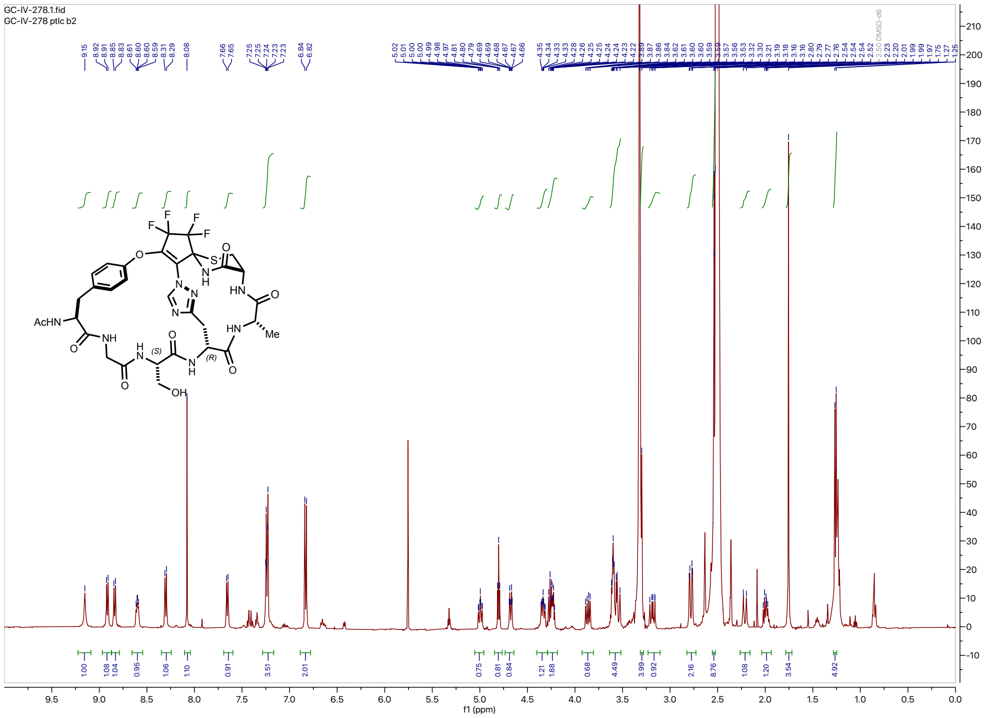

^{13}C NMR (126 MHz, DMSO)

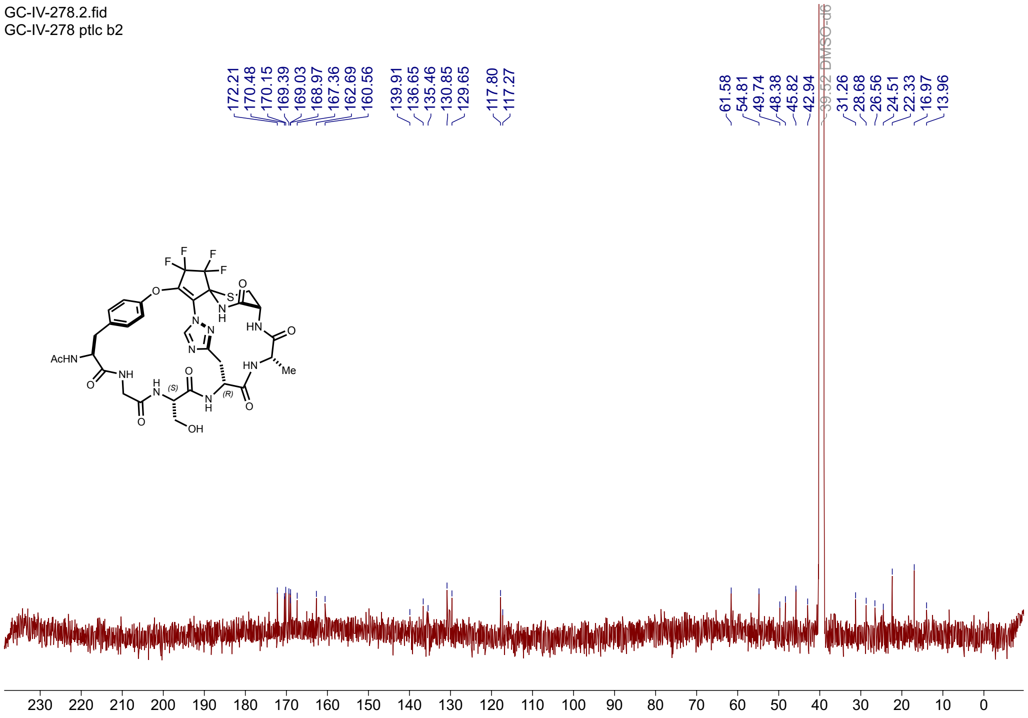

^{19}F NMR (565 MHz, DMSO)

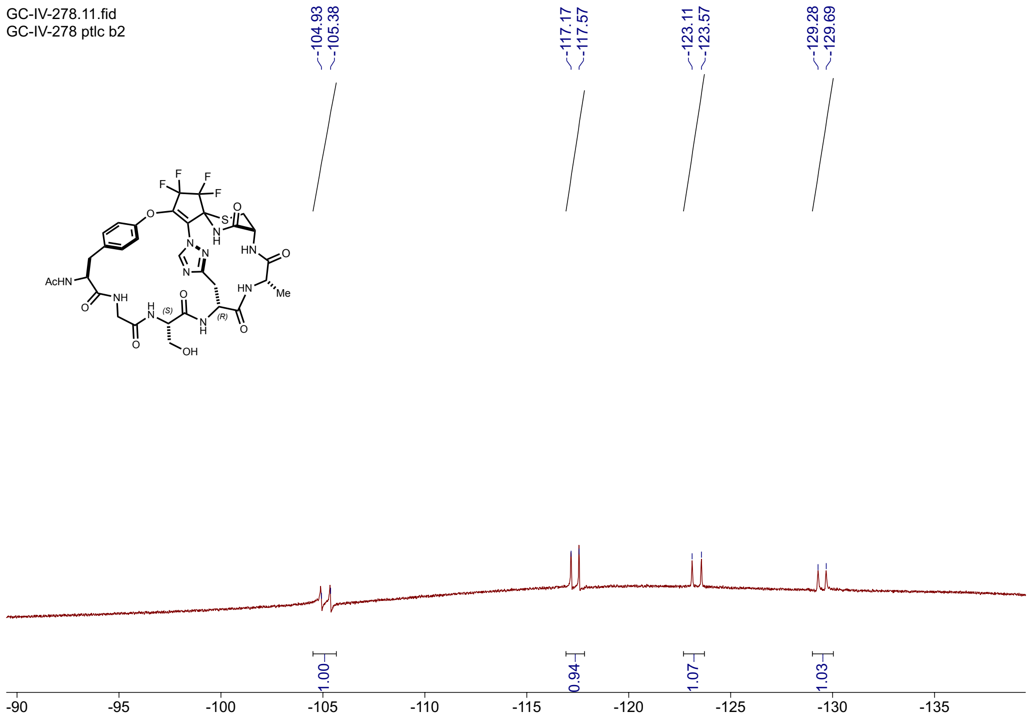

References

(1) Mendoza, A.; Bernardino, S. J.; Dweck, M. J.; Valencia, I.; Evans, D.; Tian, H.; Lee, W.; Li, Y.; Houk, K. N.; Harran, P. G. Cascade Synthesis of Fluorinated Spiroheterocyclic Scaffolding for Peptidic Macrobicycles. *Journal of the American Chemical Society* **2023**, *145* (29), 15888-15895. DOI: 10.1021/jacs.3c03071.

(2) Tsunemi, T.; Bernardino, S. J.; Mendoza, A.; Jones, C. G.; Harran, P. G. Syntheses of Atypically Fluorinated Peptidyl Macrocycles through Sequential Vinylic Substitutions. *Angewandte Chemie International Edition* **2020**, *59* (2), 674-678. DOI: https://doi.org/10.1002/anie.201910136.

(3) Neumann, K.; Farnung, J.; Baldauf, S.; Bode, J. W. Prevention of aspartimide formation during peptide synthesis using cyanosulfurylides as carboxylic acid-protecting groups. *Nature Communications* **2020**, *11* (1), 982. DOI: 10.1038/s41467-020-14755-6.

(4) Zhao, F.; Mazis, G.; Yi, F.; Lotti, J. S.; Layeux, M. S.; Schultz, E. P.; Bunch, L.; Hansen, K. B.; Clausen, R. P. Discovery of (R)-2-amino-3-triazolpropanoic acid derivatives as NMDA receptor glycine site agonists with GluN2 subunit-specific activity. *Frontiers in Chemistry* **2022**, *Volume 10 - 2022*, Original Research. DOI: 10.3389/fchem.2022.1008233.

(5) Case, D. A.; Cheatham III, T. E.; Darden, T.; Gohlke, H.; Luo, R.; Merz Jr., K. M.; Onufriev, A.; Simmerling, C.; Wang, B.; Woods, R. J. The Amber biomolecular simulation programs. *J Comput Chem* **2005**, *26* (16), 1668-1688. DOI: https://doi.org/10.1002/jcc.20290.

(6) He, X.; Man, V. H.; Yang, W.; Lee, T.-S.; Wang, J. A fast and high-quality charge model for the next generation general AMBER force field. *The Journal of Chemical Physics* **2020**, *153* (11). DOI: 10.1063/5.0019056 (acccessed 11/24/2025).

(7) Wang, S.; Witek, J.; Landrum, G. A.; Riniker, S. Improving Conformer Generation for Small Rings and Macrocycles Based on Distance Geometry and Experimental Torsional-Angle Preferences. *Journal of Chemical Information and Modeling* **2020**, *60* (4), 2044-2058. DOI: 10.1021/acs.jcim.0c00025.

(8) Bento, A. P.; Hersey, A.; Félix, E.; Landrum, G.; Gaulton, A.; Atkinson, F.; Bellis, L. J.; De Veij, M.; Leach, A. R. An open source chemical structure curation pipeline using RDKit. *Journal of Cheminformatics* **2020**, *12* (1), 51. DOI: 10.1186/s13321-020-00456-1.

(9) Wang, J.; Wang, W.; Kollman, P. A.; Case, D. A. Antechamber: an accessory software package for molecular mechanical calculations. *J. Am. Chem. Soc* **2001**, *222* (1), 2001.

(10) Berendsen, H. J.; Postma, J. v.; Van Gunsteren, W. F.; DiNola, A.; Haak, J. R. Molecular dynamics with coupling to an external bath. *The Journal of chemical physics* **1984**, *81* (8), 3684-3690.

(11) Zhang, Z.; Liu, X.; Yan, K.; Tuckerman, M. E.; Liu, J. Unified efficient thermostat scheme for the canonical ensemble with holonomic or isokinetic constraints via molecular dynamics. *The Journal of Physical Chemistry A* **2019**, *123* (28), 6056-6079.

(12) JP, C. R. Numerical integration of the Cartesian equations of motion of a system with constraints: molecular dynamics of n-alkanes. *Journal of Computational Physics* **1977**, *23*, 15.

(13) Rodriguez, A.; Laio, A. Clustering by fast search and find of density peaks. *Science* **2014**, *344* (6191), 1492-1496.

(14) Rodriguez, A.; d'Errico, M.; Facco, E.; Laio, A. Computing the Free Energy without Collective Variables. *J Chem Theory Comput* **2018**, *14* (3), 1206-1215. DOI: 10.1021/acs.jctc.7b00916.
(15) Dai, B.; Chen, J.-N.; Zeng, Q.; Geng, H.; Wu, Y.-D. Accurate Structure Prediction for Cyclic Peptides Containing Proline Residues with High-Temperature Molecular Dynamics. *The Journal of Physical Chemistry B* **2024**, *128* (30), 7322-7331. DOI: 10.1021/acs.jpcb.4c02004.
(16) Kabsch, W. XDS. *Acta Crystallographica Section D* **2010**, *66* (2), 125-132. DOI: doi:10.1107/S0907444909047337.
(17) Sheldrick, G. M. *Acta. Cryst.* **2015**, *C71*, 3-8. DOI: doi:10.1107/S2053229614024218.
(18) Sheldrick, G. M. *Acta. Cryst.* **2015**, *A71*, 3-8. DOI: doi:10.1107/S2053273314026370.
(19) Groom, C. R.; Bruno, I. J.; Lightfoot, M. P.; Ward, S. C. The Cambridge Structural Database. *Acta Crystallographica Section B* **2016**, *72* (2), 171-179. DOI: doi:10.1107/S2052520616003954.
